# Synthesis and Biological Evaluation of Curvularin-Type Derivatives with Potential Anti-Inflammatory, Anticancer, and Antimicrobial Activities

**DOI:** 10.3390/molecules31061061

**Published:** 2026-03-23

**Authors:** Kyung Hee Kim, Tai Kyoung Kim, Ju-Mi Hong, Jin A Kim, Min Ju Kim, Jin-Hyoung Kim, Joung Han Yim, Il-Chan Kim, Se Jong Han

**Affiliations:** 1 Division of Life Sciences, Korea Polar Research Institute, Incheon 21990, Republic of Korea; happy_vu@naver.com (K.H.K.); wnal5555@kopri.re.kr (J.-M.H.); kimja611@kopri.re.kr (J.A.K.); mjkim1113@kopri.re.kr (M.J.K.); kimjh@kopri.re.kr (J.-H.K.); 2 CRYOTECH Inc., 2F-211-3, 71 Mieumsandan 5-ro 41beon-gil, Gangseo-gu, Busan 46744, Republic of Korea; tkkim@cryotech.co.kr; 3 Department of Marine Sciences, Incheon National University, Incheon 22012, Republic of Korea; 4 Department of Polar Science, University of Science and Technology, Daejeon 34113, Republic of Korea

**Keywords:** curvularin analogues, anti-inflammatory, anticancer, antimicrobial, *Staphylococcus aureus*, *Candida albicans*

## Abstract

Curvularins, a class of macrocyclic lactones, have cytotoxic, antimicrobial, and anti-inflammatory properties. Curvularin, a 12-membered macrolactone, was used as a scaffold to design and synthesize structurally modified analogues to investigate structure–activity relationships and improve biological efficacy. Three series of curvularin-based analogues, Cur-5H-OMe, Cur-4P-OMe, and Cur-OMe, were synthesized with the same core structure but different substituent sizes and positions. Nine representative derivatives were evaluated for anti-inflammatory, anticancer, antibacterial, and antifungal activities. In LPS-stimulated RAW 264.7 macrophages, most compounds inhibited nitric oxide (NO) production in a concentration-dependent manner but exhibited cytotoxicity at high concentrations. Cytotoxicity assays against HaCaT cells and human cancer cell lines (HCT116, HeLa, and A375) revealed limited selectivity toward cancer cells. Antimicrobial evaluation indicated selective activity against the Gram-positive bacteria, *Staphylococcus aureus*. Compound **23** exhibited superior antibacterial potency compared with kanamycin and notable antifungal activity against *Candida albicans*. This study provides a versatile synthetic platform and identifies key structural features of curvularin derivatives, demonstrating their potential as anti-inflammatory and antimicrobial lead compounds.

## 1. Introduction

The exploration of extreme environments, particularly in polar marine ecosystems, has revealed several microorganisms that were previously unknown [1,2,3]. Among these, marine-derived fungi have emerged as a prolific and promising source of structurally unique and biologically active secondary metabolites [4,5]. Unlike terrestrial and mesophilic fungal species, marine psychrophilic fungi have adapted to survive and thrive under extreme environmental stressors, such as sub-zero temperatures, high salinity, and limited nutrient availability. These harsh conditions have driven the evolution of distinct metabolic pathways that enable the production of novel bioactive compounds [6,7,8]. Consequently, psychrophilic fungi have attracted growing interest in the fields of natural product chemistry, biotechnology, and pharmaceutical research.

Marine fungi isolated from polar environments are particularly noteworthy for their ability to biosynthesize metabolites with both unprecedented chemical scaffolds and a wide range of physiological functions. Many of these compounds have demonstrated antimicrobial, antifungal, anticancer, and anti-inflammatory properties [9,10,11,12]. Such characteristics render polar marine fungi an invaluable and relatively untapped reservoir for drug discovery and development. The fungi have biosynthetic gene clusters that differ significantly from those of more commonly studied fungi, opening new avenues for identifying structurally novel compounds with desirable pharmacological properties. Moreover, the increasing global demand for alternative therapies to treat chronic diseases, such as inflammation-related disorders, has further underscored the need to explore new and sustainable biological resources [13,14,15].

Among the marine-derived psychrophilic fungi investigated by our research group, the strain *Penicillium* sp. SF-5859 has shown particularly promising potential [16]. This strain was isolated from a marine sponge collected in the frigid waters of the Antarctic Ocean, an environment characterized by extreme cold and ecological isolation. During preliminary studies, this fungal isolate was found to produce a range of curvularin-type secondary metabolites. Curvularins are a class of macrocyclic lactones known for their cytotoxic, antimicrobial, and anti-inflammatory properties. In this case, the secondary metabolites isolated from *Penicillium* sp. SF-5859 demonstrated strong anti-inflammatory activity, particularly through the suppression of inflammatory mediators such as nitric oxide (NO) and prostaglandin E2 (PGE2).

Given the demonstrated efficacy of the natural curvularin-type metabolites in reducing key inflammatory markers, expanding upon this discovery through chemical modification is crucial. Derivatives of natural products often display improved pharmacokinetic profiles, enhanced potency, and increased selectivity compared with their parent compounds. Therefore, in this study, we aimed to synthesize a series of curvularin-based analogues to investigate the relationship between molecular structure and anti-inflammatory activity. Moreover, we explored how specific functional group modifications affect the biological activity of these compounds.

## 2. Results and Discussion

### 2.1. Synthesis of Curvularin Analogues

**Scheme 1:**
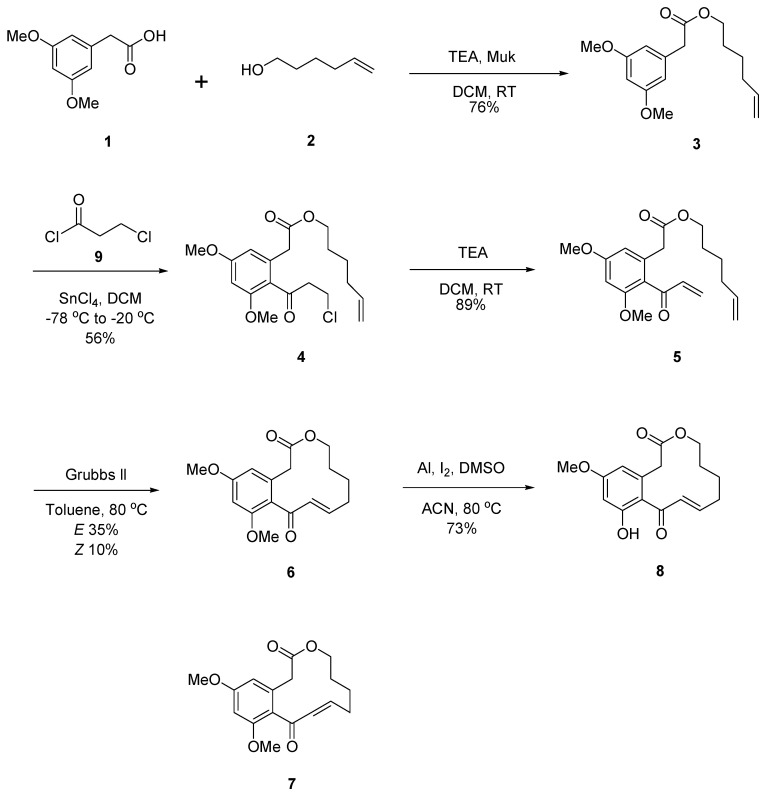
Schematic outlining the preparation of Cur-5H-OMe2-F1 (**6**), Cur-5H-OMe2-F2 (**7**), and Cur-5H-OMe1 (**8**). The Supplementary Materials section describes the synthesis and properties of the compounds.

**Scheme 2:**
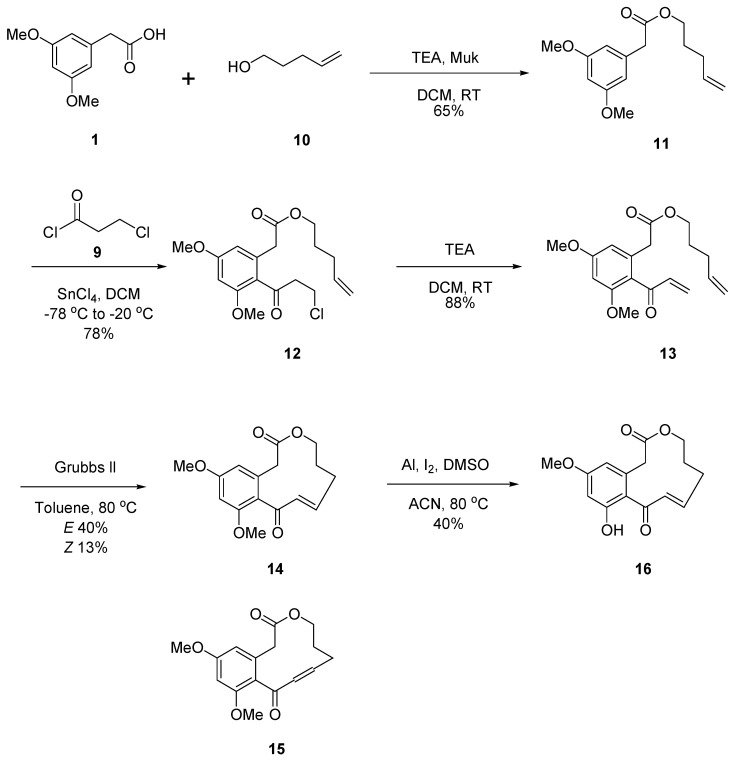
Schematic outlining the preparation of Cur-4P-OMe2-F1 (**14**), Cur-4P-OMe2-F2 (**15**), and Cur-4P-OMe1 (**16**). The Supplementary Materials section describes the synthesis and properties of the compounds.

**Scheme 3:**
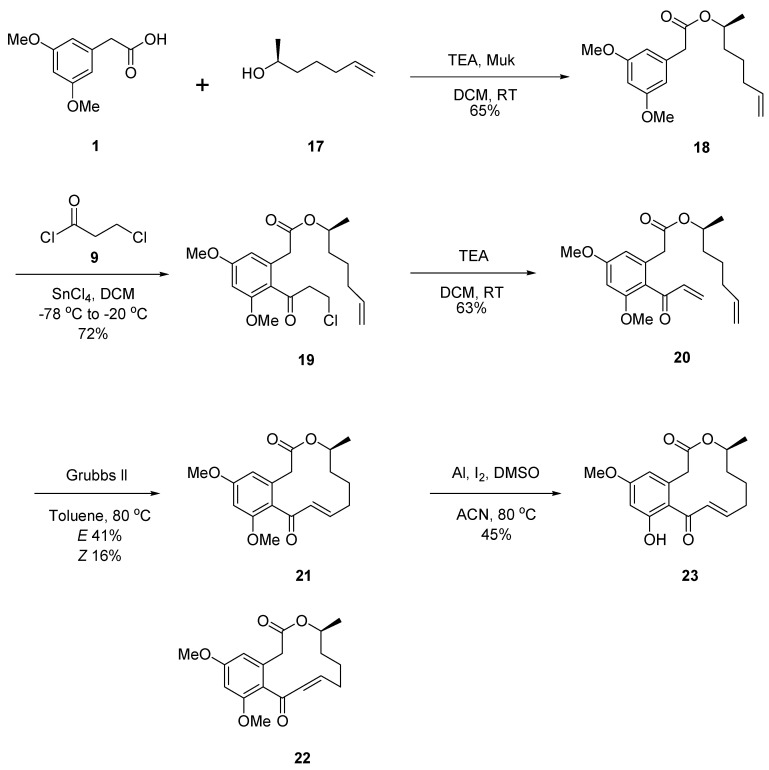
Schematic outlining the preparation of Cur-OMe2-F1 (**21**), Cur-OMe2-F2 (**22**), and Cur-OMe1 (**23**). The Supplementary Materials section describes the synthesis and properties of the compounds.

To systematically investigate the structure–activity relationships (SARs) of curvularin, three series of analogues, namely Cur-5H-OMe (**8**), Cur-4P-OMe (**16**), and Cur-OMe (**23**), were synthesized. The synthesis of all three series followed a unified, convergent strategy comprising esterification, Friedel–Crafts acylation, dehydrohalogenation, ring-closing metathesis (RCM), and selective demethylation (Scheme 1, Scheme 2 and Scheme 3).

Initially, the esterification of 3,5-dimethoxyphenylacetic acid (**1**) with the respective alcohols (**2**, **10**, **17**) was attempted using DCC and DMAP. However, the complete removal of the dicyclohexylurea (DCU) byproduct posed difficulty, which reduced the overall purification efficiency [17]. By switching to the Mukaiyama reagent (2-chloro-1-methylpyridinium iodide) in the presence of triethylamine (TEA), the esterification proceeded smoothly [18]. This optimized condition successfully afforded the ester intermediates **3** (76%), **11** (65%), and **18** (65%).

The ester intermediates were subsequently subjected to Friedel–Crafts acylation with 3-chloropropanoyl chloride (**9**). For this transformation, SnCl_4_ was specifically selected as a mild Lewis acid catalyst; unlike stronger Lewis acids (such as AlCl_3_), SnCl_4_ efficiently promotes the acylation of the highly electron-rich 3,5-dimethoxyphenyl ring while preventing unwanted side reactions, such as the cleavage of the methyl ether groups (demethylation) [19,20]. This optimized acylation yielded the acylated compounds **4** (56%), **12** (78%), and **19** (72%). Following this acylation, dehydrohalogenation was performed by treating these compounds with TEA, which successfully yielded the key diene intermediates **5** (89%), **13** (88%), and **20** (63%) ready for cyclization.

The RCM step was extensively optimized using diene **20** as a model. Notably, rigorous degassing (argon bubbling for 2 h) and highly diluted conditions (4 mM in toluene) at 80 °C with 20 mol% of Grubbs II catalyst were critical to prevent intermolecular oligomerization and overcome the entropic penalties of medium-to-large macrocycle formation. Under these optimized conditions, the RCM of the dienes provided the corresponding macrolactones as mixtures of E/Z diastereomers: the 12-membered rings **6** (E, 35%) and **7** (Z, 10%); the 11-membered rings **14** (E, 40%) and **15** (Z, 13%); and the 12-membered rings **21** (E, 41%) and **22** (Z, 16%).

Although these conditions were heavily optimized, the overall yields remained moderate (45–57%). This can be attributed to the difficulty in the formation of medium-sized (11- to 12-membered) rings, which inherently suffer from high transannular ring strain and severe entropic penalties [21]. Furthermore, despite the highly diluted environment, unavoidable competitive intermolecular oligomerization, along with potential catalyst deactivation caused by the chelation of Lewis basic oxygen atoms (such as ester, ketone, and methoxy groups) present in the diene substrates, likely limited the final RCM efficiency [22,23].

Regarding stereoselectivity, the RCM reactions predominantly yielded the thermodynamically more stable E-isomers across all series. Interestingly, during structural characterization, we observed an isomeric phase transition for the purified Z-isomers (**7**, **15**, and **22**). When dissolved in nuclear magnetic resonance (NMR) solvents, these Z-isomers slowly and spontaneously isomerized to their respective E-isomers over time, further confirming the strong thermodynamic preference for the E-configuration in these curvularin scaffolds.

Finally, to generate the mono-methoxy target derivatives, the E-isomer macrolactones (**6**, **14**, and **21**) were subjected to selective demethylation using aluminum powder, iodine, and dimethyl sulfoxide (DMSO) in acetonitrile [19]. This final step successfully afforded the newly synthesized derivatives **8** (73%), **16** (40%), and **23** (45%).

The Cur-5H-OMe, Cur-4P-OMe, and Cur-OMe analogues were designed to retain the 12-membered macrolactone skeleton, the principal physiologically active framework of curvularin, while modulating electronic and steric properties by varying substituent position and sizes. Curvularin derivatives exhibit a range of biological activities, including antibacterial [24], antifungal [25], anti-inflammatory [16,26,27], anticancer [28], and cytoprotective activity [29]. The results of this synthesis are significant because they demonstrate the successful preparation of various curvularin analogues through a unified reaction, building on previous studies [19,30,31]. Although the process is sensitive to the conditions of each step, it provides high yield and selectivity and establishes a synthetic paradigm useful for constructing future bioactivity screening libraries. This synthetic route provides a stable platform for expanding the activity range of curvularin derivatives and conducting precise SAR studies.

### 2.2. Anti-Inflammatory Activity

**Figure 1:**
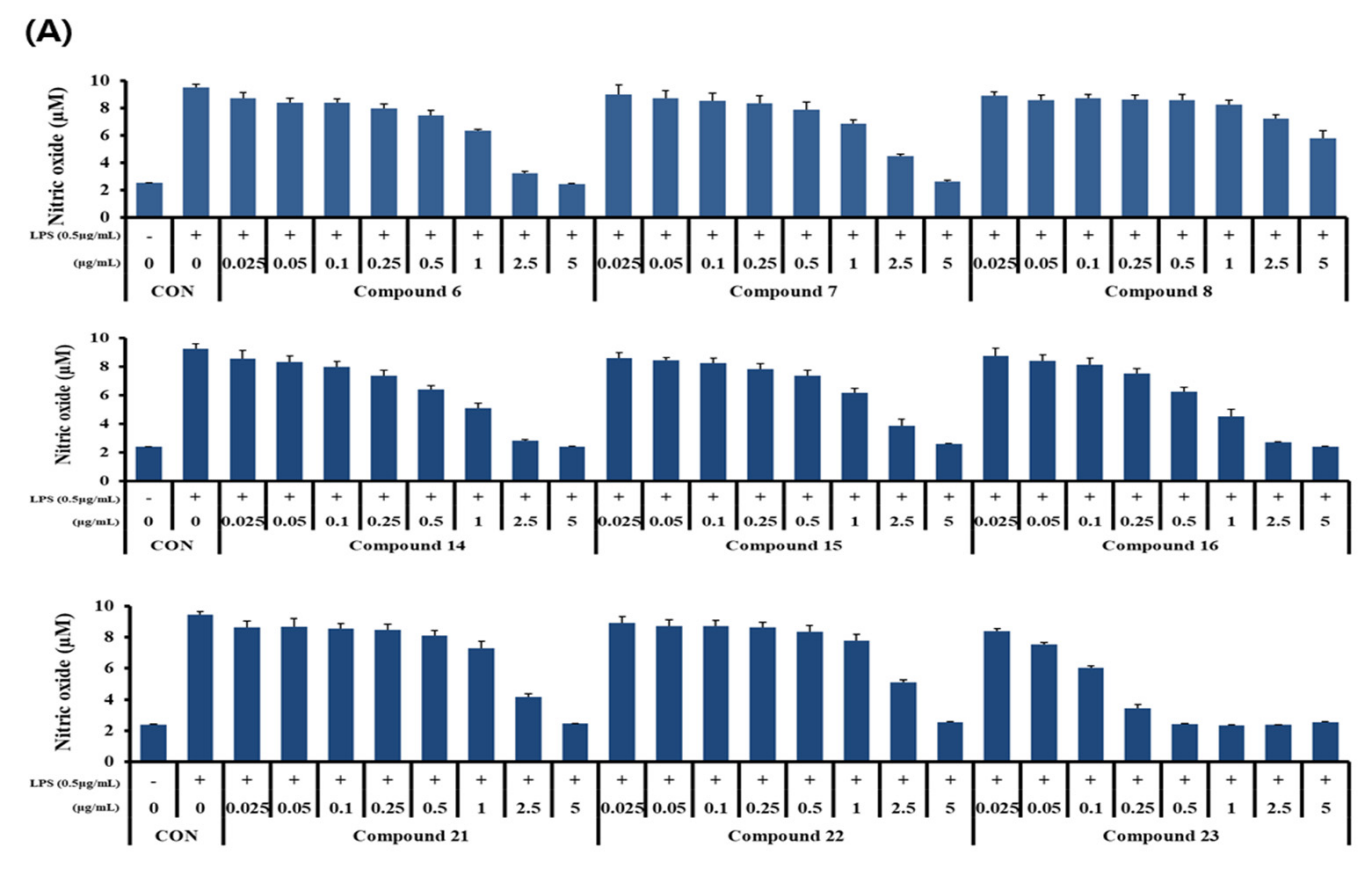

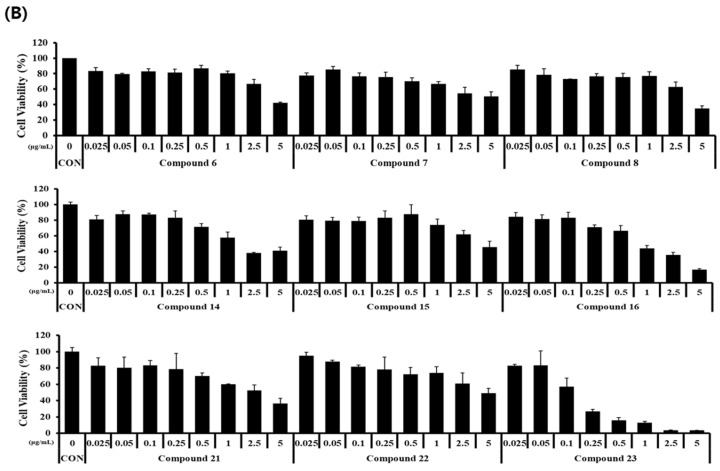
Effects of curvularin derivatives on (**A**) LPS-induced nitric oxide (NO) production and (**B**) cell viability in RAW 264.7 macrophages. NO levels were quantified using the Griess assay, and viability was measured using the MTT assay. Data are presented as mean ± SD (*n* = 3).

Inhibition of nitric oxide (NO) production in LPS-induced RAW 264.7 cells was measured to determine the nitric oxide activity of nine derivatives (compounds **6**–**8**, **14**–**16**, and **21**–**23**). The concentrations of the nine derivatives were 0, 0.025, 0.05, 0.1, 2.5, and 5 μg/mL. Nitrite concentrations were measured using the Griess reaction and were found to be significantly increased in LPS-stimulated RAW 264.7 cells. However, NO production was inhibited in a concentration-dependent manner by the compounds synthesized in this study, except for compound **8** (Figure 1A). Although the synthesized compounds reduced NO production at high concentrations, a corresponding decrease in cell viability was observed, indicating that the NO reduction at these doses was likely due to cytotoxicity (Figure 1B). Compound **7** exhibited an anti-inflammatory effect, inhibiting NO production by more than 70% while maintaining cell survival by more than 50% even at high concentrations.

The anti-inflammatory potential of curvularin derivatives has been previously explored [16,26,29,32]. In the present study, most derivatives inhibited NO production in LPS-induced RAW 264.7 cells. However, to distinguish true anti-inflammatory activity from cytotoxicity mediated NO reduction, a concern raised by the correlation between NO inhibition and cell death at high concentrations, we calculated the selectivity index (SI) for each compound (Table 1).

While some derivatives (e.g., compounds **16** and **23**) exhibited low SI values (<1.0), indicating that their apparent inhibitory effects were primarily due to cytotoxicity, compounds **6**, **7**, and **15** demonstrated a favourable safety profile with SI values greater than 2.0. Notably, compound **6** (SI = 2.27) and compound **7** (SI = 2.20) inhibited NO production with half-maximal inhibitory concentration (IC_50_ values) of 1.83 μg/mL and 2.32 μg/mL, respectively, which were significantly lower than their cytotoxic concentrations (IC_50_ > 4.0 μg/mL). These quantitative results suggest that compounds **6** and **7** exhibit genuine anti-inflammatory potential and warrant further development as therapeutic agents.

Notably, from an SAR perspective, compound **7** (12-membered macrocycle with a Z-alkene configuration and di-methoxy substituents) exhibited the most favourable anti-inflammatory profile (high NO inhibition with low cytotoxicity). This suggests that the increased conformational flexibility of the 12-membered ring, combined with the specific spatial arrangement dictated by the Z-geometry, may facilitate selective binding to inflammatory targets such as iNOS [33,34]. Furthermore, retaining the di-methoxy groups (avoiding free phenols) appears crucial for mitigating generalized cytotoxicity, thereby widening the therapeutic window for anti-inflammatory applications [35].

### 2.3. Anticancer Activity

**Figure 2:**
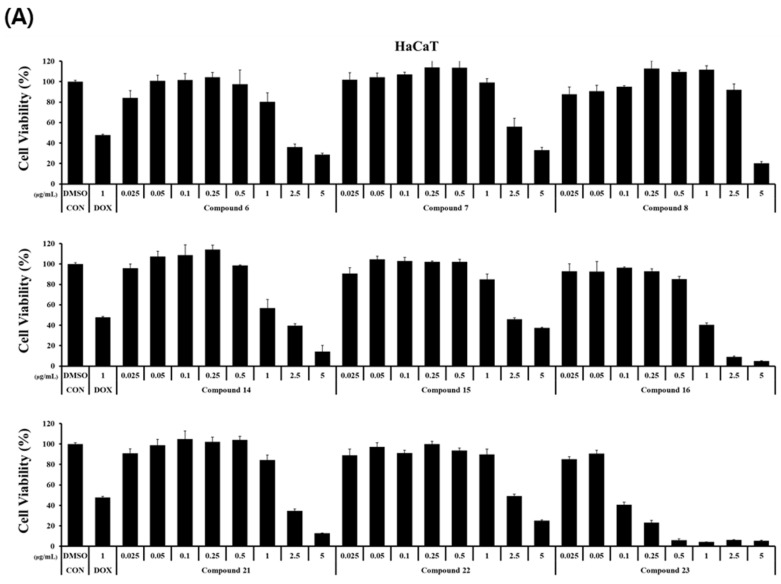

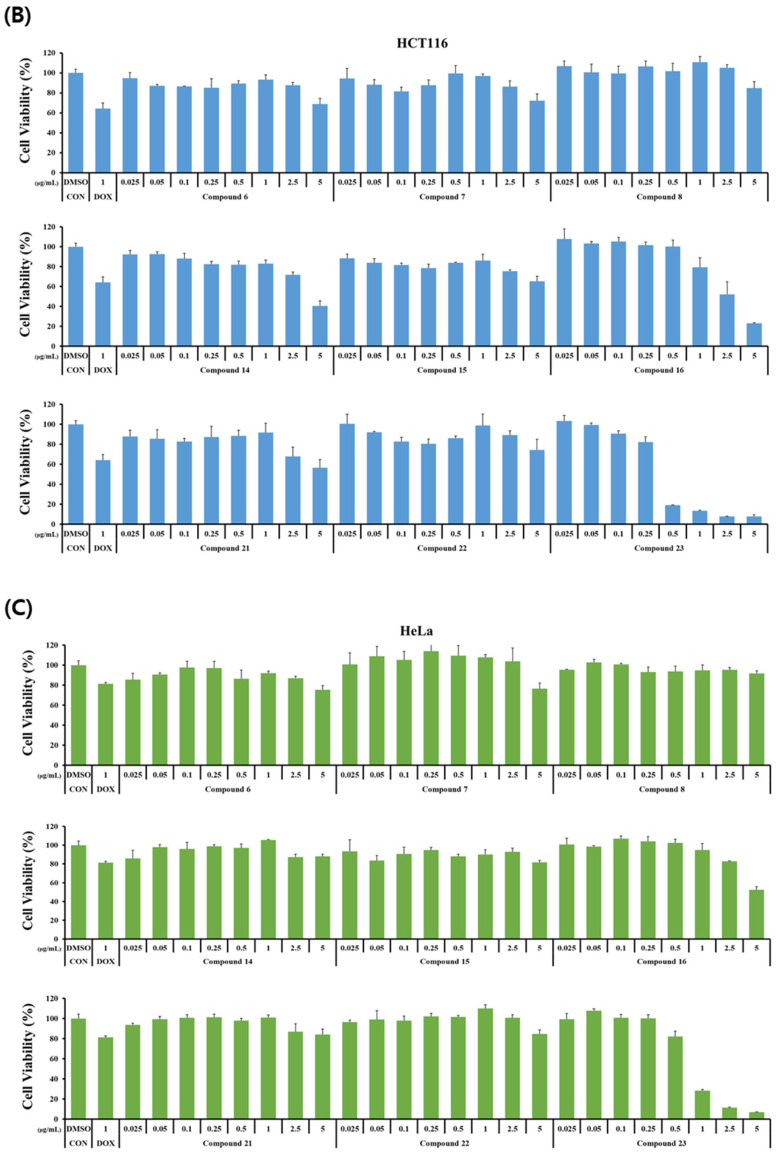

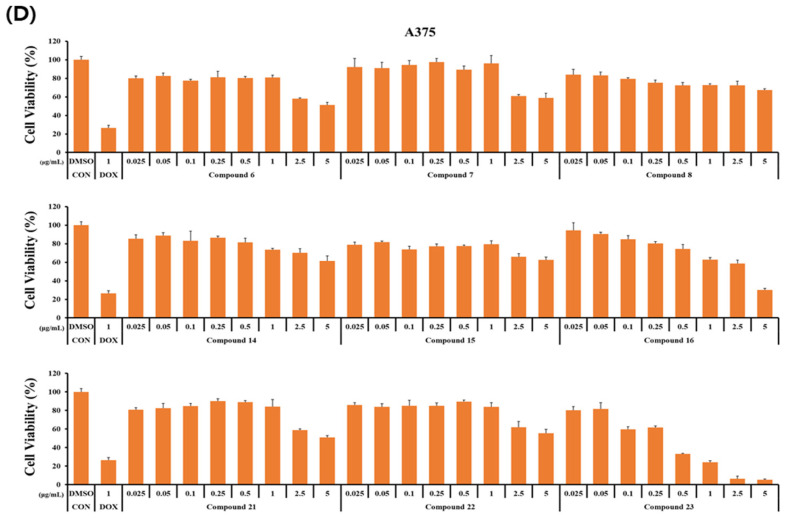
Cytotoxic effects of curvularin derivatives on four human cell lines. Cells were treated for 24 h with compounds (**6**–**8**, **14**–**16**, **21**–**23**) at concentrations of 0.025, 0.05, 0.1, 0.25, 0.5, 1, 2.5, and 5 μg/mL. Cell viability was assessed after treatment; the data represent the mean ± SD values of three independent experiments: (**A**) HaCaT keratinocytes, (**B**) HCT116 colorectal carcinoma cells, (**C**) HeLa cervical carcinoma cells, and (**D**) A375 melanoma cells.

The cytotoxicity of the synthesized curvularin derivatives was investigated on normal human keratinocytes (HaCaT) and three human cancer cell lines (HCT116, HeLa, and A375). Cells were incubated with the nine derivatives at concentrations ranging from 0 to 5 μg/mL for 24 h. The dose–response profiles are presented in Figure 2, and the corresponding half-maximal inhibitory concentration (IC_50_) values are summarized in Table 2.

Most derivatives exhibited weak cytotoxicity against the tested cancer cell lines (Figure 2). This observation is confirmed by the quantitative analysis in Table 2, which indicates that the majority of compounds display IC_50_ values greater than 5.0 μg/mL. While compounds **16** and **23** displayed potent inhibitory effects on cancer cell growth (Figure 2), a comparison with normal HaCaT cells revealed a lack of selectivity.

Specifically, compound **23** exhibited strong cytotoxicity against all cancer cell lines, with IC_50_ values ranging from 0.35 to 0.79 μg/mL (Table 2). However, it was significantly more toxic to normal HaCaT cells (IC_50_ = 0.09 μg/mL). Similarly, compound **16** displayed moderate activity against cancer cells (IC_50_ = 2.68–3.25 μg/mL) but demonstrated higher toxicity toward HaCaT cells (IC_50_ = 0.89 μg/mL).

These findings align with those of previous studies indicating that curvularin derivatives often exhibit a broad spectrum of cytotoxicity, as observed in HT1080, T46D, A2780S [36], A549, HeLa, MCF-7 [25], and other human tumour cell lines [37]. Although potent cytotoxicity has been reported repeatedly for curvularin analogues, selective activity against specific cancer cell lines remains limited. Therefore, our data suggest that while compounds **16** and **23** are potent cytotoxic agents, further target-directed design is required to improve their selectivity for cancer cells over normal cells.

The SAR analysis of the anticancer results reveals a clear integration of chemical modification and pharmacological outcome. The notable increase in cytotoxicity observed for compounds **16** and **23** highlights the critical role of the selective demethylation step in our synthetic strategy. The unmasking of a free phenolic hydroxyl group on the benzene ring serves as a structural “on-switch” for cytotoxicity [38]. While the completely methylated precursors were largely inactive against cancer cells, the free phenol likely enhances non-specific interactions with cell membranes, acts as a potent hydrogen bond donor to protein targets, or participates in redox cycling to generate cytotoxic reactive oxygen species (ROS) [39]. However, as this leads to potent, but non-selective, toxicity (affecting normal HaCaT cells as well), future synthetic efforts must focus on modifying the macrocyclic backbone of these mono-hydroxy derivatives to impart target-specific selectivity.

### 2.4. Antibacterial and Antifungal Activity

Cell inhibition against *S. aureus* and *E. coli* was measured at concentrations of 0, 2.5, 25, 50, 100, and 200 μg/mL to determine the antibacterial activity of nine derivatives (compounds **6**–**8**, **14**–**16**, and **21**–**23**). The IC_50_ values revealed no activity against *E. coli*; however, seven derivatives (compounds **7**, **8**, **14**, **16**, **21**, **22**, and **23**) exhibited activity against *S. aureus*. Four derivatives (compounds **8**, **14**, **16**, and **23**) showed minimum inhibitory concentration (MIC) values against *S. aureus* within the tested concentration range. Compound **23** exhibited an IC_50_ value of 8.90 ± 2.28 μg/mL, which was stronger than that of kanamycin (14.97 ± 6.17 μg/mL), suggesting the potential for antibiotic activity (Table 3).

The growth inhibition of *C. albicans* cells was measured at concentrations of 0, 2.5, 25, 50, 100, and 200 μg/mL to determine the antifungal activity of the nine derivatives. The IC_50_ values confirmed the activity of six derivatives (compounds **7**, **8**, **15**, **16**, **22**, and **23**) against *C. albicans*, whereas MIC values were obtained for three derivatives (compounds **8**, **16**, **23**). Compounds **8** and **23** exhibited low IC_50_ values, indicating the potential for these derivatives to exhibit potent antifungal effects (Table 4).

In tests targeting *S. aureus* and *E. coli*, no significant inhibitory effect was observed against *E. coli*, whereas seven derivatives (**7**, **8**, **14**, **16**, **21**, **22**, **23**) displayed activity against *S. aureus*. This is consistent with previous reports indicating that the curvularin skeleton was more effective at targeting Gram-positive bacteria than Gram-negative bacteria [25]. Previous studies have shown that some curvularin derivatives also displayed activity against *E. coli*; therefore, a detailed investigation is needed to understand how structural differences affect efficacy [36,40,41].

Moreover, antifungal activity against *C. albicans* was confirmed for curvularin and 11-alpha-methoxycurvularin [36,42]. Among the nine compounds synthesized in this study, compounds **8**, **16**, and **23**, each comprising only one O-methyl group on the benzene ring, exhibited relatively higher antibacterial and antifungal activities than the other compounds. This can be attributed to the fact that methylation often reduces the polarity of drug molecules, which can change the binding affinity for specific receptors and reduce toxicity [35]. The presence of two O-methyl groups on the benzene ring appears to reduce toxicity, resulting in lower antibacterial and antifungal activity in the other compounds.

Curvularin derivatives were effective against *B. megaterium*, *M. violaceum*, *S. tritici*, *C. fusca* [43], *H. pylori* [44], *P. capsici* zoospores [45], *M. marinum* [46], *V. alginolyticus*, *V. harveyi* [24], and *M. tuberculosis* [47]. The compounds synthesized in this study also need to be further studied for their efficacy against different types of pathogenic microorganisms. Compound **23**, which has antibacterial and antifungal activity, is considered a promising starting material that can be developed into an antibiotic candidate through structural optimization.

### 2.5. Quantitative Comparison of Activities of Curvularin and Its Derivatives

The physiological activities of curvularin, dehydrocurvularin, and the derivative (i.e., compound **23**) synthesized in this study were quantitatively compared (Table 5). Compound **23** exhibited anti-inflammatory activity with an IC_50_ value of 0.59 μM, which was higher than that of dehydrocurvularin (0.44 μM) but significantly lower than that of curvularin (18.1 μM). Although a direct comparison of anti-cancer activity was difficult, compound **23** exhibited lower IC_50_ values than that previously reported. The compounds synthesized in this study did not show MIC against *E. coli* within the tested concentrations, whereas previous studies have shown that dehydrocurvularin exhibited activity, and compound **23** exhibited better activity against *S. aureus*. Compound **23** exhibited lower MIC against *C. albicans* than that of dehydrocurvularin.

## 3. Materials and Methods

### 3.1. General Experimental Information

Anhydrous solvents and reagents used in this study were purchased from Sigma-Aldrich (St. Louis, MO, USA) and TCI (Tokyo, Japan) and used as directed without further purification. All glassware was completely dried in a drying oven at 80 °C and cooled under dry argon immediately before use. All reactions were performed under argon gas, and solvents and liquid reagents were transferred using syringes. The organic extract was dried over MgSO_4_ and concentrated under reduced pressure using a rotary evaporator. The residual solvent was removed under high vacuum (Vacuubrand RZ 2.5, Wertheim, Germany, 1 × 10^−2^ mbar). TLC was performed using 0.25 mm silica gel plates (Merck, Darmstadt, Germany) and observed under 254 nm ultraviolet light (UV CABINET CN-15.LC, VILBER, Vilber Lourmat, France). Flash column chromatography used silica gel 60 (230–400 mesh, Merck, Darmstadt, Germany) as the stationary phase and a mixture of *n*-hexane and ethyl acetate as the mobile phase. Reversed-phase flash column chromatography was performed using an AI-580S system (Yamazen Science, Inc., Burlingame, CA, USA). C18 resin (ODS-SM, 50 μm, 120 Å) was used as the stationary phase, eluted with a gradient of water and methanol.

The NMR spectra were measured using a Jeol JNM ECP-400 spectrometer (Jeol Ltd., Tokyo, Japan) and a Bruker Avance III HD 600 MHz spectrometer (Bruker Daltonics, Leipzig, Germany), using methanol-d_4_ (CD_3_OD) or chloroform-d_4_ (CDCl_3_) as the solvent. Chemical shifts were set based on internal standards or residual solvent signals (MeOH-d_4_: δ_H_ 3.31/δ_C_ 49.1; CDCl_3_: δ_H_ 7.27/δ_C_ 77.0; tetramethylsilane). Peak splitting patterns were abbreviated as m (multiplets), s (singlets), d (doublets), t (triplets), dd (doublet of doublets), dt (doublet of triplets), td (triplet of doublets), and br (broad). Accurate mass was measured using an AB Sciex Triple TOF 4600 instrument (AB Sciex, Framingham, MA, USA) in direct injection mode.

### 3.2. General Procedures for the Synthesis of Curvularin Derivatives

#### 3.2.1. General Procedure A: Esterification (Step 1)

In a flame-dried round-bottom flask, 3,5-dimethoxyphenylacetic acid (1.0 equiv), the corresponding primary alcohol (e.g., 5-hexen-1-ol, 4-propan-1-ol, or (S)-hept-6-en-2-ol; 1.2 equiv), and 2-chloro-1-methylpyridinium iodide (Mukaiyama reagent, 1.2 equiv) were dissolved in anhydrous DCM. The mixture was stirred at room temperature for 15 min. Triethylamine (TEA, 3.0 equiv) was then added dropwise, and the reaction mixture was stirred at room temperature for 20 h. Subsequently, the mixture was filtered and concentrated under reduced pressure. The crude product was purified by flash column chromatography (hexane/EtOAc) to afford the corresponding ester intermediate.

#### 3.2.2. General Procedure B: Friedel–Crafts Acylation (Step 2)

A flame-dried flask was charged with anhydrous DCM and cooled to −78 °C before adding 3-chloropropanoyl chloride (1.3 equiv). A 1 M solution of SnCl_4_ in DCM (1.3 equiv) was added slowly, and the mixture was stirred for 15 min. The ester intermediate prepared in Step 1 (1.0 equiv) was dissolved in DCM and added dropwise to the reaction mixture at −78 °C. The temperature was gradually raised to −20 °C, and the mixture was stirred for 1.5–2.5 h. The reaction was quenched by pouring the mixture into a separatory funnel containing ice and water. The organic layer was separated, washed successively with saturated aqueous NaHCO_3_ and water, dried over MgSO_4_, filtered, and concentrated. The crude product was purified by flash column chromatography to obtain the acylated intermediate.

#### 3.2.3. General Procedure C: Dehydrohalogenation (Step 3)

The acylated intermediate (1.0 equiv) was dissolved in anhydrous DCM, and TEA (2.0 equiv) was added. The mixture was stirred overnight at room temperature to promote dehydrohalogenation and intramolecular ring-forming rearrangement. The reaction was quenched with saturated aqueous NH_4_Cl and extracted with DCM. The combined organic layers were dried over MgSO_4_, filtered, and concentrated. The concentrate was purified using flash column chromatography (hexane/EtOAc, 8:2) to afford the diene intermediate.

#### 3.2.4. General Procedure D: Ring-Closing Metathesis (Step 4)

In a flame-dried round-bottom flask, the diene compound prepared in Step 3 (1.0 equiv) was dissolved in anhydrous toluene to reach a highly dilute concentration (approx. 4 mM). The solution was thoroughly degassed by bubbling argon gas for 2 h. A solution of Grubbs second-generation catalyst (0.2 equiv, 20 mol%) in a small amount of toluene was added, and the reaction mixture was heated to 80 °C and stirred for 1 h. Upon completion, the mixture was concentrated, pre-adsorbed onto silica gel for dry loading, and purified using an open silica-gel column (hexane/EtOAc eluents) to yield the corresponding metathesis products (*E*/*Z* mixtures).

#### 3.2.5. General Procedure E: Selective Demethylation (Step 5)

The metathesis product (1.0 equiv) was dissolved in anhydrous ACN, followed by the addition of aluminum powder (10.0 equiv) and DMSO (5.0 equiv). Iodine (3.3 equiv) was added in one portion, and the reaction mixture was heated to 80 °C for 30 min. After cooling to room temperature, the mixture was diluted with 2 M aqueous HCl and extracted with ethyl acetate. The combined organic layers were dried over MgSO_4_, filtered, and concentrated. The final crude product was purified via flash column chromatography (hexane/EtOAc, 7:3) to yield the target curvularin analogues.

#### 3.2.6. Synthesis of Hex-5-en-1-yl 2-(3,5-dimethoxyphenyl)acetate (**3**)

General procedure A. TLC R*_f_* = 0.57 (hexane/EtOAc, 7:3). ^1^H NMR (400 MHz, CD_3_OD) δ 6.43 (d, *J* = 2.3 Hz, 1H, H-4; 1H, H-8), 6.37 (t, *J* = 2.3 Hz, 1H, H-6), 5.77 (m, 1H, H-5′), 4.98 (m, 2H, H-6′), 4.93 (m, 2H, H-6′), 4.09 (t, *J* = 6.4 Hz, 2H, H-1′), 3.75 (s, 3H, H-5 OMe; 3H, H-7 OMe), 3.52 (s, 2H, H-2), 2.04 (m, 2H, H-4′), 1.62 (m, 2H, H-2′), 1.40 (m, 2H, H-3′); 13C NMR (100 MHz, CD3OD) δ 173.4, 162.4, 162.4, 139.5, 137.7, 115.2, 108.3, 108.3, 100.0, 65.8, 55.7, 55.7, 42.3, 34.3, 29.1, 26.3; HRESIMS *m*/*z* 279.1596 [M + H]^+^ (calcd for C_16_H_23_O_4_, 279.1596).

#### 3.2.7. Synthesis of Hex-5-en-1-yl 2-(2-(3-chloropropanoyl)-3,5-dimethoxyphenyl)acetate (**4**)

General procedure B. TLC R*_f_* = 0.42 (hexane/EtOAc, 7:3). ^1^H NMR (400 MHz, CDCl_3_) δ 6.41 (d, *J* = 2.3 Hz, 1H, H-6), 6.37 (d, *J* = 2.3 Hz, 1H, H-4), 5.78 (m, 1H, H-5′), 5.00 (m, 2H, H-6′), 4.95 (m, 2H, H-6′), 4.08 (t, *J* = 6.9 Hz, 2H, H-1′), 3.84 (s, 3H, H-5 OMe), 3.82 (s, 3H, H-7 OMe), 3.82 (m, 2H, H-11), 3.67 (s, 2H, H-2), 3.35 (t, *J* = 7.1 Hz, 2H, H-10), 2.05 (m, 2H, H-4′), 1.64 (m, 2H, H-2′), 1.43 (m, 2H, H-3′); ^13^C NMR (100 MHz, CDCl_3_) δ 202.5, 171.5, 162.0, 159.5, 138.5, 135.8, 122.8, 114.9, 108.6, 97.6, 65.0, 55.8, 55.6, 47.1, 39.5, 39.2, 33.4, 28.1, 25.5; HRESIMS *m*/*z* 369.1476 [M + H]^+^ (calcd for C_19_H_26_ClO_5_, 369.1469).

#### 3.2.8. Synthesis of Hex-5-en-1-yl 2-(2-acryloyl-3,5-dimethoxyphenyl)acetate (**5**)

General procedure C. TLC R*_f_* = 0.42 (hexane/EtOAc, 7:3). ^1^H NMR (400 MHz, CDCl_3_) δ 6.68 (dd, *J* = 17.4, 10.5 Hz, 1H, H-10), 6.42 (d, *J* = 2.3 Hz, 1H, H-4), 6.40 (d, *J* = 2.3 Hz, 1H, H-6), 6.09 (dd, *J* = 17.4, 1.4 Hz, 2H, H-11), 5.79 (m, 2H, H-11), 5.76 (m, 1H, H-5′), 4.99 (m, 2H, H-6′), 4.94 (m, 2H, H-6′), 4.05 (t, *J* = 6.9 Hz, 2H, H-1′), 3.82 (s, 3H, H-5 OMe), 3.77 (s, 3H, H-7 OMe), 3.61 (s, 2H, H-2), 2.04 (m, 2H, H-4′), 1.40 (m, 2H, H-3′); ^13^C NMR (100 MHz, CDCl_3_) δ 195.8, 171.2, 161.8, 159.3, 138.5, 138.2, 135.5, 128.5, 122.3, 114.9, 107.9, 97.7, 64.9, 55.8, 38.9, 33.3, 28.1, 25.2; HRESIMS *m*/*z* 333.1632 [M + H]^+^ (calcd for C_19_H_25_O_5_, 333.1702).

#### 3.2.9. Synthesis of (E)-11,13-Dimethoxy-4,5,6,7-tetrahydro-2H-benzo[d][1]oxacyclododecine-2,10(1H)-dione (**6**)

General procedure D. TLC R*_f_* = 0.38 (hexane/EtOAc, 6:4). ^1^H NMR (400 MHz, CDCl_3_) δ 6.55 (d, *J* = 1.8, 1H, H-4), 6.53 (d, *J* = 2.3, 1H, H-6), 6.46 (dd, *J* = 15.8, 7.8, 1H, H-11), 6.21 (dt, *J* = 15.6, 1.2 Hz, 1H, H-10), 4.06 (t, *J* = 4.6, 2H, H-15), 3.83 (s, 3H, H-5 OMe), 3.75 (s, 3H, H-7 OMe), 3.40 (s, 2H, H-2), 2.31 (m, 2H, H-14), 1.71 (m, 2H, H-12; 2H, H-13); ^13^C NMR (100 MHz, CDCl_3_) δ 201.3, 172.2, 162.8, 159.4, 159.0, 133.9, 133.7, 125.6, 108.3, 98.5, 68.1, 56.2, 56.0, 40.1, 34.8, 28.3, 27.2; HRESIMS *m*/*z* 305.1378 [M + H]^+^ (calcd for C_17_H_21_O_5_, 305.1389).

#### 3.2.10. Synthesis of 11,13-Dimethoxy-4,5,6,7-tetrahydro-2H-benzo[d][1]oxacyclododecine-2,10(1H)-dione (**7**)

General procedure D. TLC R*_f_* = 0.48 (hexane/EtOAc, 6:4). ^1^H NMR (400 MHz, CDCl_3_) δ 6.47 (m, 1H, H-10), 6.41 (m, 1H, H-4), 6.37 (m, 1H, H-6), 5.90 (dt, *J* = 11.9, 8.2 Hz, 1H, H-11), 4.01 (m, 2H, H-15), 3.83 (s, 3H, H-5 OMe), 3.74 (s, 3H, H-7 OMe), 3.41 (s, 2H, H-2), 2.29 (m, 2H, H-12), 1.60 (m, 2H, H-13), 1.60 (m, 2H, H-14); ^13^C NMR (100 MHz, CDCl_3_) δ 197.6, 170.7, 161.7, 159.3, 144.1, 134.4, 133.1, 124.5, 108.0, 97.9, 67.6, 56.0, 55.6, 39.3, 28.2, 27.6, 24.8; HRESIMS *m*/*z* 305.1378 [M + H]^+^ (calcd for C_17_H_21_O_5_, 305.1389).

#### 3.2.11. Synthesis of (E)-11-Hydroxy-13-methoxy-4,5,6,7-tetrahydro-2H-benzo[d][1]oxacyclododecine-2,10(1H)-dione (**8**)

General procedure E. TLC R*_f_* = 0.53 (hexane/EtOAc, 1:1). ^1^H NMR (600 MHz, CDCl_3_) δ 6.70 (m, 1H, H-10), 6.60 (m, 1H, H-11), 6.42 (d, *J* = 2.6 Hz, 1H, H-6), 6.33 (t, *J* = 2.9 Hz, 1H, H-4), 4.17 (dd, *J* = 6.2, 2.5 Hz, 2H, H-15), 3.83 (s, 3H, OMe), 3.82 (s, 2H, H-2), 2.45 (m, 2H, H-14), 1.83 (m, 2H, H-12; 2H, H-13); ^13^C NMR (150 MHz, CDCl_3_) δ 196.0, 171.3, 167.0, 164.3, 148.1, 137.4, 131.7, 114.0, 113.6, 100.4, 66.3, 55.5, 43.9, 32.8, 27.1, 26.7; HRESIMS *m*/*z* 291.1223 [M + H]^+^ (calcd for C_16_H_19_O_5_, 291.1232).

#### 3.2.12. Synthesis of Pent-4-en-1-yl 2-(3,5-dimethoxyphenyl)acetate (**11**)

General procedure A. TLC R*_f_* = 0.60 (hexane/EtOAc, 7:3). ^1^H NMR (400 MHz, CDCl_3_) δ 6.44 (d, *J* = 2.3 Hz, 1H, H-4; 1H, H-8), 6.37 (t, *J* = 2.3 Hz, 1H, H-6), 5.77 (m, 1H, H-4′), 5.00 (m, 2H, H-5′), 4.97 (m, 2H, H-5′), 4.10 (t, *J* = 6.6 Hz, 2H, H-1′), 3.78 (s, 3H, H-5 OMe; 3H, H-7 OMe), 3.55 (s, 2H, H-2′), 2.09 (m, 2H, H-3′), 1.72 (m, 2H, H-2′); ^13^C NMR (100 MHz, CDCl_3_) δ 171.5, 161.0, 161.0, 137.5, 136.3, 115.4, 107.4, 107.4, 99.3, 64.4, 55.4, 55.4, 41.8, 30.1, 27.9; HRESIMS *m*/*z* 265.1437 [M + H]^+^ (calcd for C_15_H_21_O_4_, 265.1440).

#### 3.2.13. Synthesis of Pent-4-en-1-yl 2-(2-(3-chloropropanoyl)-3,5-dimethoxyphenyl)acetate (**12**)

General procedure B. TLC R*_f_* = 0.5 (hexane/EtOAc, 7:3). ^1^H NMR (400 MHz, CDCl_3_) δ 6.41 (d, *J* = 2.3 Hz, 1H, H-6), 6.37 (d, *J* = 2.3 Hz, 1H, H-4), 5.78 (m, 1H, H-4′), 5.02 (m, 2H, H-5′), 4.97 (m, 2H, H-5′), 4.09 (t, *J* = 6.6 Hz, 2H, H-1′), 3.84 (s, 3H, H-5 OMe), 3.82 (s, 3H, H-7 OMe), 3.81 (m, 2H, H-11), 3.67 (s, 2H, H-2), 3.35 (t, *J* = 7.1 Hz, 2H, H-10), 2.10 (m, 2H, H-3′), 1.72 (m, 2H, H-2′); ^13^C NMR (100 MHz, CDCl_3_) δ 202.5, 171.5, 162.1, 159.5, 137.6, 135.8, 122.8, 115.4, 108.6, 97.6, 64.5, 55.8, 55.6, 47.1, 39.5, 39.2, 30.1, 27.9; HRESIMS *m*/*z* 355.1286 [M + H]^+^ (calcd for C_18_H_24_ClO_5_, 355.1312).

#### 3.2.14. Synthesis of Pent-4-en-1-yl 2-(2-acryloyl-3,5-dimethoxyphenyl)acetate (**13**)

General procedure C. TLC *R_f_* = 0.78 (hexane/EtOAc, 6:4). ^1^H NMR (400 MHz, CDCl_3_) δ 6.69 (dd, *J* = 17.4, 10.5 Hz, 1H, H-10), 6.42 (d, *J* = 2.3 Hz, 1H, H-4), 6.41 (d, *J* = 2.3 Hz, 1H, H-6), 6.09 (dd, *J* = 17.4, 1.4 Hz, 2H, H-11), 5.79 (m, 2H, H-11), 5.76 (m, 1H, H-4′), 4.97 (m, 2H, H-5′), 4.05 (t, *J* = 6.6 Hz, 2H, H-1′), 3.82 (s, 3H, H-5 OMe), 3.77 (s, 3H, H-7 OMe), 3.61 (s, 2H, H-2), 2.07 (m, 2H, H-3′), 1.69 (m, 2H, H-2′); ^13^C NMR (100 MHz, CDCl_3_) δ 195.8, 171.2, 161.8, 159.3, 138.2, 137.6, 135.5, 128.5, 122.3, 115.3, 107.9, 97.7, 64.4, 55.8, 55.5, 38.9, 30.1, 27.8; HRESIMS *m*/*z* 319.1517 [M + H]^+^ (calcd for C_18_H_23_O_5_, 319.1545).

#### 3.2.15. Synthesis of (E)-10,12-Dimethoxy-5,6-dihydrobenzo[d][1]oxacycloundecine-2,9(1H,4H)-dione (**14**)

General procedure D. TLC *R_f_* = 0.44 (hexane/EtOAc, 6:4). ^1^H NMR (400 MHz, CDCl_3_) δ 6.43 (s, 1H, H-4; 1H, H-6), 6.38 (m, 1H, H-11), 6.25 (td, *J* = 16.0, 1.4 Hz, 1H, H-10), 4.29 (m, 2H, H-14), 3.84 (s, 3H, H-5 OMe), 3.76 (s, 3H, H-7 OMe), 3.36 (s, 2H, H-2), 2.45 (m, 2H, H-12), 2.36 (m, 2H, H-12), 2.02 (m, 2H, H-13), 1.78 (m, 2H, H-13); ^13^C NMR (100 MHz, CDCl_3_) δ 197.9, 171.1, 161.3, 157.8, 155.8, 133.2, 132.5, 122.9, 108.4, 97.9, 65.1, 56.1, 55.6, 40.3, 31.1, 26.9; HRESIMS *m*/*z* 291.1132 [M + H]^+^ (calcd for C_16_H_19_O_5_, 291.1232).

#### 3.2.16. Synthesis of 10,12-Dimethoxy-5,6-dihydrobenzo[d][1]oxacycloundecine-2,9(1H,4H)-dione (**15**)

General procedure D. TLC *R_f_* = 0.58 (hexane/EtOAc, 6:4). ^1^H NMR (400 MHz, CDCl_3_) δ 6.49 (s, 1H, H-4; 1H, H-6), 6.38 (m, 1H, H-11), 5.93 (m, 1H, H-10), 4.29 (m, 2H, H-14), 3.84 (s, 3H, H-5 OMe), 3.76 (s, 3H, H-7 OMe), 3.36 (s, 2H, H-2), 2.45 (m, 2H, H-12), 2.36 (m, 2H, H-12), 2.02 (m, 2H, H-13), 1.80 (m, 2H, H-13); ^13^C NMR (100 MHz, CDCl_3_) δ 197.9, 171.1, 161.3, 157.8, 140.4, 133.2, 132.5, 122.9, 108.4, 97.9, 65.1, 56.1, 55.6, 40.3, 31.1, 26.9; HRESIMS *m*/*z* 291.1132 [M + H]^+^ (calcd for C_16_H_19_O_5_, 291.1232).

#### 3.2.17. Synthesis of (E)-10-Hydroxy-12-methoxy-5,6-dihydrobenzo[d][1]oxacycloundecine-2,9(1H,4H)-dione (**16**)

General procedure E. TLC *R_f_* = 0.55 (hexane/EtOAc, 1:1). ^1^H NMR (600 MHz, CDCl_3_) δ 6.45 (d, *J* = 2.5 Hz, 1H, H-4), 6.40 (d, *J* = 2.5 Hz, 1H, H-6), 6.36 (dt, *J* = 16.3, 1.5 Hz, 1H, H-10), 5.91 (dt, *J* = 16.2, 7.0 Hz, 1H, H-11), 4.31 (t, *J* = 6.0 Hz, 2H, H-14), 3.83 (s, 3H, OMe), 3.46 (s, 2H, H-2), 2.40 (m, 2H, H-12), 1.97 (m, 2H, H-13); ^13^C NMR (150 MHz, CDCl_3_) δ 200.6, 171.7, 164.4, 163.6, 147.0, 137.3, 131.7, 116.1, 113.5, 100.3, 64.8, 55.7, 42.2, 30.5, 24.6; HRESIMS *m*/*z* 277.0906 [M + H]^+^ (calcd for C_15_H_17_O_5_, 277.1076).

#### 3.2.18. Synthesis of (S)-Hept-6-en-2-yl 2-(3,5-dimethoxyphenyl)acetate (**18**)

General procedure A. TLC *R_f_* = 0.67 (hexane/EtOAc, 7:3). ^1^H NMR (400 MHz, CD_3_OD) δ 6.43 (d, *J* = 2.3 Hz, 1H, H-4; 1H, H-8), 6.37 (t, *J* = 2.3 Hz, 1H, H-6), 5.74 (m, 1H, H-5′), 4.96 (m, 2H, H-6′), 4.93 (m, 1H, H-1′), 4.90 (m, 2H, H-6′), 3.75 (s, 3H, H-5 OMe; 3H, H-7 OMe), 3.52 (s, 2H, H-2), 1.99 (m, 2H, H-4′), 1.54 (m, 2H, H-2′), 1.34 (m, 2H, H-3′), 1.20 (d, *J* = 6.4 Hz, 3H, H-7′); ^13^C NMR (100 MHz, CD_3_OD) δ 173.1, 162.4, 162.4, 139.6, 137.8, 115.1, 108.3, 108.3, 100.0, 72.7, 55.7, 55.7, 42.8, 36.4, 34.5, 25.7, 20.2; HRESIMS *m*/*z* 293.1761 [M + H]^+^ (calcd for C_17_H_25_O_4_, 293.1753).

#### 3.2.19. Synthesis of (S)-Hept-6-en-2-yl 2-(2-(3-chloropropanoyl)-3,5-dimethoxyphenyl)acetate (**19**)

General procedure B. TLC *R_f_* = 0.5 (hexane/EtOAc, 7:3). ^1^H NMR (600 MHz, CDCl_3_) δ 6.40 (d, *J* = 2.3 Hz, 1H, H-6), 6.37 (d, *J* = 2.3 Hz, 1H, H-4), 5.78 (m, 1H, H-5′), 5.00 (m, 2H, H-6′), 4.95 (m, 2H, H-6′), 4.89 (m, 2H, H-1′), 3.83 (s, 3H, H-5 OMe), 3.82 (m, 2H, H-11), 3.81 (s, 3H, H-7 OMe), 3.67 (s, 2H, H-2), 3.35 (t, *J* = 7.1 Hz, 2H, H-10), 2.03 (m, 2H, H-4′), 1.58 (m, 2H, H-2′), 1.49 (m, 2H, H-2′), 1.38 (m, 2H, H-3′), 1.21 (d, *J* = 6.4 Hz, 3H, H-7′); ^13^C NMR (150 MHz, CDCl_3_) δ 202.3, 170.9, 161.8, 159.2, 138.4, 135.6, 122.6, 114.6, 108.2, 97.3, 71.4, 55.6, 55.3, 46.9, 39.3, 39.2, 35.2, 33.4, 24.5, 19.8; HRESIMS *m*/*z* 383.1616 [M + H]^+^ (calcd for C_20_H_28_ClO_5_, 383.1625).

#### 3.2.20. Synthesis of (S)-Hept-6-en-2-yl 2-(2-acryloyl-3,5-dimethoxyphenyl)acetate (**20**)

General procedure C. TLC *R_f_* = 0.60 (hexane/EtOAc, 6:4). ^1^H NMR (600 MHz, CDCl_3_) δ 6.68 (dd, *J* = 17.4, 10.5 Hz, 1H, H-10), 6.43 (d, *J* = 2.3 Hz, 1H, H-4), 6.41 (d, *J* = 2.3 Hz, 1H, H-6), 6.09 (dd, *J* = 17.4, 1.4 Hz, 2H, H-11), 5.79 (m, 2H, H-11), 5.76 (m, 1H, H-5′), 4.96 (m, 2H, H-6′), 4.94 (m, 2H, H-6′), 4.88 (m, 2H, H-1′), 3.83 (s, 3H, H-5 OMe), 3.78 (s, 3H, H-7 OMe), 3.60 (s, 2H, H-2), 2.03 (m, 2H, H-4′), 1.57 (m, 2H, H-2′), 1.36 (m, 2H, H-3′), 1.18 (d, *J* = 6.4 Hz, 3H, H-7′); ^13^C NMR (150 MHz, CDCl_3_) δ 195.8, 170.7, 161.6, 159.1, 138.5, 138.1, 135.4, 128.5, 122.2, 114.6, 107.6, 97.5, 71.4, 55.6, 55.4, 38.9, 35.2, 33.4, 24.5, 19.9; HRESIMS *m*/*z* 347.1882 [M + H]^+^ (calcd for C_20_H_27_O_5_, 347.1858).

#### 3.2.21. Synthesis of (S,E)-11,13-Dimethoxy-4-methyl-4,5,6,7-tetrahydro-2H-benzo[d][1]oxacyclododecine-2,10(1H)-dione (**21**)

General procedure D. TLC *R_f_* = 0.38 (hexane/EtOAc, 6:4). ^1^H NMR (400 MHz, CD_3_OD) δ 6.54 (d, *J* = 2.3 Hz, 1H, H-4), 6.53 (d, *J* = 2.3 Hz, 1H, H-6), 6.48 (dd, *J* = 15.6, 7.8 Hz, 1H, H-11), 6.21 (dt, *J* = 15.6, 1.2 Hz, 1H, H-10), 4.89 (m, 1H, H-15), 3.83 (s, 3H, H-5 OMe), 3.75 (s, 3H, H-7 OMe), 3.37 (s, 2H, H-2), 2.33 (m, 2H, H-12), 2.20 (m, 2H, H-12), 1.90 (m, 2H, H-13), 1.80 (m, 2H, H-14), 1.49 (m, 2H, H-14), 1.42 (m, 2H, H-13), 1.15 (d, *J* = 6.4 Hz, 3H, H-16); ^13^C NMR (100 MHz, CD_3_OD) δ 201.2, 172.0, 162.8, 159.4, 159.0, 134.1, 133.8, 123.4, 108.3, 98.5, 74.5, 56.3, 56.0, 40.2, 35.1, 35.0, 25.5, 20.5; HRESIMS *m*/*z* 319.1466 [M + H]^+^ (calcd for C_18_H_23_O_5_, 319.1545).

#### 3.2.22. (S)-11,13-Dimethoxy-4-methyl-4,5,6,7-tetrahydro-2H-benzo[d][1]oxacyclododecine-2,10(1H)-dione (**22**)

General procedure D. TLC *R_f_* = 0.45 (hexane/EtOAc, 6:4). ^1^H NMR (400 MHz, CD_3_OD) δ 6.53 (d, *J* = 1.8 Hz, 1H, H-4), 6.51 (d, *J* = 2.3 Hz, 1H, H-6), 6.37 (dt, *J* = 12.4, 1.6 Hz, 1H, H-10), 5.99 (dt, *J* = 12.4, 8.2 Hz, 1H, H-11), 4.80 (m, 1H, H-15), 3.83 (s, 3H, H-5 OMe), 3.78 (s, 3H, H-7 OMe), 3.37 (s, 2H, H-2), 1.40–2.25 (m, 2H, H-12; 2H, H-13; 2H, H-14), 1.13 (d, *J* = 6.4 Hz, 3H, H-16); ^13^C NMR (100 MHz, CD_3_OD) δ 199.9, 171.8, 163.6, 160.6, 146.3, 135.2, 133.8, 125.4, 109.4, 98.5, 75.2, 56.5, 56.0, 40.1, 31.9, 28.4, 26.0, 19.8; HRESIMS *m*/*z* 319.1466 [M + H]^+^ (calcd for C_18_H_23_O_5_, 319.1545).

#### 3.2.23. Synthesis of (S,E)-11-Hydroxy-13-methoxy-4-methyl-4,5,6,7-tetrahydro-2H-benzo[d][1]oxacyclododecine-2,10(1H)-dione (**23**)

General procedure E. TLC *R_f_* = 0.64 (hexane/EtOAc, 1:1). ^1^H NMR (400 MHz, CDCl_3_) δ 6.67 (dd, *J* = 16.5, 1.0 Hz, 1H, H-10), 6.62 (ddd, *J* = 15.1, 8.2, 3.6 Hz, 1H, H-11), 6.41 (d, *J* = 2.8 Hz, 1H, H-6), 6.32 (d, *J* = 2.8 Hz, 1H, H-4), 4.85 (m, 1H, H-15), 4.04 (d, *J* = 17.9 Hz, 2H, H-2), 3.82 (s, 3H, OMe), 3.54 (d, *J* = 17.9 Hz, 2H, H-2), 2.47 (m, 2H, H-12), 2.32 (m, 2H, H-12), 1.97 (m, 2H, H-14), 1.89 (m, 2H, H-13), 1.65 (m, 2H, H-14), 1.64 (m, 2H, H-13), 1.24 (d, *J* = 6.4 Hz, 3H, H-16); ^13^C NMR (100 MHz, CDCl_3_) δ 196.4, 171.3, 166.9, 164.4, 148.3, 137.6, 131.5, 114.3, 113.6, 100.6, 72.7, 55.6, 44.2, 34.1, 32.8, 24.2, 20.1; HRESIMS *m*/*z* 305.1411 [M + H]^+^ (calcd for C_17_H_21_O_5_, 305.1389).

### 3.3. Measurement of NO Production

Nitrite concentrations were measured in culture supernatants using the Griess reagent (1% sulfanilamide, 0.1% *N*-(1-naphthyl)-ethylenediamine dihydrochloride, 5% phosphoric acid). Cells were seeded in 96-well plates at a density of 5 × 10^5^ cells/mL and treated with specific concentrations of compounds **6**, **7**, **8**, **14**, **15**, **16**, **21**, **22**, and **23** at 37 °C for 1 h. Subsequently, cells were stimulated with 0.5 μg/mL LPS (Sigma-Aldrich, Carlsbad, CA, USA) and incubated for 24 h at a final volume of 200 μL. Thereafter, 100 μL of the cell culture supernatant was mixed with an equal volume of the Griess reagent. The mixture was incubated at room temperature for 5 min. A standard curve was generated using sodium nitrite, and the absorbance was measured at 540 nm to calculate the nitrite concentration. All experiments were performed in triplicate.

### 3.4. Cytotoxicity Measurement

Cytotoxicity was determined using the 3-(4,5-dimethyl-2-thiazolyl)-2,5-diphenyl-2H-tetrazolium bromide (MTT, Amresco, Solon, OH, USA) assay. RAW 264.7 cells were seeded in 96-well plates at a concentration of 1 × 10^5^ cells/mL and incubated at 37 °C for 24 h with various concentrations of compounds **6**, **7**, **8**, **14**, **15**, **16**, **21**, **22**, and **23**. Thereafter, 5 μL of a 5 mg/mL MTT solution was added to the cells and incubated at 37 °C for 4 h. To dissolve the crystals, 100 μL of fresh DMSO was added and incubated for 10 min. The absorbance was measured at 570 nm using a microplate reader (Thermo Scientific Inc., San Diego, CA, USA). Relative cell viability was calculated based on the absorbance of the untreated control. All experiments were performed in triplicate.

### 3.5. Measurement of Antibiotic and Antibacterial Activity

Antibacterial and antifungal activity assays were performed using 96-well plates with *Staphylococcus aureus* KCTC 3881 (bacterium), *Escherichia coli* DH5α (bacterium), and *Candida albicans* KCTC 27242 (yeast) (Korean Collection for Type Cultures, Daejeon, Republic of Korea). Cell cultures were diluted with a sterile medium to a 0.5 McFarland Standard, and the *C. albicans* culture was further diluted 100-fold before use. After dispensing 95 μL of culture medium into each well, compounds dissolved in DMSO were added to achieve final concentrations of 12.5, 25, 50, 100, and 200 μg/mL, and the final volume in each well was adjusted to 100 μL. The plates were incubated at 37 °C for 24 h, and cell inhibition was measured at 600 nm for *S. aureus* and *E. coli* and at 530 nm for *C. albicans* using a Multiskan GO Microplate Spectrophotometer (Thermo Scientific, Waltham, MA, USA). IC_50_ values were calculated using an exponential trend line in Excel software (Microsoft, Redmond, WA, USA), and values were expressed as the mean ± SD of triplicate measurements. Kanamycin was used as a positive control for bacteria (*S. aureus* and *E. coli*) and nystatin was used as the positive control for fungi (*C. albicans*).

## 4. Conclusions

In this study, we synthesized derivatives based on the structure of curvularin isolated from *Penicillium* sp. derived from Antarctic marine sponges, and systematically evaluated their anti-inflammatory, anticancer, and antibacterial activities. Among the synthesized derivatives, compounds **6**, **7**, **8**, **14**, **15**, **16**, and **23** were newly synthesized. Some exhibited high cancer cytotoxicity and inhibitory effects on inflammatory mediators, confirming their potential as anti-inflammatory and anticancer candidates. Furthermore, antibacterial activity tests demonstrated that the novel derivatives exhibited excellent growth inhibition against *S. aureus* and *C. albicans*, suggesting their potential as novel antibacterial agents. This study confirms the possibility of enhancing physiological activity through structural changes in curvularin derivatives, thereby suggesting an important direction for future research on natural product-based drug development.

## Figures and Tables

**Table 1:** Anti-inflammatory activity and selectivity index (SI) of derivatives.

Compounds	NO Inhibition	Cytotoxicity	SI (Selectivity Index)
	IC_50_ Value (μg/mL)		(Cytotoxicity/ NO Inhibition)
**6**	1.83	4.15	2.27
**7**	2.32	5.1	2.20
**8**	>5.0	3.63	<1.0
**14**	1.31	1.35	1.03
**15**	2.01	4.28	2.13
**16**	0.95	0.85	0.89
**21**	2.21	2.89	1.31
**22**	2.84	4.91	1.73
**23**	0.18	0.13	0.72

**Table 2:** Anticancer activity of curvularin derivatives.

Compounds	IC_50_ Value (μg/mL)			
	HaCaT	HCT116	HeLa	A375
**6**	2.02	>5.0	>5.0	>5.0
**7**	3.16	>5.0	>5.0	>5.0
**8**	3.97	>5.0	>5.0	>5.0
**14**	1.59	4.25	>5.0	>5.0
**15**	2.34	>5.0	>5.0	>5.0
**16**	0.89	2.68	>5.0	3.25
**21**	2.03	>5.0	>5.0	>5.0
**22**	2.47	>5.0	>5.0	>5.0
**23**	0.09	0.38	0.79	0.35

**Table 3:** Antibacterial activity of curvularin derivatives.

Compounds	*Staphylococcus aureus*		*Escherichia coli*	
	IC_50_ (μg/mL)	MIC (μg/mL)	IC_50_ (μg/mL)	MIC (μg/mL)
Kanamycin ^a^	14.97 ± 6.17	29.18 ± 4.71	7.31 ± 2.61	26.32 ± 2.50
**6**	>200	-	>200	-
**7**	137.43 ± 27.62	-	>200	-
**8**	49.94 ± 3.29	120.24 ± 6.60	>200	-
**14**	90.56 ± 14.35	168.50 ± 42.88	>200	-
**15**	>200	-	>200	-
**16**	39.04 ± 8.97	93.46 ± 15.59	>200	-
**21**	192.99 ± 14.36	-	>200	-
**22**	192.47 ± 5.30	-	>200	-
**23**	8.90 ± 2.28	25.70 ± 1.34	>200	-

^a^ Positive control: Kanamycin (*S. aureus*, *E. coli*).

**Table 4:** Antifungal activity of curvularin derivatives.

Compounds	*Candida albicans*	
	IC_50_ (μg/mL)	MIC (μg/mL)
Nystatin ^a^	0.83 ± 0.22	1.11 ± 0.07
**6**	>200	-
**7**	116.10 ± 9.22	-
**8**	6.86 ± 0.59	57.36 ± 0.85
**14**	>200	-
**15**	157.02 ± 7.53	-
**16**	32.25 ± 5.32	139.58 ± 12.21
**21**	>200	-
**22**	133.30 ± 9.29	-
**23**	5.24 ± 0.02	10.48 ± 0.04

^a^ Positive control: Nystatin (*C. albicans*).

**Table 5:** Comparison of the physiological activities of curvularin and its derivatives.

Compound	Activity				Reference
	Anti-Inflamatory (IC_50_)	Anti-Cancer (IC_50_)	Anti-Bacteria (MIC)	Anfi-Fungal (MIC)	
Curvularin	18.1 μM				[16]
Dehydrocurvularin	0.44 μM				[26]
		10.56–29.17 μM (against HT1080, T46D, and A2780S)	40 μg/mL (against *S. aureus*)	40 (μg/mL) (against *C. albicans*)	[36]
		10.3 μg/mL (against HeLa)	375 μg/mL (against *S. aureus*)	-	[25]
		1.25–30.06 μM (against human tumour cell lines)			[37]
			1 μg/mL (against *E. coli*)		[40]
		7.9 μM (against HeLa)	2.5 μg/mL (against *E. coli*)		[41]
11-α-methoxycurvularin			50 μg/mL (against *S. aureus*) 50 μg/mL (against *E. coli*)		[42]
Compound **23** (*S*,*E*)-11-hydroxy-13-methoxy-4-methyl-4,5,6,7-tetrahydro-2*H*-benzo[*d*][1]oxacyclododecine-2,10(1*H*)-dione	0.59 μM	0.38 μM (HCT116), 0.79 μM (HeLa), 0.35 μM (A375)	25.7 μg/mL (against *S. aureus*)	10.48 μg/mL (against *C. albicans*)	This study

## Data Availability

The original contributions presented in this study are included in the article. Further inquiries can be directed to the corresponding author.

## Supplementary Materials

The following supporting information can be downloaded at: https://www.mdpi.com/article/10.3390/molecules31061061/s1, Synthetic procedures and characterization data for the compounds **3**–**8**, **11**–**16**, **18**–**23**.

## Abbreviations

The following abbreviations are used in this manuscript:

NO	Nitric oxide
RCM	Ring-closing metathesis
SAR	Structure–activity relationship
SI	Selectivity index
DMSO	Dimethyl sulfoxide
NMR	Nuclear magnetic resonance
TLC	Thin-layer chromatography
MTT	3-(4,5-dimethyl-2-thiazolyl)-2,5-diphenyl-2H-tetrazolium bromide
