# Supplementary material for: Synthesis and Biological Evaluation of Curvularin-Type Derivatives with Potential Anti-Inflammatory, Anticancer, and Antimicrobial Activities
Source: Molecules. 2026 Mar 23;31(6):1061. doi: 10.3390/molecules31061061 (PMC13029060; doi:10.3390/molecules31061061)
Supplement: Supplementary file 1 [file molecules-31-01061-s001.zip › molecules-4178468-supplementary.pdf]

Supporting Information

Synthesis and Biological Evaluation of Curvularin-Type Derivatives with Potential Anti-Inflammatory, Anticancer, and Antimicrobial Activities

Kyung Hee Kim [1,†], Tai Kyoung Kim [2,†], Ju-Mi Hong [1,3], Jin A Kim [1,3], Min Ju Kim [1], Jin-Hyoung Kim [1,4], Joung Han Yim [1,2,*], Il-Chan Kim [1,*] and Se Jong Han [1,4,*]

[1] Division of Life Sciences, Korea Polar Research Institute, Incheon 21990, Republic of Korea; happy_vu@naver.com (K.H.K.); wnal5555@kopri.re.kr (J.-M.H.); kimja611@kopri.re.kr (J.A.K.); mjkim1113@kopri.re.kr (M.J.K.); kimjh@kopri.re.kr (J.H.K.);

[2] CRYOTECH Inc., 2F-211-3, 71 Mieumsandan 5-ro 41beon-gil, Gangseo-gu, Busan 46744, Republic of Korea; tkkim@cryotech.co.kr (T.K.K.)

[3] Department of Marine Sciences, Incheon National University, Incheon 22012, Republic of Korea

[4] Department of Polar Science, University of Science and Technology, Daejeon 34113, Republic of Korea

* Correspondence: jhyim@kopri.re.kr (J.H.Y.); ickim@kopri.re.kr (I.-C.K.); hansj@kopri.re.r (S.J.H.); Tel.: +82-32-760-5540 (J.H.Y.); +82-760-5541 (I.-C.K.); +82-760-5521 (S. J. H.); Fax: +82-32-760-5509

† These authors contributed equally to this work.

Contents

Synthetic procedures and characterization data for the compound **3** S4

Figure S1. NMR spectrums of Hex-5-en-1-yl 2-(3,5-dimethoxyphenyl)acetate (**3**) S7

Synthetic procedures and characterization data for the compound **4** S8

Figure S2. NMR spectrums of Hex-5-en-1-yl 2-(2-(3-chloropropanoyl)-3,5-dimethoxyphenyl) acetate (**4**) S14

Synthetic procedures and characterization data for the compound **5** S15

Figure S3. NMR spectrums of Hex-5-en-1-yl 2-(2-acryloyl-3,5-dimethoxyphenyl) acetate (**5**) S21

Synthetic procedures and characterization data for the compound **6** S22

Figure S4. NMR spectrums of (*E*)-11,13-dimethoxy-4,5,6,7-tetrahydro-2*H*-benzo[*d*][1] oxacyclododecine-2,10(1*H*)-dione (**6**) S25

Synthetic procedures and characterization data for the compound **7** S26

Figure S5. NMR spectrums of 11,13-dimethoxy-4,5,6,7-tetrahydro-2*H*-benzo[*d*][1] oxacyclododecine-2,10(1*H*)-dione (**7**) S32

Synthetic procedures and characterization data for the compound **8** S33

Figure S6. NMR spectrums of (*E*)-11-hydroxy-13-methoxy-4,5,6,7-tetrahydro-2*H*-benzo[*d*][1] oxacyclododecine-2,10(1*H*)-dione (**8**) S38

Synthetic procedures and characterization data for the compound **11** S39

Figure S7. NMR spectrums of pent-4-en-1-yl 2-(3,5-dimethoxyphenyl)acetate (**11**) S45

Synthetic procedures and characterization data for the compound **12** S46

Figure S8. NMR spectrums of pent-4-en-1-yl 2-(2-(3-chloropropanoyl)-3,5-dimethoxyphenyl) acetate (**12**) S52

Synthetic procedures and characterization data for the compound **13** S53

Figure S9. NMR spectrums of pent-4-en-1-yl 2-(2-acryloyl-3,5-dimethoxyphenyl)acetate (**13**) S59

Synthetic procedures and characterization data for the compound **14** S60

Figure S10. NMR spectrums of (*E*)-10,12-dimethoxy-5,6-dihydrobenzo[*d*][1] oxacycloundecine-2,9(1*H*,4*H*)-dione (**14**) S66

Synthetic procedures and characterization data for the compound **15** S67

Figure S11. NMR spectrums of 10,12-dimethoxy-5,6-dihydrobenzo[*d*][1]oxacycloundecine-2,9(1*H*,4*H*)-dione (**15**) S69

Synthetic procedures and characterization data for the compound **16** S70

Figure S12. NMR spectrums of (*E*)-10-hydroxy-12-methoxy-5,6-dihydrobenzo[*d*][1] oxacycloundecine-2,9(1*H*,4*H*)-dione (**16**) S76

Synthetic procedures and characterization data for the compound **18** S77

Figure S13. NMR spectrums of (*S*)-hept-6-en-2-yl 2-(3,5-dimethoxyphenyl)acetate (**18**) S80

Synthetic procedures and characterization data for the compound **19** S81

Figure S14. NMR spectrums of (*S*)-hept-6-en-2-yl 2-(2-(3-chloropropanoyl)-3,5-dimethoxyphenyl)acetate (**19**) S86

Synthetic procedures and characterization data for the compound **20** S87

Figure S15. NMR spectrums of (*S*)-hept-6-en-2-yl 2-(2-acryloyl-3,5-dimethoxyphenyl)acetate (**20**) S92

Synthetic procedures and characterization data for the compound **21** S93

Figure S16. NMR spectrums of (*S*,*E*)-11,13-dimethoxy-4-methyl-4,5,6,7-tetrahydro-2*H*-benzo[*d*][1]oxacyclododecine-2,10(1*H*)-dione (**21**) S96

Synthetic procedures and characterization data for the compound **22** S97

Figure S17. NMR spectrums of (*S*)-11,13-dimethoxy-4-methyl-4,5,6,7-tetrahydro-2*H*-benzo[*d*][1]oxacyclododecine-2,10(1*H*)-dione (**22**) S100

Synthetic procedures and characterization data for the compound **23** S101

Figure S18. NMR spectrums of (*S*,*E*)-11-hydroxy-13-methoxy-4-methyl-4,5,6,7-tetrahydro-2*H*-benzo[*d*][1]oxacyclododecine-2,10(1*H*)-dione (**23**) S107

Synthetic procedures and characterization data for the compound **3**

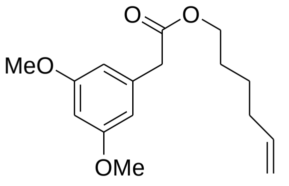

3

Hex-5-en-1-yl 2-(3,5-dimethoxyphenyl)acetate (**3**)

A flame-dried 100 mL round-bottom flask equipped with a magnetic stir bar was charged with anhydrous DCM (10 mL), 3,5-Dimethoxyphenylacetic Acid (**1**) (1.0 g, 5.1 mmol), 5-Hexen-1-ol (2) (721 μL, 6.1 mmol), and Mukaiyama reagent (2-Chloro-1-methylpyridinium iodide) (1.56 g, 6.1 mmol). The mixture was stirred at room temperature for 15 min. Then, TEA (2.13 mL, 15.3 mmol) was slowly added to the mixture, and the mixture was stirred at room temperature for 20 h. The reaction mixture was filtered and purified by flash column chromatography (hexane/EtOAc, 9:1) to give **3** (1.08 g, 76%).

TLC R_f= 0.57 (hexane/EtOAc, 7:3). ^{1}H NMR (400 MHz, CD_3OD) δ 6.43 (d, *J* = 2.3 Hz, 1H, H-4; 1H, H-8), 6.37 (t, *J* = 2.3 Hz, 1H, H-6), 5.77 (m, 1H, H-5′), 4.98 (m, 2H, H-6′), 4.93 (m, 2H, H-6′), 4.09 (t, *J* = 6.4 Hz, 2H, H-1′), 3.75 (s, 3H, H-5 OMe; 3H, H-7 OMe), 3.52 (s, 2H, H-2), 2.04 (m, 2H, H-4′), 1.62 (m, 2H, H-2′), 1.40 (m, 2H, H-3′); ^{13}C NMR (100 MHz, CD_3OD) δ 173.4, 162.4, 162.4, 139.5, 137.7, 115.2, 108.3, 108.3, 100.0, 65.8, 55.7, 55.7, 42.3, 34.3, ,29.1, 26.3; HRESIMS *m/z* 279.1596 [M + H]$^+$ (calcd for $C_{16}H_{23}O_4$, 279.1596).

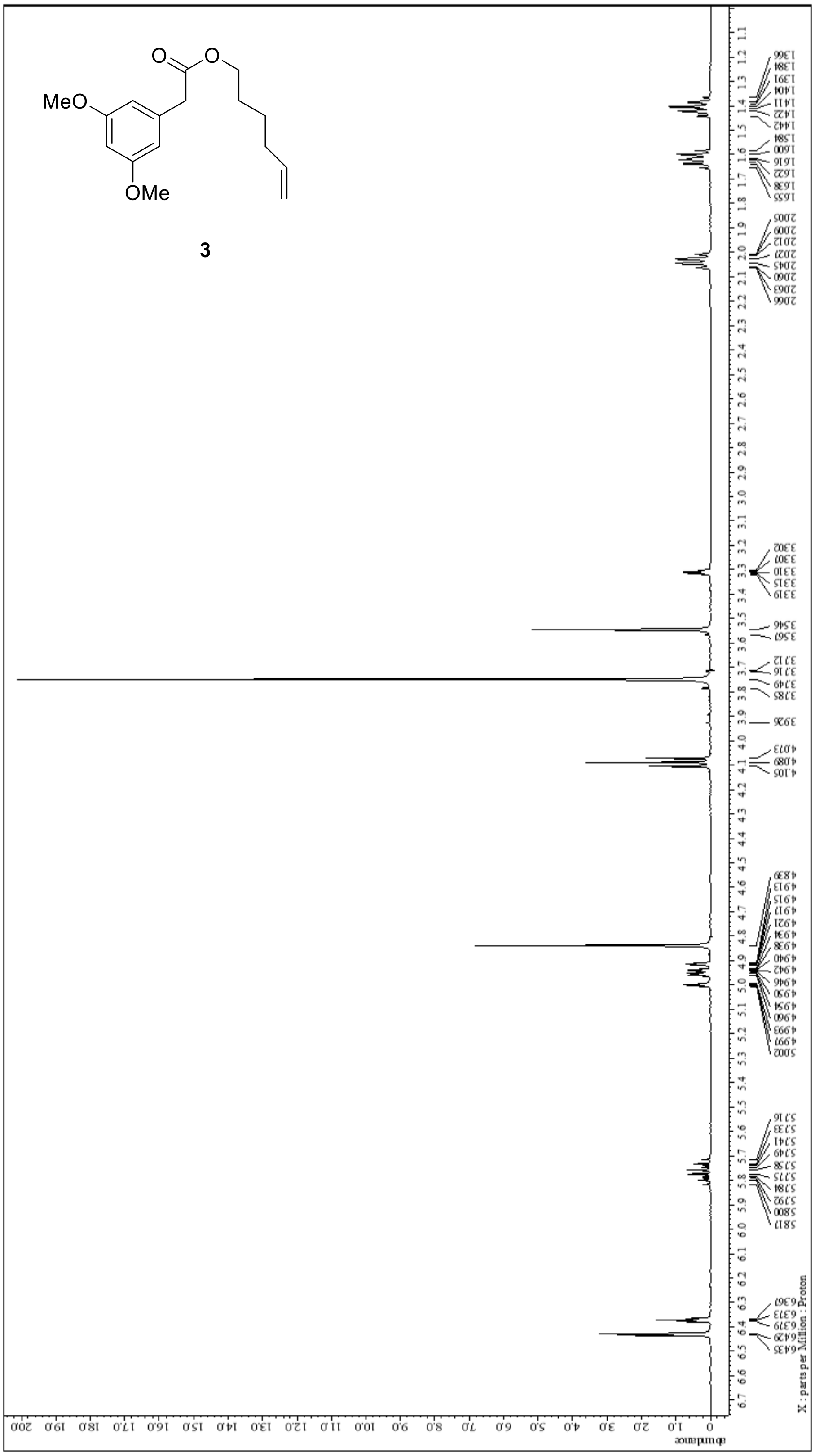

MeO
OMe
3
X : parts per Million : Proton
abundance

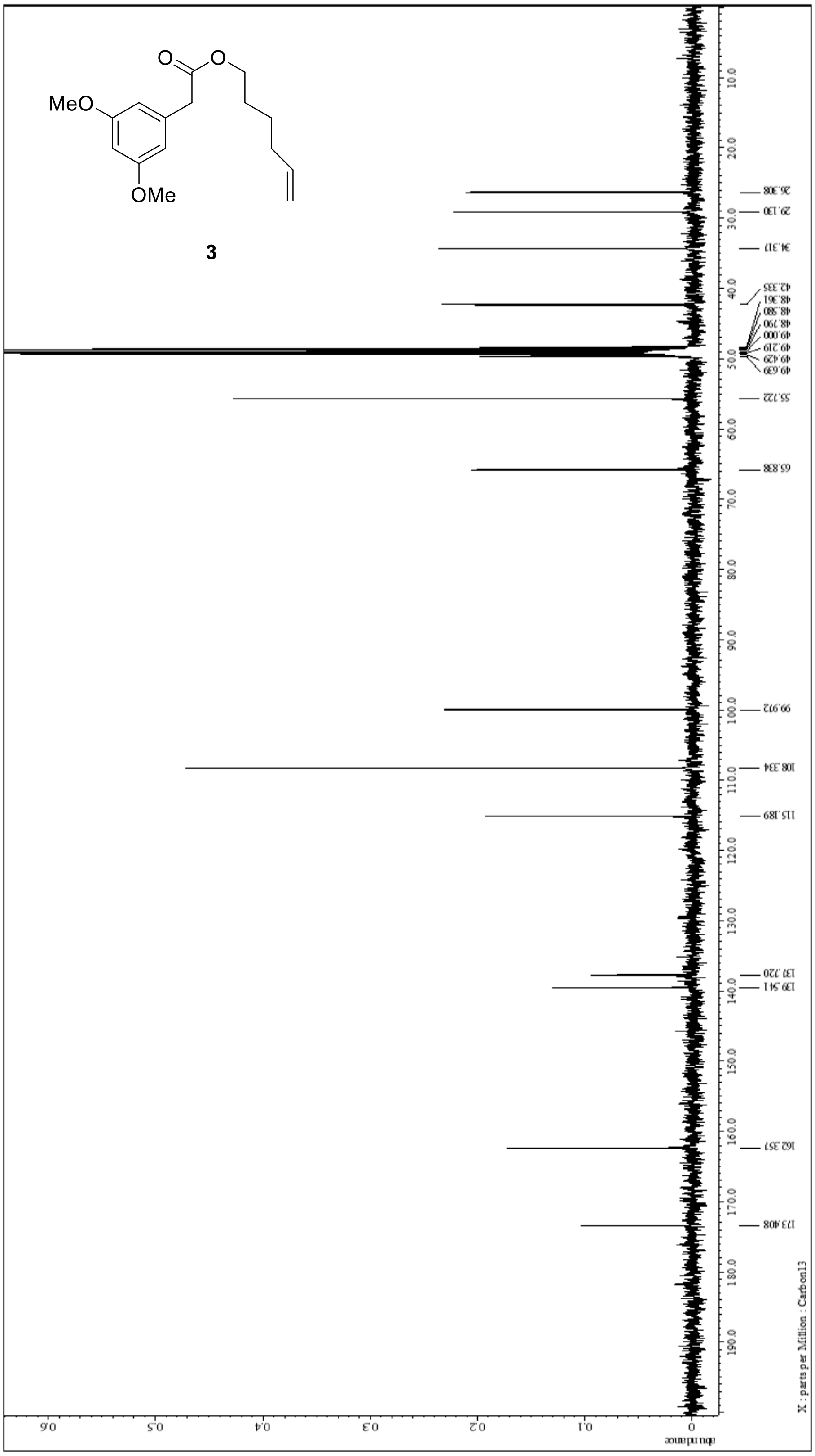
MeO
OMe
O
O
3
26.308
29.130
34.317
42.335
48.361
48.580
48.790
49.000
49.219
49.429
49.639
55.122
65.838
99.912
108.334
115.189
137.720
139.541
162.357
173.408
X : parts per Million : Carbon13
abundance

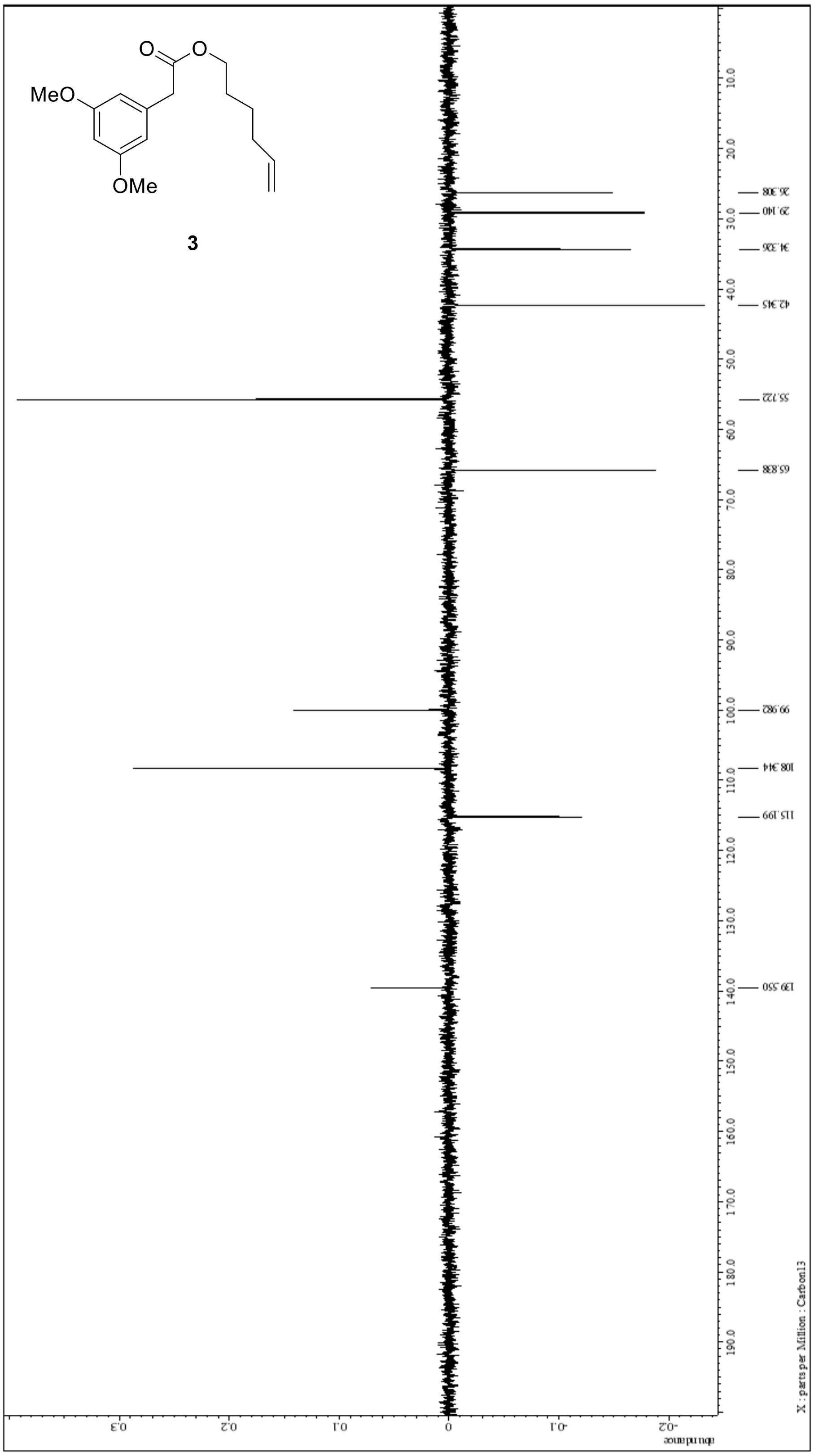

Figure S1. NMR spectrums of Hex-5-en-1-yl 2-(3,5-dimethoxyphenyl)acetate (**3**)

Synthetic procedures and characterization data for the compound **4**

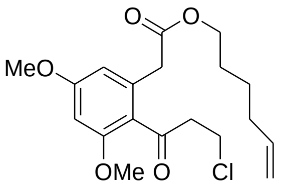

4

Hex-5-en-1-yl 2-(2-(3-chloropropanoyl)-3,5-dimethoxyphenyl)acetate (4)

A flame-dried 50 mL round-bottom flask equipped with a magnetic stir bar was charged with anhydrous DCM (3 mL), and 3-chloropropanoyl chloride (**9**) (87 μL, 0.9 mmol) was added. After cooling to –78 °C, $SnCl_4$ (1 M in DCM, 906 μL, 0.9 mmol) was added and stirred for 15 min. Compound **3** (194 mg, 0.7 mmol) was dissolved in DCM (3 mL) and added to the reaction mixture. The reaction temperature was then raised to –20 °C and stirred for 2 h. After completion of the reaction, the reactant was poured into a separatory funnel filled with ice (3 g) and H_2O (3 mL), and extraction was performed by adding DCM. The obtained organic layer was washed with sat. aq. $NaHCO_3$ (4 mL) and H_2O (4 mL), dried with $MgSO_4$, filtered, and concentrated using a rotary evaporator. The reaction mixture was purified using flash column chromatography (hexane/EtOAc, 9:1) to obtain 4 (144 mg, 56%).

TLC R_f= 0.42 (hexane/EtOAc, 7:3). ^{1}H NMR (400 MHz, $CDCl_3$) δ 6.41 (d, *J* = 2.3 Hz, 1H, H-6), 6.37 (d, *J* = 2.3 Hz, 1H, H-4), 5.78 (m, 1H, H-5′), 5.00 (m, 2H, H-6′), 4.95 (m, 2H, H-6′), 4.08 (t, *J* = 6.9 Hz, 2H, H-1′), 3.84 (s, 3H, H-5 OMe), 3.82 (s, 3H, H-7 OMe), 3.82 (m, 2H, H-11), 3.67 (s, 2H, H-2), 3.35 (t, *J* = 7.1 Hz, 2H, H-10), 2.05 (m, 2H, H-4′), 1.64 (m, 2H, H-2′), 1.43 (m, 2H, H-3′); ^{13}C NMR (100 MHz, $CDCl_3$) δ 202.5, 171.5, 162.0, 159.5, 138.5, 135.8, 122.8, 114.9, 108.6, 97.6, 65.0, 55.8, 55.6, 47.1, 39.5, 39.2, 33.4, 28.1, 25.5; HRESIMS *m/z* 369.1476 $[M + H]^+$ (calcd for $C_{19}H_{26}ClO_5$, 369.1469).

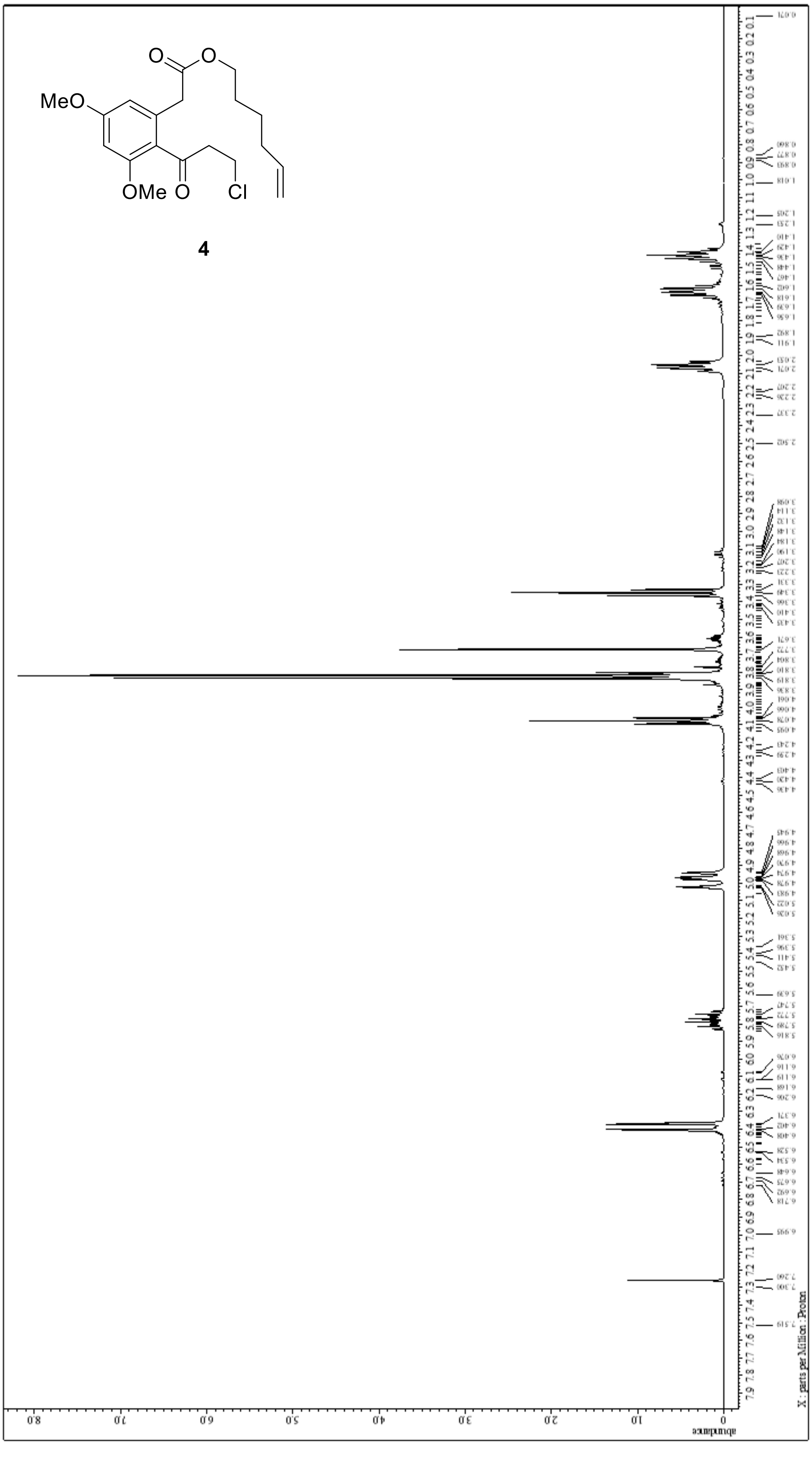
4

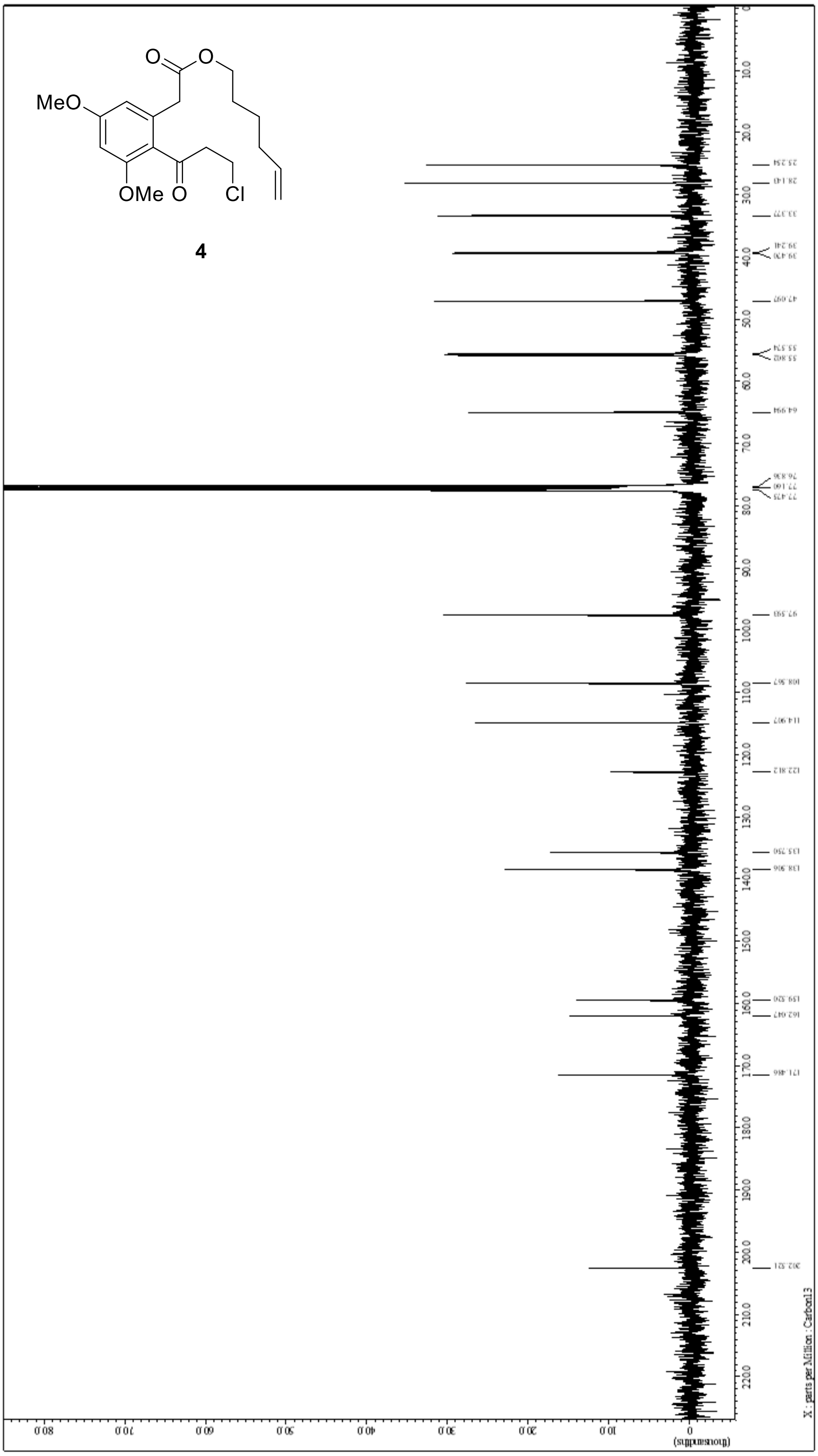

4

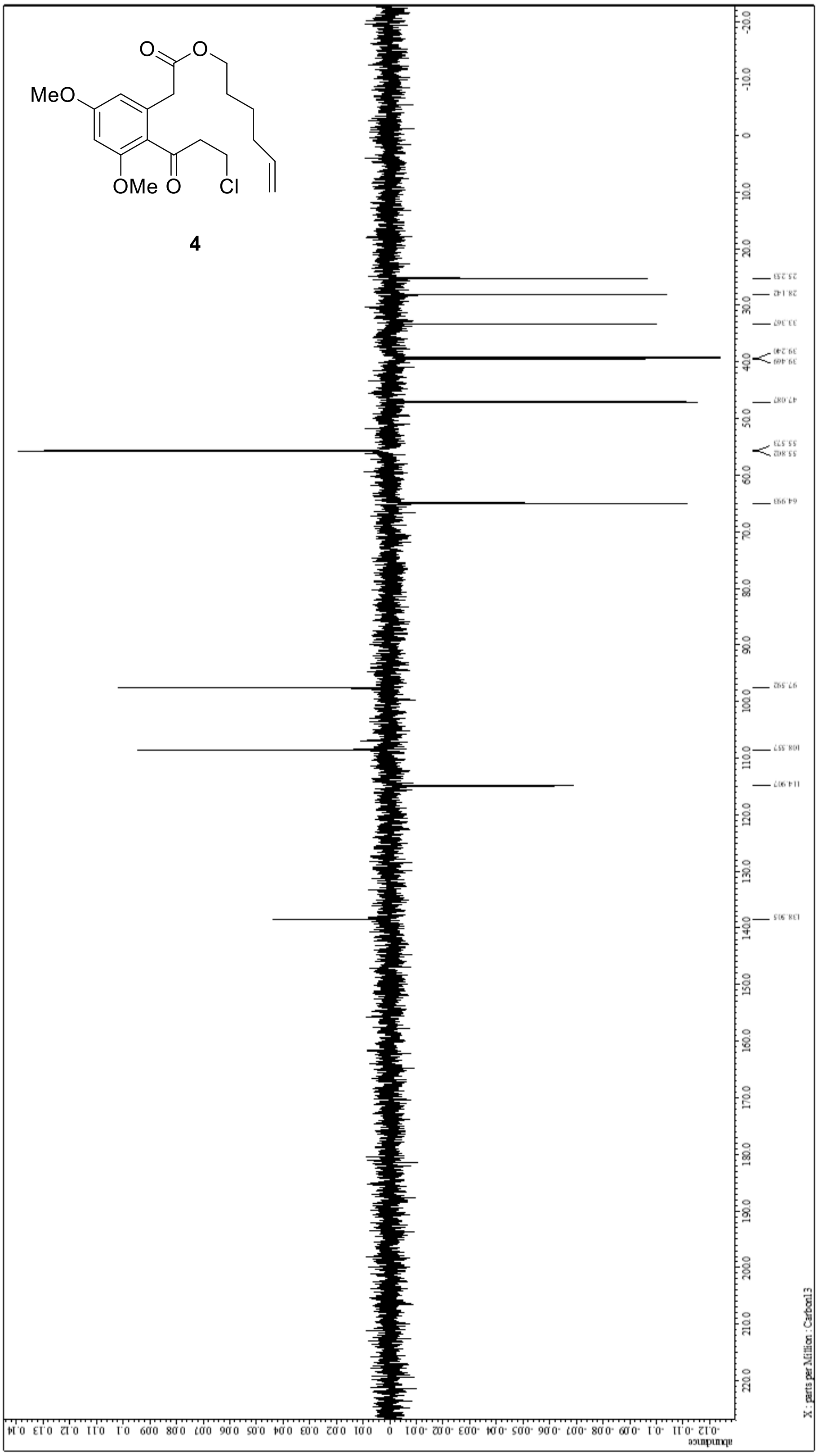

4

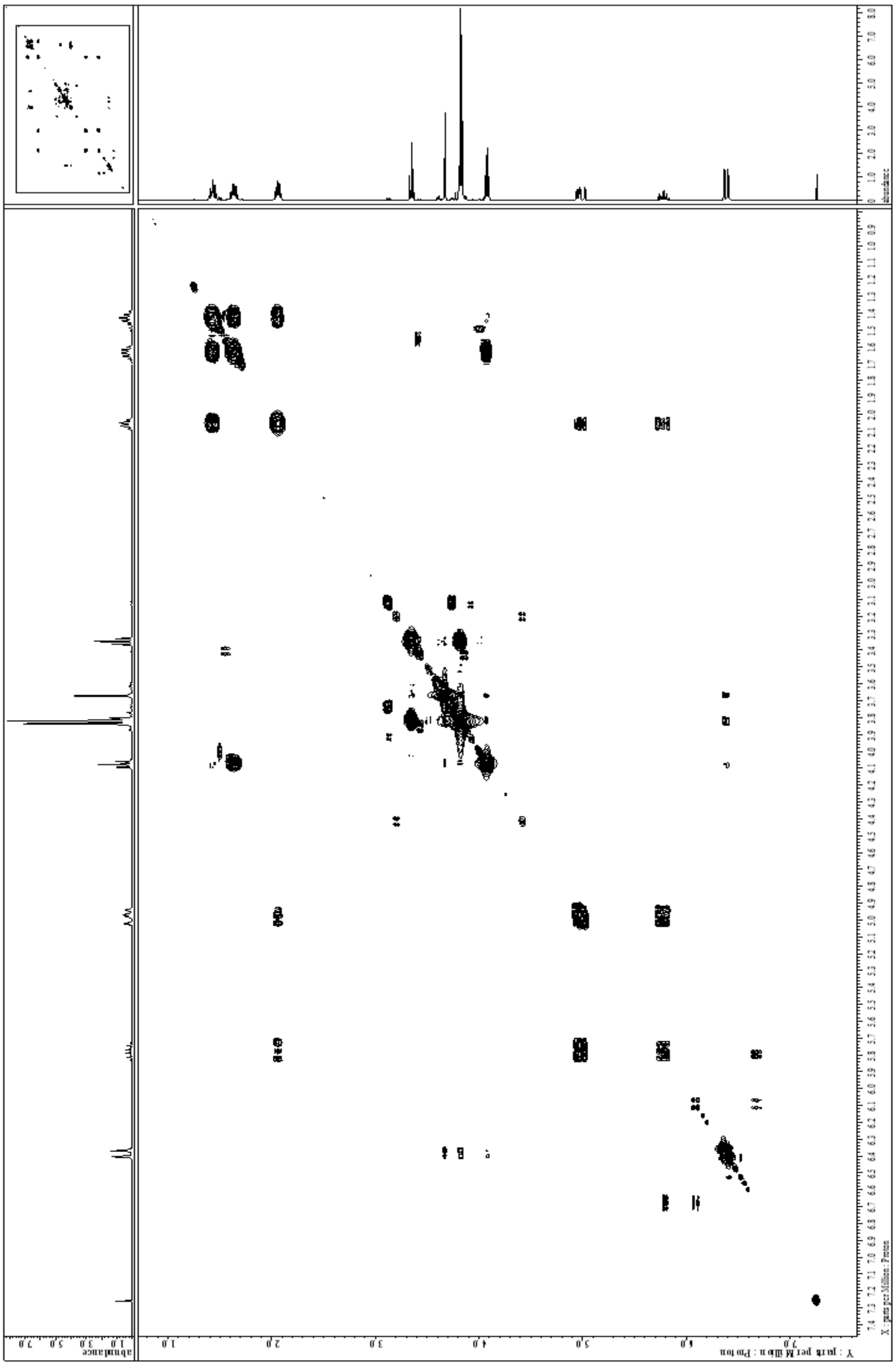

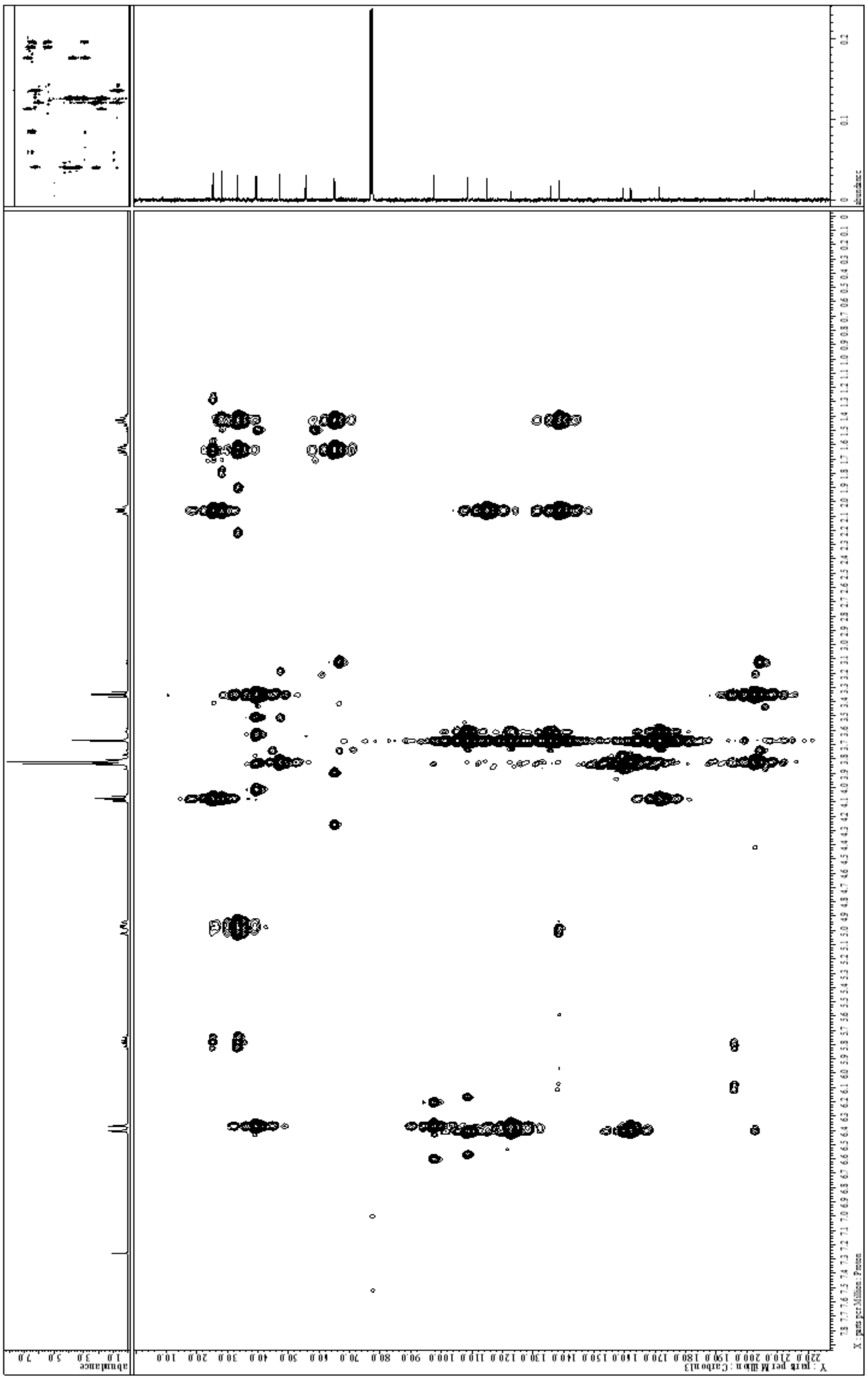

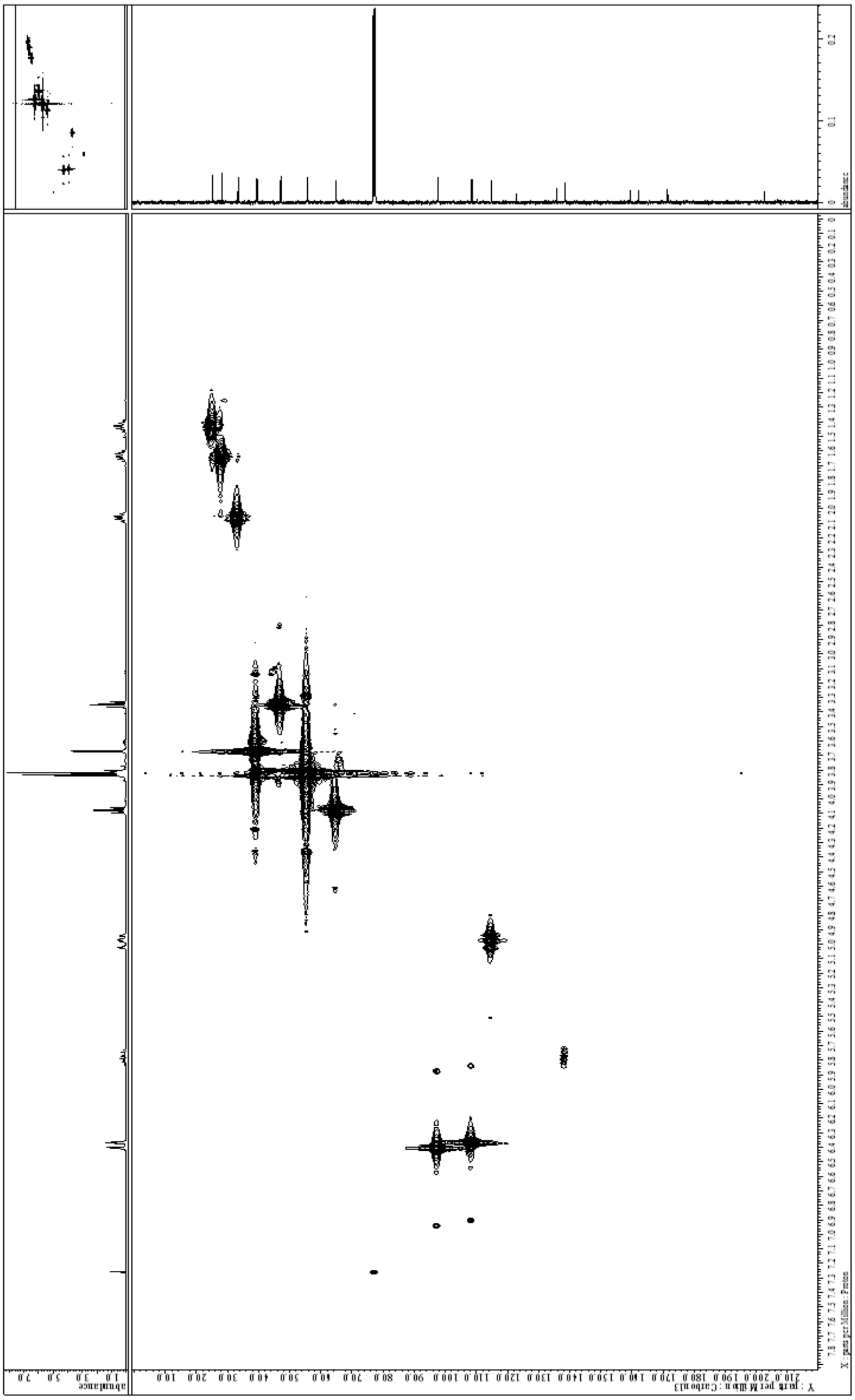

Figure S2. NMR spectrums of Hex-5-en-1-yl 2-(2-(3-chloropropanoyl)-3,5-dimethoxyphenyl) acetate (**4**)

Synthetic procedures and characterization data for the compound **5**

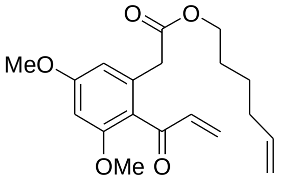

5

Hex-5-en-1-yl 2-(2-acryloyl-3,5-dimethoxyphenyl)acetate (5)

A flame-dried 500 mL round-bottom flask equipped with a magnetic stir bar was charged with anhydrous DCM (170 mL), and compound 4 (2.96 g, 8.0 mmol) was added. TEA (2.24 mL, 16 mmol) was added, and the mixture was stirred at room temperature overnight. The reaction mixture, to which sat. aq. NH_4Cl was added, was extracted with DCM (3 × 75 mL) using a separatory funnel. The obtained organic layer was dried over $MgSO_4$, filtered, and concentrated using a rotary evaporator. The reaction mixture was purified using flash column chromatography (hexane/EtOAc, 8:2) to obtain **5** (2.37 g, 89%).

TLC R_f= 0.42 (hexane/EtOAc, 7:3). ^{1}H NMR (400 MHz, $CDCl_3$) δ 6.68 (dd, *J* = 17.4, 10.5 Hz, 1H, H-10), 6.42 (d, *J* = 2.3 Hz, 1H, H-4), 6.40 (d, *J* = 2.3 Hz, 1H, H-6), 6.09 (dd, *J* = 17.4, 1.4 Hz, 2H, H-11), 5.79 (m, 2H, H-11), 5.76 (m, 1H, H-5′), 4.99 (m, 2H, H-6′), 4.94 (m, 2H, H-6′), 4.05 (t, *J* = 6.9 Hz, 2H, H-1′), 3.82 (s, 3H, H-5 OMe), 3.77 (s, 3H, H-7 OMe), 3.61 (s, 2H, H-2), 2.04 (m, 2H, H-4′), 1.40 (m, 2H, H-3′); ^{13}C NMR (100 MHz, $CDCl_3$) δ 195.8, 171.2, 161.8, 159.3, 138.5, 138.2, 135.5, 128.5, 122.3, 114.9, 107.9, 97.7, 64.9, 55.8, 38.9, 33.3, 28.1, 25.2; HRESIMS *m/z* 333.1632 $[M + H]^+$ (calcd for $C_{19}H_{25}O_5$, 333.1702).

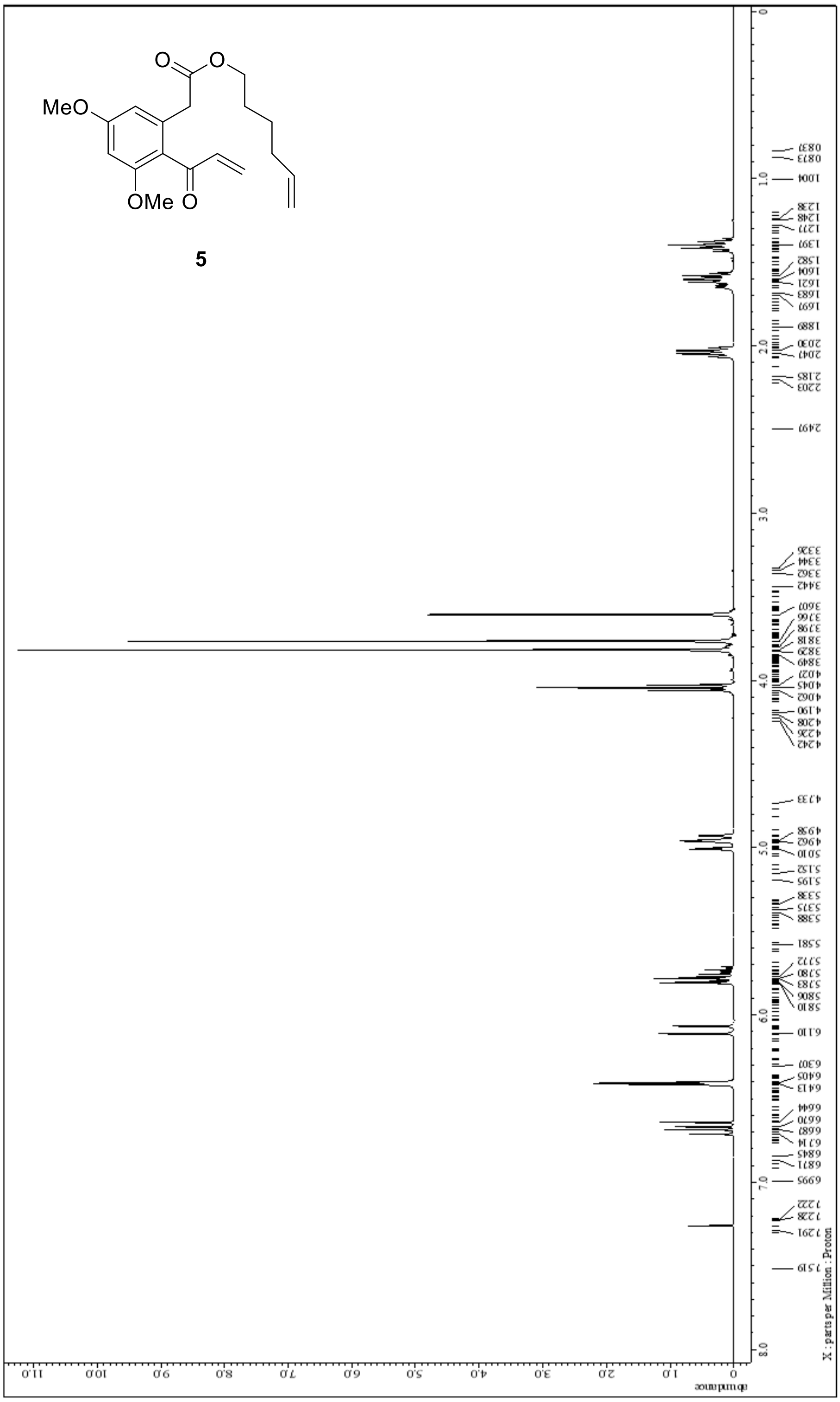
MeO
OMe O
5

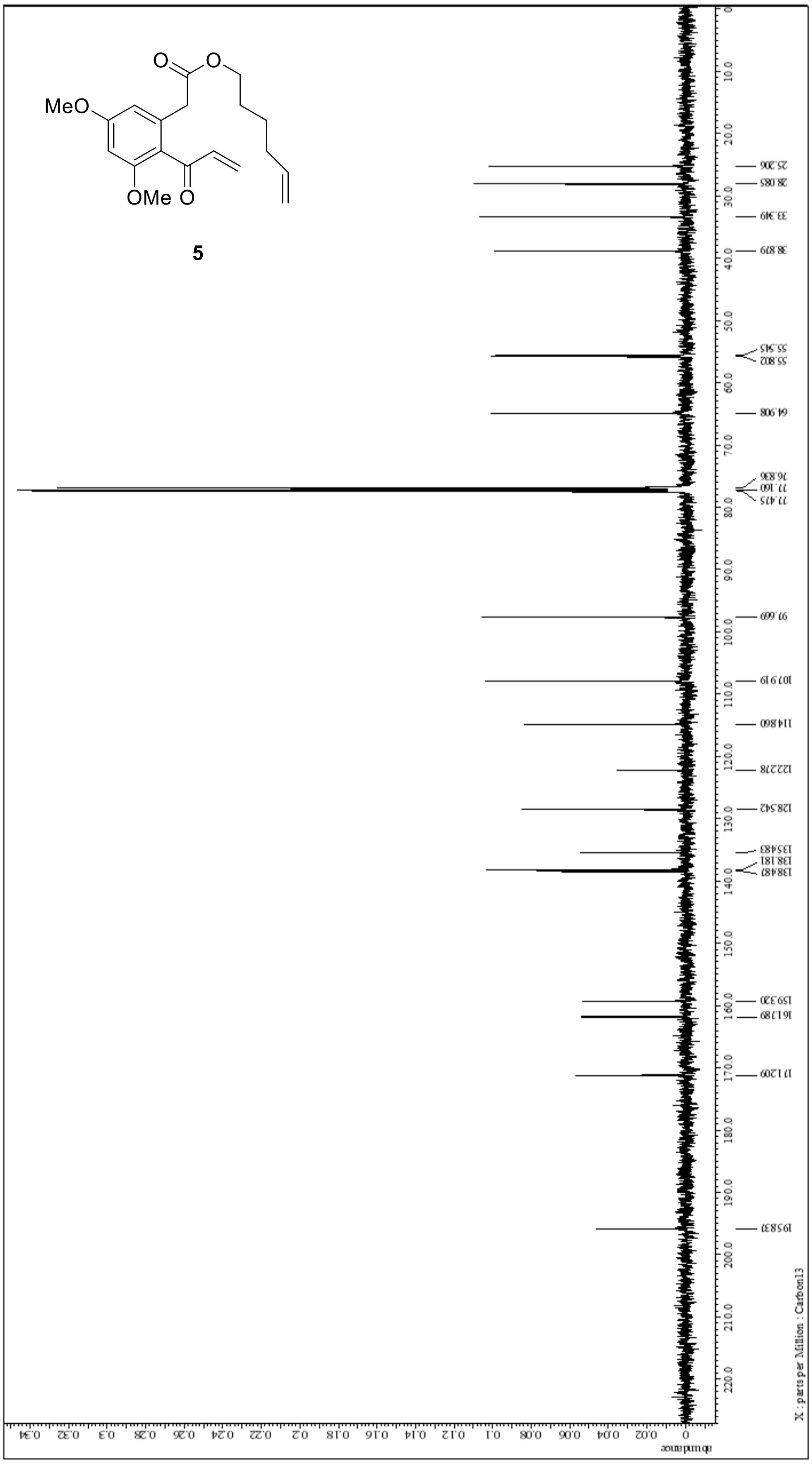
MeO
OMe
O
O
O
5
25.206
28.085
33.319
38.879
55.545
55.802
64.908
76.836
77.160
77.415
97.669
107.919
114.860
122.278
128.542
135.483
138.181
138.487
159.330
161.789
171.209
195.837
X : parts per Million : Carbon13
abundance

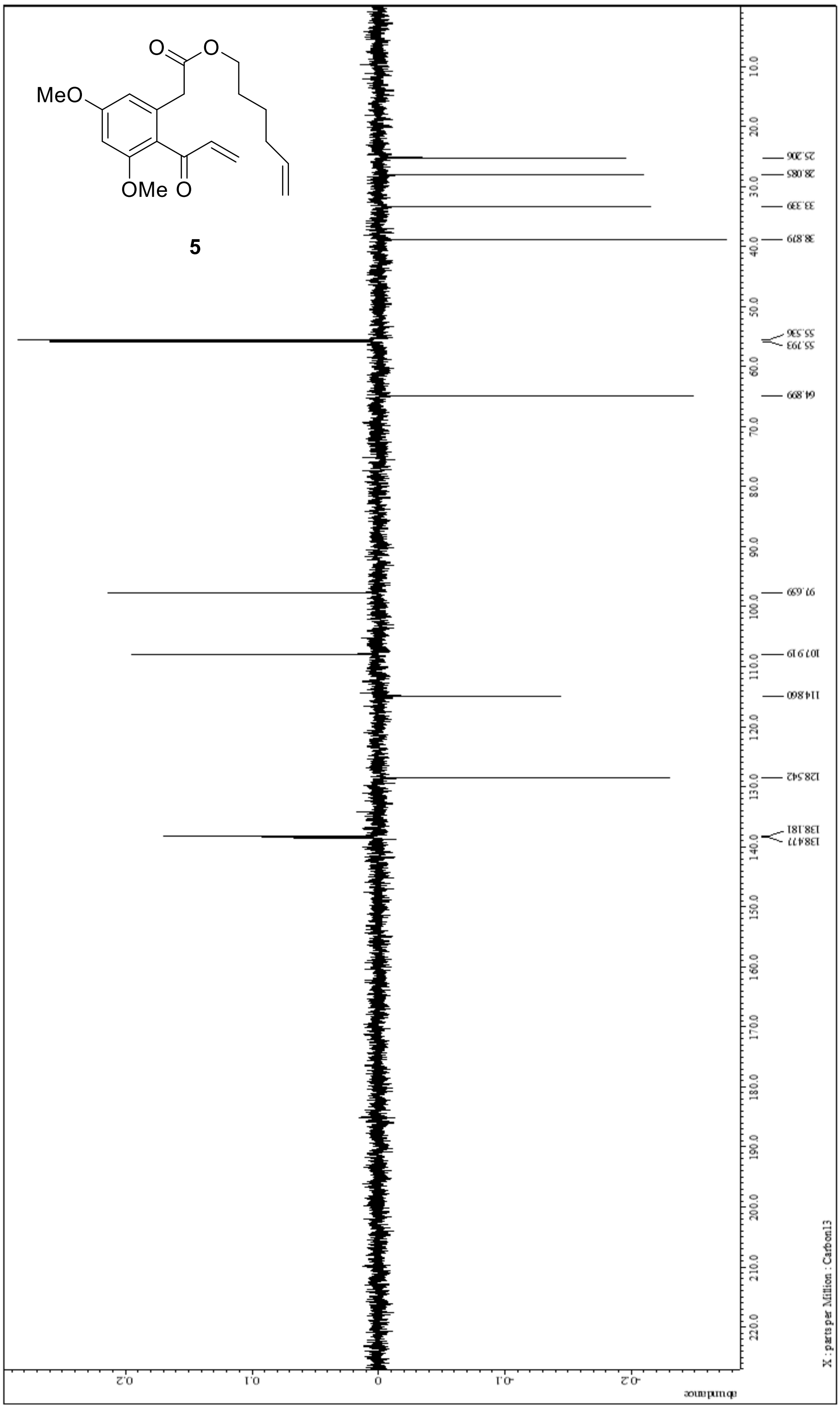
MeO
OMe
O
5
X : parts per Million : Carbon13
abundance
25.206
28.085
33.339
38.879
55.536
55.793
64.899
97.659
107.919
114.860
128.542
138.181
138.477

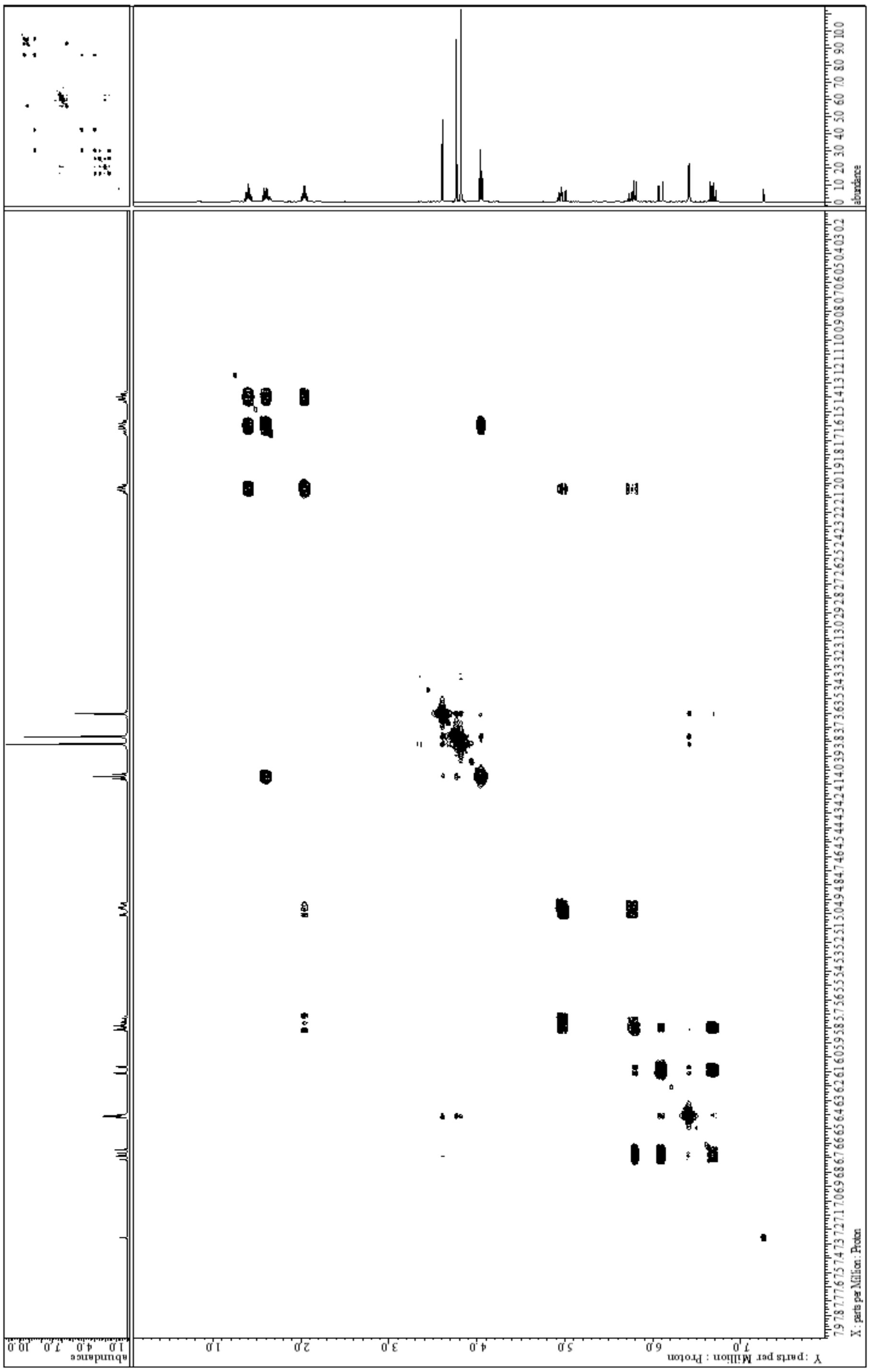

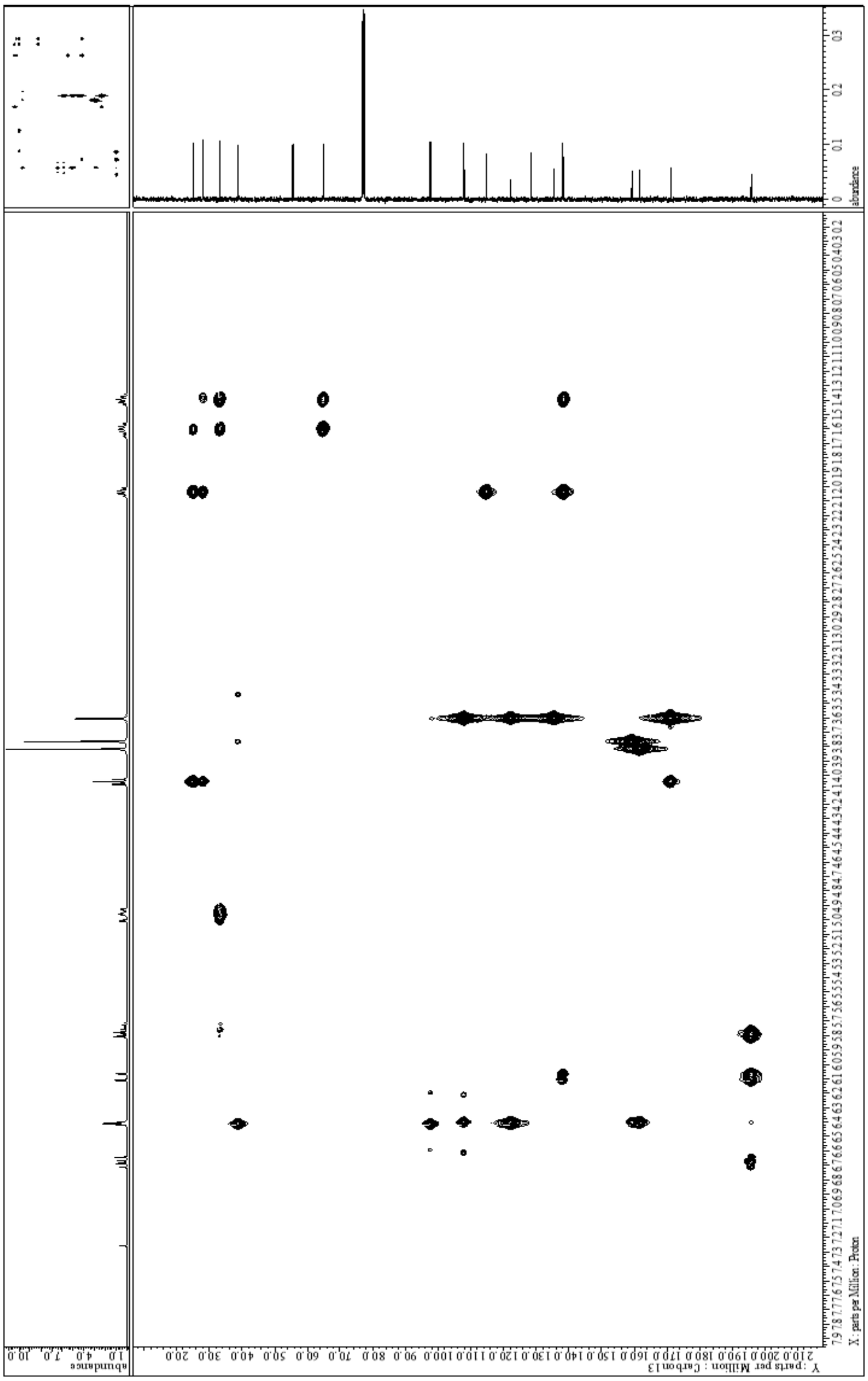
abundance
X : parts per Million : Proton
Y : parts per Million : Carbon13
abundance

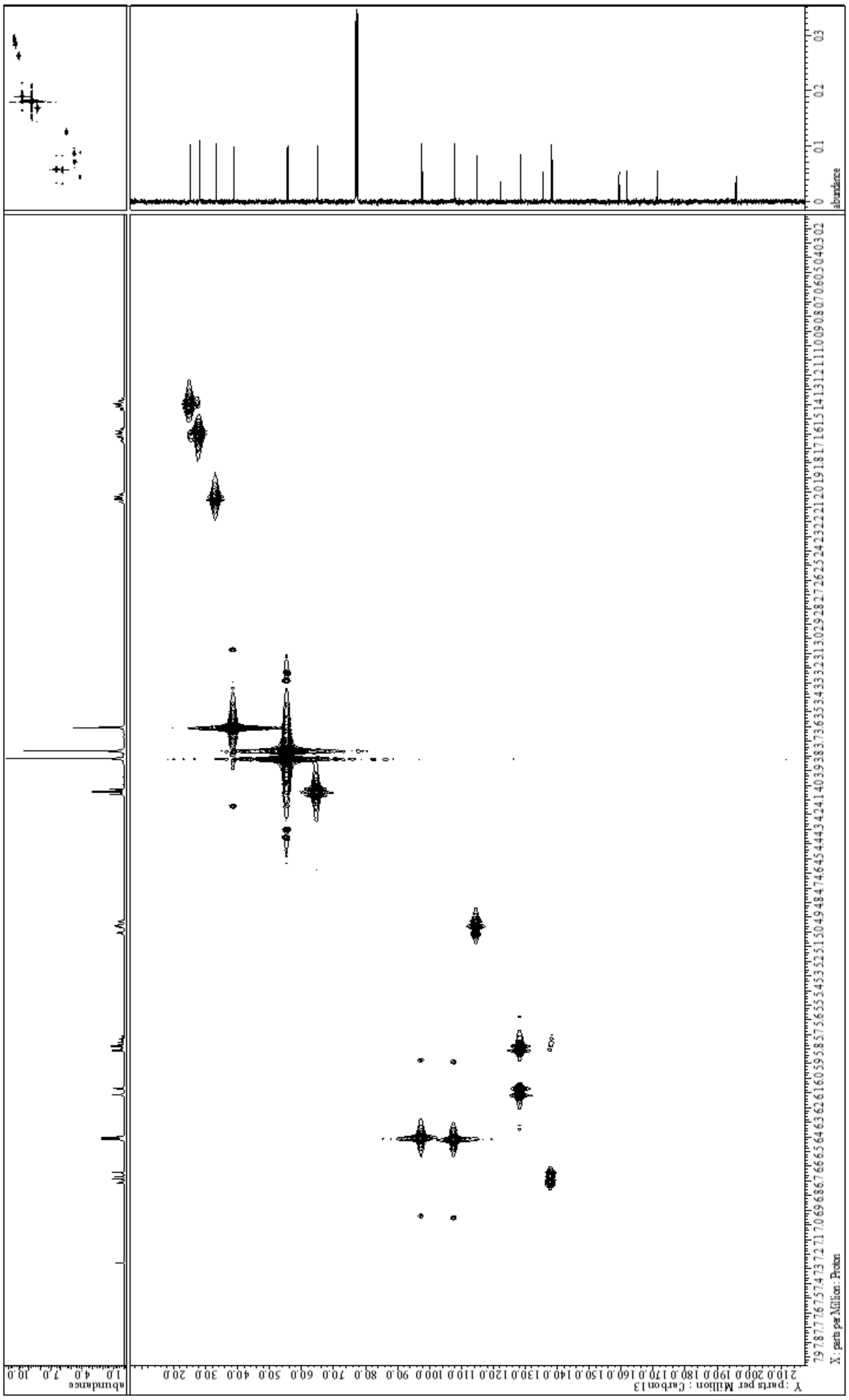

Figure S3. NMR spectrums of Hex-5-en-1-yl 2-(2-acryloyl-3,5-dimethoxyphenyl)acetate (**5**)

Synthetic procedures and characterization data for the compound **6**

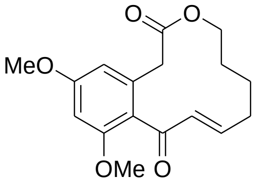

6

(*E*)-11,13-dimethoxy-4,5,6,7-tetrahydro-2*H*-benzo[*d*][1]oxacyclododecine-2,10(1*H*)-dione (6)

Anhydrous toluene (790 mL) was charged into a flame-dried 2000 mL round-bottom flask equipped with a magnetic stir bar, and compound **5** (1.04 g, 3.15 mmol) was added. Argon gas was bubbled through the mixture for 2h. After bubbling, Grubbs II (534 mg, 20 mol%) catalyst dissolved in toluene (40 mL) was added and stirred at 80 °C for 1 h. The reaction mixture was concentrated using a rotary evaporator and purified using flash column chromatography (hexane/EtOAc, 8:2) to obtain a separated mixture of **6** (335 mg, 35%) and **7** (95 mg, 10%). (35%, *E*-isomer)

TLC R_f= 0.38 (hexane/EtOAc, 6:4). ^{1}H NMR (400 MHz, $CDCl_3$) δ 6.55 (d, *J* = 1.8, 1H, H-4), 6.53 (d, *J* = 2.3, 1H, H-6), 6.46 (dd, *J* = 15.8, 7.8, 1H, H-11), 6.21 (dt, *J* = 15.6, 1.2 Hz, 1H, H-10), 4.06 (t, *J* = 4.6, 2H, H-15), 3.83 (s, 3H, H-5 OMe), 3.75 (s, 3H, H-7 OMe), 3.40 (s, 2H, H-2), 2.31 (m, 2H, H-14), 1.71 (m, 2H, H-12; 2H, H-13); ^{13}C NMR (100 MHz, $CDCl_3$) δ 201.3, 172.2, 162.8, 159.4, 159.0, 133.9, 133.7, 125.6, 108.3, 98.5, 68.1, 56.2, 56.0, 40.1, 34.8, 28.3, 27.2; HRESIMS *m/z* 305.1378 $[M + H]^+$ (calcd for $C_{17}H_{21}O_5$, 305.1389).

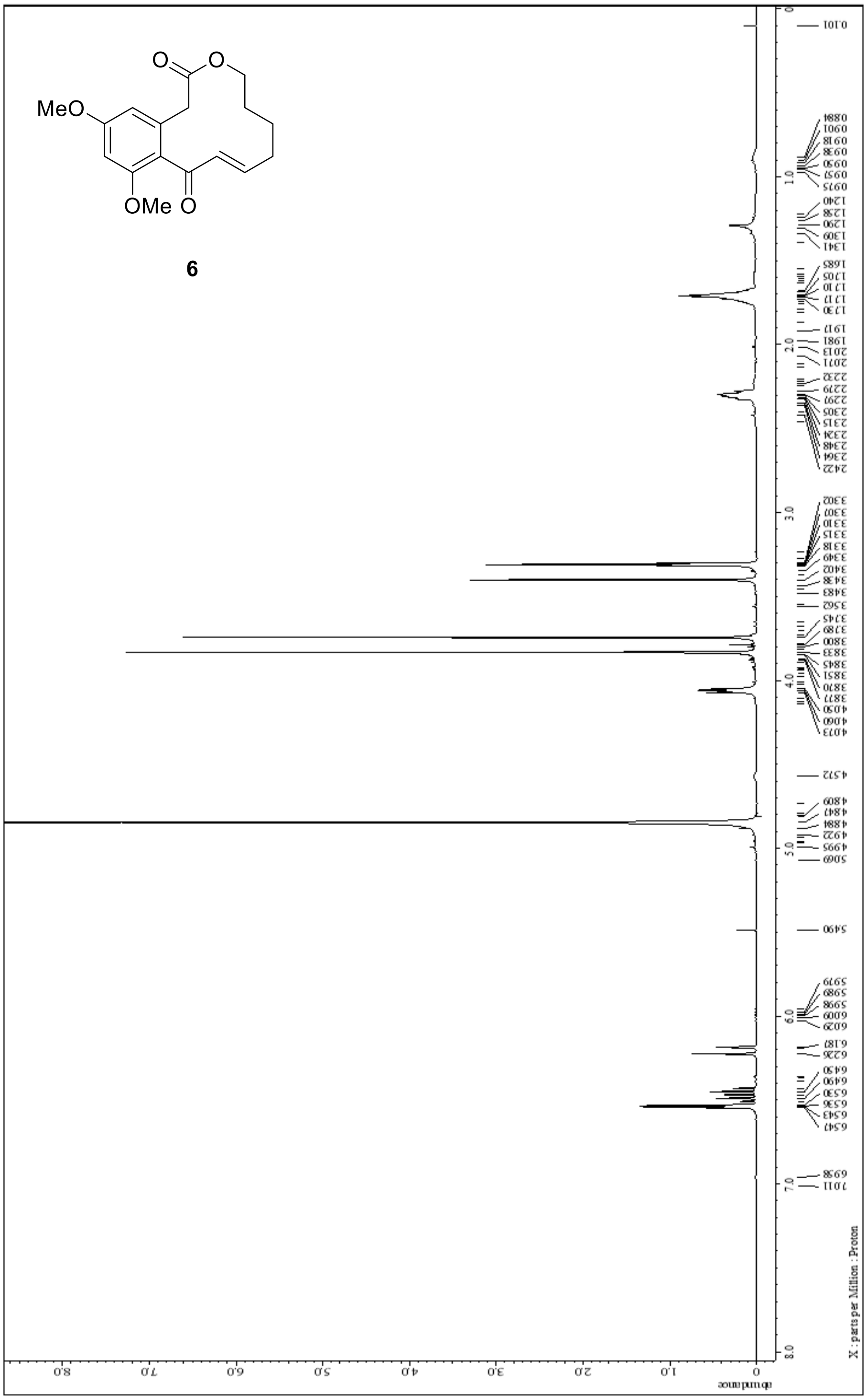
MeO
OMe
6
X : parts per Million : Proton
abundance

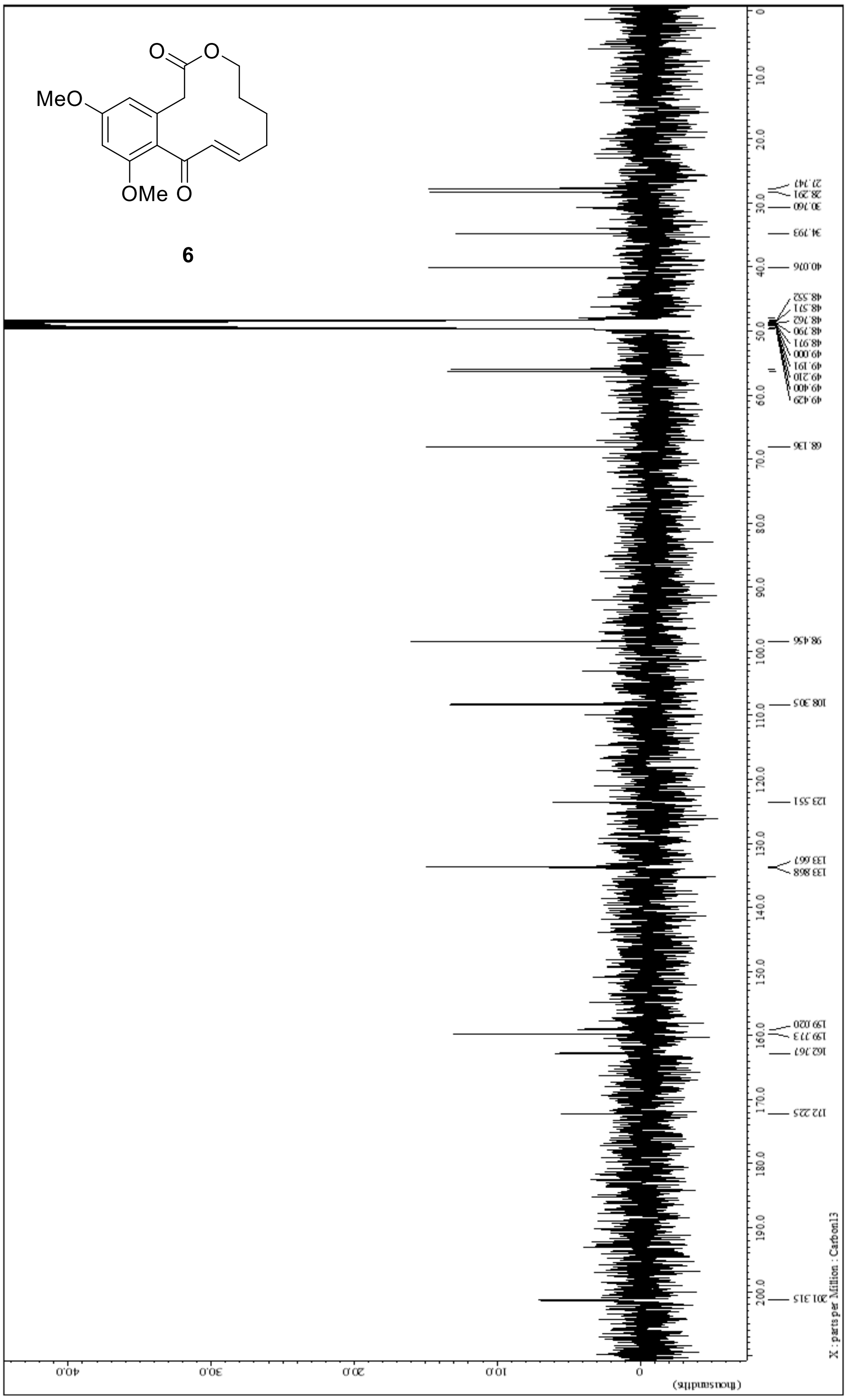
MeO
OMe
6

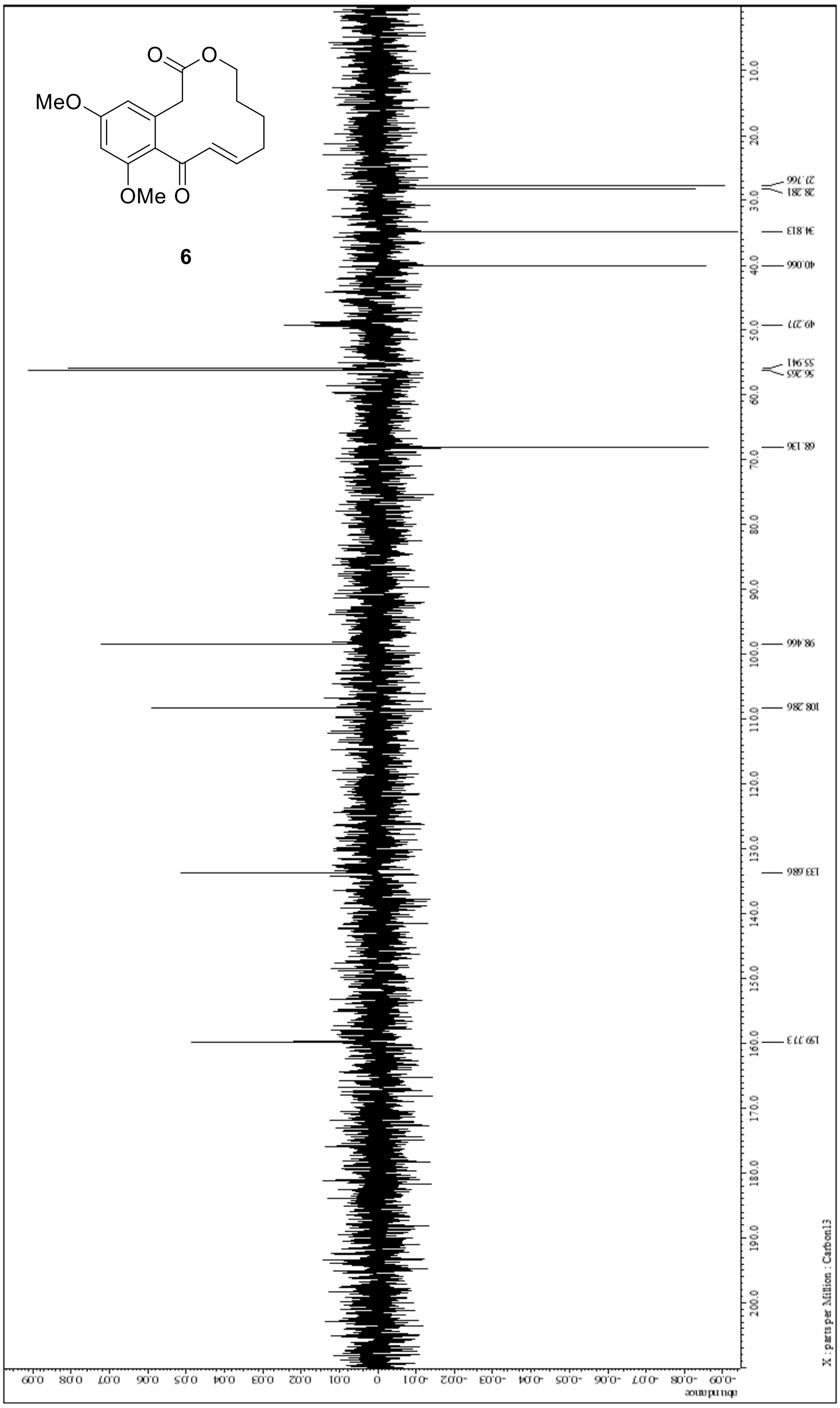

Figure S4. NMR spectrums of (*E*)-11,13-dimethoxy-4,5,6,7-tetrahydro-2*H*-benzo[*d*][1]oxacyclododecine-2,10(1*H*)-dione (**6**)

Synthetic procedures and characterization data for the compound **7**

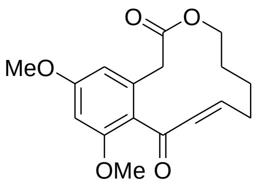

7

11,13-dimethoxy-4,5,6,7-tetrahydro-2*H*-benzo[*d*][1]oxacyclododecine-2,10(1*H*)-dione (7)

Anhydrous toluene (790 mL) was charged into a flame-dried 2000 mL round-bottom flask equipped with a magnetic stir bar, and compound **5** (1.04 g, 3.15 mmol) was added. Argon gas was bubbled through the mixture for 2h. After bubbling, Grubbs II (534 mg, 20 mol%) catalyst dissolved in toluene (40 mL) was added and stirred at 80 °C for 1 h. The reaction mixture was concentrated using a rotary evaporator and purified using flash column chromatography (hexane/EtOAc, 8:2) to obtain a separated mixture of **6** (335 mg, 35%) and **7** (95 mg, 10%). (10%, *Z*-isomer)

TLC R_f= 0.48 (hexane/EtOAc, 6:4). ^{1}H NMR (400 MHz, $CDCl_3$) δ 6.47 (m, 1H, H-10), 6.41 (m, 1H, H-4), 6.37 (m, 1H, H-6), 5.90 (dt, J = 11.9, 8.2 Hz, 1H, H-11), 4.01 (m, 2H, H-15), 3.83 (s, 3H, H-5 OMe), 3.74 (s, 3H, H-7 OMe), 3.41 (s, 2H, H-2), 2.29 (m, 2H, H-12), 1.60 (m, 2H, H-13), 1.60 (m, 2H, H-14); ^{13}C NMR (100 MHz, $CDCl_3$) δ 197.6, 170.7, 161.7, 159.3, 144.1, 134.4, 133.1, 124.5, 108.0, 97.9, 67.6, 56.0, 55.6, 39.3, 28.2, 27.6, 24.8; HRESIMS *m/z* 305.1378 $[M + H]^+$ (calcd for $C_{17}H_{21}O_5$, 305.1389).

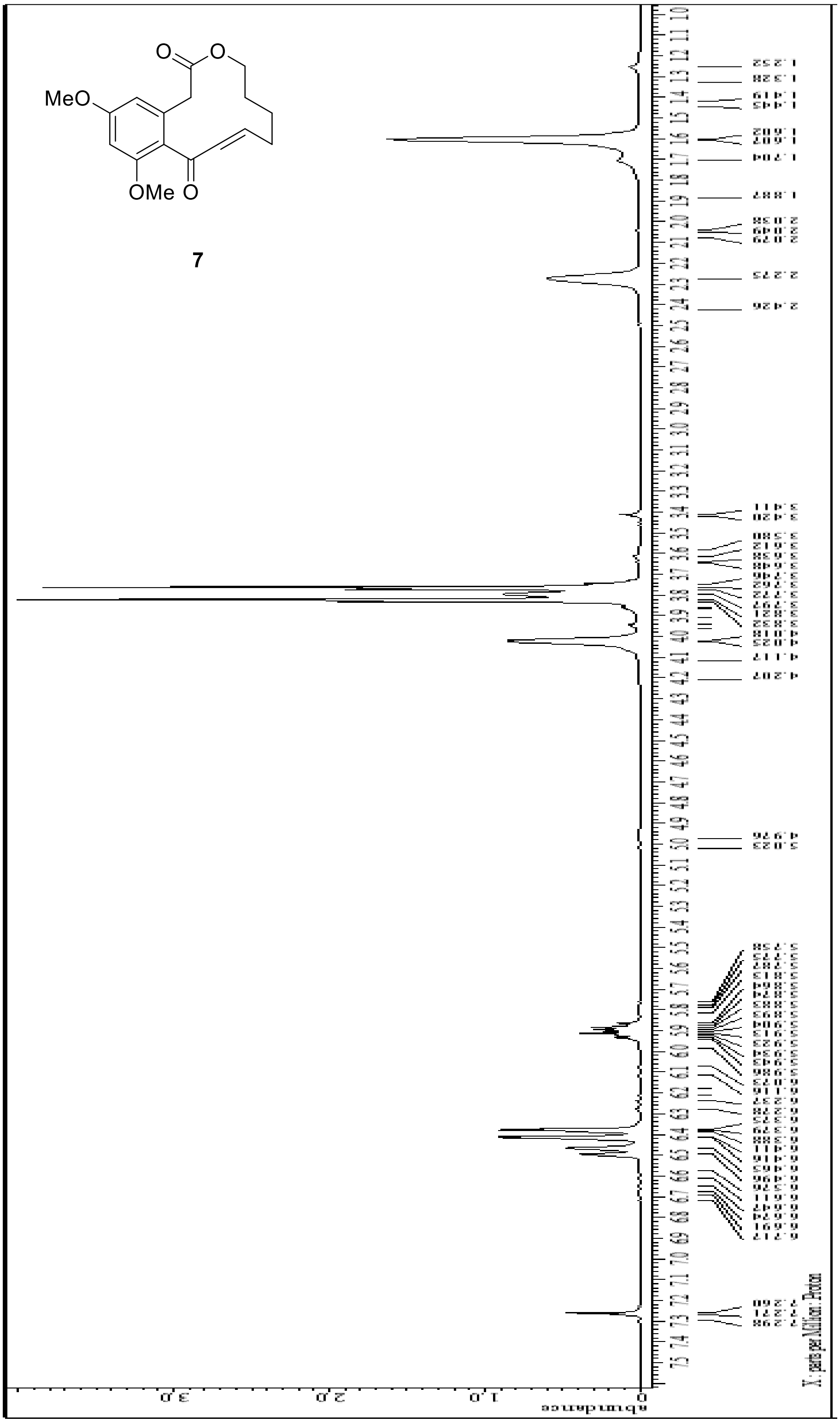
MeO
OMe
O
7
abundance
X : parts per Million : Proton

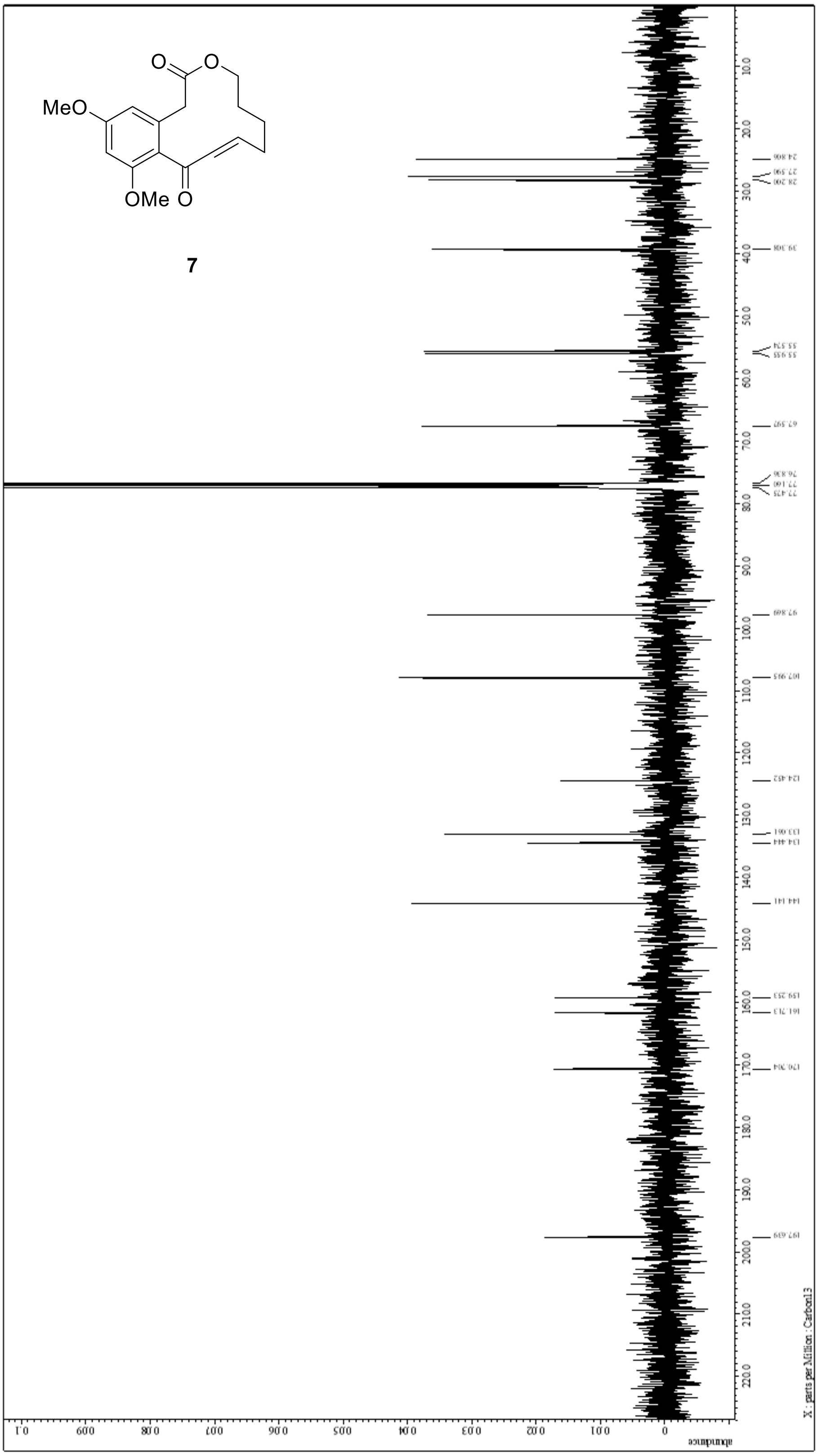

7

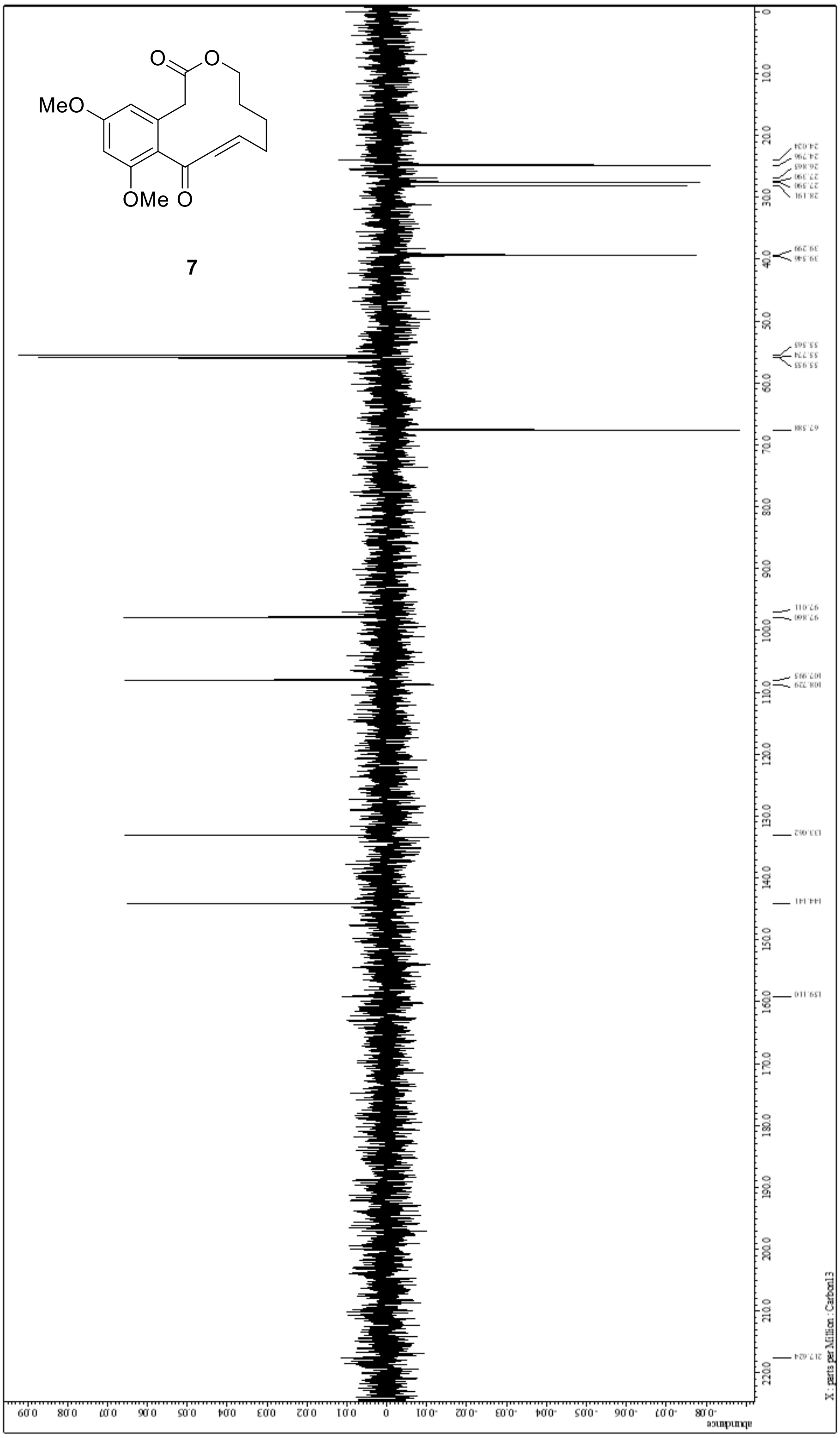
MeO
OMe
7

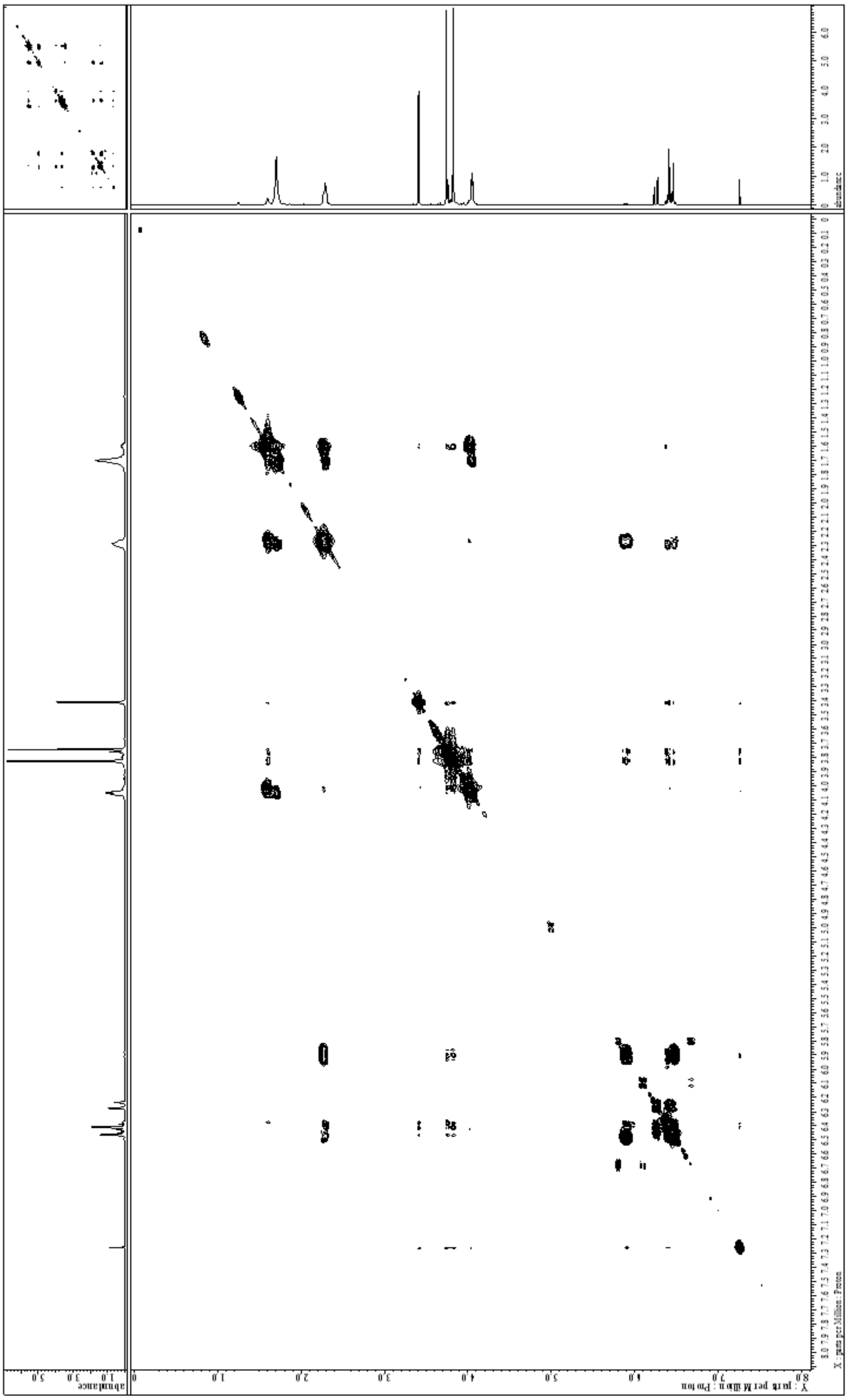

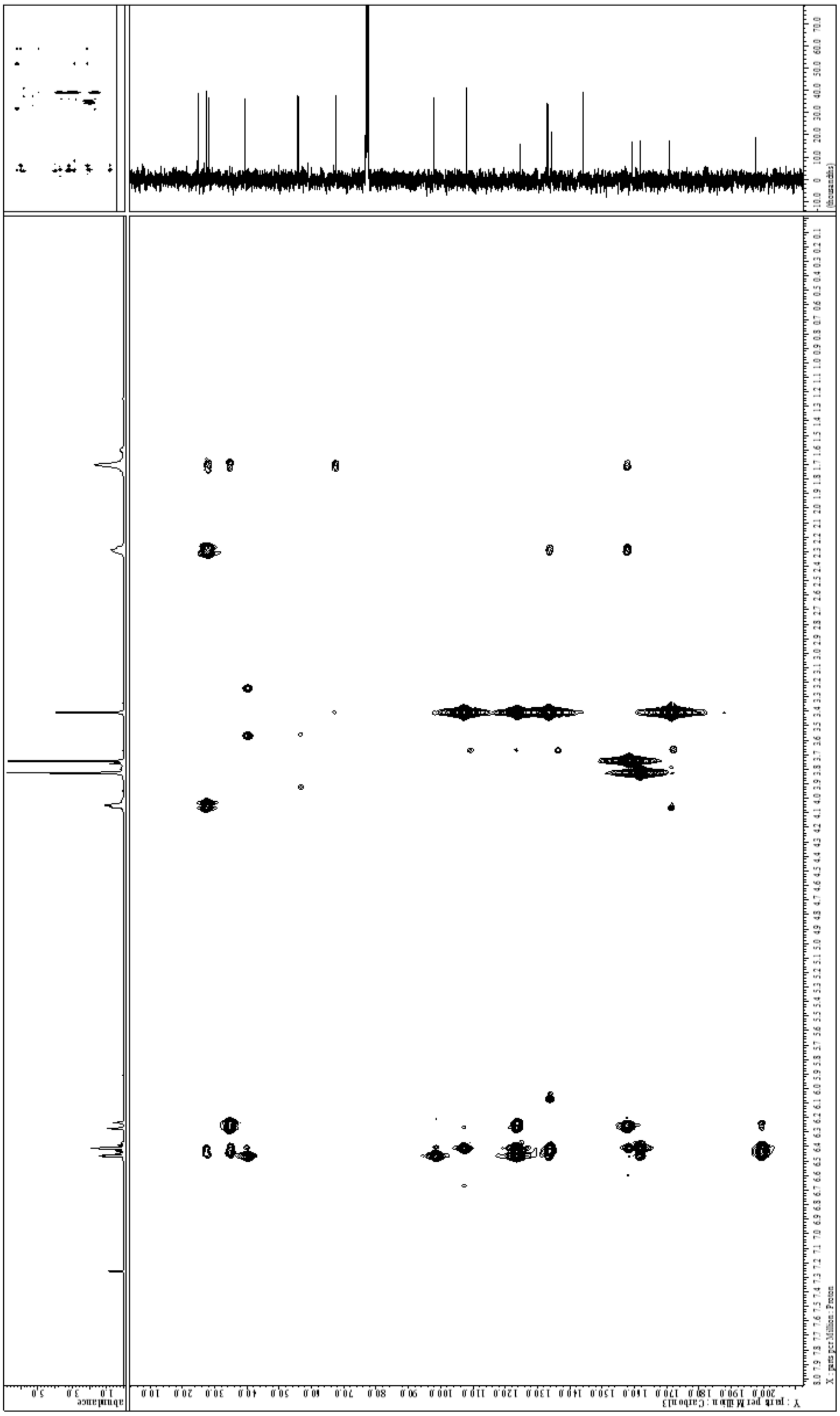

Y : parts per Million : Carbon13
X : parts per Million : Proton
abundance

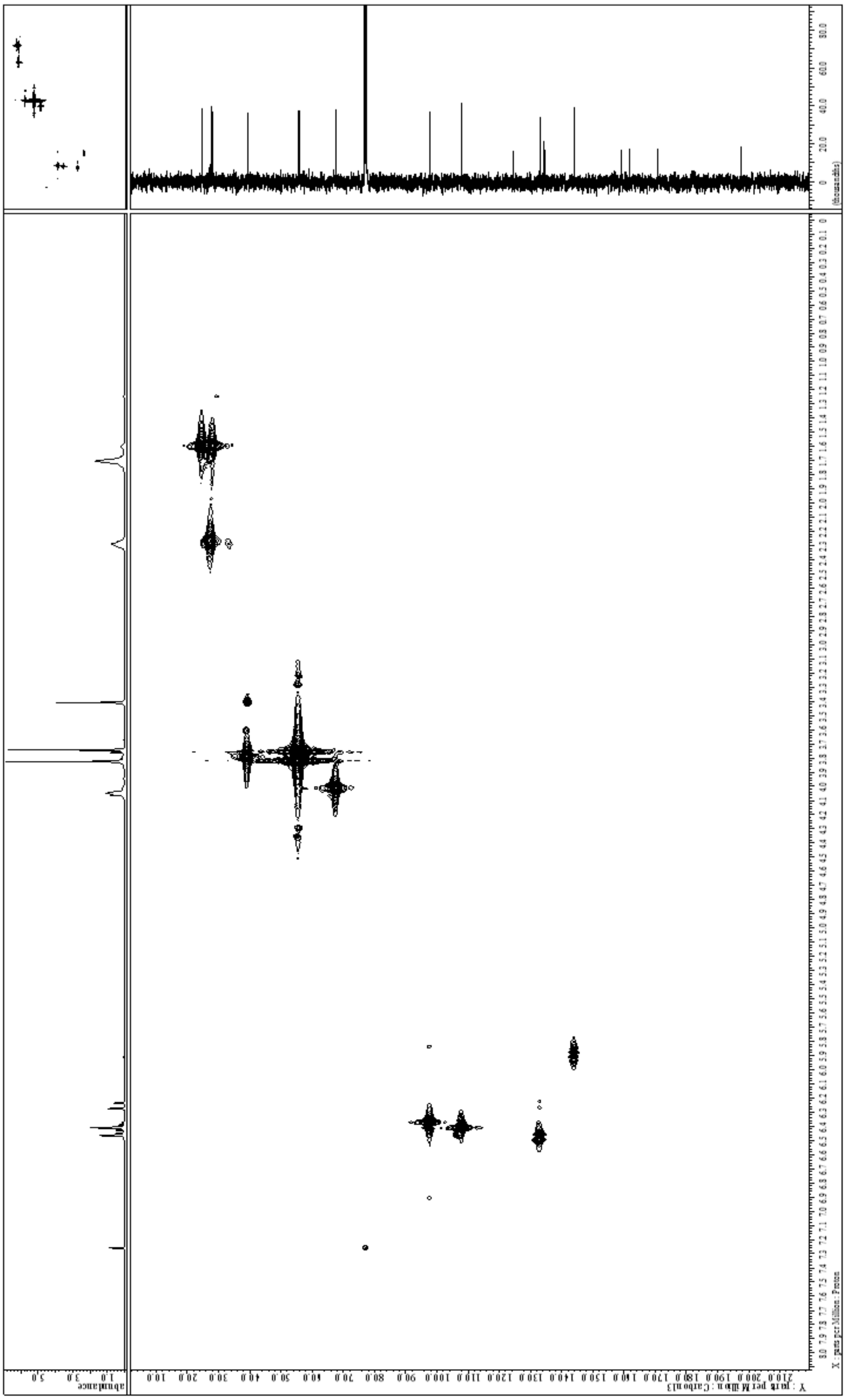

Figure S5. NMR spectrums of 11,13-dimethoxy-4,5,6,7-tetrahydro-2*H*-benzo[*d*][1] oxacyclododecine-2,10(1*H*)-dione (**7**)

Synthetic procedures and characterization data for the compound **8**

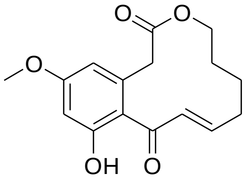

8

(*E*)-11-hydroxy-13-methoxy-4,5,6,7-tetrahydro-2*H*-benzo[*d*][1]oxacyclododecine-2,10(1*H*)-dione (8)

A flame-dried 50 mL round-bottom flask equipped with a magnetic stir bar was charged with anhydrous ACN (10 mL), followed by adding compound **6** (200 mg, 0.66 mmol), Al powder (178 mg, 6.57 mmol), and DMSO (233 μL, 3.29 mmol). Iodine (551 mg, 2.17 mmol) was added, and the mixture was stirred at 80 °C for 1 h. After completion of the reaction, the mixture was cooled to room temperature and diluted with aq. HCl (2 M, 5 mL). The mixture was extracted with EtOAc (3 × 20 mL) using a separatory funnel, and the organic layer was dried over $MgSO_4$. After filtration, the mixture was concentrated using a rotary evaporator, and the reaction mixture was purified using flash column chromatography (hexane/EtOAc, 7:3) to obtain **8** (140 mg, 73%).

TLC R_f= 0.53 (hexane/EtOAc, 1:1). ^{1}H NMR (600 MHz, $CDCl_3$) δ 6.70 (m, 1H, H-10), 6.60 (m, 1H, H-11), 6.42 (d, *J* = 2.6 Hz, 1H, H-6), 6.33 (t, *J* = 2.9 Hz, 1H, H-4), 4.17 (dd, *J* = 6.2, 2.5 Hz,, 2H, H-15), 3.83 (s, 3H, OMe), 3.82 (s, 2H, H-2), 2.45 (m, 2H, H-14), 1.83 (m, 2H, H-12; 2H, H-13); ^{13}C NMR (150 MHz, $CDCl_3$) δ 196.0, 171.3, 167.0, 164.3, 148.1, 137.4, 131.7, 114.0, 113.6, 100.4, 66.3, 55.5, 43.9, 32.8, 27.1, 26.7; HRESIMS *m/z* 291.1223 $[M + H]^+$ (calcd for $C_{16}H_{19}O_5$, 291.1232).

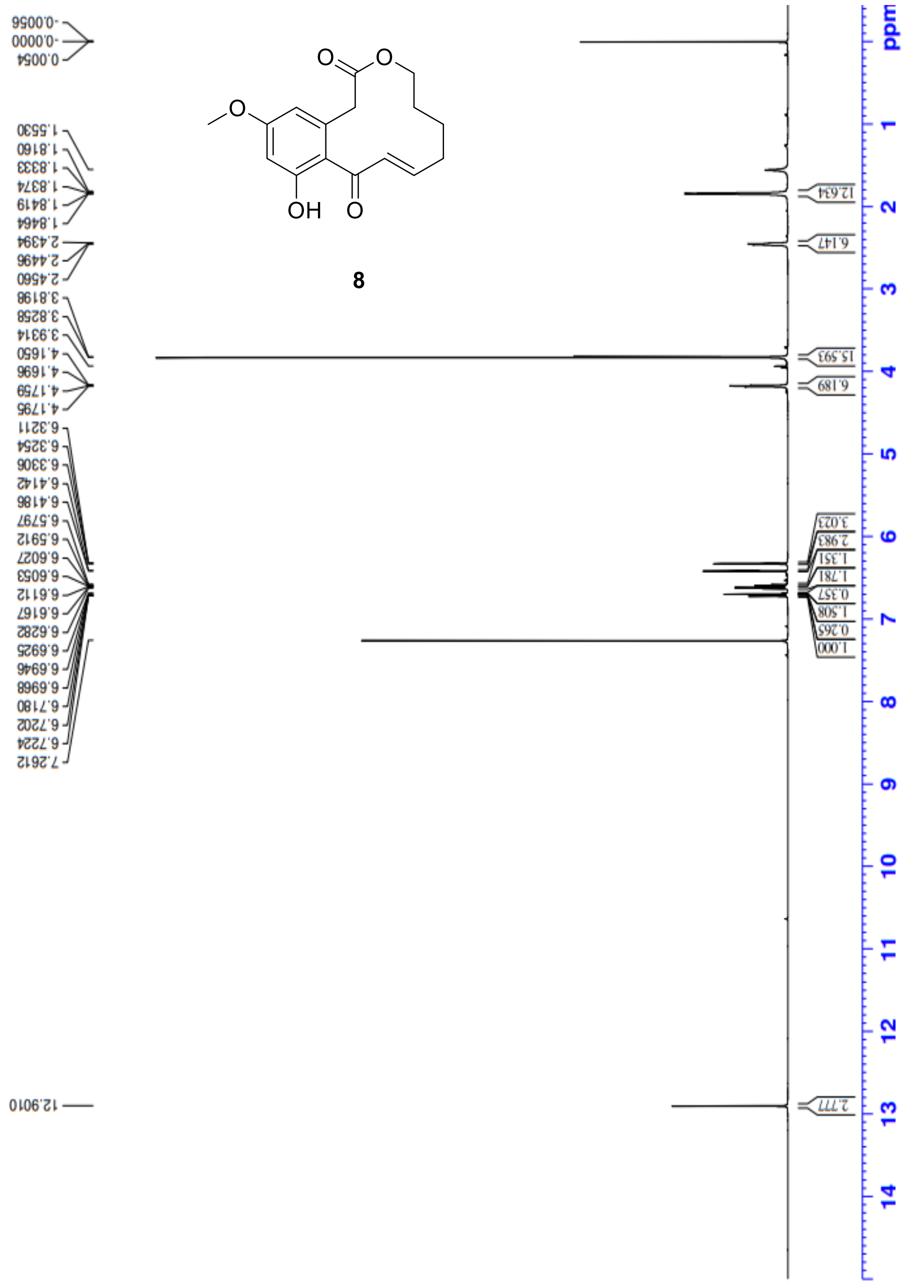
0.0054
-0.0000
-0.0056
1.5530
1.8160
1.8333
1.8374
1.8419
1.8464
2.4394
2.4496
2.4560
3.8198
3.8258
3.9314
4.1650
4.1696
4.1759
4.1796
6.3211
6.3254
6.3306
6.4142
6.4186
6.5797
6.5912
6.6027
6.6053
6.6112
6.6167
6.6282
6.6925
6.6946
6.6968
6.7180
6.7202
6.7224
7.2612
12.9010
OH
O
12.634
6.147
15.593
6.189
3.023
2.983
1.351
1.781
0.357
1.508
0.265
1.000
2.777
ppm
1
2
3
4
5
6
7
8
9
10
11
12
13
14

8

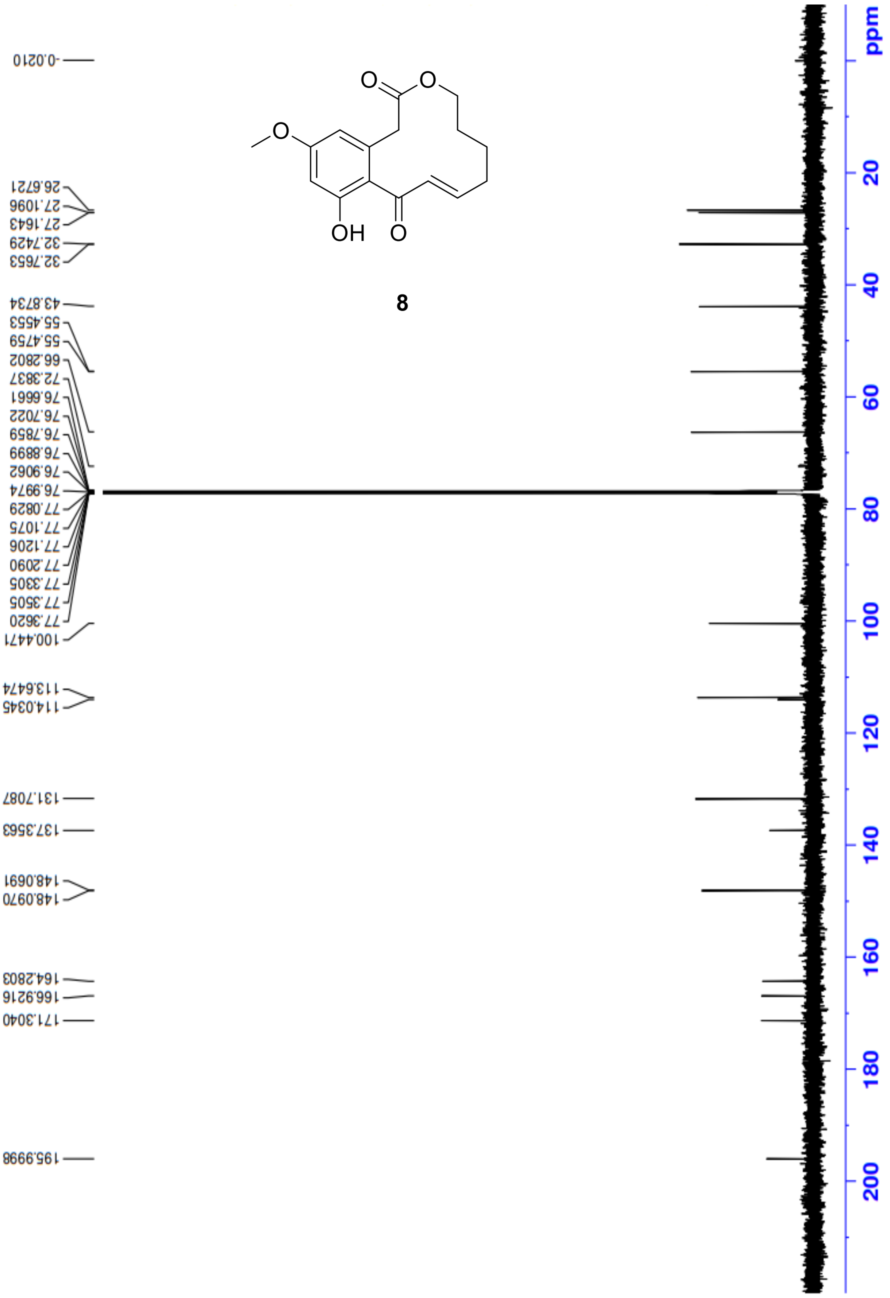
-0.0210
26.6721
27.1096
27.1643
32.7429
32.7653
43.8734
55.4553
55.4759
66.2802
72.3837
76.6661
76.7022
76.7859
76.8899
76.9062
76.9974
77.0829
77.1075
77.1206
77.2090
77.3305
77.3505
77.3620
100.4471
113.6474
114.0345
131.7087
137.3563
148.0691
148.0970
164.2803
166.9216
171.3040
195.9998
O
O
O
OH
O
8
ppm
20
40
60
80
100
120
140
160
180
200

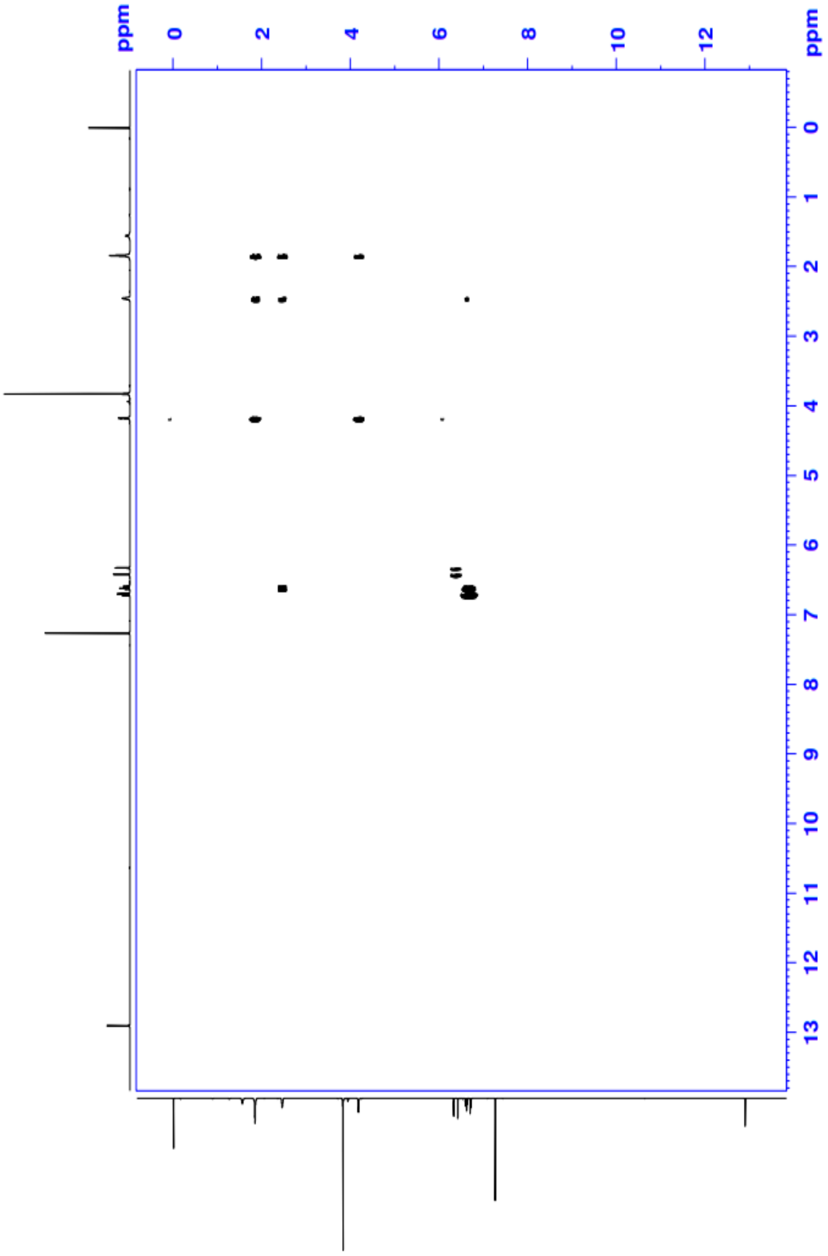
ppm
0
2
4
6
8
10
12
ppm
0
1
2
3
4
5
6
7
8
9
10
11
12
13

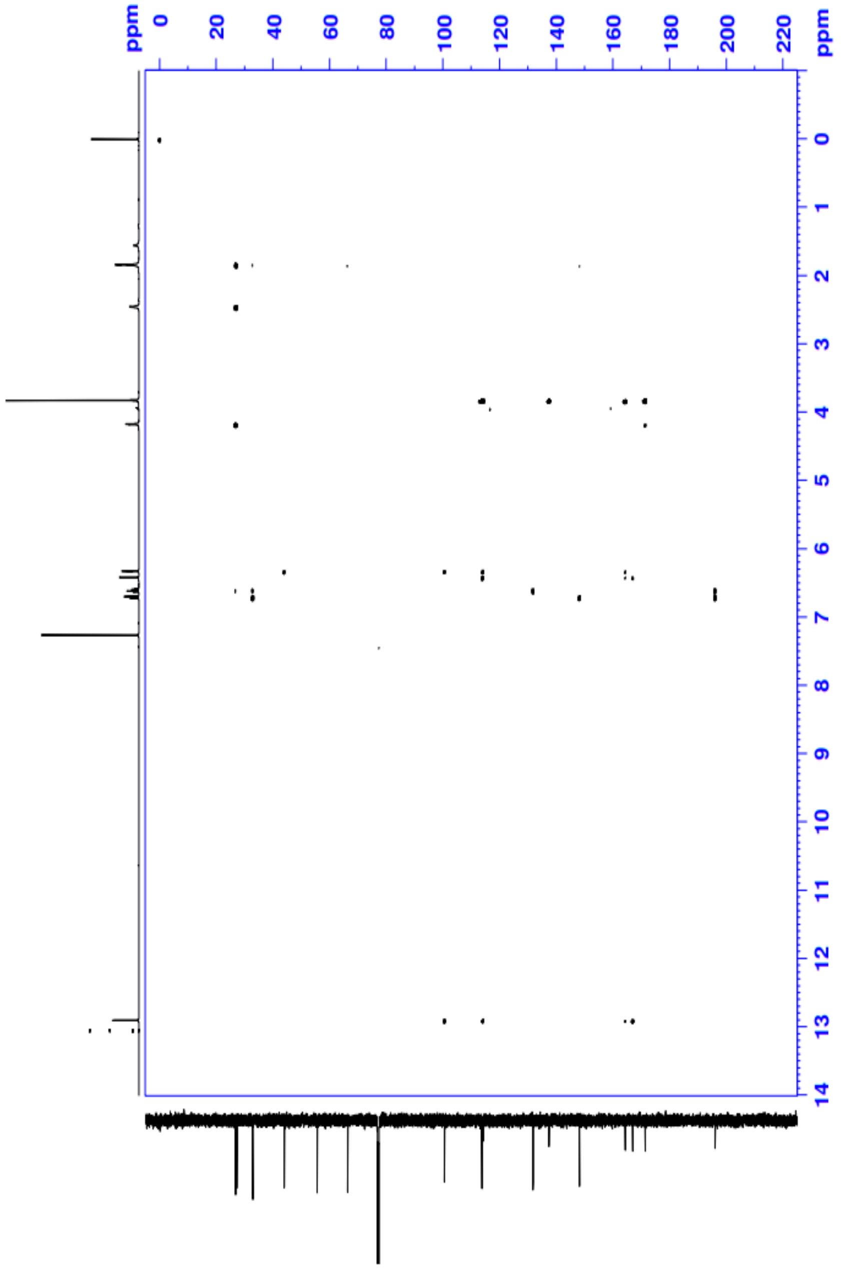
ppm
0
20
40
60
80
100
120
140
160
180
200
220
ppm
0
1
2
3
4
5
6
7
8
9
10
11
12
13
14

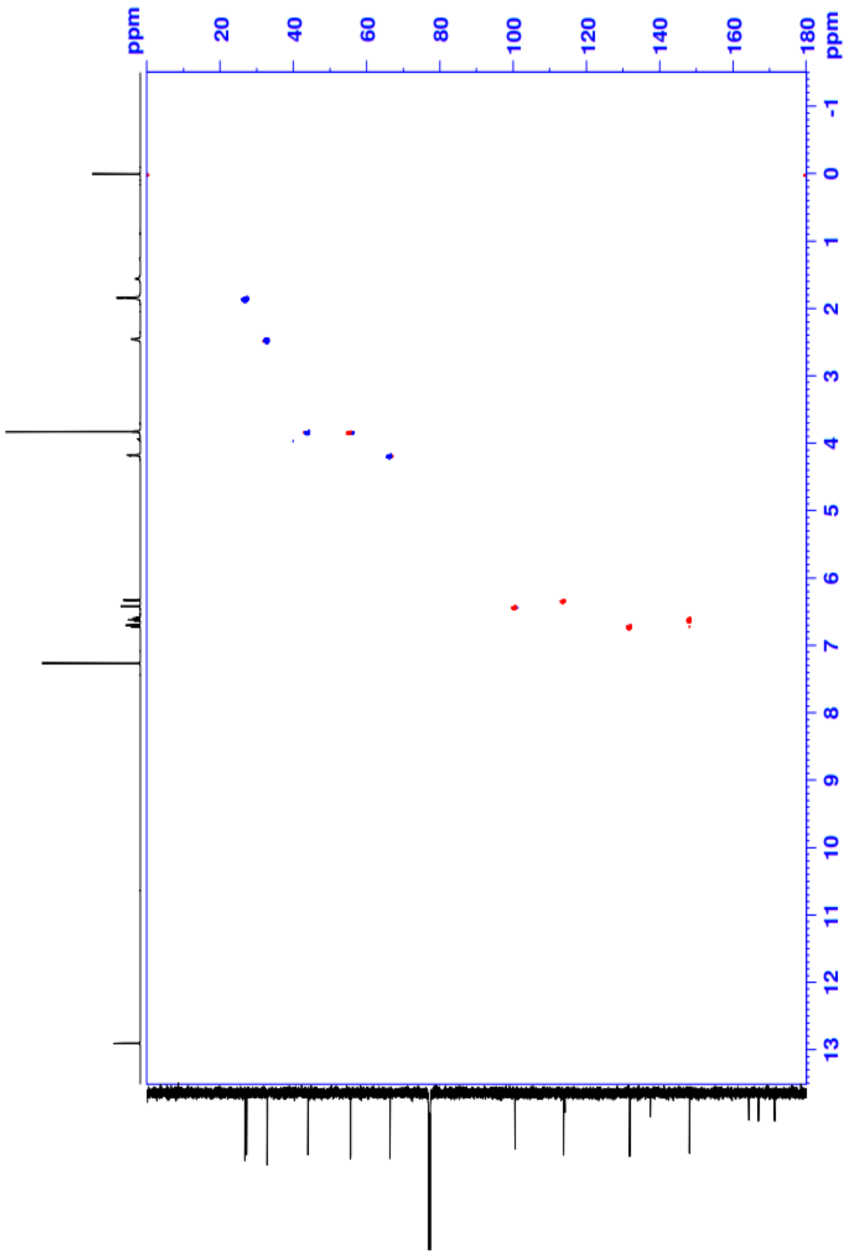

Figure S6. NMR spectrums of (*E*)-11-hydroxy-13-methoxy-4,5,6,7-tetrahydro-2*H*-benzo[*d*][1]oxacyclododecine-2,10(1*H*)-dione (**8**)

Synthetic procedures and characterization data for the compound **11**

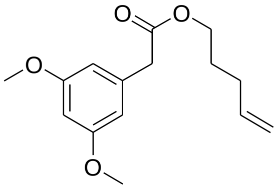

11

pent-4-en-1-yl 2-(3,5-dimethoxyphenyl)acetate (11)

A flame-dried 100 mL round-bottom flask equipped with a magnetic stir bar was charged with anhydrous DCM (10 mL), 3,5-Dimethoxyphenylacetic Acid (**1**) (1.0 g, 5.1 mmol), 4-Penten-1-ol (10) (620 μL, 6.1 mmol), and Mukaiyama reagent (2-Chloro-1-methylpyridinium iodide) (1.56 g, 6.1 mmol). The mixture was stirred at room temperature for 15 min. Then, TEA (2.13 mL, 15.3 mmol) was slowly added to the mixture, and the mixture was stirred at room temperature for 20 h. The reaction mixture was filtered and purified by flash column chromatography (hexane/EtOAc, 9:1) to give **11** (876 mg, 65%).

TLC R_f= 0.60 (hexane/EtOAc, 7:3). ^{1}H NMR (400 MHz, $CDCl_3$) δ 6.44 (d, *J* = 2.3 Hz, 1H, H-4; 1H, H-8), 6.37 (t, *J* = 2.3 Hz, 1H, H-6), 5.77 (m, 1H, H-4′), 5.00 (m, 2H, H-5′), 4.97 (m, 2H, H-5′), 4.10 (t, *J* = 6.6 Hz, 2H, H-1′), 3.78 (s, 3H, H-5 OMe; 3H, H-7 OMe), 3.55 (s, 2H, H-2′), 2.09 (m, 2H, H-3′), 1.72 (m, 2H, H-2′); ^{13}C NMR (100 MHz, $CDCl_3$) δ 171.5, 161.0, 161.0, 137.5, 136.3, 115.4, 107.4, 107.4, 99.3, 64.4, 55.4, 55.4, 41.8, 30.1, 27.9; HRESIMS *m/z* 265.1437 $[M + H]^+$ (calcd for $C_{15}H_{21}O_4$, 265.1440).

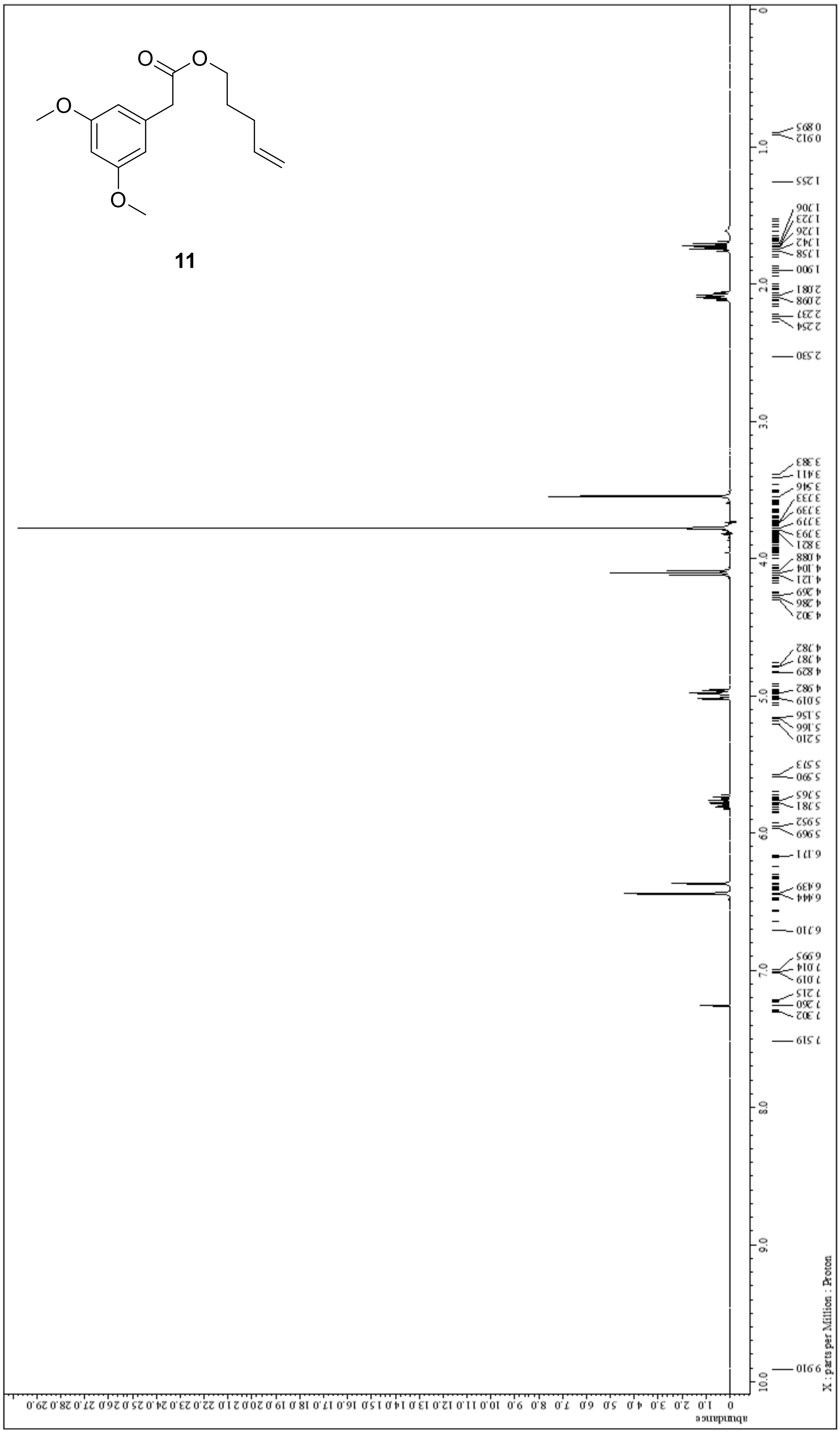

11

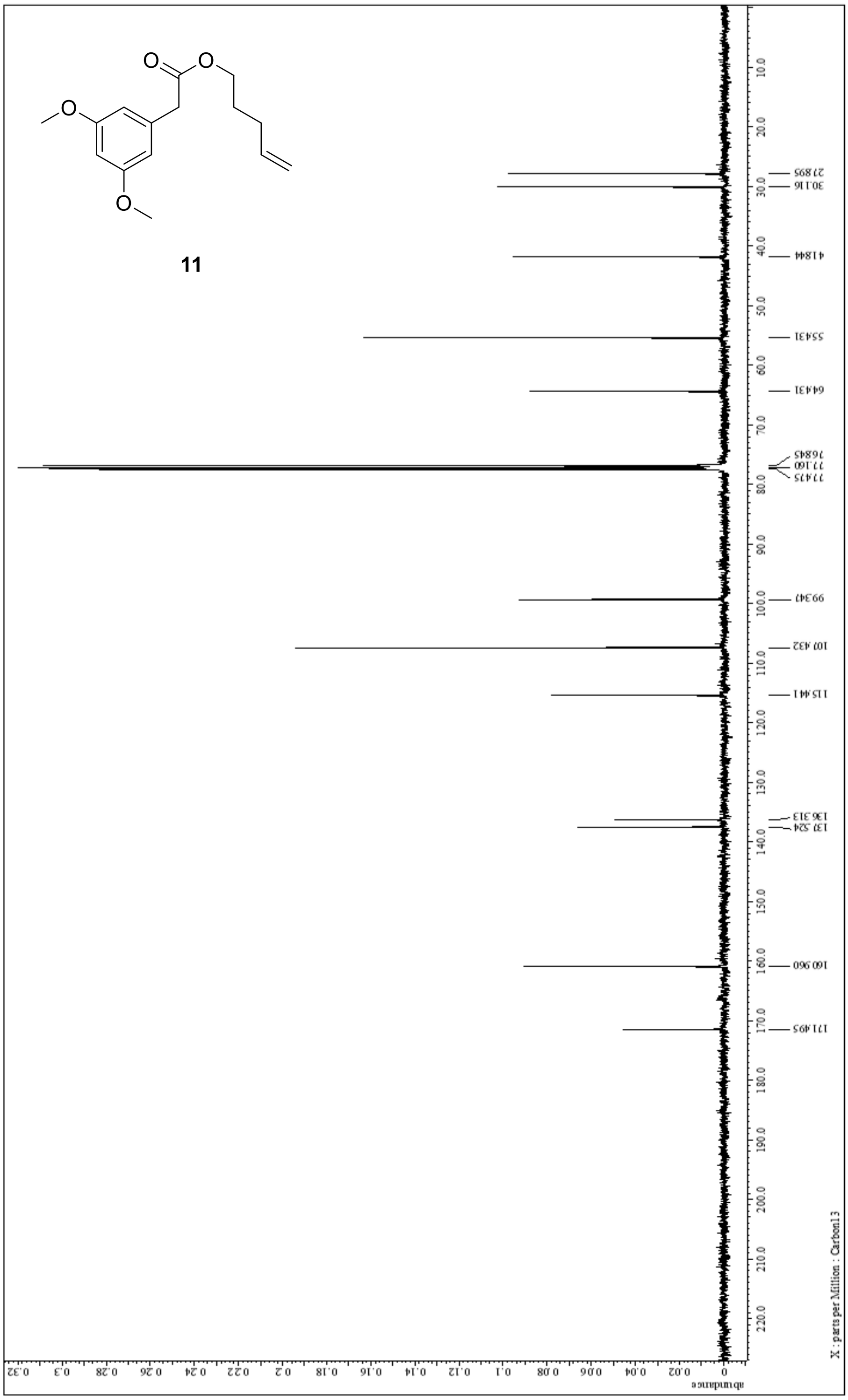
11
21.895
30.116
41.844
55.431
64.431
76.845
77.160
77.415
99.347
107.432
115.441
136.313
137.524
160.960
171.495
X : parts per Million : Carbon13
abundance

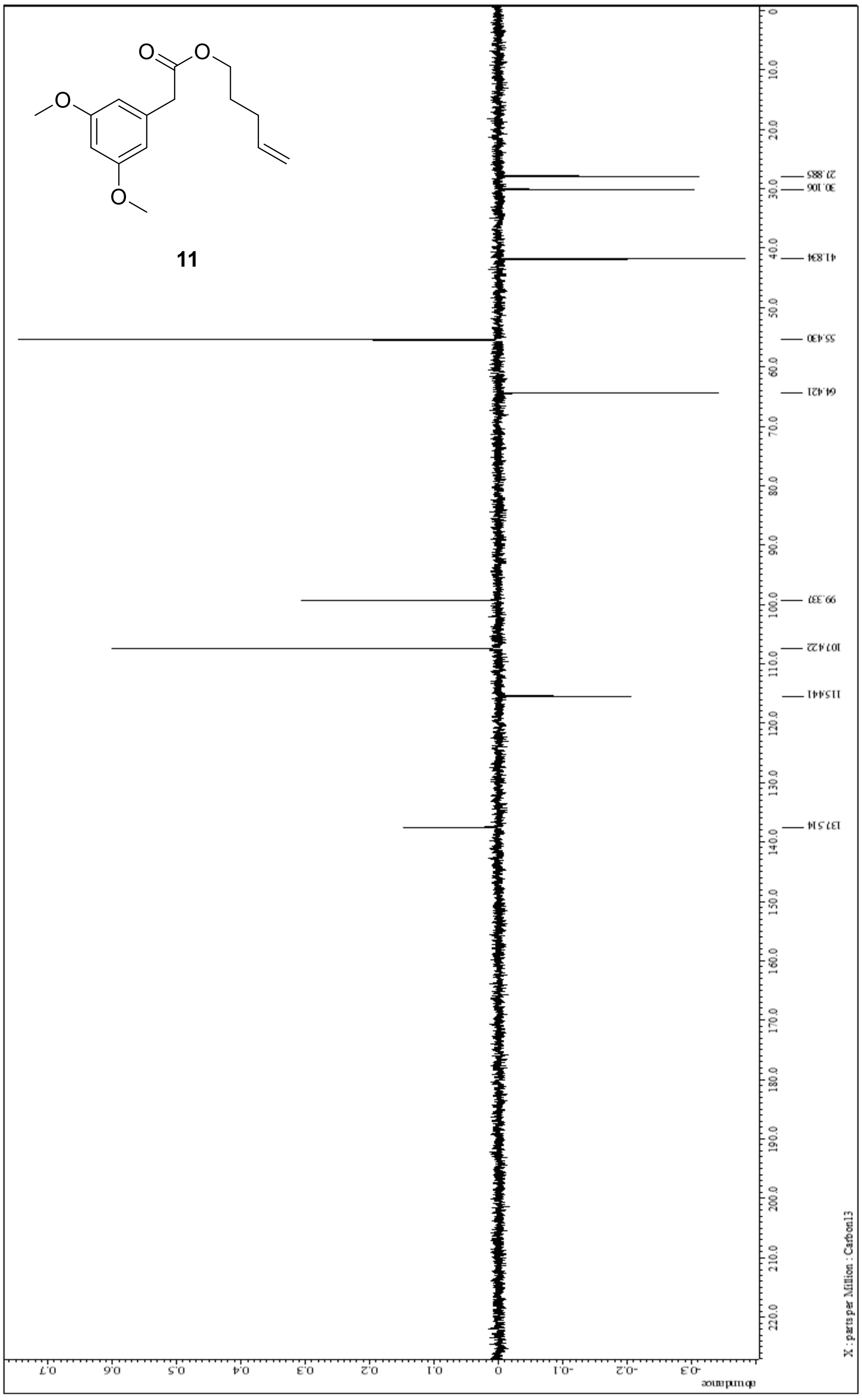
11
27.885
30.106
41.834
55.430
64.421
99.339
107.422
115.441
137.514
X : parts per Million : Carbon13
abundance

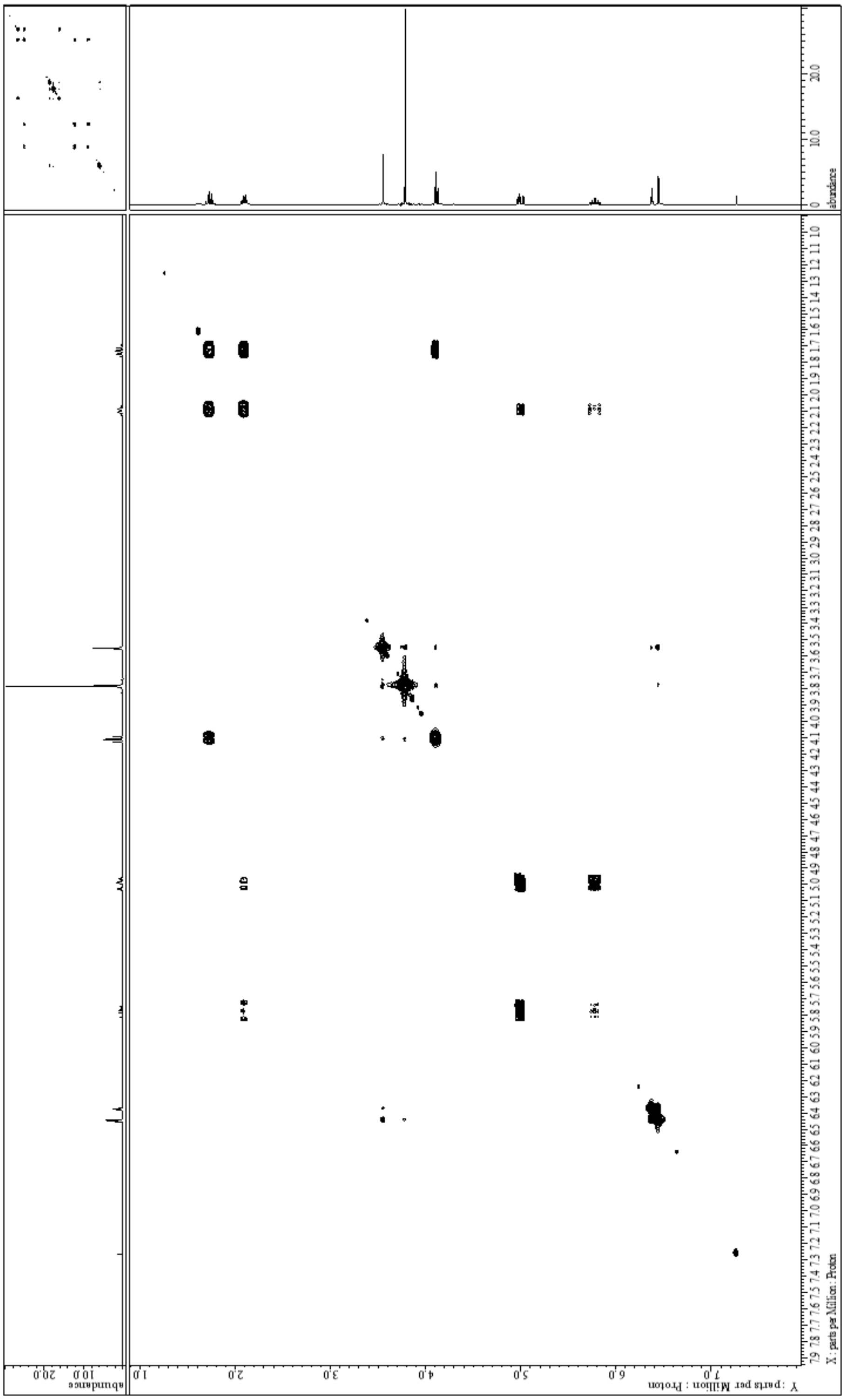
abundance
X : parts per Million : Proton
Y : parts per Million : Proton
abundance

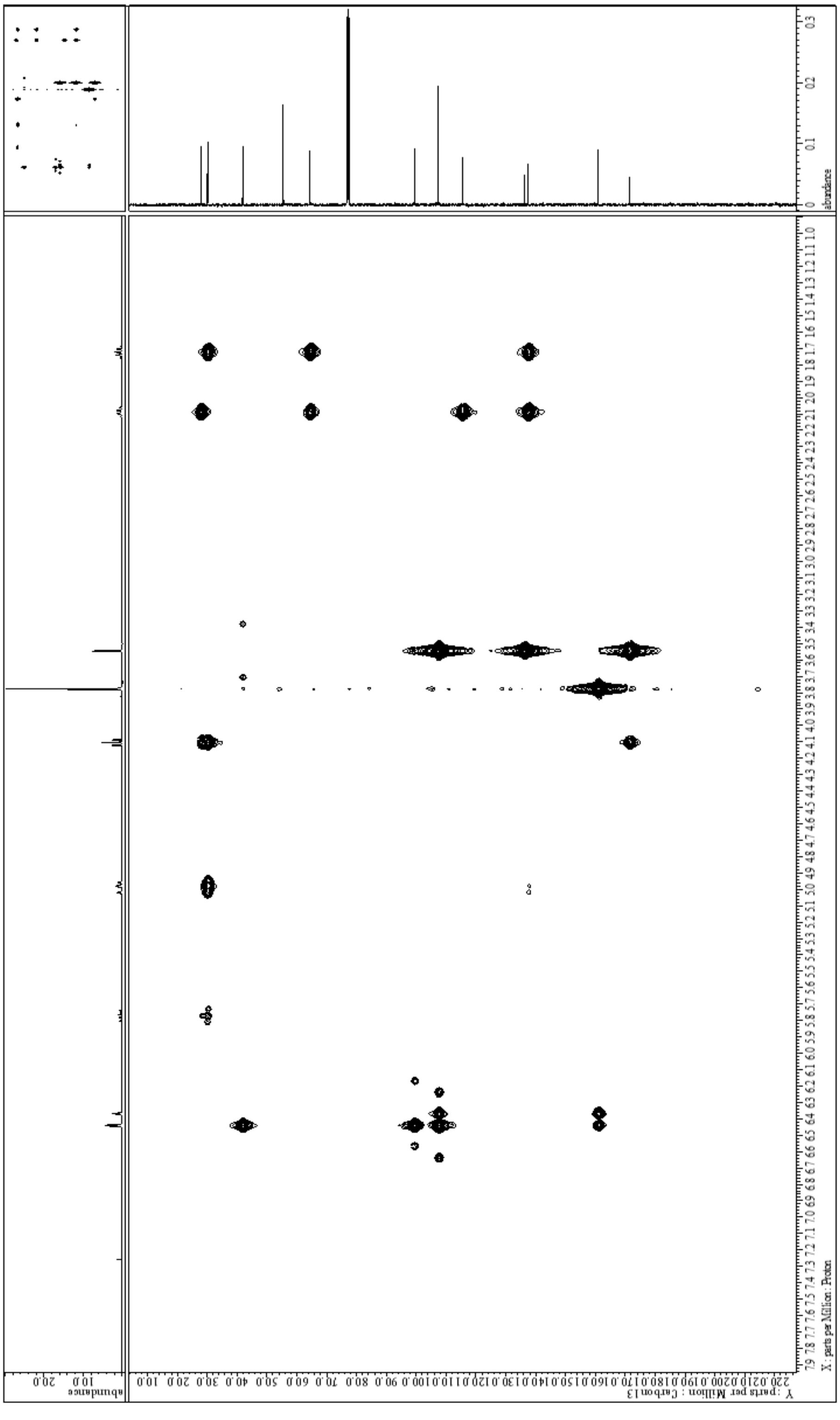
X : parts per Million : Proton
Y : parts per Million : Carbon13
abundance

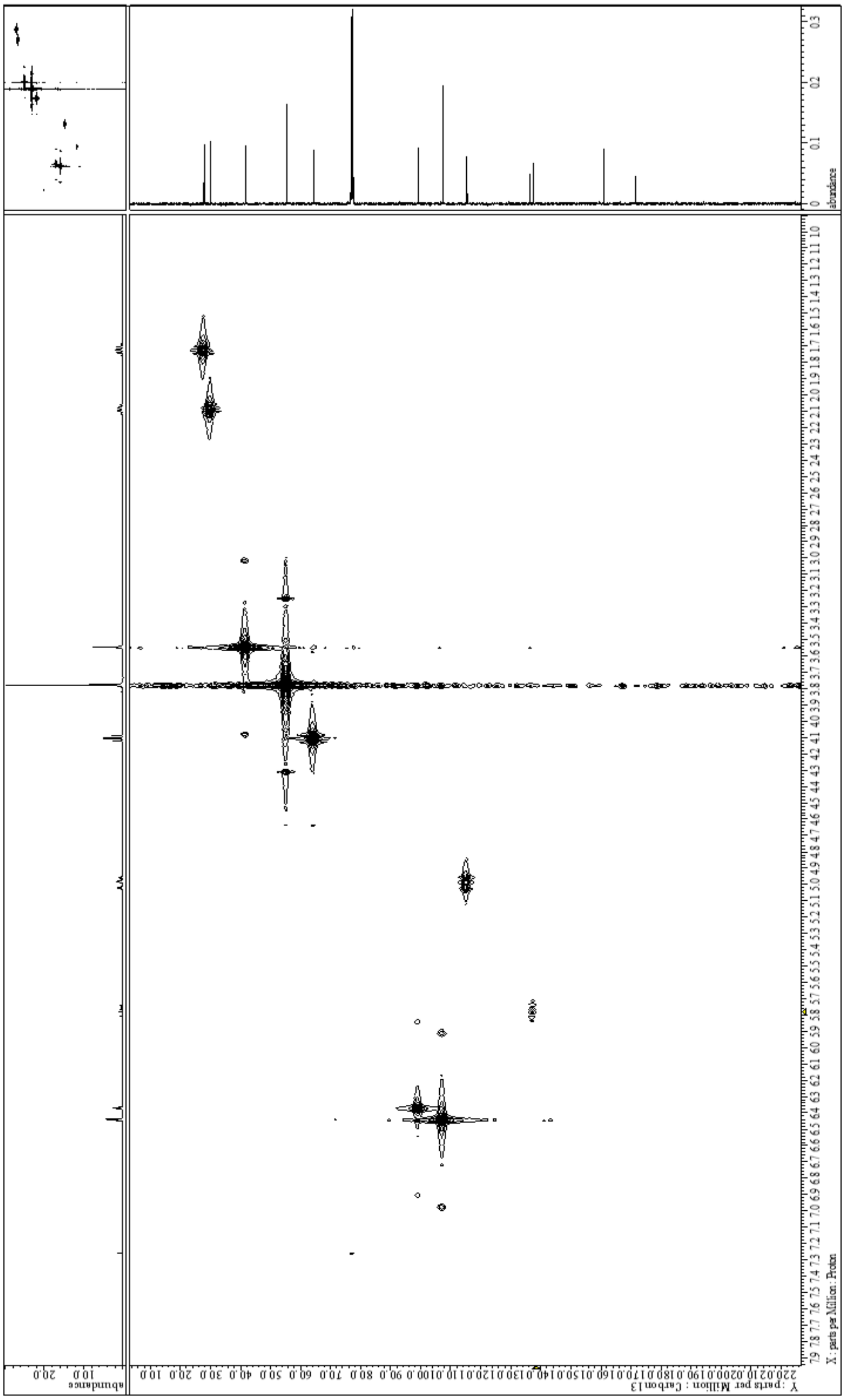

Figure S7. NMR spectrums of pent-4-en-1-yl 2-(3,5-dimethoxyphenyl)acetate (**11**)

Synthetic procedures and characterization data for the compound **12**

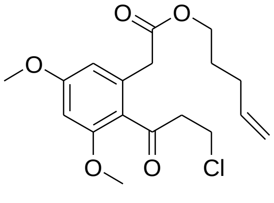

12

pent-4-en-1-yl 2-(2-(3-chloropropanoyl)-3,5-dimethoxyphenyl)acetate (12)

A flame-dried 500 mL round-bottom flask equipped with a magnetic stir bar was charged with anhydrous DCM (156 mL), and 3-chloropropanoyl chloride (**9**) (3.5 mL, 36.9 mmol) was added. After cooling to –78 °C, $SnCl_4$ (1 M in DCM, 37 mL, 36.9 mmol) was added, and the mixture was stirred for 15 min. Compound **11** (7.5 g, 28.4 mmol) was dissolved in DCM (156 mL) and added to the reaction mixture. The reaction temperature was then raised to –20 °C and stirred for 1 h 30 min. After completion of the reaction, the reactant was poured into a separatory funnel filled with ice (150 g) and H_2O (150 mL), and extraction was performed by adding DCM. The obtained organic layer was washed with sat. aq. After washing with $NaHCO_3$ (100 mL) and H_2O (100 mL), the mixture was dried with $MgSO_4$, filtered, and concentrated using a rotary evaporator. The reaction mixture was purified using flash column chromatography (hexane/EtOAc, 9:1) to obtain **12** (7.85 g, 78%).

TLC R_f= 0.5 (hexane/EtOAc, 7:3). ^{1}H NMR (400 MHz, $CDCl_3$) δ 6.41 (d, *J* = 2.3 Hz, 1H, H-6), 6.37 (d, *J* = 2.3 Hz, 1H, H-4), 5.78 (m, 1H, H-4′), 5.02 (m, 2H, H-5′), 4.97 (m, 2H, H-5′), 4.09 (t, *J* = 6.6 Hz, 2H, H-1′), 3.84 (s, 3H, H-5 OMe), 3.82 (s, 3H, H-7 OMe), 3.81 (m, 2H, H-11), 3.67 (s, 2H, H-2), 3.35 (t, *J* = 7.1 Hz, 2H, H-10), 2.10 (m, 2H, H-3′), 1.72 (m, 2H, H-2′); ^{13}C NMR (100 MHz, $CDCl_3$) δ 202.5, 171.5, 162.1, 159.5, 137.6, 135.8, 122.8, 115.4, 108.6, 97.6, 64.5, 55.8, 55.6, 47.1, 39.5, 39.2, 30.1, 27.9; HRESIMS *m/z* 355.1286 $[M + H]^+$ (calcd for $C_{18}H_{24}ClO_5$, 355.1312).

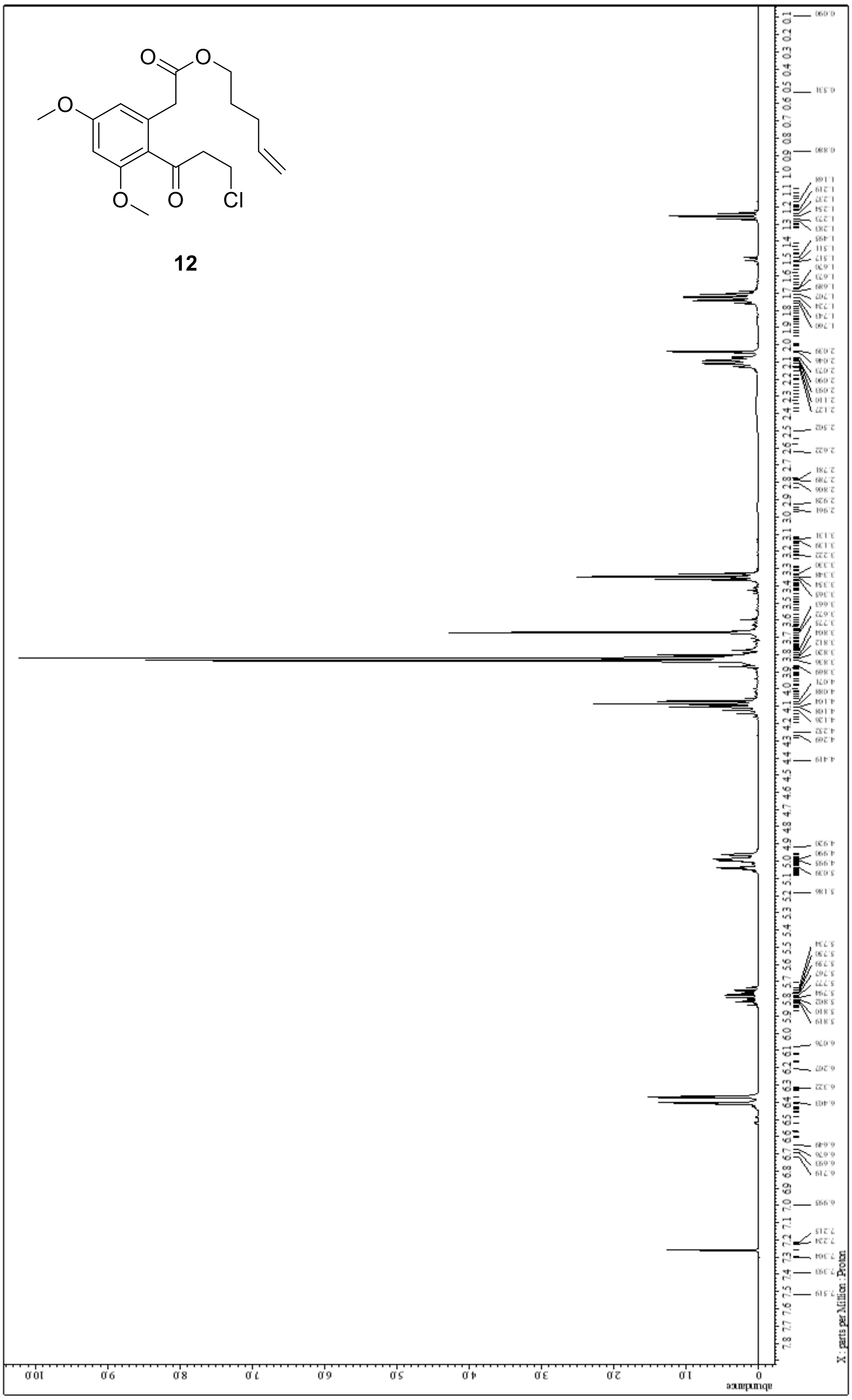
O
O
O
O
O
Cl
12
X : parts per Million : Proton
abundance

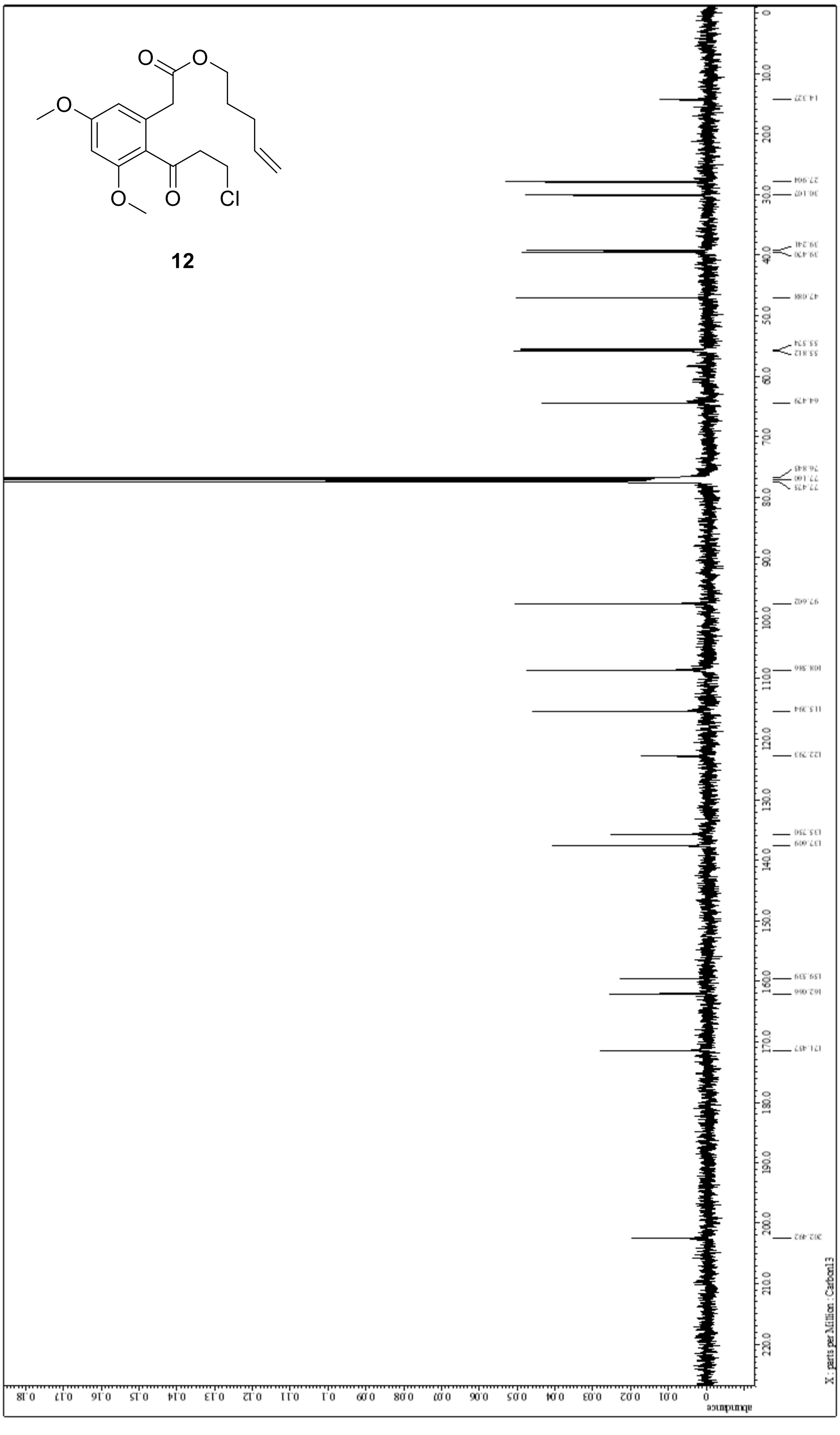

12
X : parts per Million : Carbon13
abundance

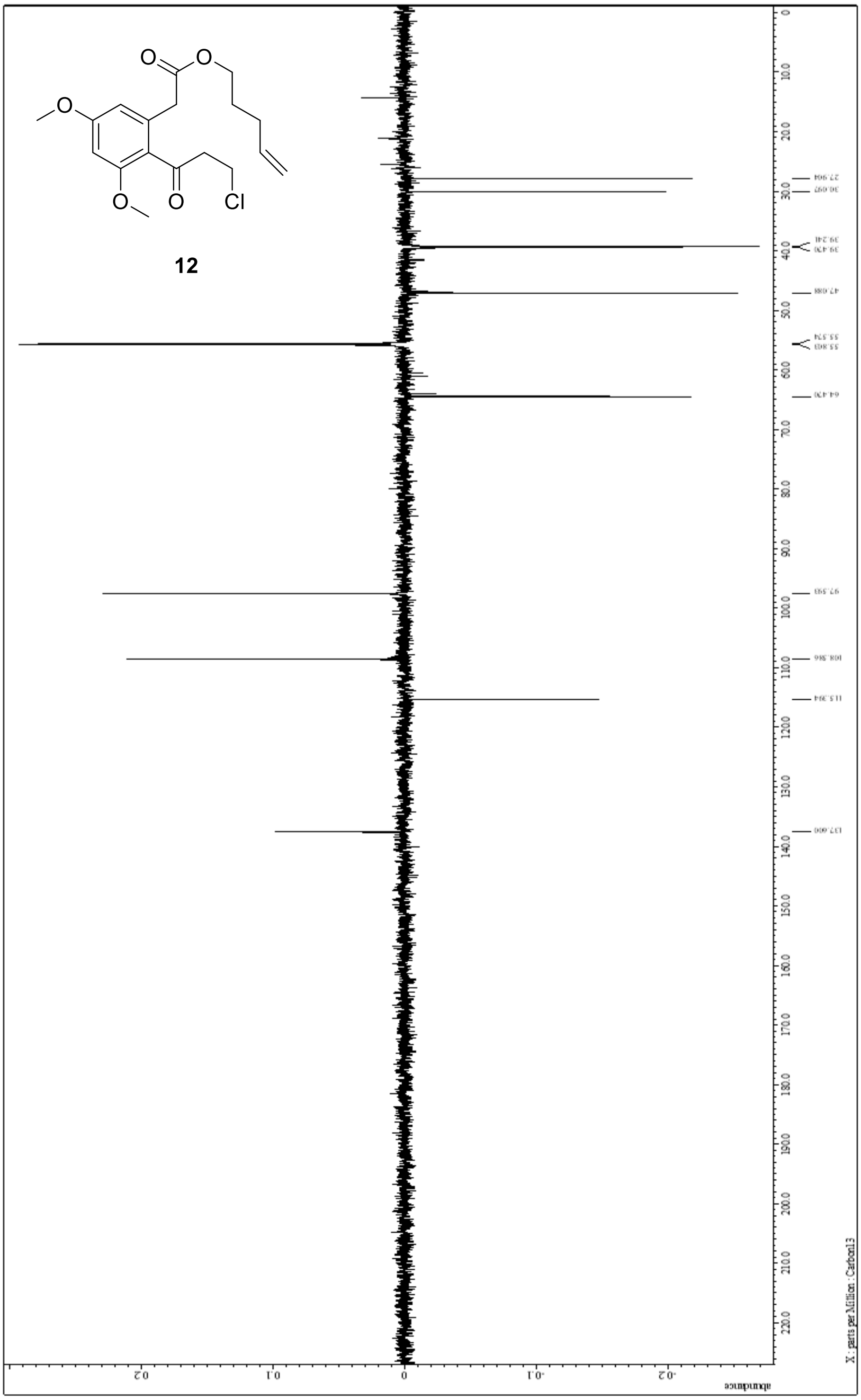
12
O
Cl
27.904
30.097
39.241
39.430
47.088
55.524
55.803
64.430
97.593
108.386
115.394
137.600
X : parts per Million : Carbon13
abundance

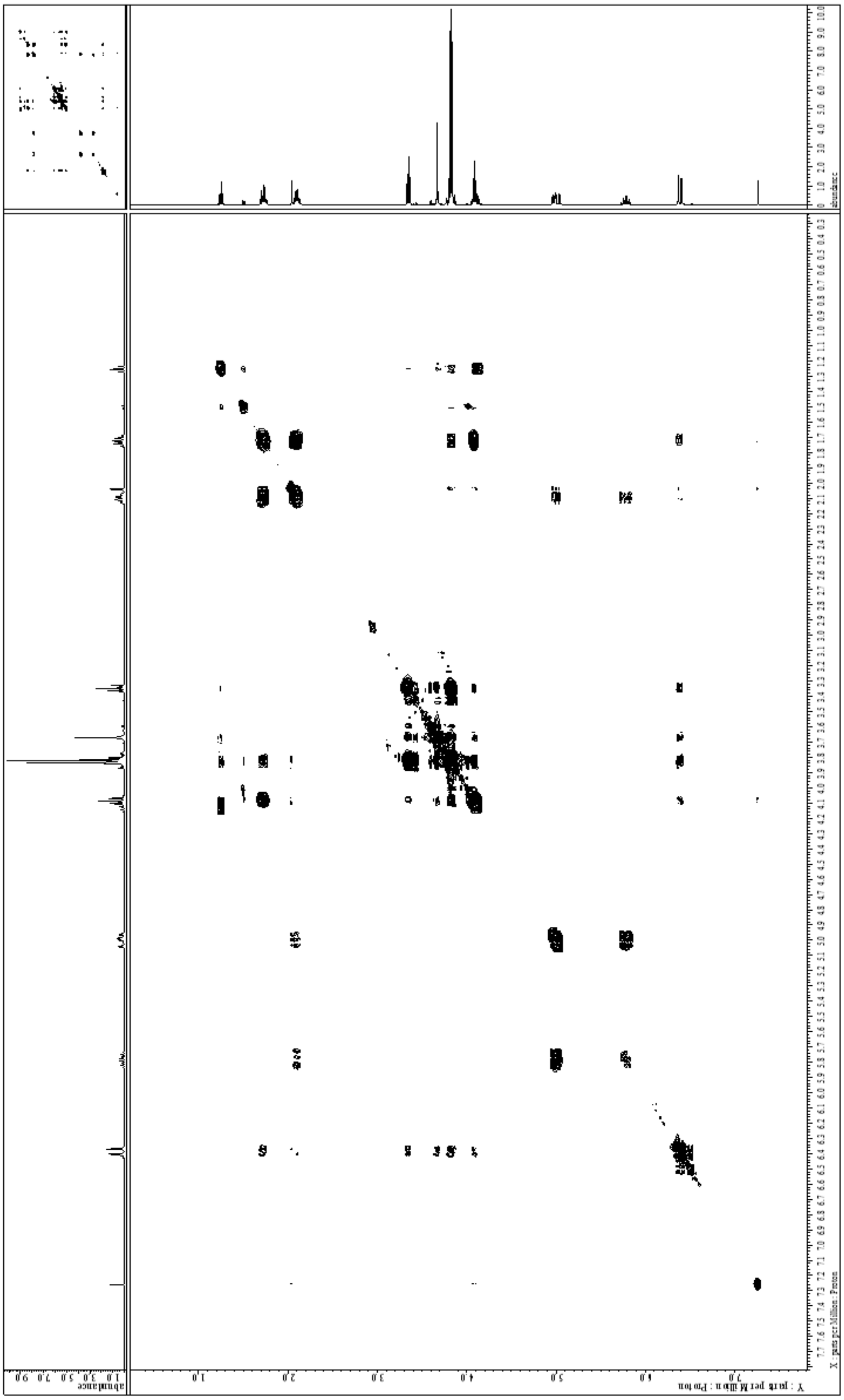
X : parts per Million : Proton
Y : parts per Million : Proton
abundance

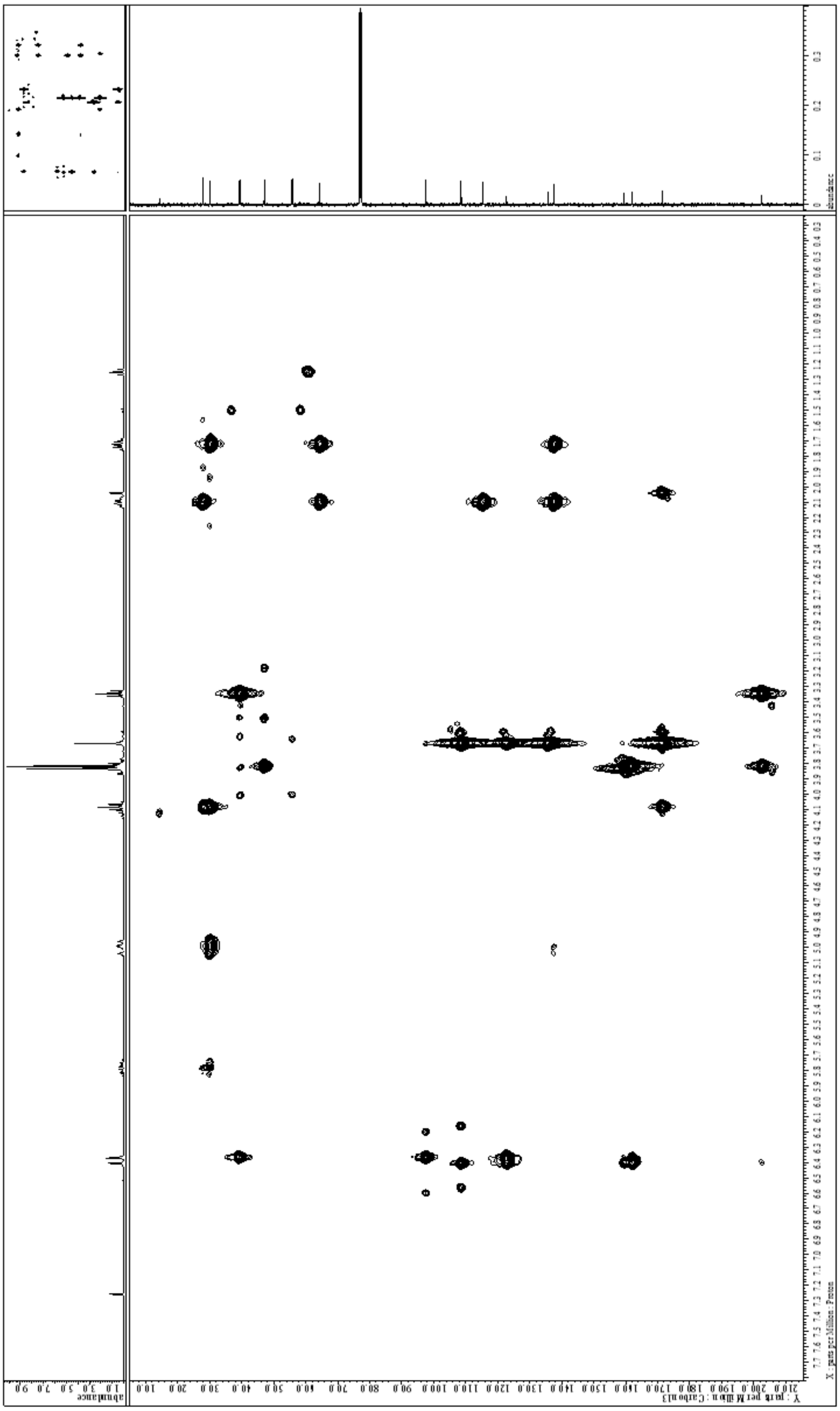

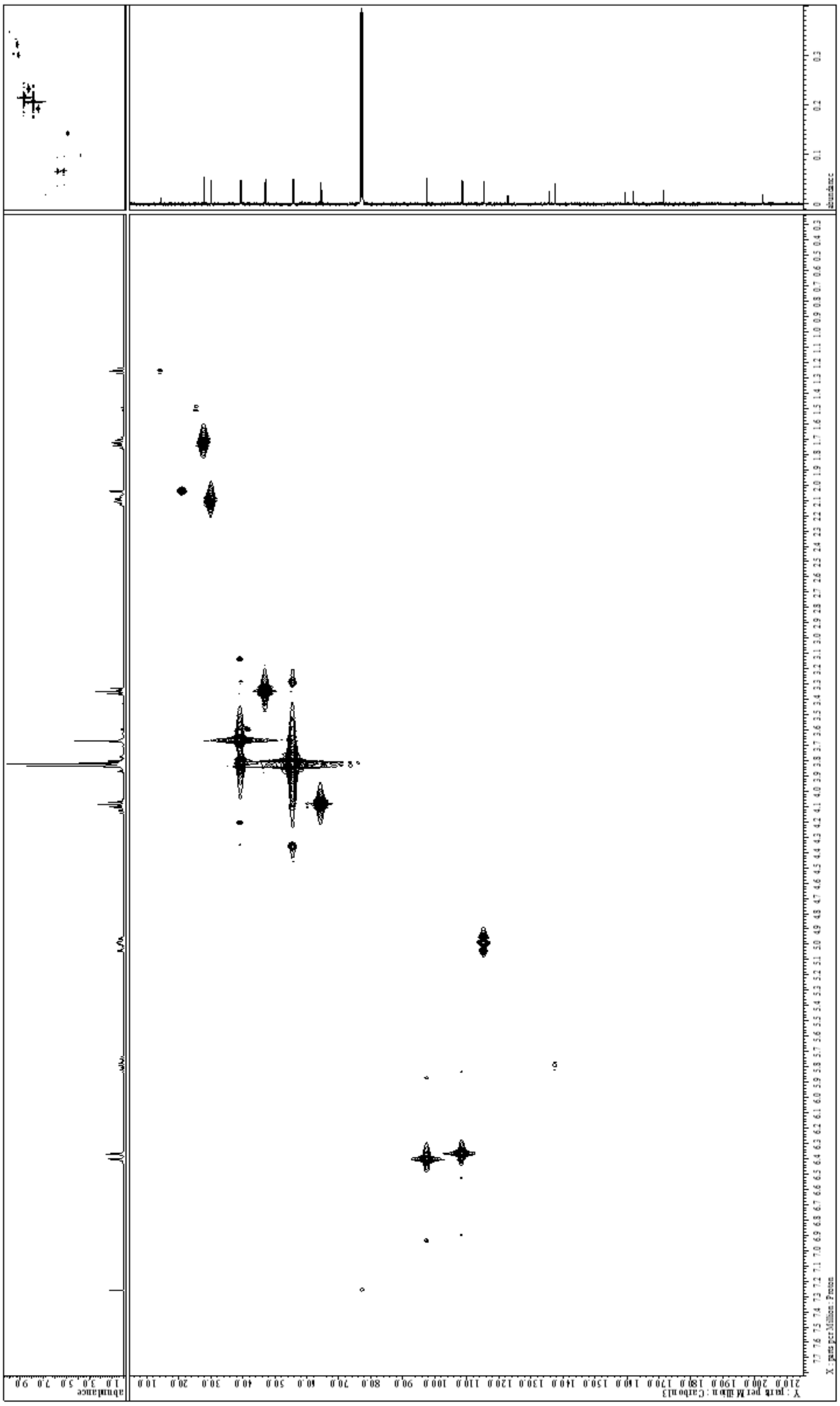

Figure S8. NMR spectrums of pent-4-en-1-yl 2-(2-(3-chloropropanoyl)-3,5-dimethoxyphenyl)acetate (**12**)

Synthetic procedures and characterization data for the compound **13**

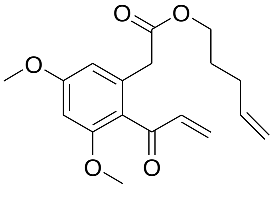

13

pent-4-en-1-yl 2-(2-acryloyl-3,5-dimethoxyphenyl)acetate (13)

A flame-dried 500 mL round-bottom flask equipped with a magnetic stir bar was charged with anhydrous DCM (214 mL), and compound **12** (3.61 g, 10.2 mmol) was added. TEA (2.84 mL, 20.4 mmol) was then added, and the mixture was stirred at room temperature overnight. The reaction mixture, to which sat. aq. NH_4Cl was added, was extracted with DCM (3 × 100 mL) using a separatory funnel. The obtained organic layer was dried over $MgSO_4$, filtered, and concentrated using a rotary evaporator. The reaction mixture was purified using flash column chromatography (hexane/EtOAc, 8:2) to obtain **13** (2.85 g, 88%).

TLC R_f= 0.78 (hexane/EtOAc, 6:4). ^{1}H NMR (400 MHz, $CDCl_3$) δ 6.69 (dd, *J* = 17.4, 10.5 Hz, 1H, H-10), 6.42 (d, *J* = 2.3 Hz, 1H, H-4), 6.41 (d, *J* = 2.3 Hz, 1H, H-6), 6.09 (dd, *J* = 17.4, 1.4 Hz, 2H, H-11), 5.79 (m, 2H, H-11), 5.76 (m, 1H, H-4′), 4.97 (m, 2H, H-5′), 4.05 (t, *J* = 6.6 Hz, 2H, H-1′), 3.82 (s, 3H, H-5 OMe), 3.77 (s, 3H, H-7 OMe), 3.61 (s, 2H, H-2), 2.07 (m, 2H, H-3′), 1.69 (m, 2H, H-2′); ^{13}C NMR (100 MHz, $CDCl_3$) δ 195.8, 171.2, 161.8, 159.3, 138.2, 137.6, 135.5, 128.5, 122.3, 115.3, 107.9, 97.7, 64.4, 55.8, 55.5, 38.9, 30.1, 27.8; HRESIMS *m/z* 319.1517 $[M + H]^+$ (calcd for $C_{18}H_{23}O_5$, 319.1545).

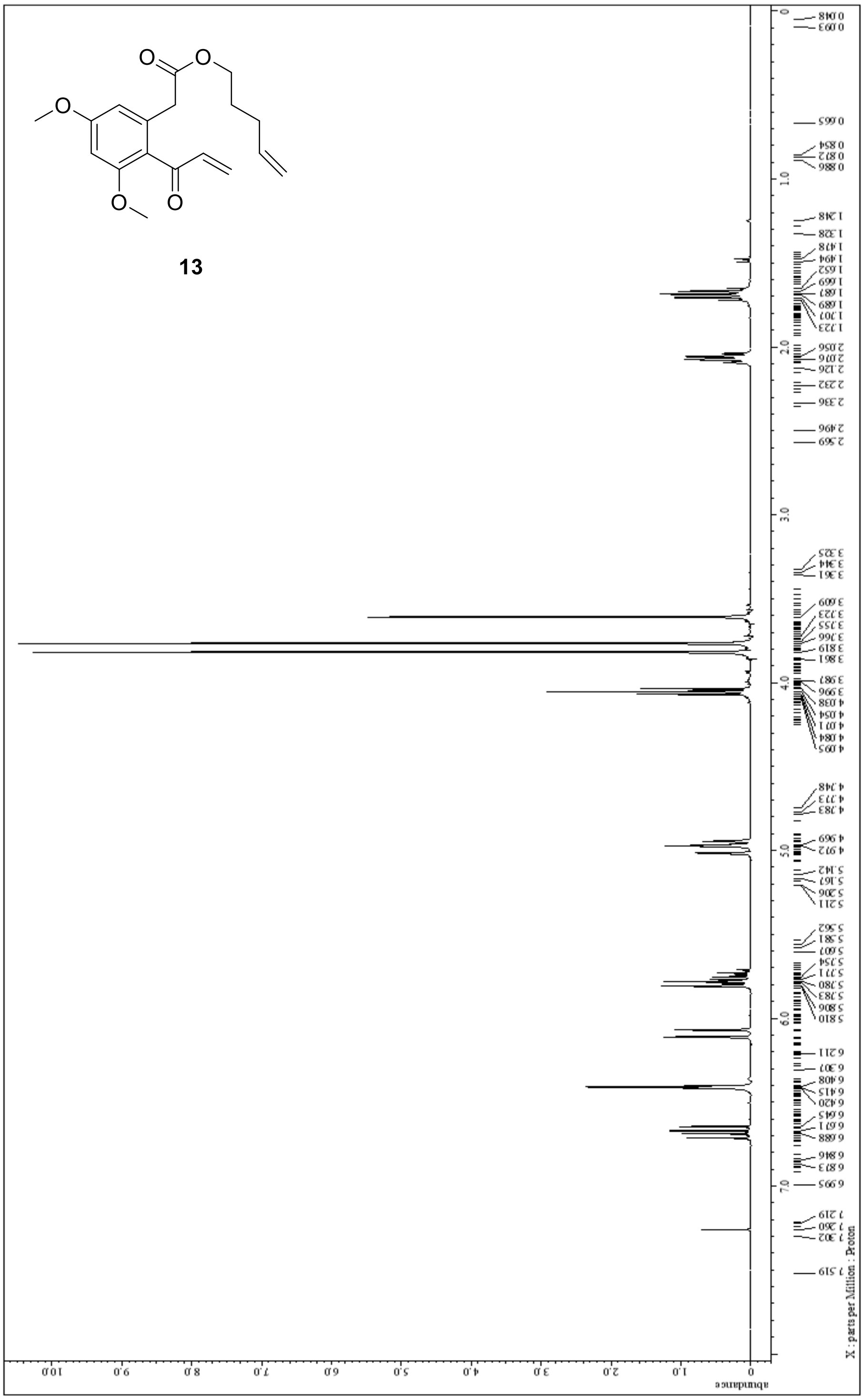
13
X : parts per Million : Proton
abundance

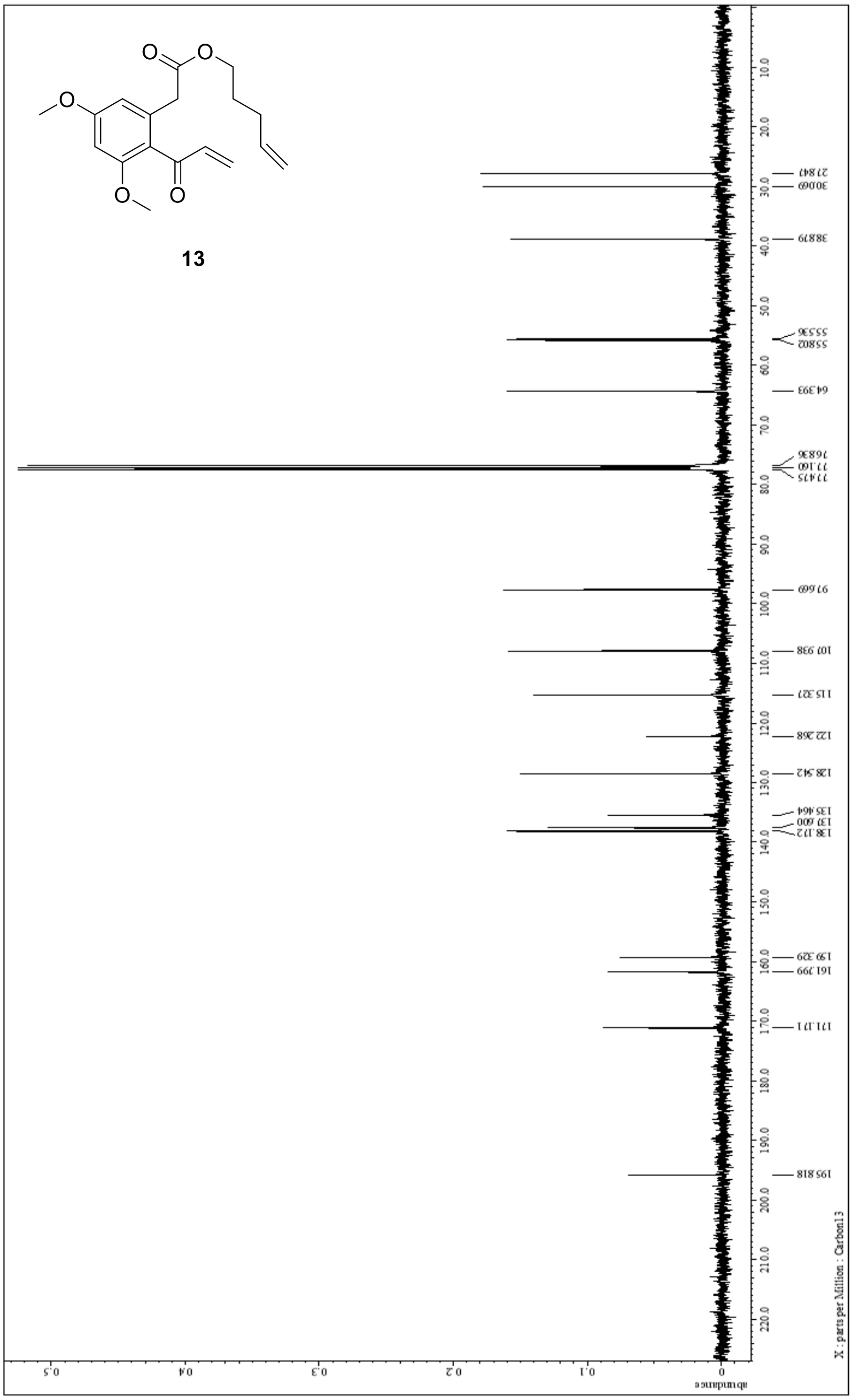
13
21.847
30.069
38.879
55.536
55.802
64.393
76.836
77.160
77.415
97.669
107.938
115.321
122.268
128.542
135.464
137.600
138.172
159.329
161.799
171.171
195.818
X : parts per Million : Carbon13
abundance

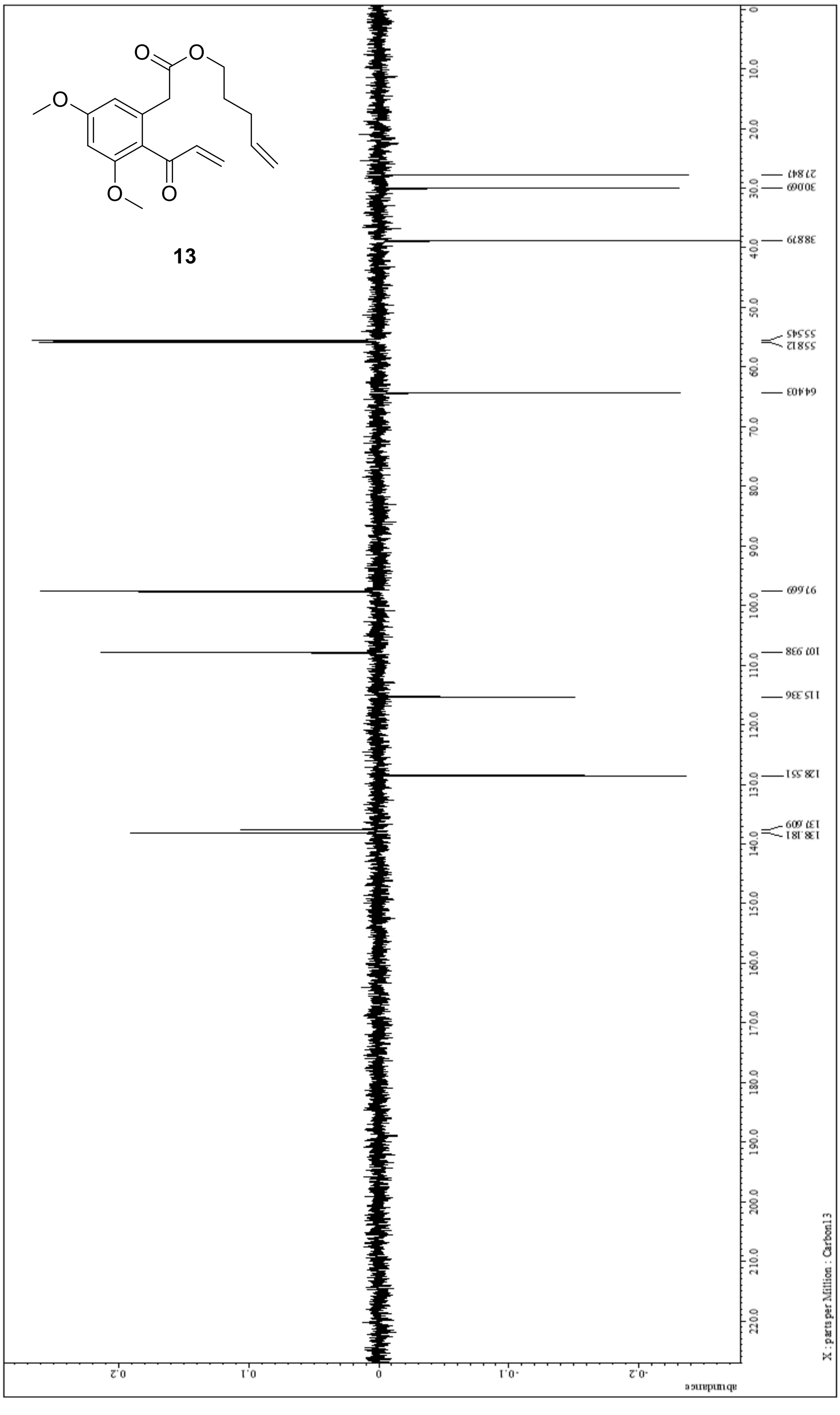

13
21.841
30.069
38.879
55.545
55.812
64.403
97.669
107.938
115.336
128.551
137.609
138.181
X : parts per Million : Carbon13
abundance

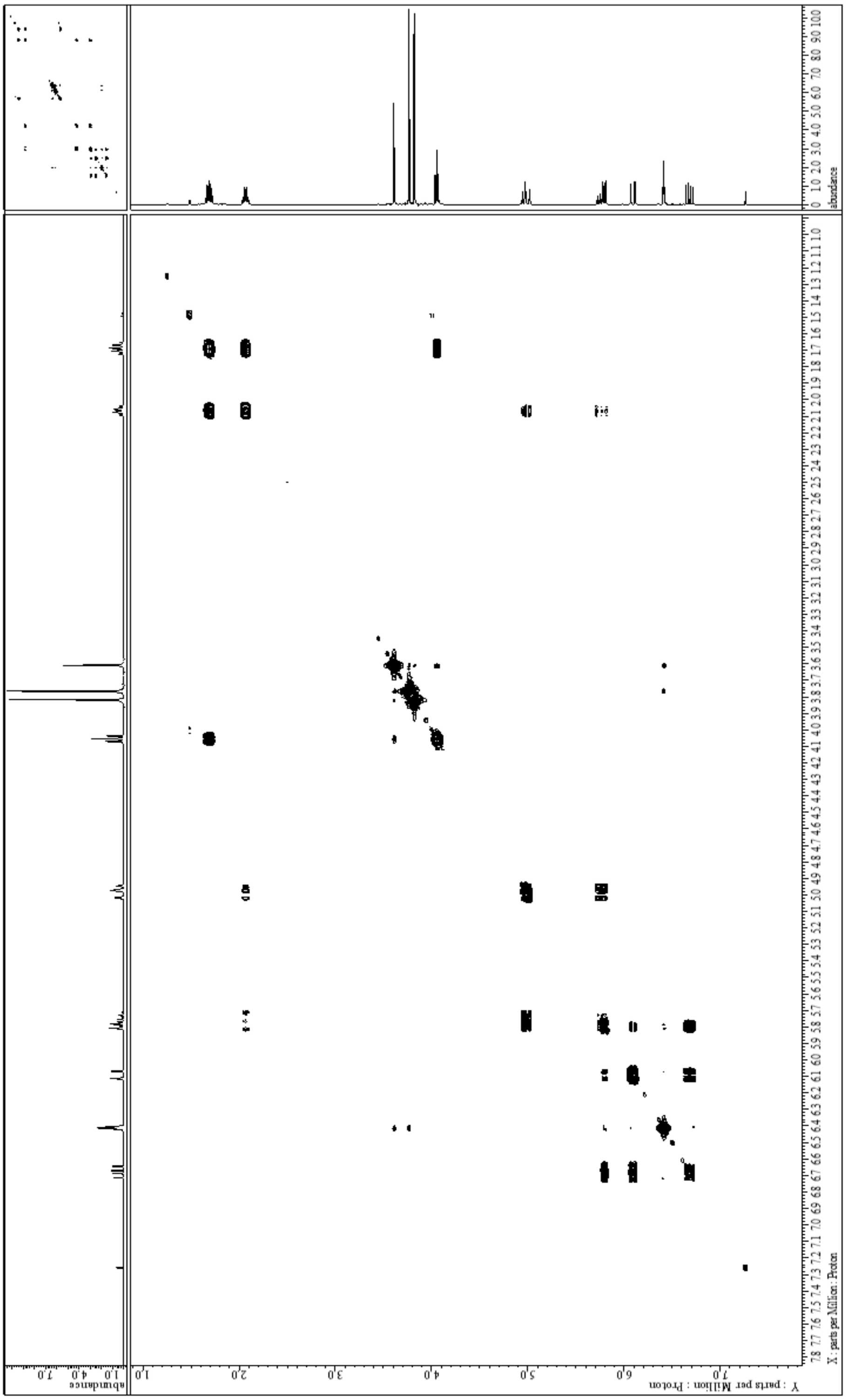
X : parts per Million : Proton
Y : parts per Million : Proton
abundance

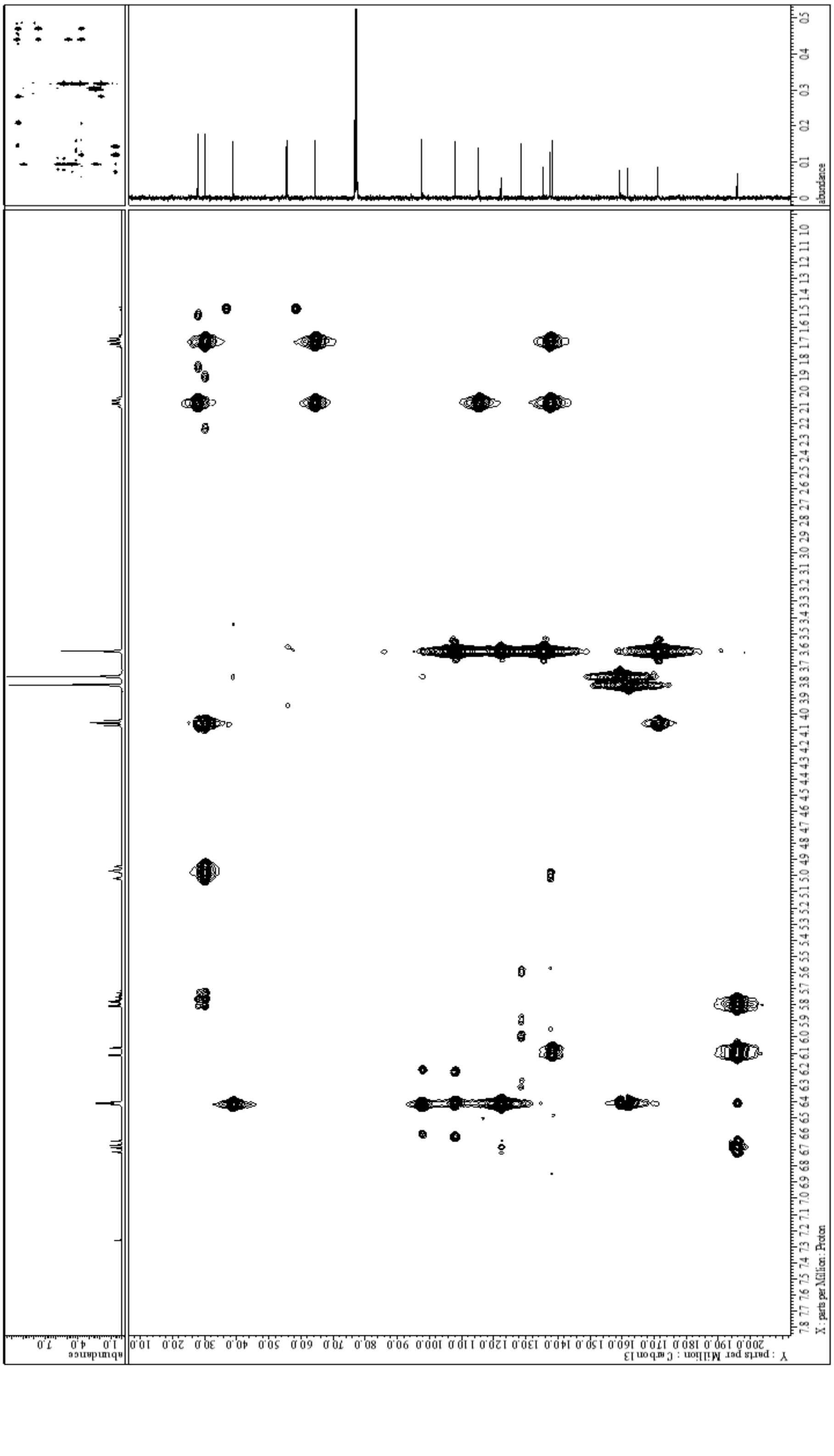

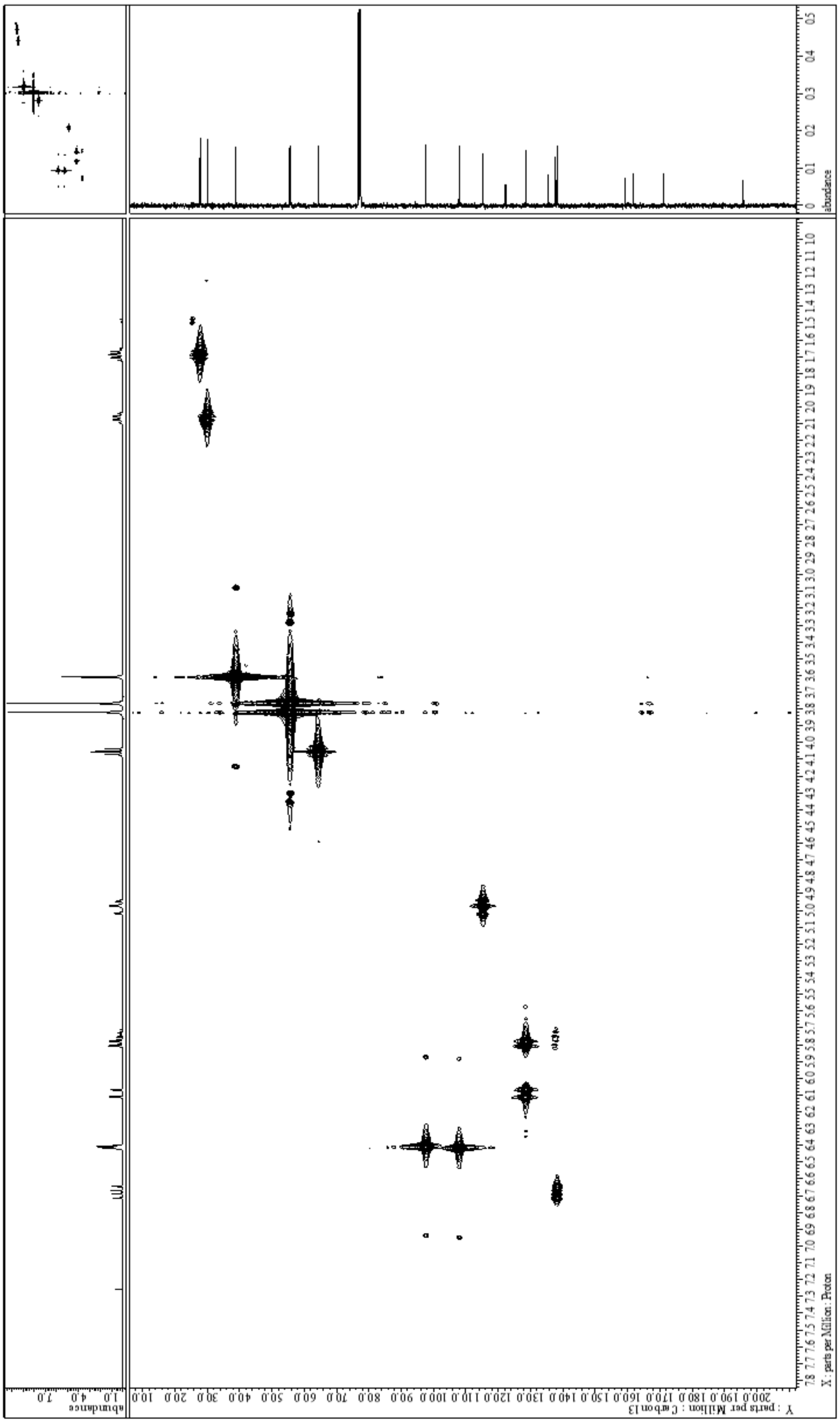

Figure S9. NMR spectrums of pent-4-en-1-yl 2-(2-acryloyl-3,5-dimethoxyphenyl)acetate (**13**)

Synthetic procedures and characterization data for the compound **14**

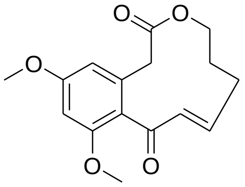

14

(*E*)-10,12-dimethoxy-5,6-dihydrobenzo[*d*][1]oxacycloundecine-2,9(1*H*,4*H*)-dione (14)

Anhydrous toluene (1084 mL) was charged into a flame-dried 2000 mL round-bottom flask equipped with a magnetic stir bar, and compound **13** (1.38 g, 4.33 mmol) was added. Argon gas was bubbled through the mixture for 2h. After bubbling, Grubbs II (735 mg, 20 mol%) catalyst dissolved in toluene (40 mL) was added and stirred at 80 °C for 1 h. The reaction mixture was concentrated using a rotary evaporator and purified using flash column chromatography (hexane/EtOAc, 8:2) to obtain a separated mixture of **14** (502 mg, 40%) and **15** (163 mg, 13%). (40%, *E*-isomer)

TLC R_f= 0.44 (hexane/EtOAc, 6:4). ^{1}H NMR (400 MHz, $CDCl_3$) δ 6.43 (s, 1H, H-4; 1H, H-6), 6.38 (m, 1H, H-11), 6.25 (td, *J* = 16.0, 1.4 Hz, 1H, H-10), 4.29 (m, 2H, H-14), 3.84 (s, 3H, H-5 OMe), 3.76 (s, 3H, H-7 OMe), 3.36 (s, 2H, H-2), 2.45 (m, 2H, H-12), 2.36 (m, 2H, H-12), 2.02 (m, 2H, H-13), 1.78 (m, 2H, H-13); ^{13}C NMR (100 MHz, $CDCl_3$) δ 197.9, 171.1, 161.3, 157.8, 155.8, 133.2, 132.5, 122.9, 108.4, 97.9, 65.1, 56.1, 55.6, 40.3, 31.1, 26.9; HRESIMS *m/z* 291.1132 [M + H]$^+$ (calcd for $C_{16}H_{19}O_5$, 291.1232).

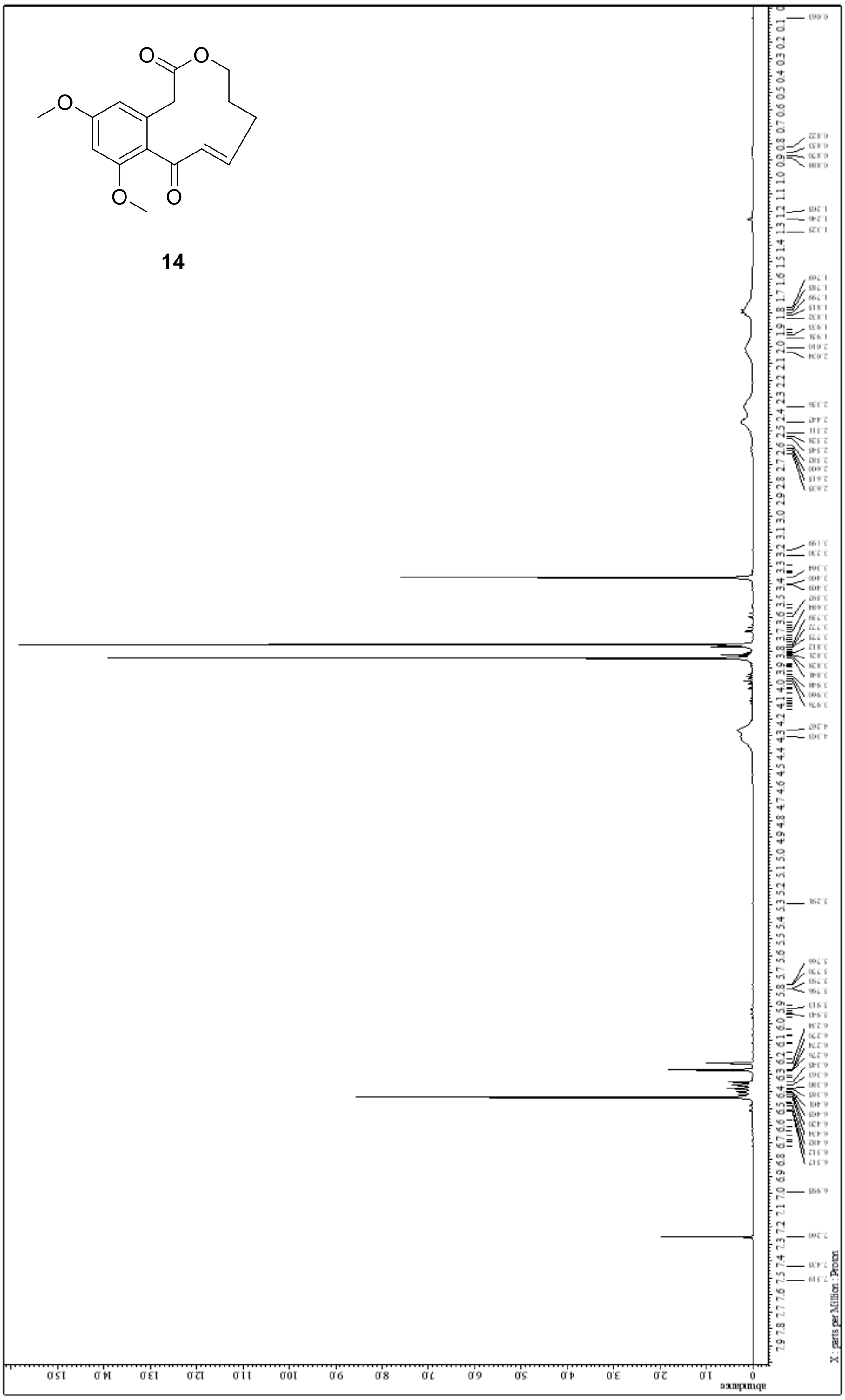
14
X : parts per Million : Proton
abundance

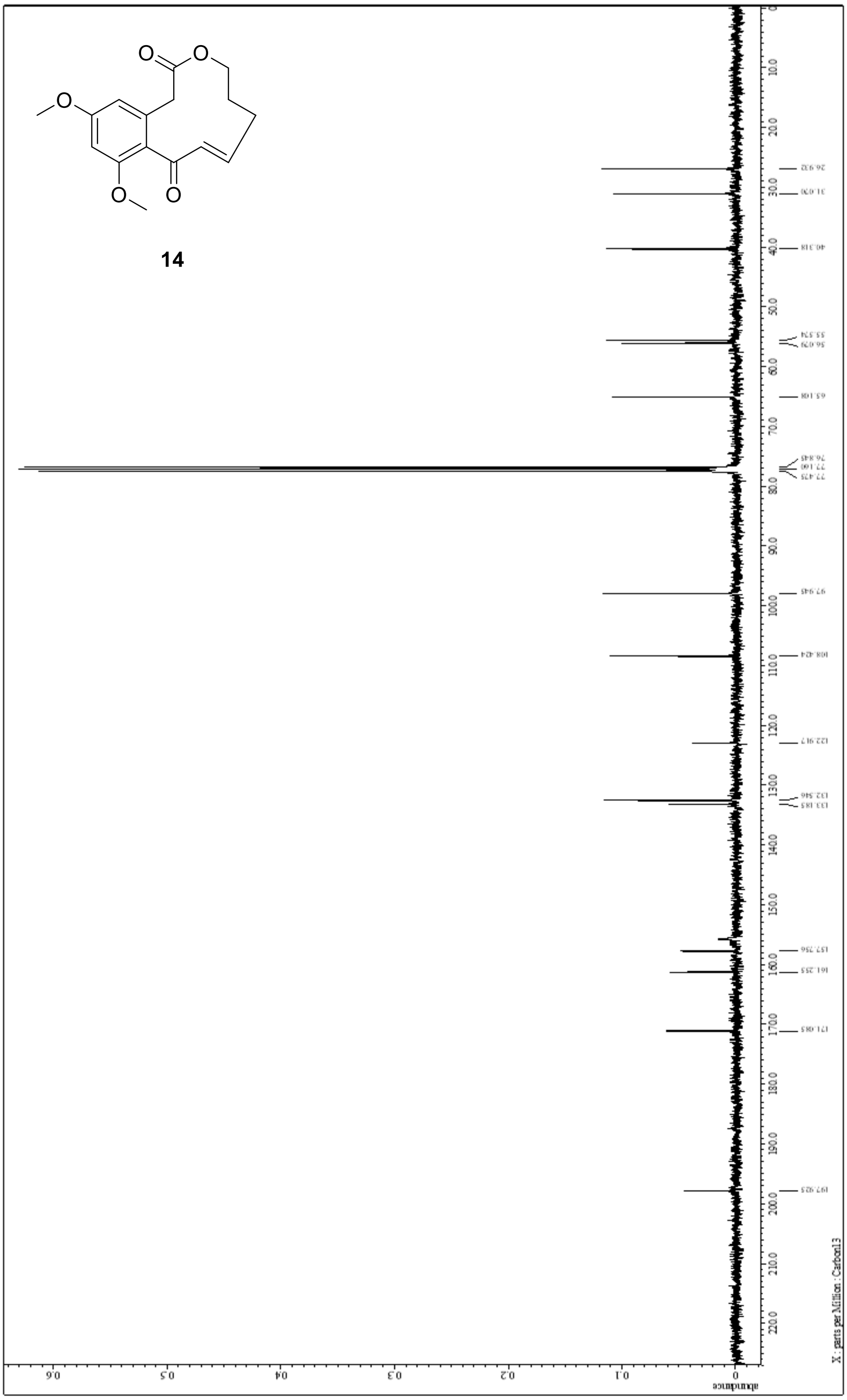
14

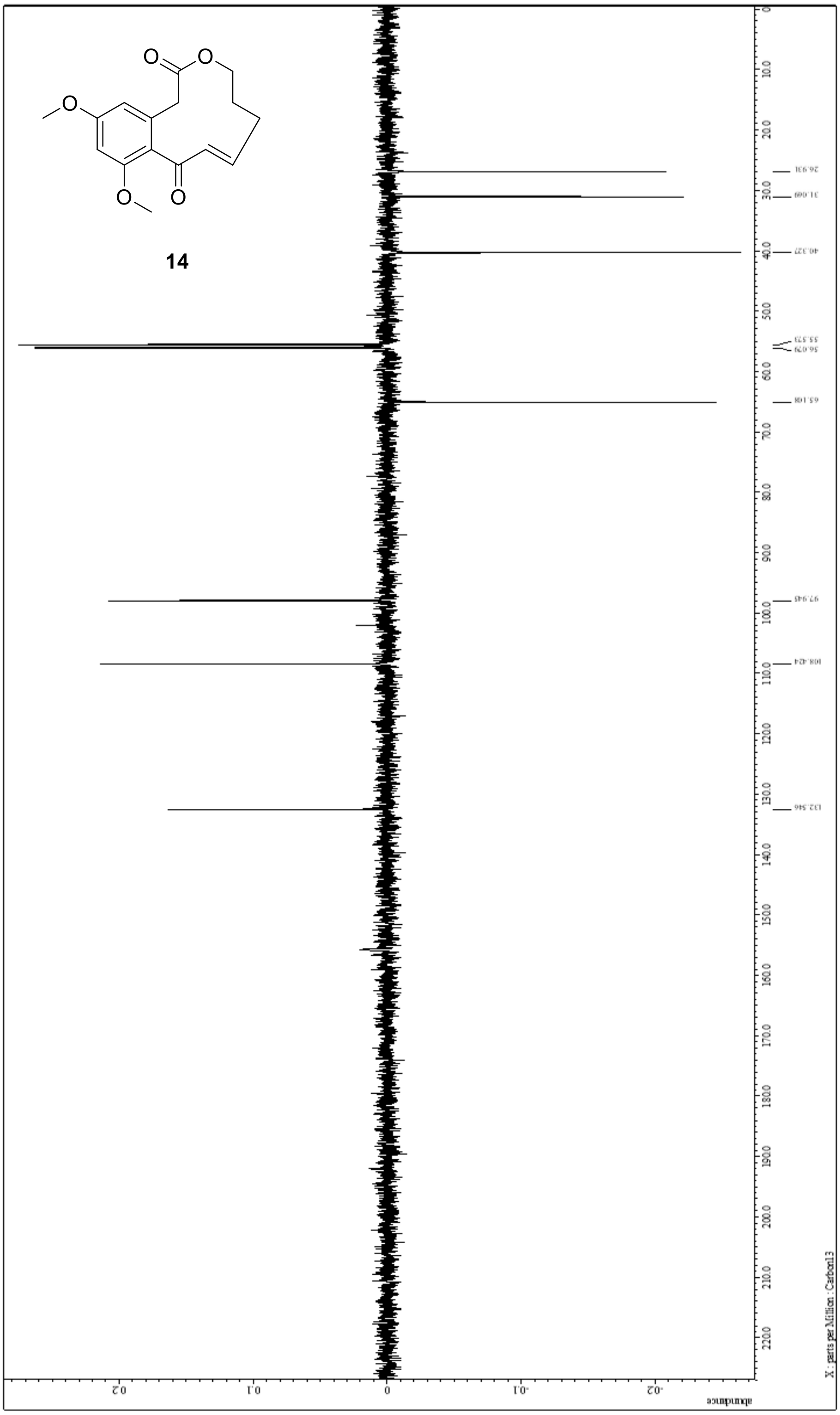

14
26.931
31.669
40.327
55.573
56.029
65.108
97.945
108.424
132.546
X : parts per Million : Carbon13
abundance

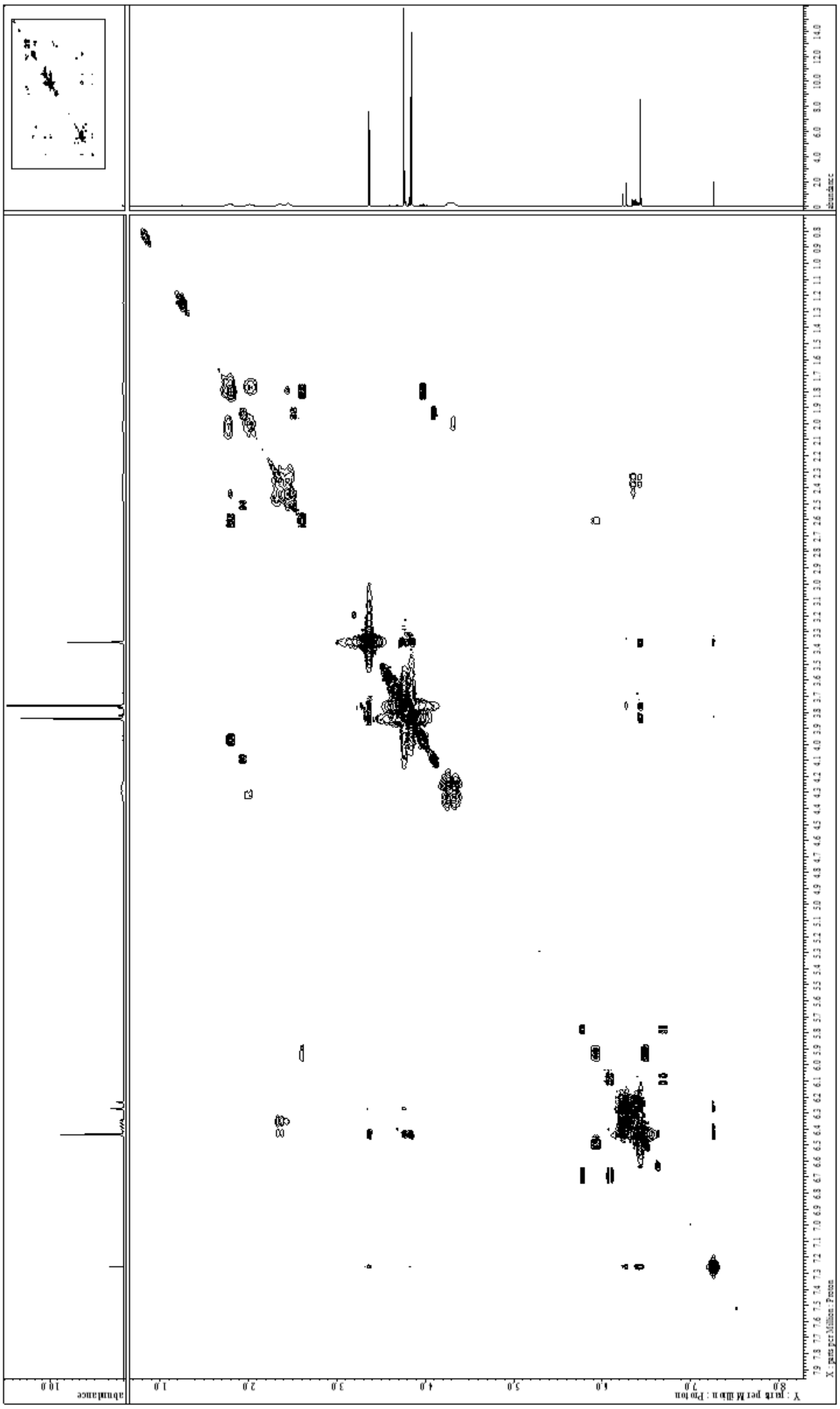

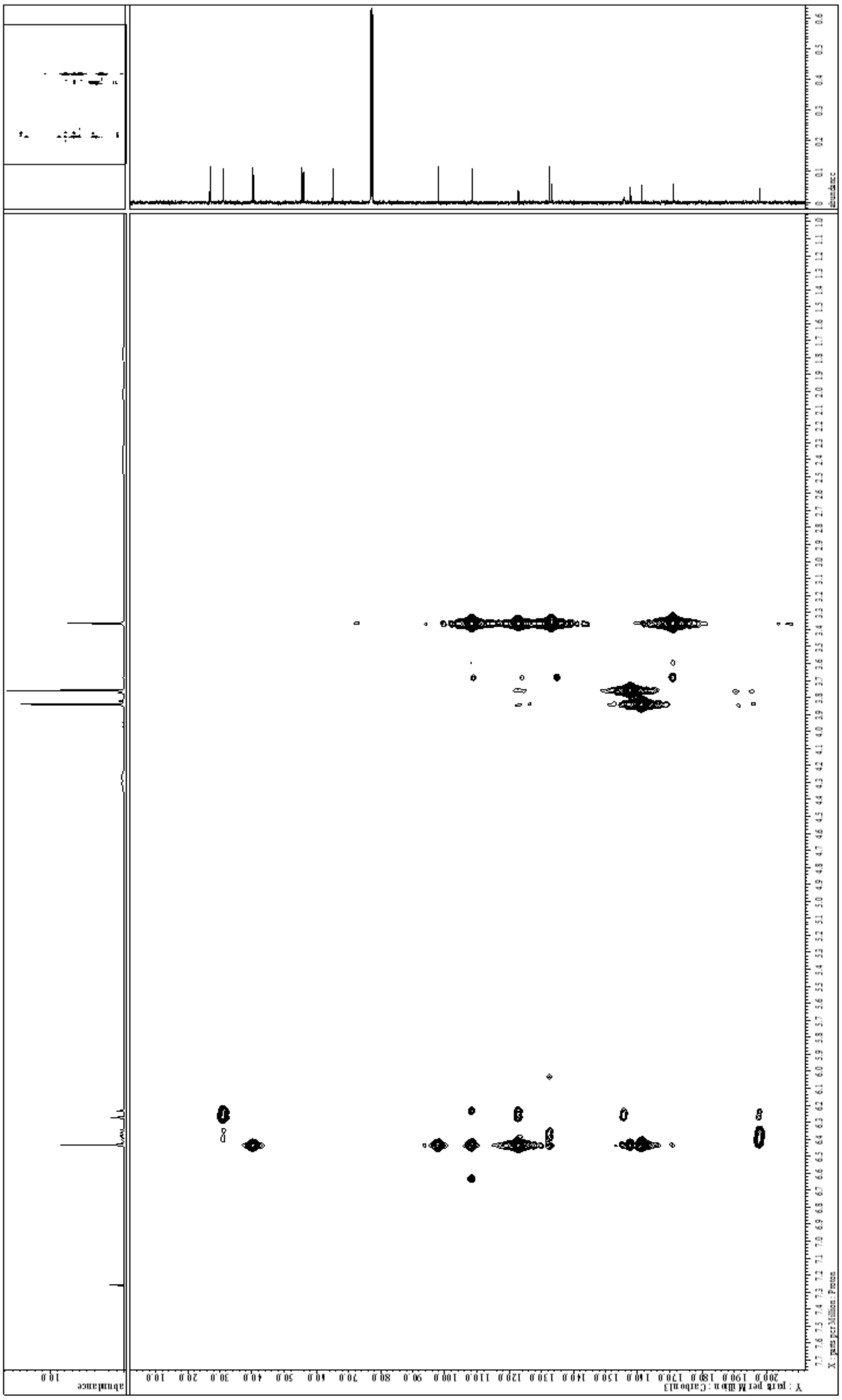

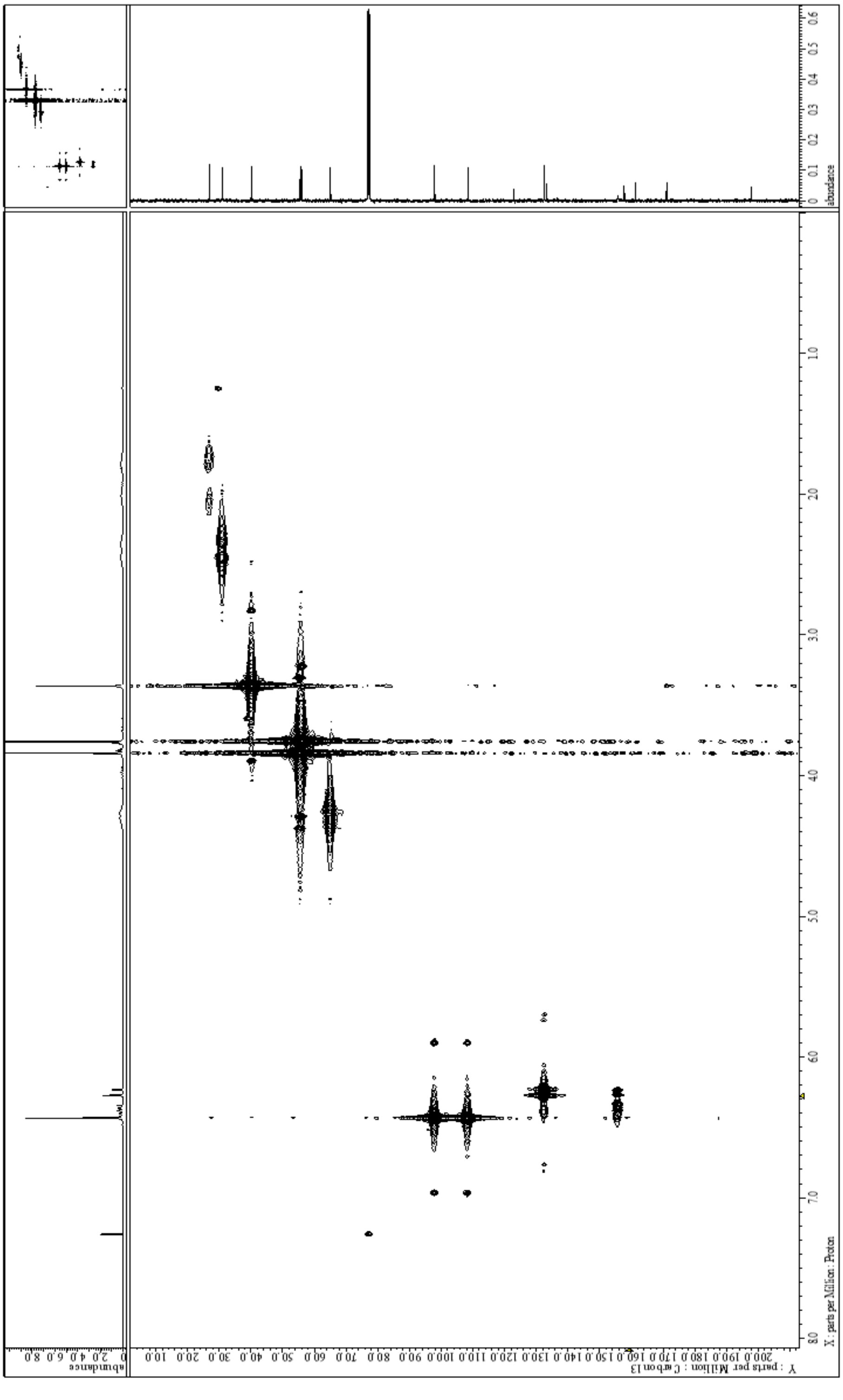

Figure S10. NMR spectrums of (*E*)-10,12-dimethoxy-5,6-dihydrobenzo[*d*][1]oxacycloundecine-2,9(1*H*,4*H*)-dione (**14**)

Synthetic procedures and characterization data for the compound **15**

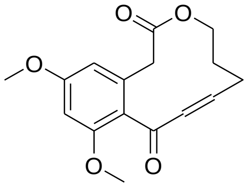

15

10,12-dimethoxy-5,6-dihydrobenzo[*d*][1]oxacycloundecine-2,9(1*H*,4*H*)-dione (15)

Anhydrous toluene (1084 mL) was charged into a flame-dried 2000 mL round-bottom flask equipped with a magnetic stir bar, and compound **13** (1.38 g, 4.33 mmol) was added. Argon gas was bubbled through the mixture for 2h. After bubbling, Grubbs II (735 mg, 20 mol%) catalyst dissolved in toluene (40 mL) was added and stirred at 80 °C for 1 h. The reaction mixture was concentrated using a rotary evaporator and purified using flash column chromatography (hexane/EtOAc, 8:2) to obtain a separated mixture of **14** (502 mg, 40%) and **15** (163 mg, 13%). (13%, *Z*-isomer);

TLC R_f= 0.58 (hexane/EtOAc, 6:4). ^{1}H NMR (400 MHz, $CDCl_3$) δ 6.49 (s, 1H, H-4; 1H, H-6), 6.38 (m, 1H, H-11), 5.93 (m, 1H, H-10), 4.29 (m, 2H, H-14), 3.84 (s, 3H, H-5 OMe), 3.76 (s, 3H, H-7 OMe), 3.36 (s, 2H, H-2), 2.45 (m, 2H, H-12), 2.36 (m, 2H, H-12), 2.02 (m, 2H, H-13), 1.80 (m, 2H, H-13); ^{13}C NMR (100 MHz, $CDCl_3$) δ 197.9, 171.1, 161.3, 157.8, 140.4, 133.2, 132.5, 122.9, 108.4, 97.9, 65.1, 56.1, 55.6, 40.3, 31.1, 26.9; HRESIMS *m/z* 291.1132 $[M + H]^+$ (calcd for $C_{16}H_{19}O_5$, 291.1232).

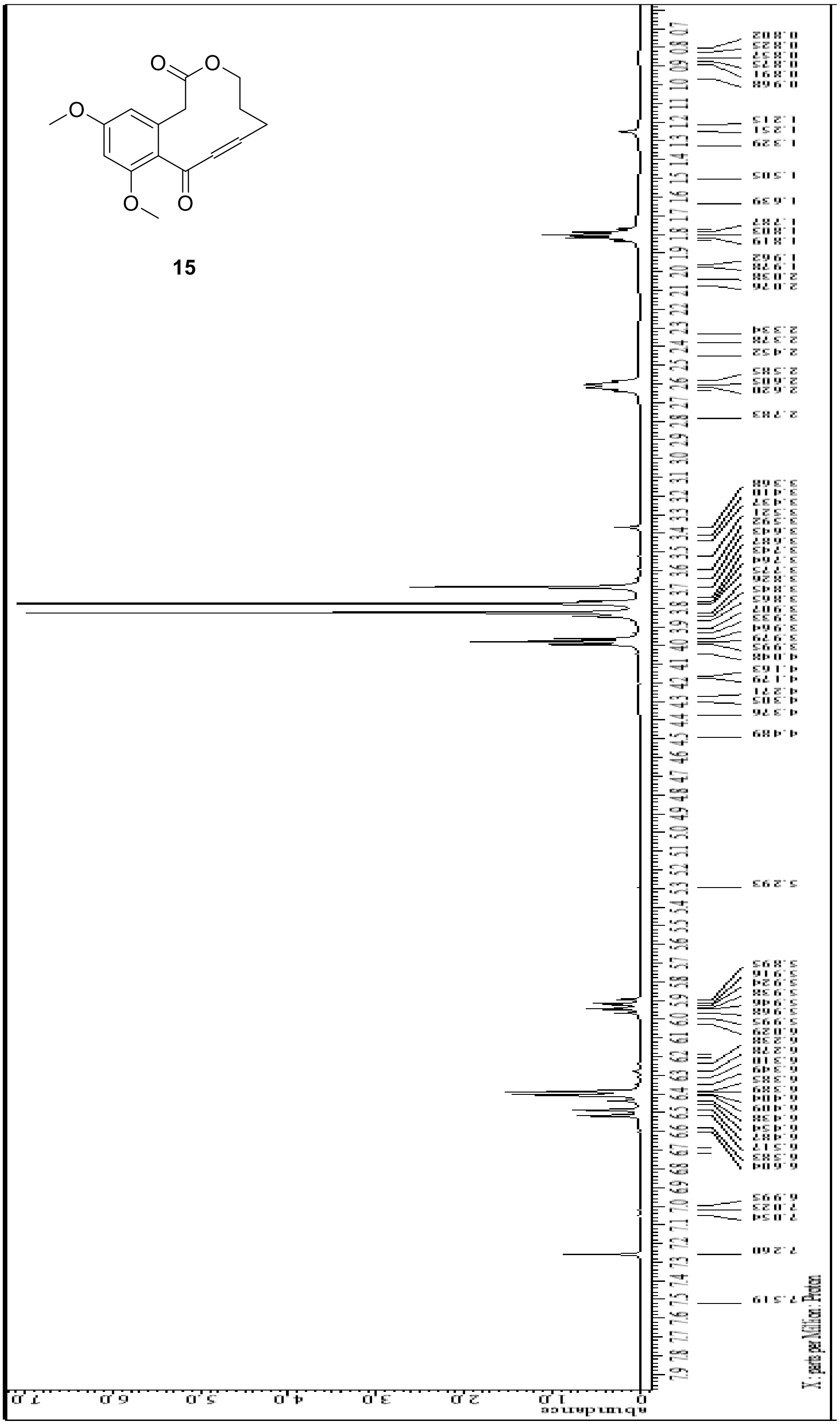
15
abundance
X : parts per Million : Proton

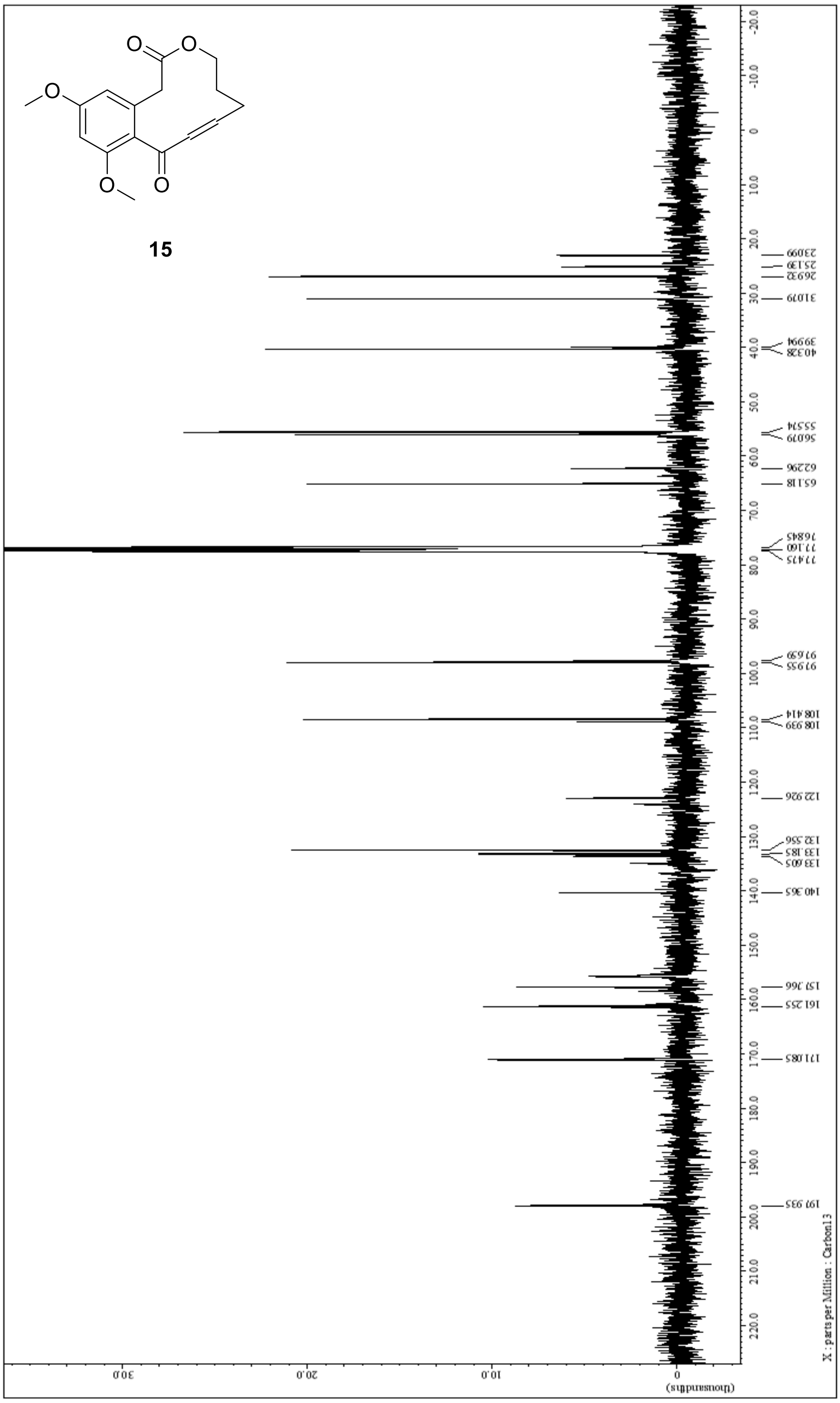

Figure S11. NMR spectrums of 10,12-dimethoxy-5,6-dihydrobenzo[*d*][1]oxacycloundecine-2,9(1*H*,4*H*)-dione (**15**)

Synthetic procedures and characterization data for the compound **16**

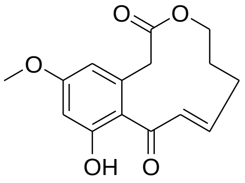

16

(*E*)-10-hydroxy-12-methoxy-5,6-dihydrobenzo[*d*][1]oxacycloundecine-2,9(1*H*,4*H*)-dione (16)

A flame-dried 50 mL round-bottom flask equipped with a magnetic stir bar was charged with anhydrous ACN (10 mL), followed by adding compound **14** (204 mg, 0.70 mmol), Al powder (190 mg, 7.03 mmol), and DMSO (250 μL, 3.51 mmol). Iodine (589 mg, 2.32 mmol) was added, and the mixture was stirred at 80 °C for 30 min. After completion of the reaction, the mixture was cooled to room temperature and diluted with aq. HCl (2 M, 5 mL). The mixture was extracted with EtOAc (3 × 20 mL) using a separatory funnel, and the organic layer was dried over $MgSO_4$. After filtration, the mixture was concentrated using a rotary evaporator, and the reaction mixture was purified using flash column chromatography (hexane/EtOAc, 7:3) to obtain **16** (77 mg, 40%).

TLC R_f= 0.55 (hexane/EtOAc, 1:1). ^{1}H NMR (600 MHz, $CDCl_3$) δ 6.45 (d, *J* = 2.5 Hz, 1H, H-4), 6.40 (d, *J* = 2.5 Hz, 1H, H-6), 6.36 (dt, *J* = 16.3, 1.5 Hz, 1H, H-10), 5.91 (dt, *J* = 16.2, 7.0 Hz, 1H, H-11), 4.31 (t, *J* = 6.0 Hz, 2H, H-14), 3.83 (s, 3H, OMe), 3.46 (s, 2H, H-2), 2.40 (m, 2H, H-12), 1.97 (m, 2H, H-13); ^{13}C NMR (150 MHz, $CDCl_3$) δ 200.6, 171.7, 164.4, 163.6, 147.0, 137.3, 131.7, 116.1, 113.5, 100.3, 64.8, 55.7, 42.2, 30.5, 24.6; HRESIMS *m/z* 277.0906 $[M + H]^+$ (calcd for $C_{15}H_{17}O_5$, 277.1076).

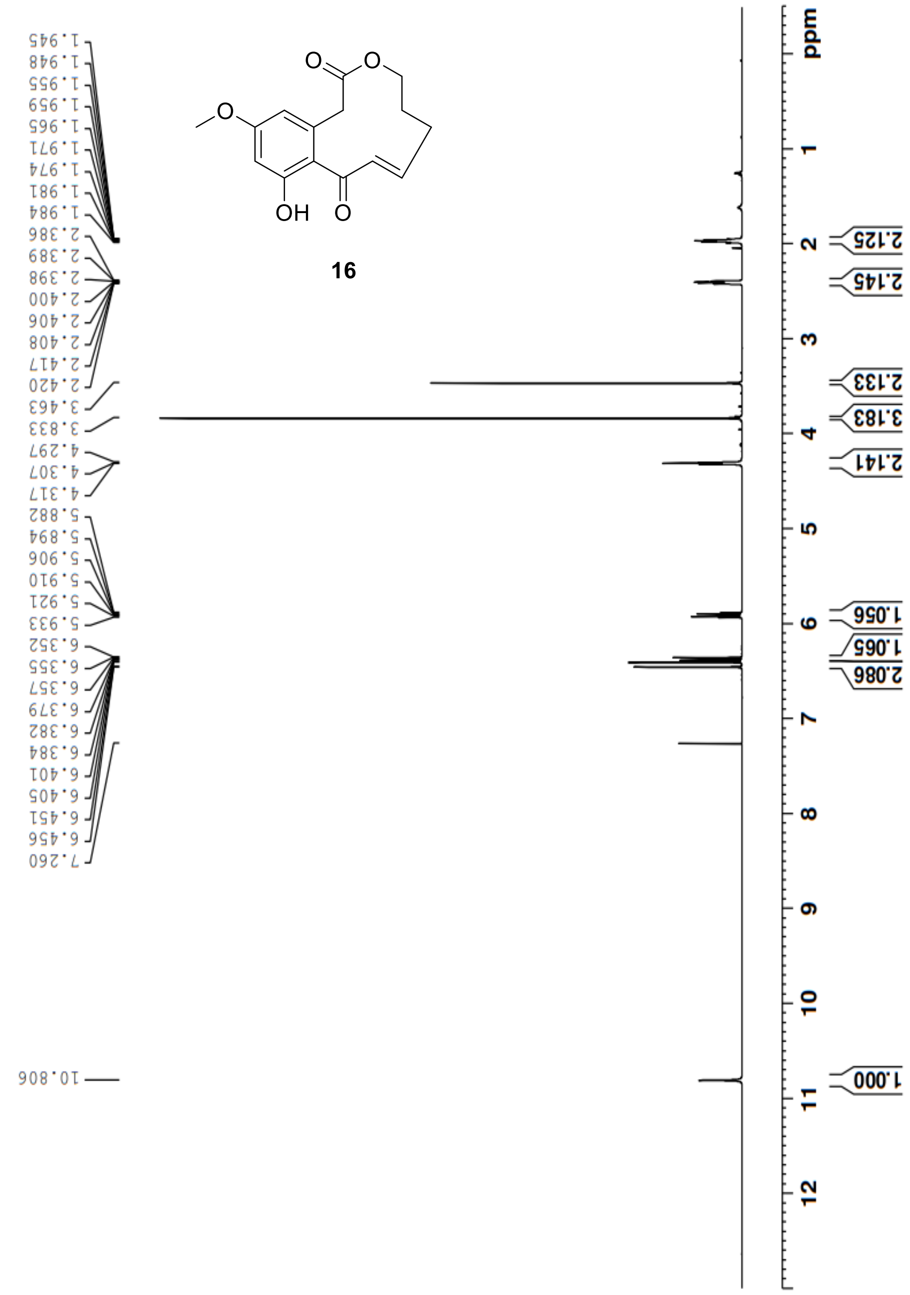
16
ppm

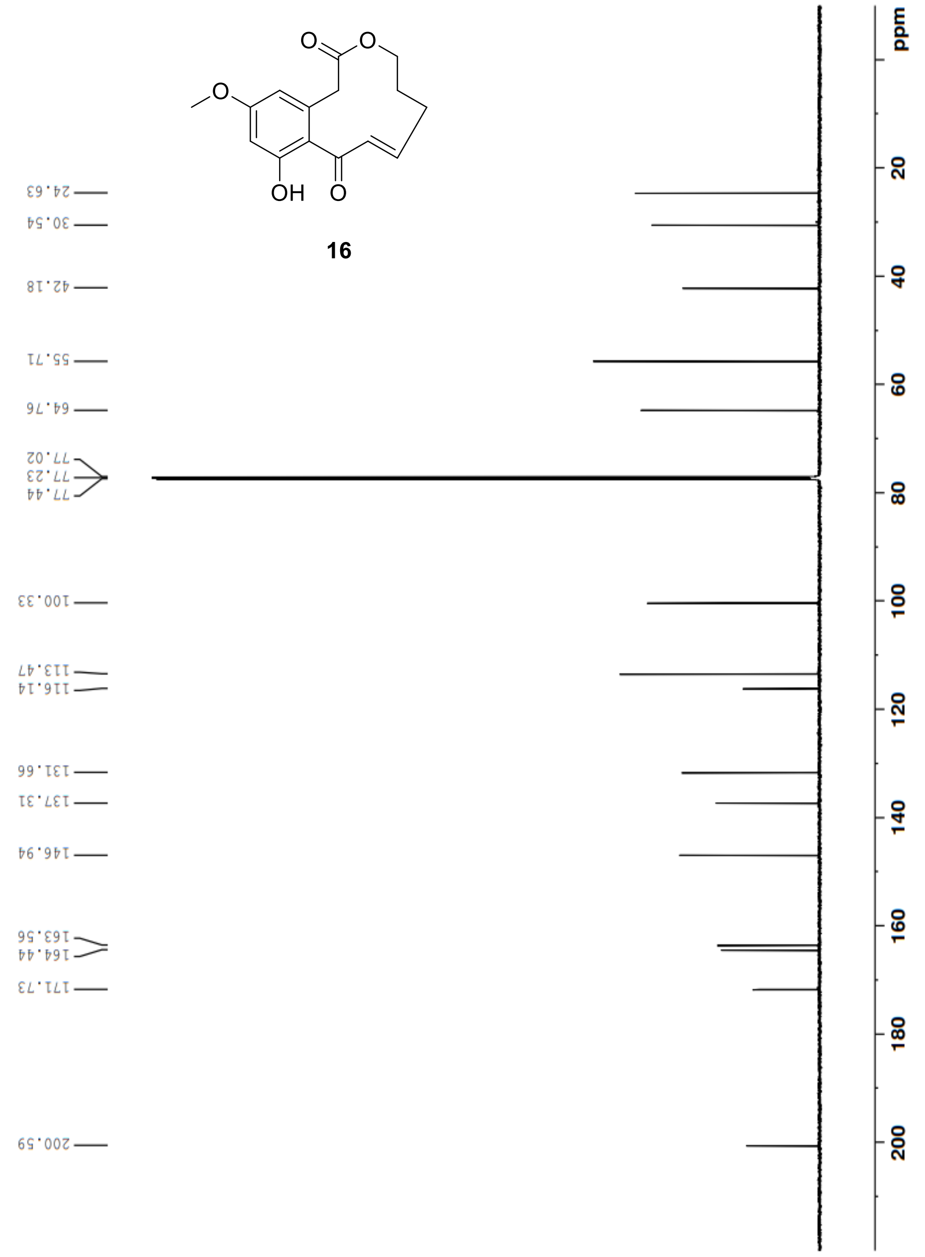

OH
16
24.63
30.54
42.18
55.71
64.76
77.02
77.23
77.44
100.33
113.47
116.14
131.66
137.31
146.94
163.56
164.44
171.73
200.59
ppm
20
40
60
80
100
120
140
160
180
200

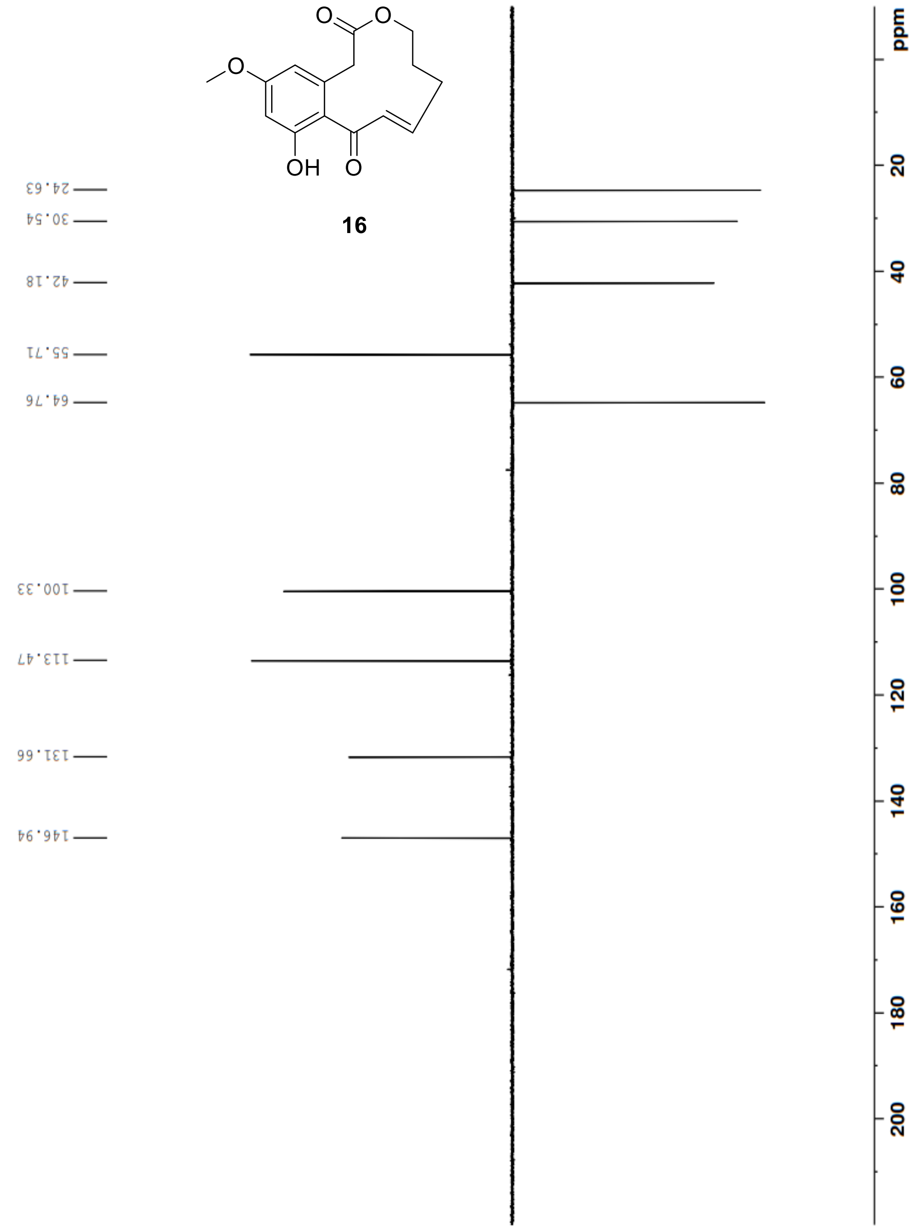
24.63
30.54
42.18
55.71
64.76
100.33
113.47
131.66
146.94
ppm
20
40
60
80
100
120
140
160
180
200
OH
O
O
O

16

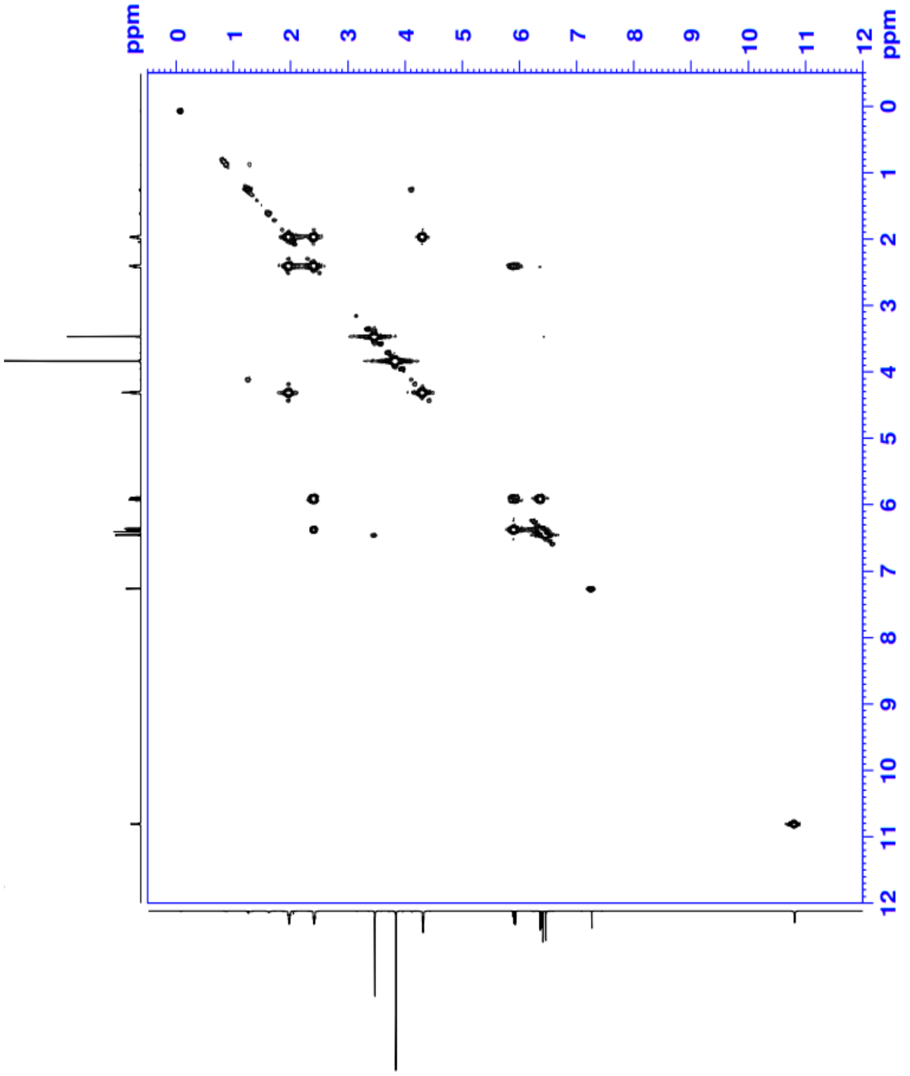
ppm
0
1
2
3
4
5
6
7
8
9
10
11
12
ppm

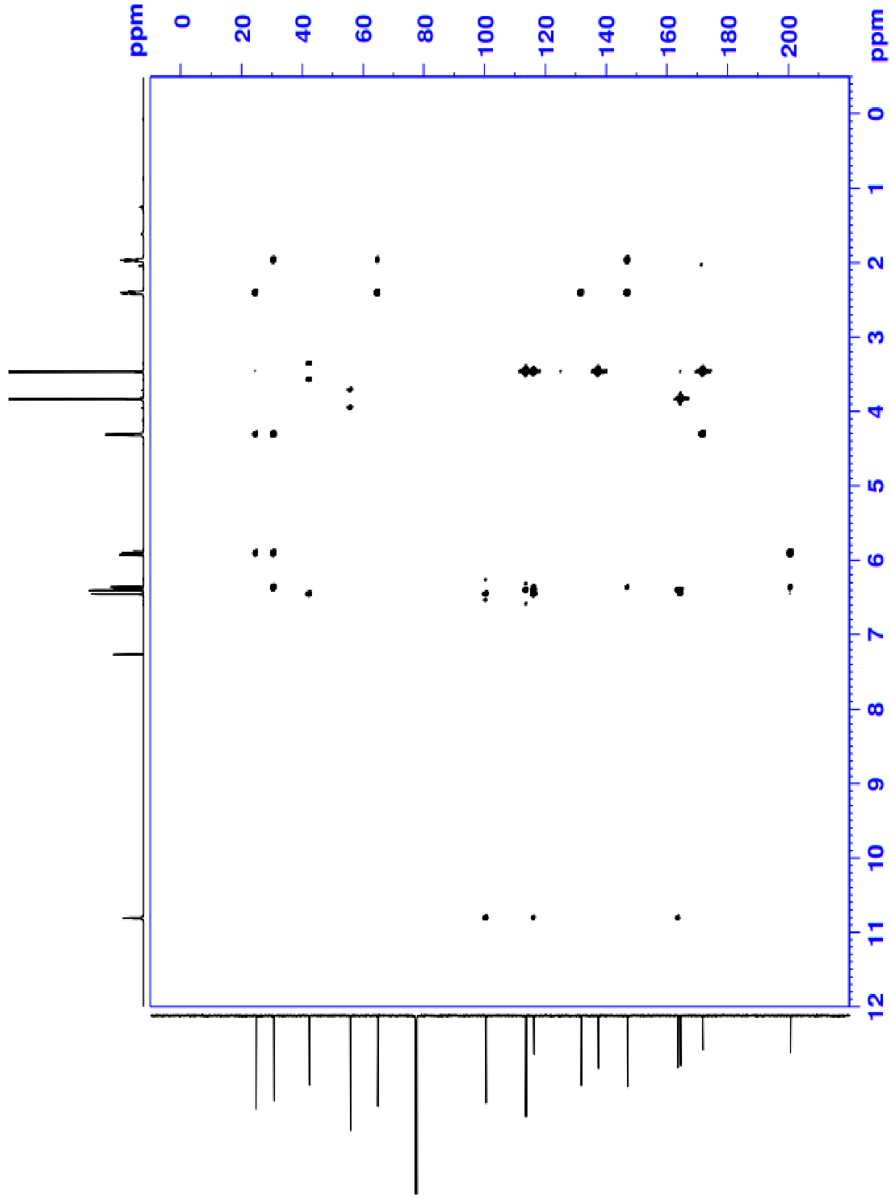

ppm
0 20 40 60 80 100 120 140 160 180 200
ppm
0 1 2 3 4 5 6 7 8 9 10 11 12

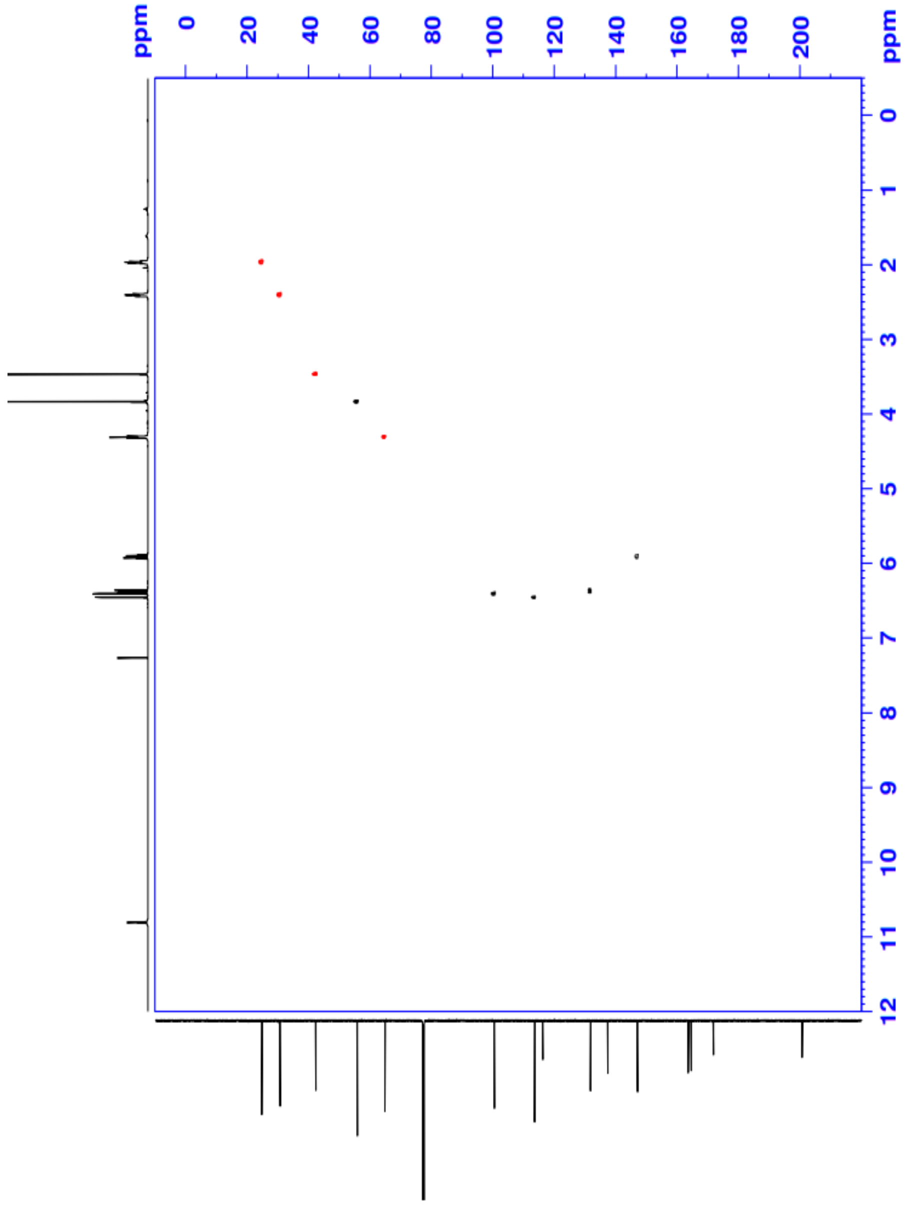

Figure S12. NMR spectrums of (*E*)-10-hydroxy-12-methoxy-5,6-dihydrobenzo[*d*][1]oxacycloundecine-2,9(1*H*,4*H*)-dione (**16**)

Synthetic procedures and characterization data for the compound **18**

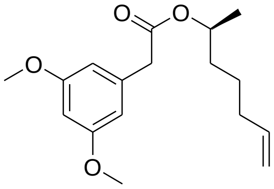

18

(*S*)-hept-6-en-2-yl 2-(3,5-dimethoxyphenyl)acetate (18)

A flame-dried 100 mL round-bottom flask equipped with a magnetic stir bar was charged with anhydrous DCM (10 mL), 3,5-Dimethoxyphenylacetic Acid (**1**) (4.0 g, 20.4 mmol), (S)-hept-6-en-2-ol (**17**) (2.87 mL, 21.4 mmol), and Mukaiyama reagent (2-Chloro-1-methylpyridinium iodide) (5.47 g, 21.4 mmol). The mixture was stirred at room temperature for 15 min. Then, TEA (8.53 mL, 61.2 mmol) was slowly added to the mixture, and the mixture was stirred at room temperature for 20 h. The reaction mixture was filtered and purified by flash column chromatography (hexane/EtOAc, 95:5) to obtain 18 (3.88 g, 65%).

TLC R_f= 0.67 (hexane/EtOAc, 7:3). ^{1}H NMR (400 MHz, CD_3OD) δ 6.43 (d, *J* = 2.3 Hz, 1H, H-4; 1H, H-8), 6.37 (t, *J* = 2.3 Hz, 1H, H-6), 5.74 (m, 1H, H-5′), 4.96 (m, 2H, H-6′), 4.93 (m, 1H, H-1′), 4.90 (m, 2H, H-6′), 3.75 (s, 3H, H-5 OMe; 3H, H-7 OMe), 3.52 (s, 2H, H-2), 1.99 (m, 2H, H-4′), 1.54 (m, 2H, H-2′), 1.34 (m, 2H, H-3′), 1.20 (d, *J* = 6.4 Hz, 3H, H-7′); ^{13}C NMR (100 MHz, CD_3OD) δ 173.1, 162.4, 162.4, 139.6, 137.8, 115.1, 108.3, 108.3, 100.0, 72.7, 55.7, 55.7, 42.8, 36.4, 34.5, 25.7, 20.2; HRESIMS *m/z* 293.1761 $[M + H]^+$ (calcd for $C_{17}H_{25}O_4$, 293.1753).

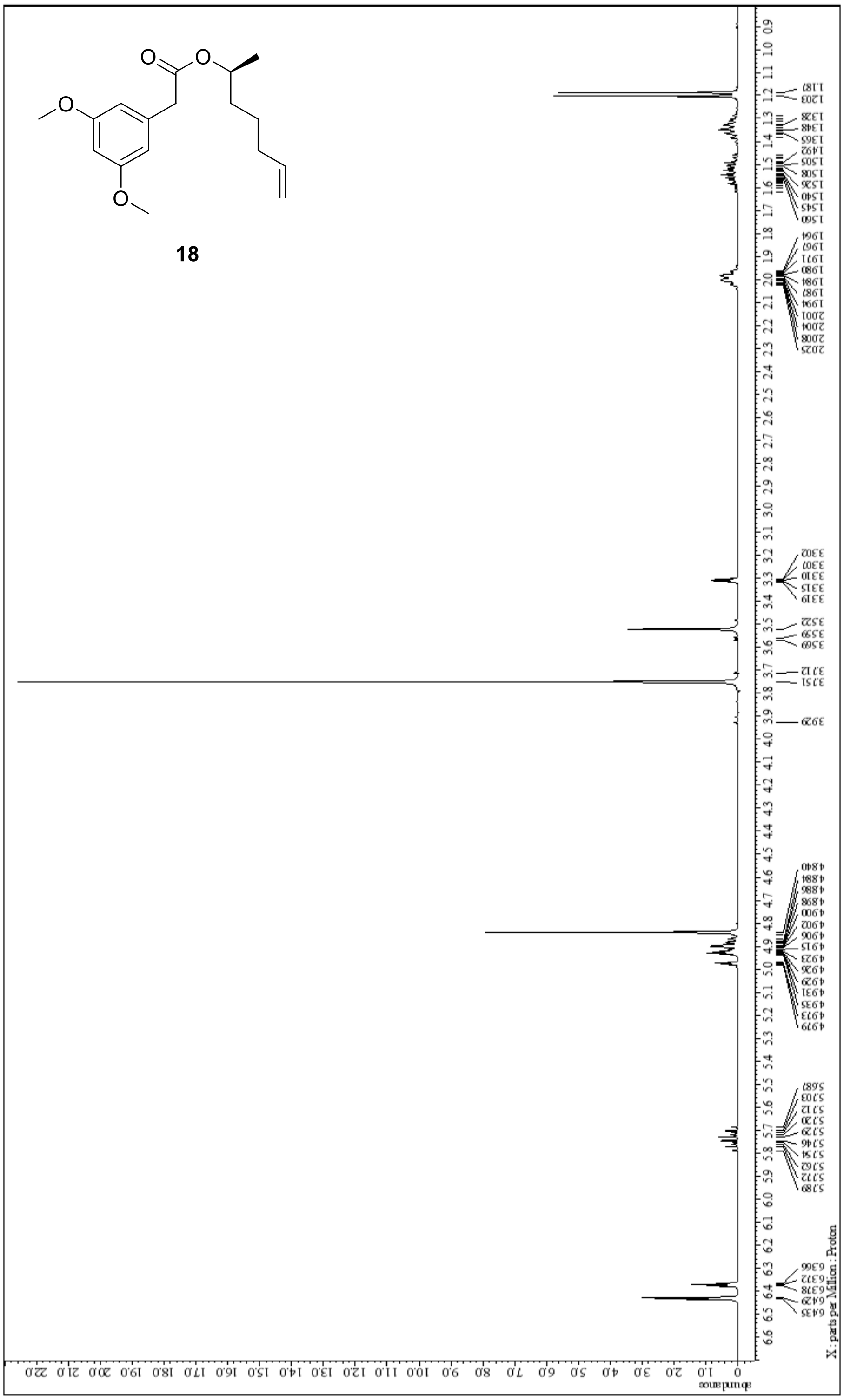
18
X : parts per Million : Proton
abundance

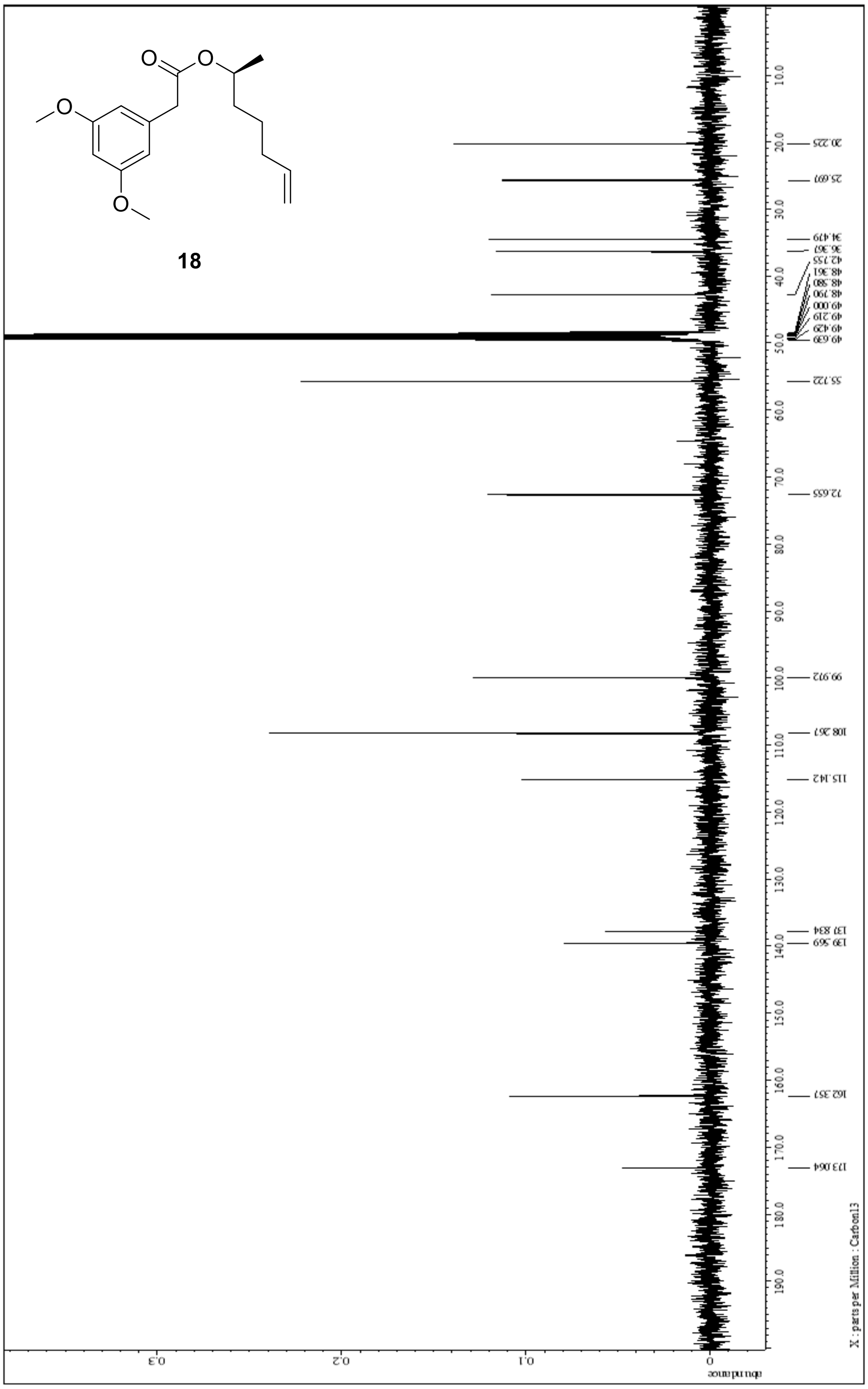
18
20.225
25.697
34.419
36.367
42.755
48.361
48.580
48.790
49.000
49.219
49.429
49.639
55.122
72.655
99.912
108.267
115.142
137.834
139.569
162.357
173.064
X : parts per Million : Carbon13
abundance

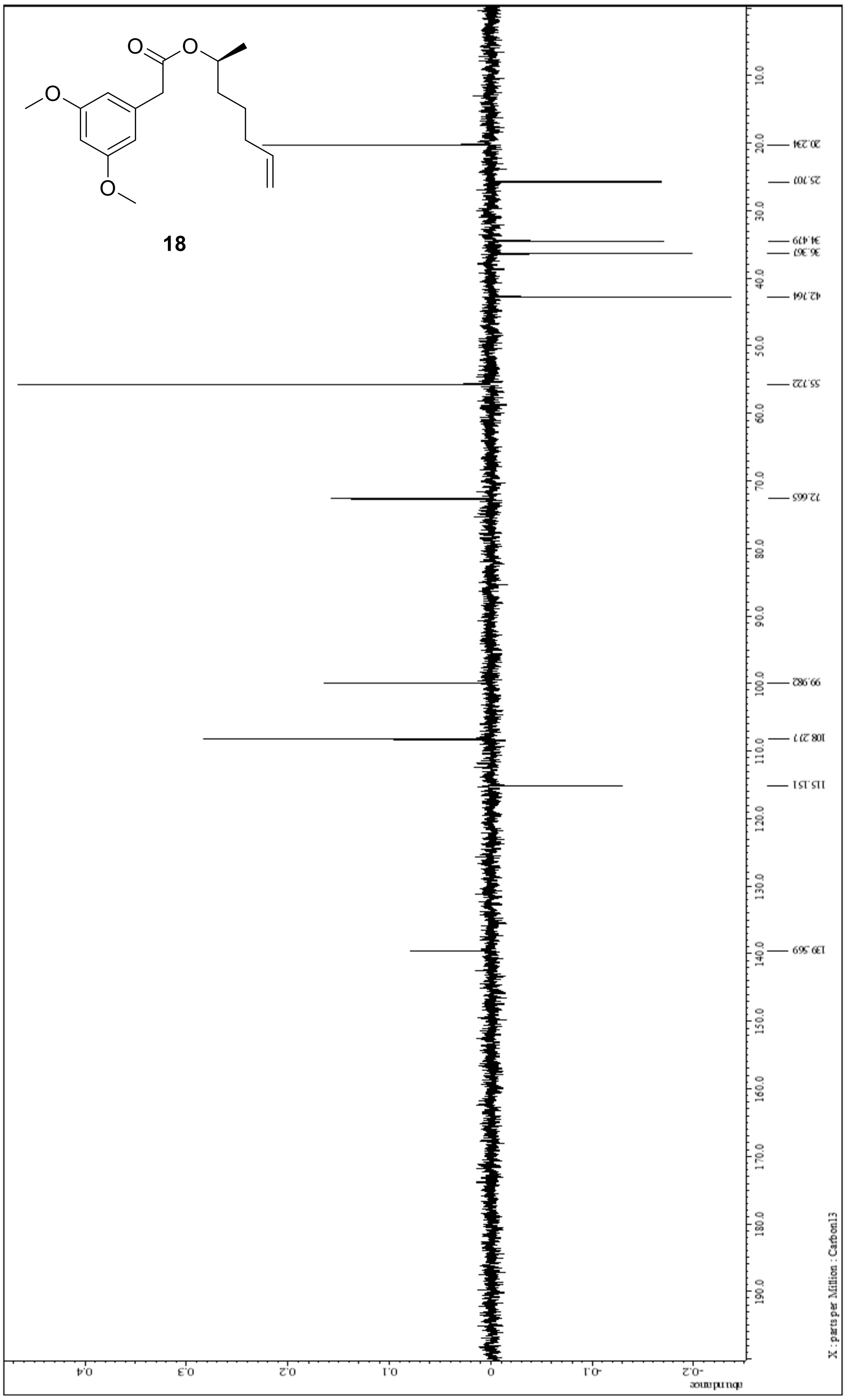

Figure S13. NMR spectrums of (*S*)-hept-6-en-2-yl 2-(3,5-dimethoxyphenyl)acetate (**18**)

Synthetic procedures and characterization data for the compound **19**

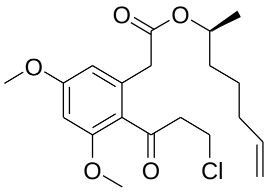

19

(*S*)-hept-6-en-2-yl 2-(2-(3-chloropropanoyl)-3,5-dimethoxyphenyl)acetate (19)

A flame-dried 500 mL round-bottom flask equipped with a magnetic stir bar was charged with anhydrous DCM (89 mL), and 3-chloropropanoyl chloride (**9**) (2 mL, 21 mmol) was added. After cooling to –78 °C, SnCl4 (1 M in DCM, 21 mL, 21 mmol) was added and stirred for 15 min. Compound **18** (4.73 g, 16.2 mmol) was dissolved in DCM (89 mL) and added to the reaction mixture. The reaction temperature was then raised to –20 °C and stirred for 2 h 30 min. After completion of the reaction, the reactant was poured into a separatory funnel filled with ice (100 g) and H_2O (100 mL), and extraction was performed by adding DCM. The obtained organic layer was washed with sat. aq. After washing with $NaHCO_3$ (70 mL) and H_2O (70 mL), the mixture was dried with $MgSO_4$, filtered, and concentrated using a rotary evaporator. The reaction mixture was purified using flash column chromatography (hexane/EtOAc, 9:1) to obtain **19** (4.5 g, 72%).

TLC R_f= 0.5 (hexane/EtOAc, 7:3). ^{1}H NMR (600 MHz, $CDCl_3$) δ 6.40 (d, *J* = 2.3 Hz, 1H, H-6), 6.37 (d, *J* = 2.3 Hz, 1H, H-4), 5.78 (m, 1H, H-5′), 5.00 (m, 2H, H-6′), 4.95 (m, 2H, H-6′), 4.89 (m, 2H, H-1′), 3.83 (s, 3H, H-5 OMe), 3.82 (m, 2H, H-11), 3.81 (s, 3H, H-7 OMe), 3.67 (s, 2H, H-2), 3.35 (t, *J* = 7.1 Hz, 2H, H-10), 2.03 (m, 2H, H-4′), 1.58 (m, 2H, H-2′), 1.49 (m, 2H, H-2′), 1.38 (m, 2H, H-3′), 1.21 (d, *J* = 6.4 Hz, 3H, H-7′); ^{13}C NMR (150 MHz, $CDCl_3$) δ 202.3, 170.9, 161.8, 159.2, 138.4, 135.6, 122.6, 114.6, 108.2, 97.3, 71.4, 55.6, 55.3, 46.9, 39.3, 39.2, 35.2, 33.4, 24.5, 19.8; HRESIMS *m/z* 383.1616 $[M + H]^+$ (calcd for $C_{20}H_{28}ClO_5$, 383.1625).

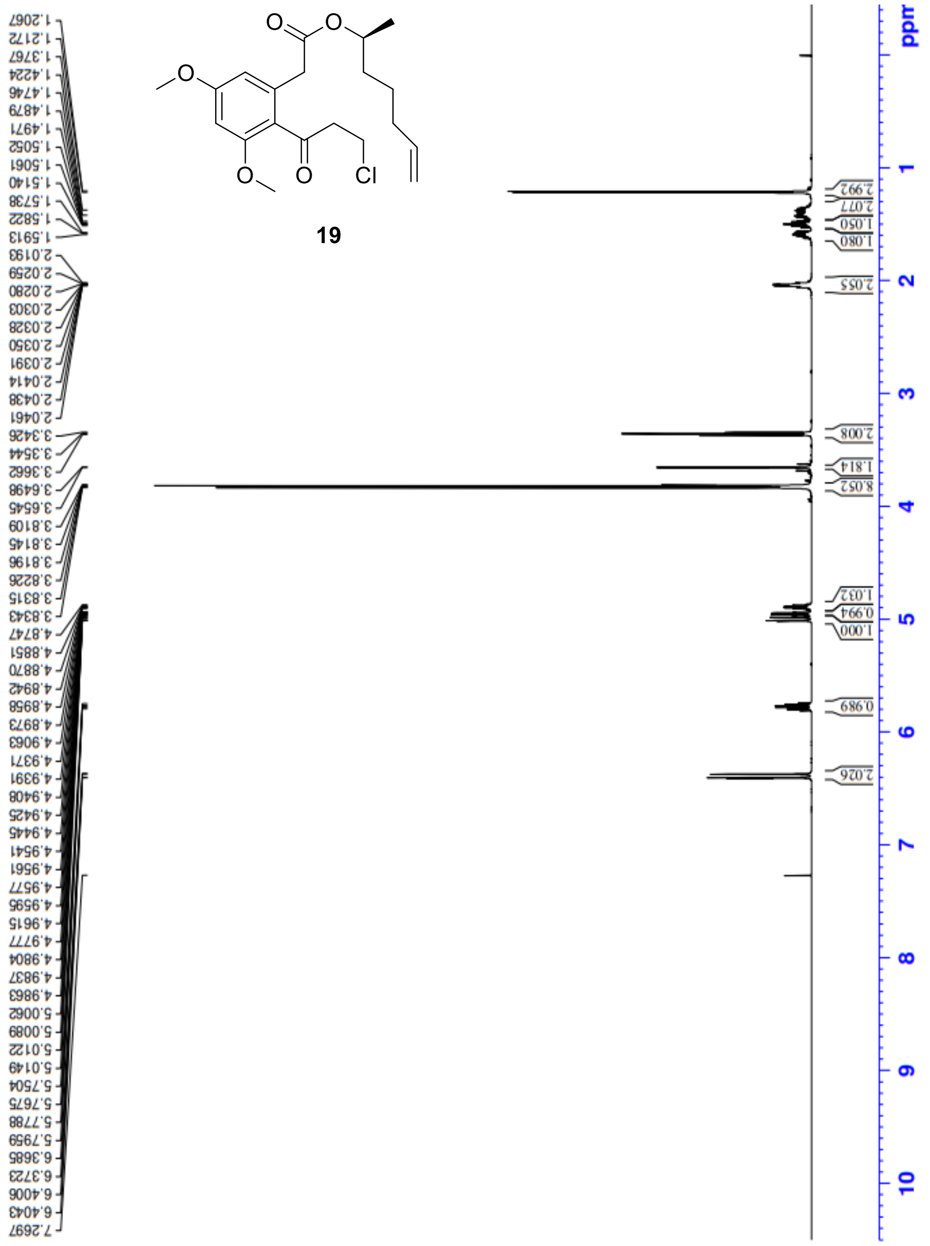
19
ppm

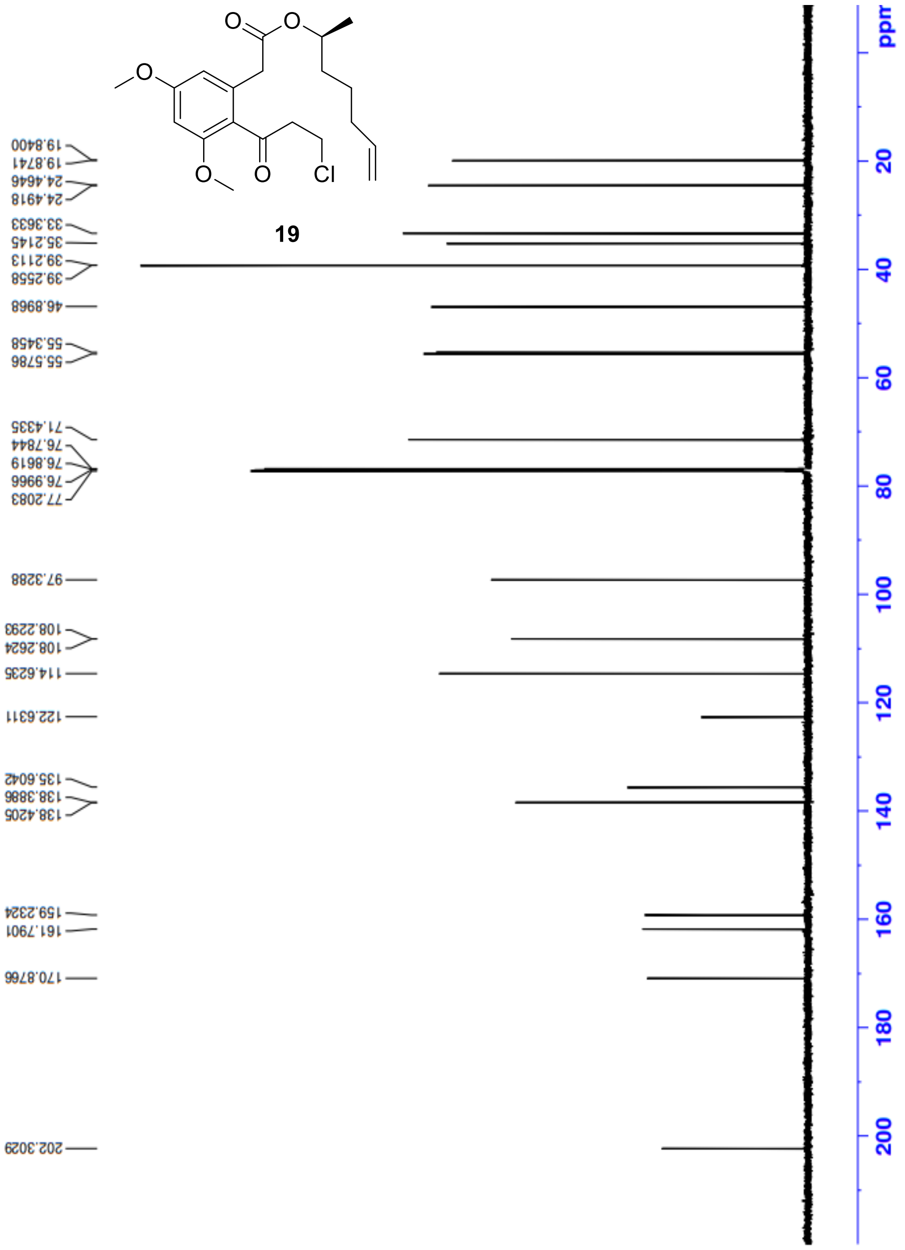
19.8400
19.8741
24.4646
24.4918
33.3633
35.2145
39.2113
39.2558
46.8968
55.3458
55.5786
71.4335
76.7844
76.8619
76.9966
77.2083
97.3288
108.2293
108.2624
114.6235
122.6311
135.6042
138.3886
138.4205
159.2324
161.7901
170.8766
202.3029
ppm
20
40
60
80
100
120
140
160
180
200
O
O
O
O
O
Cl

19

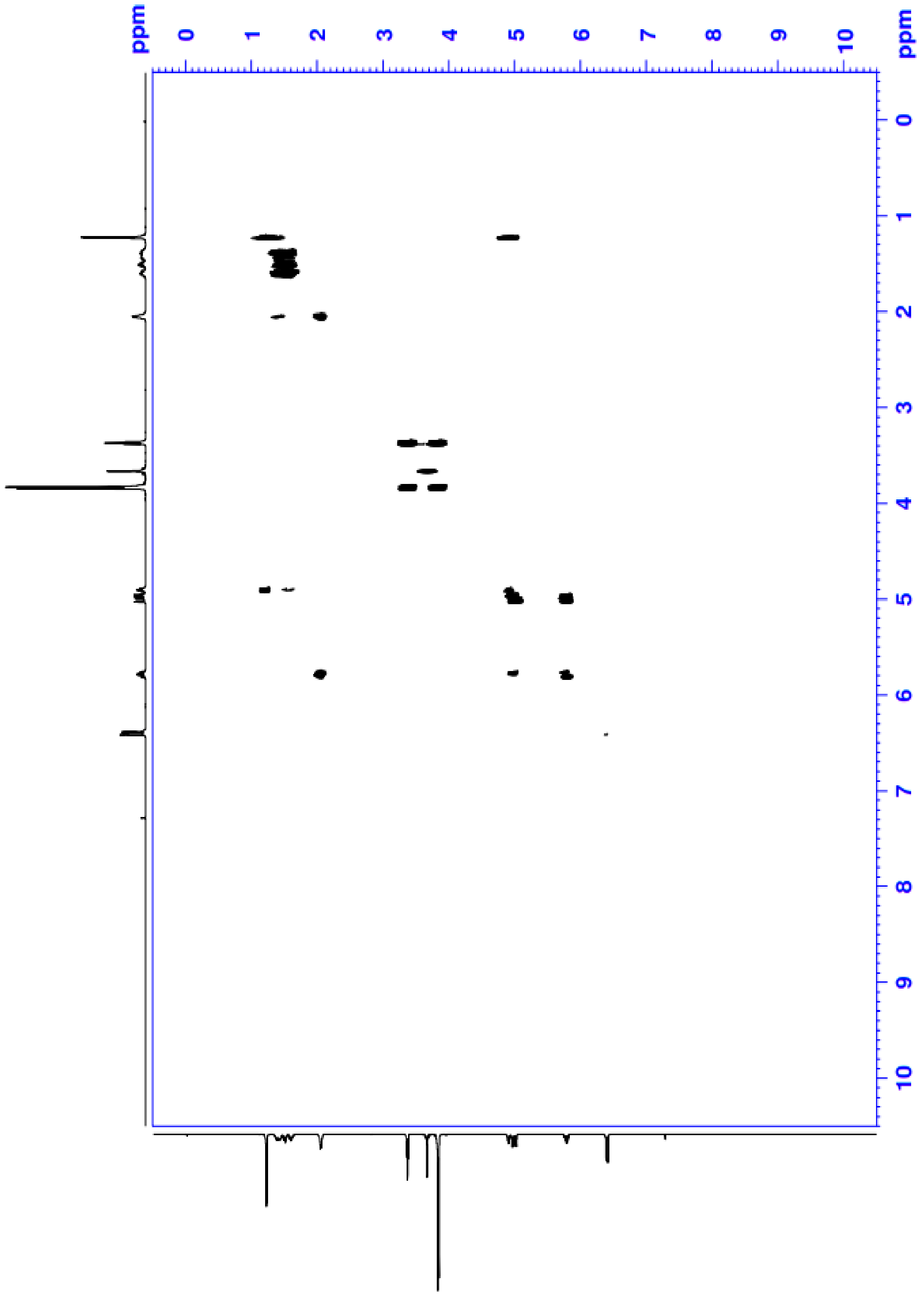
ppm
0 1 2 3 4 5 6 7 8 9 10
ppm
0 1 2 3 4 5 6 7 8 9 10

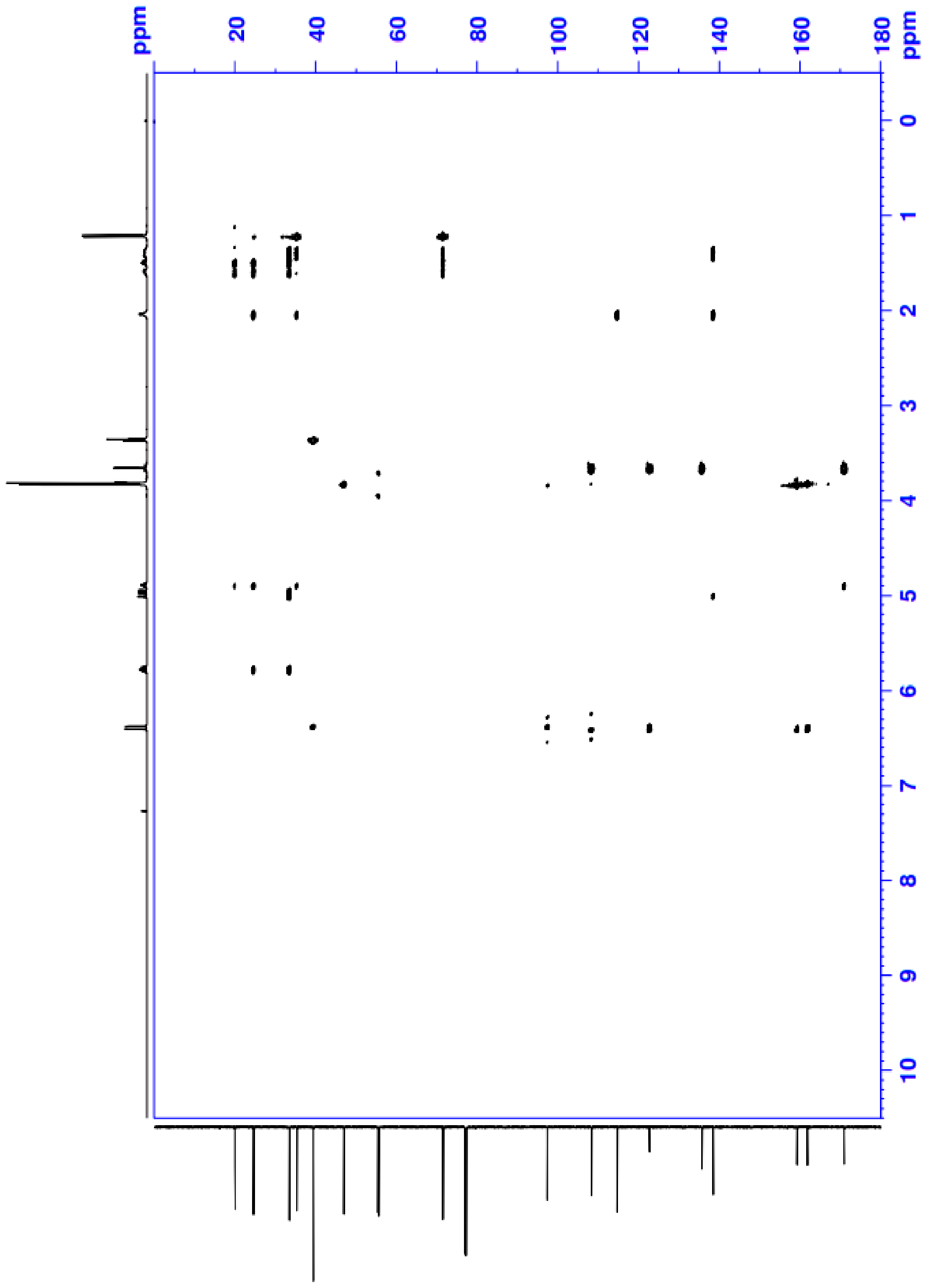
ppm
20
40
60
80
100
120
140
160
180
ppm
0
1
2
3
4
5
6
7
8
9
10

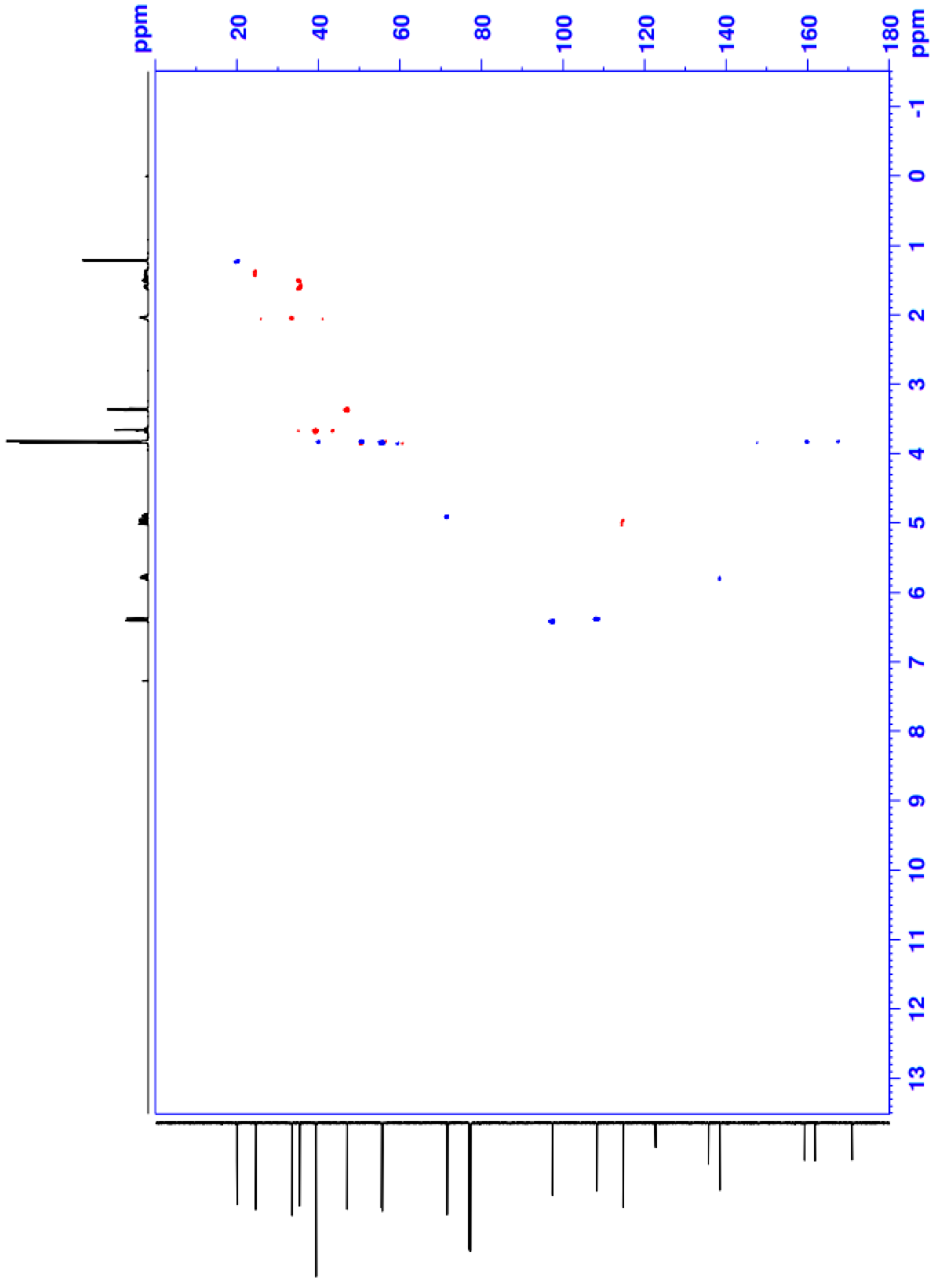

Figure S14. NMR spectrums of (*S*)-hept-6-en-2-yl 2-(2-(3-chloropropanoyl)-3,5-dimethoxyphenyl)acetate (**19**)

Synthetic procedures and characterization data for the compound **20**

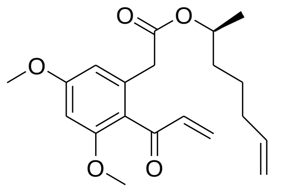

20

(*S*)-hept-6-en-2-yl 2-(2-acryloyl-3,5-dimethoxyphenyl)acetate (20)

A flame-dried 250 mL round-bottom flask equipped with a magnetic stir bar was charged with anhydrous DCM (176 mL), and compound **19** (2.7 g, 8.48 mmol) was added. TEA (2.36 mL, 16.96 mmol) was then added, and the mixture was stirred at room temperature overnight. The reaction mixture, to which sat. aq. NH_4Cl was added, was extracted with DCM (3 × 80 mL) using a separatory funnel. The obtained organic layer was dried over $MgSO_4$, filtered, and concentrated using a rotary evaporator. The reaction mixture was purified using flash column chromatography (hexane/EtOAc, 9:1) to obtain **20** (1.55 g, 63%).

TLC R_f= 0.60 (hexane/EtOAc, 6:4). ^{1}H NMR (600 MHz, $CDCl_3$) δ 6.68 (dd, *J* = 17.4, 10.5 Hz, 1H, H-10), 6.43 (d, *J* = 2.3 Hz, 1H, H-4), 6.41 (d, *J* = 2.3 Hz, 1H, H-6), 6.09 (dd, *J* = 17.4, 1.4 Hz, 2H, H-11), 5.79 (m, 2H, H-11), 5.76 (m, 1H, H-5′), 4.96 (m, 2H, H-6′), 4.94 (m, 2H, H-6′), 4.88 (m, 2H, H-1′), 3.83 (s, 3H, H-5 OMe), 3.78 (s, 3H, H-7 OMe), 3.60 (s, 2H, H-2), 2.03 (m, 2H, H-4′), 1.57 (m, 2H, H-2′), 1.36 (m, 2H, H-3′), 1.18 (d, *J* = 6.4 Hz, 3H, H-7′); ^{13}C NMR (150 MHz, $CDCl_3$) δ 195.8, 170.7, 161.6, 159.1, 138.5, 138.1, 135.4, 128.5, 122.2, 114.6, 107.6, 97.5, 71.4, 55.6, 55.4, 38.9, 35.2, 33.4, 24.5, 19.9; HRESIMS *m/z* 347.1882 $[M + H]^+$ (calcd for $C_{20}H_{27}O_5$, 347.1858).

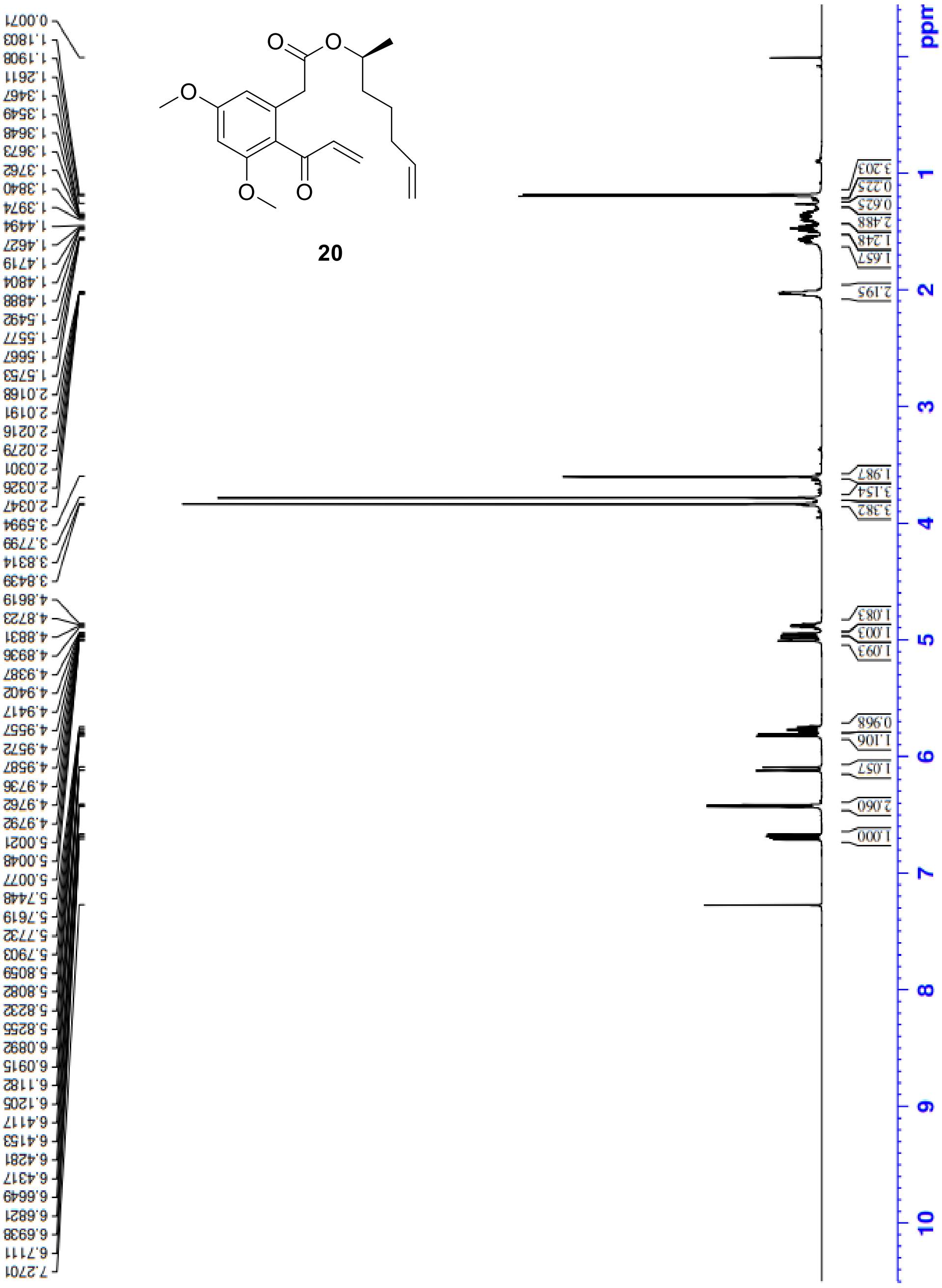
20
ppm

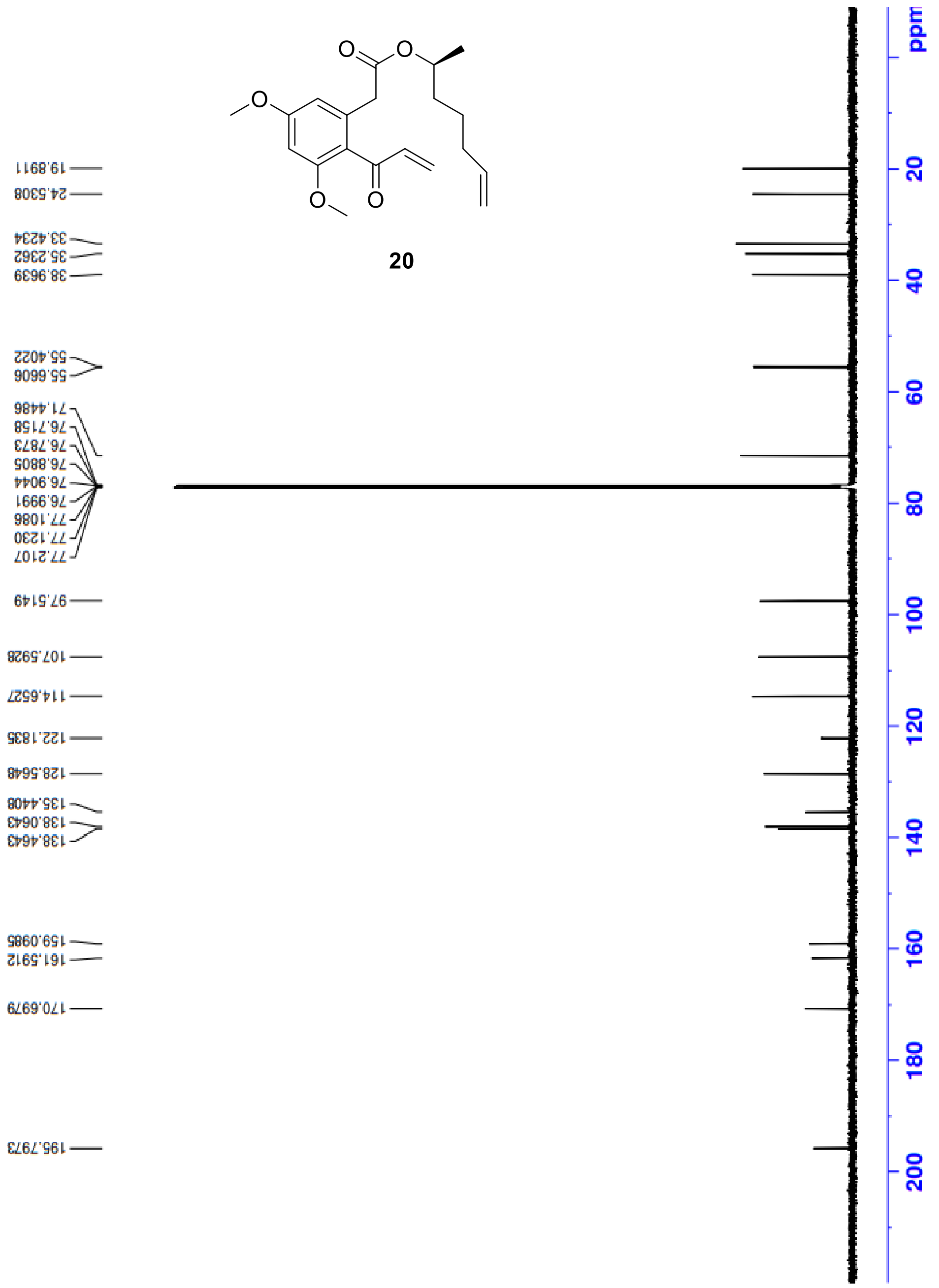

19.8911
24.5308
33.4234
35.2362
38.9639
55.4022
55.6606
71.4486
76.7158
76.7873
76.8805
76.9044
76.9991
77.1086
77.1230
77.2107
97.5149
107.5928
114.6527
122.1835
128.5648
135.4408
138.0643
138.4643
159.0985
161.5912
170.6979
195.7973
20
ppm
20
40
60
80
100
120
140
160
180
200

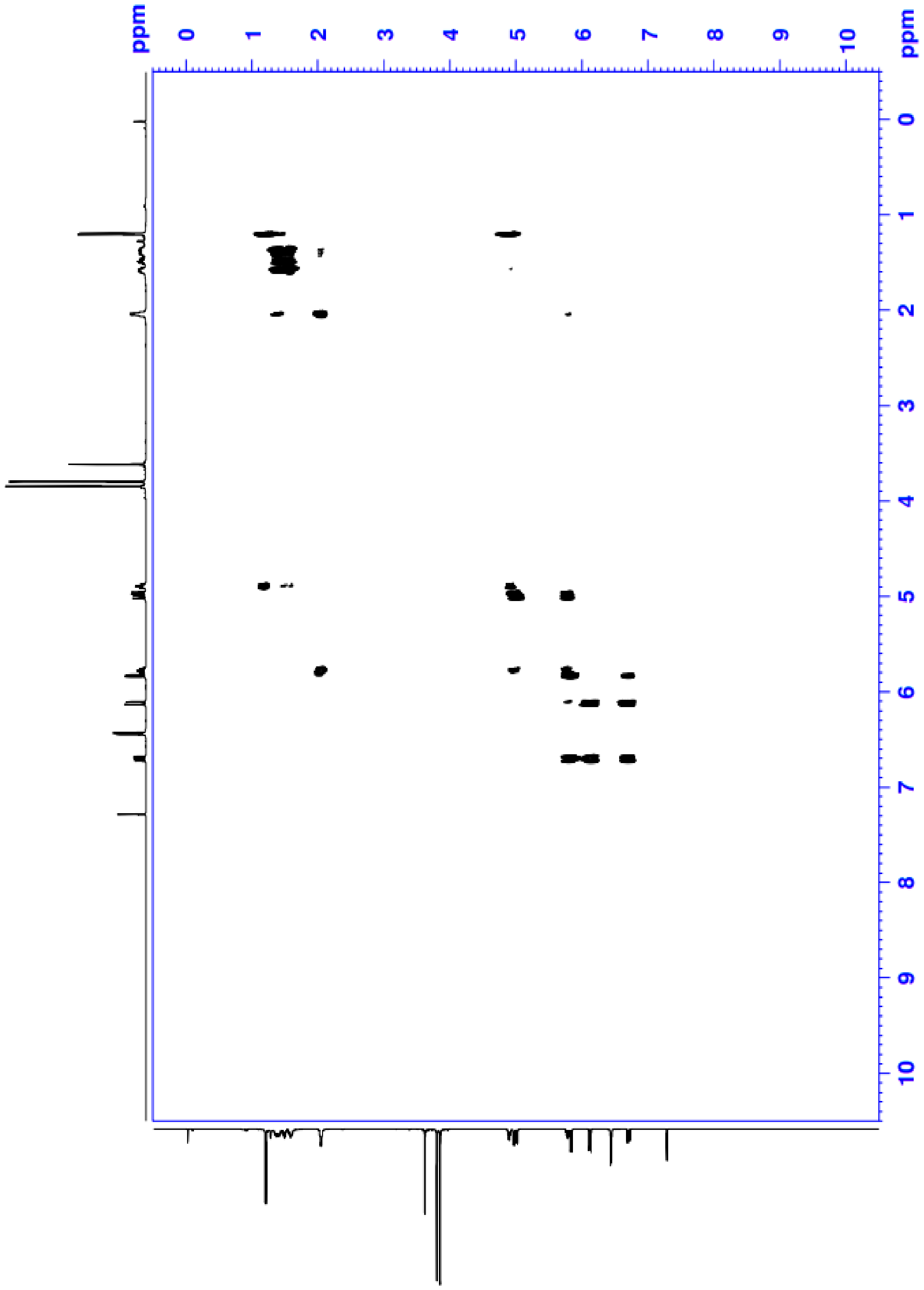
ppm
0
1
2
3
4
5
6
7
8
9
10
ppm

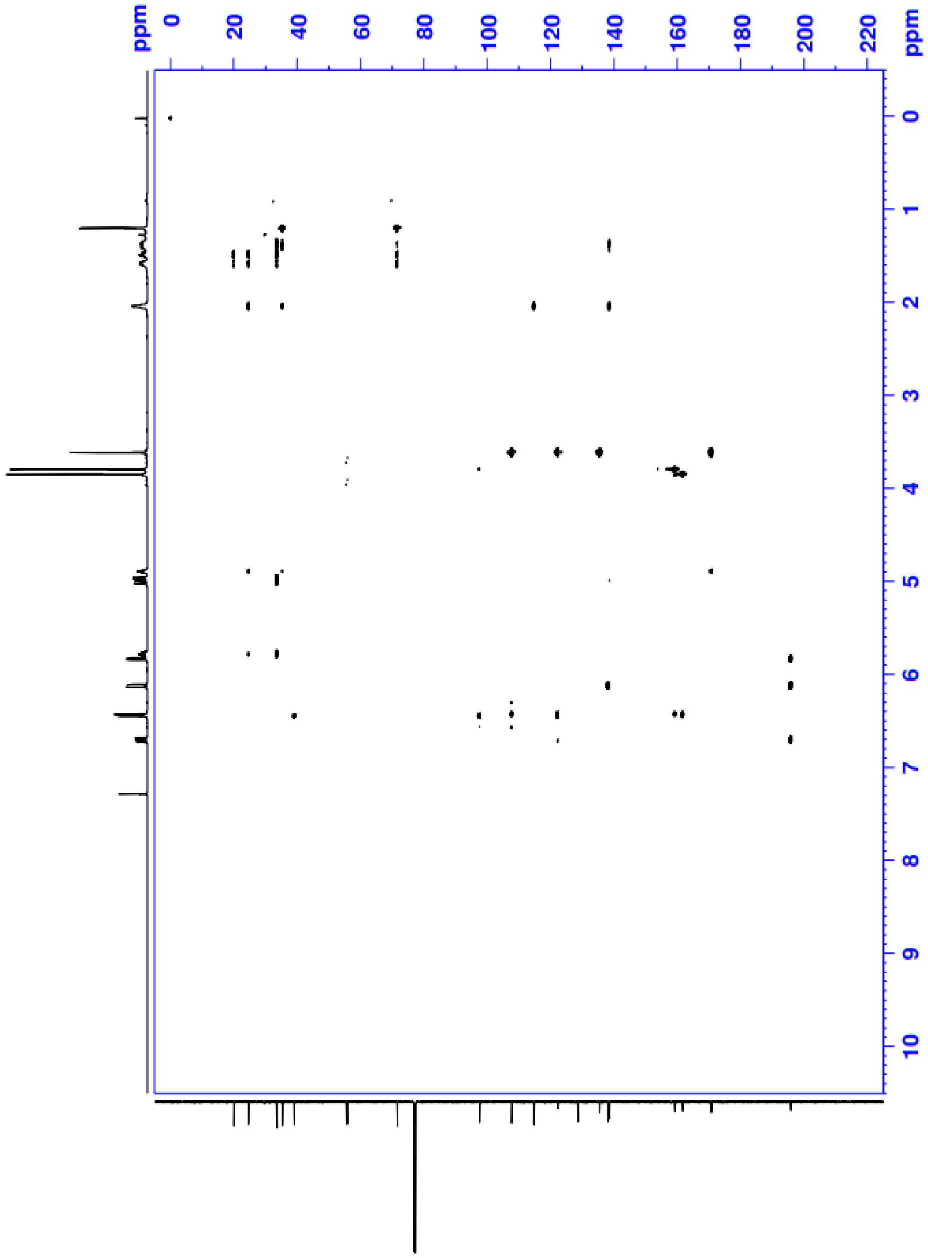
ppm
ppm

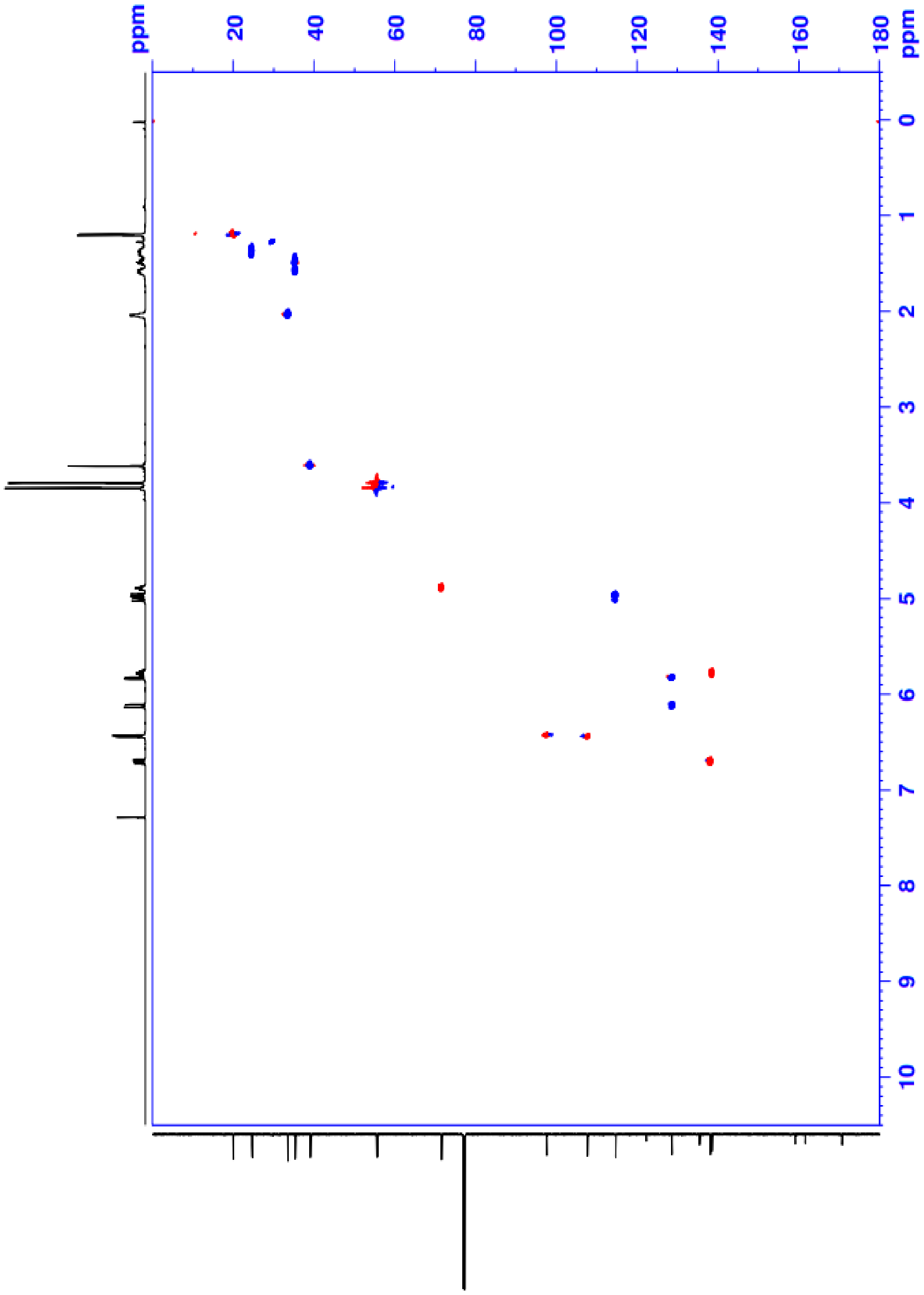

Figure S15. NMR spectrums of (*S*)-hept-6-en-2-yl 2-(2-acryloyl-3,5-dimethoxyphenyl)acetate (**20**)

Synthetic procedures and characterization data for the compound **21**

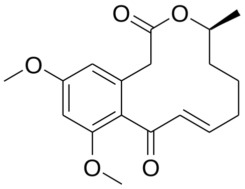

21

(*S*,*E*)-11,13-dimethoxy-4-methyl-4,5,6,7-tetrahydro-2*H*-benzo[*d*][1]oxacyclododecine-2,10(1*H*)-dione (21)

Anhydrous toluene (909 mL) was charged into a flame-dried 2000 mL round-bottom flask equipped with a magnetic stir bar, and compound **20** (1.26 g, 3.64 mmol) was added. Argon gas was bubbled through the mixture for 2h. After bubbling, Grubbs II (618 mg, 20 mol%) catalyst dissolved in toluene (40 mL) was added and stirred at 80 °C for 1 h. The reaction mixture was concentrated using a rotary evaporator and purified using flash column chromatography (hexane/EtOAc, 8:2) to obtain a separated mixture of **21** (475 mg, 41%) and **22** (185 mg, 16%). (41%, *E*-isomer)

TLC R_f= 0.38 (hexane/EtOAc, 6:4). ^{1}H NMR (400 MHz, CD_3OD) δ 6.54 (d, *J* = 2.3 Hz, 1H, H-4), 6.53 (d, *J* = 2.3 Hz, 1H, H-6), 6.48 (dd, *J* = 15.6, 7.8 Hz, 1H, H-11), 6.21 (dt, *J* = 15.6, 1.2 Hz, 1H, H-10), 4.89 (m, 1H, H-15), 3.83 (s, 3H, H-5 OMe), 3.75 (s, 3H, H-7 OMe), 3.37 (s, 2H, H-2), 2.33 (m, 2H, H-12), 2.20 (m, 2H, H-12), 1.90 (m, 2H, H-13), 1.80 (m, 2H, H-14), 1.49 (m, 2H, H-14), 1.42 (m, 2H, H-13), 1.15 (d, *J* = 6.4 Hz, 3H, H-16); ^{13}C NMR (100 MHz, CD_3OD) δ 201.2, 172.0, 162.8, 159.4, 159.0, 134.1, 133.8, 123.4, 108.3, 98.5, 74.5, 56.3, 56.0, 40.2, 35.1, 35.0, 25.5, 20.5; HRESIMS *m/z* 319.1466 $[M + H]^+$ (calcd for $C_{18}H_{23}O_5$, 319.1545).

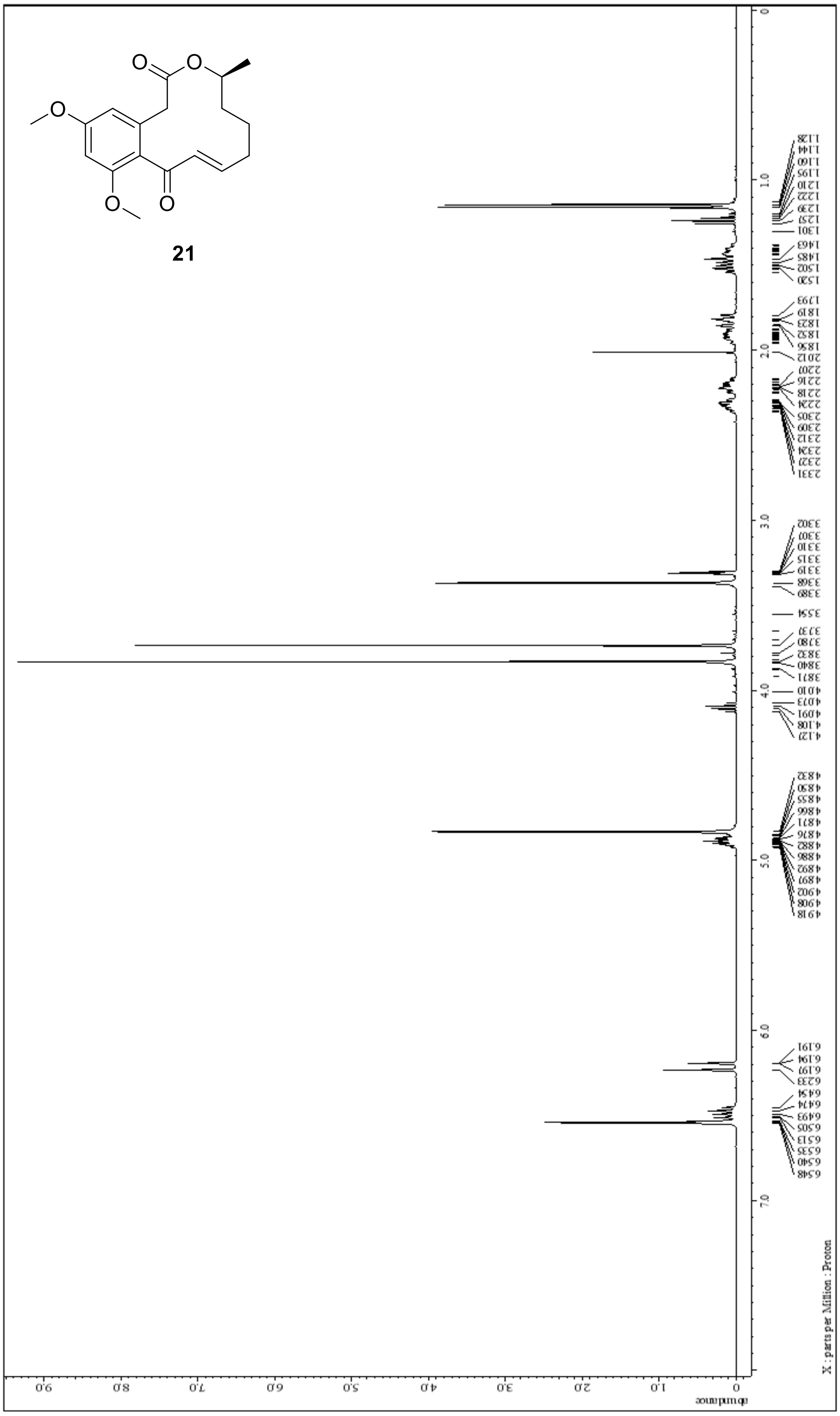
21
X : parts per Million : Proton
abundance

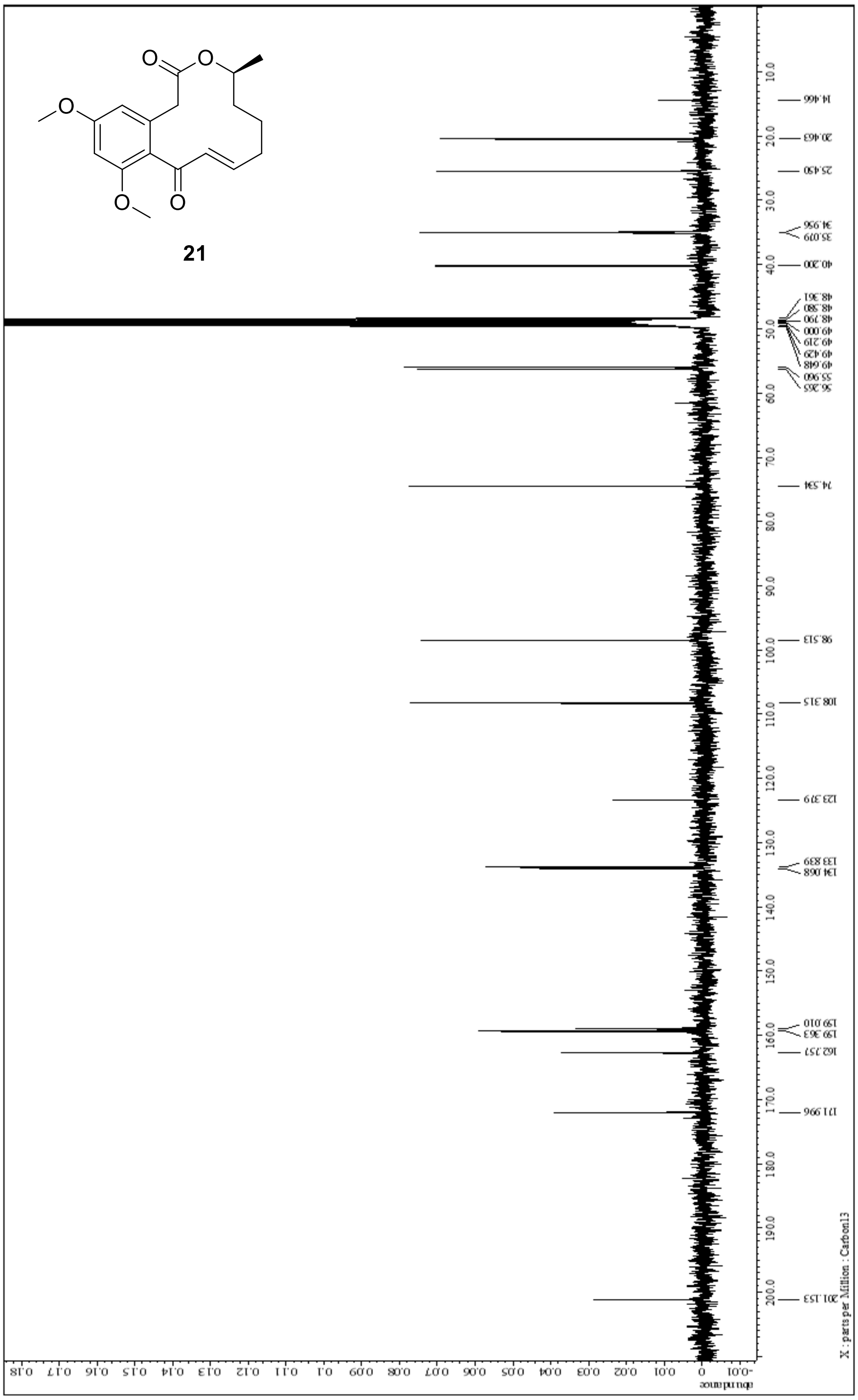
21
14.466
20.463
25.430
34.956
35.079
40.200
48.361
48.580
48.790
49.000
49.219
49.429
49.648
55.960
56.265
74.534
98.513
108.315
123.379
133.839
134.068
159.010
159.363
162.757
171.996
201.153
X : parts per Million : Carbon13
abundance

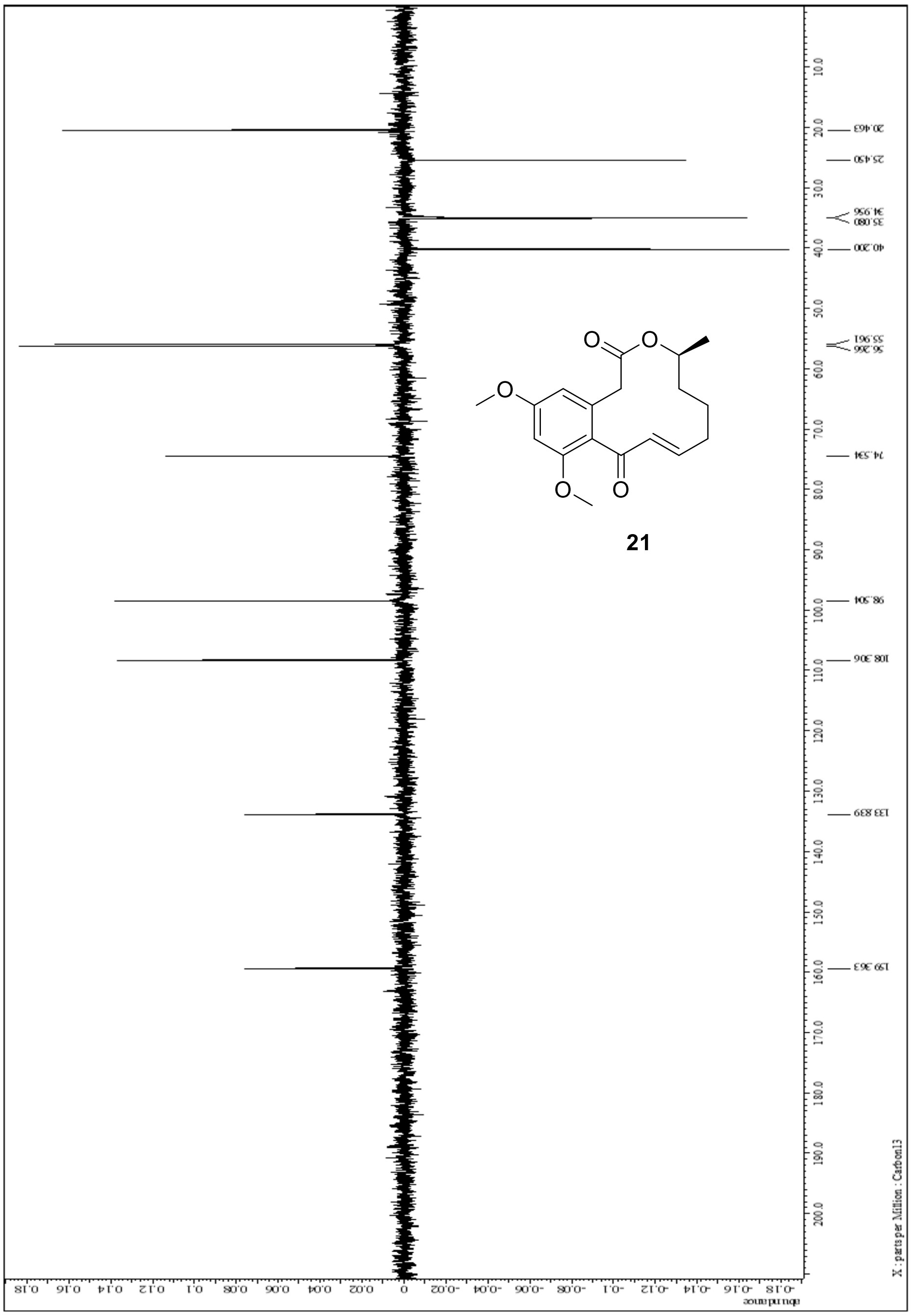

Figure S16. NMR spectrums of (*S*,*E*)-11,13-dimethoxy-4-methyl-4,5,6,7-tetrahydro-2*H*-benzo[*d*][1]oxacyclododecine-2,10(1*H*)-dione (**21**)

Synthetic procedures and characterization data for the compound **22**

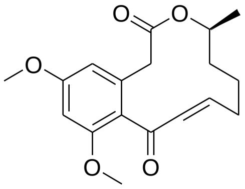

22

(*S*)-11,13-dimethoxy-4-methyl-4,5,6,7-tetrahydro-2*H*-benzo[*d*][1]oxacyclododecine-2,10(1*H*)-dione (22)

Anhydrous toluene (909 mL) was charged into a flame-dried 2000 mL round-bottom flask equipped with a magnetic stir bar, and compound **20** (1.26 g, 3.64 mmol) was added. Argon gas was bubbled through the mixture for 2h. After bubbling, Grubbs II (618 mg, 20 mol%) catalyst dissolved in toluene (40 mL) was added and stirred at 80 °C for 1 h. The reaction mixture was concentrated using a rotary evaporator and purified using flash column chromatography (hexane/EtOAc, 8:2) to obtain a separated mixture of **21** (475 mg, 41%) and **22** (185 mg, 16%). (16%, *Z*-isomer)

TLC R_f= 0.45 (hexane/EtOAc, 6:4). ^{1}H NMR (400 MHz, CD_3OD) δ 6.53 (d, *J* = 1.8 Hz, 1H, H-4), 6.51 (d, *J* = 2.3 Hz, 1H, H-6), 6.37 (dt, *J* = 12.4, 1.6 Hz, 1H, H-10), 5.99 (dt, *J* = 12.4, 8.2 Hz, 1H, H-11), 4.80 (m, 1H, H-15), 3.83 (s, 3H, H-5 OMe), 3.78 (s, 3H, H-7 OMe), 3.37 (s, 2H, H-2), 1.40−2.25 (m, 2H, H-12; 2H, H-13; 2H, H-14), 1.13 (d, *J* = 6.4 Hz, 3H, H-16); ^{13}C NMR (100 MHz, CD_3OD) δ 199.9, 171.8, 163.6, 160.6, 146.3, 135.2, 133.8, 125.4, 109.4, 98.5, 75.2, 56.5, 56.0, 40.1, 31.9, 28.4, 26.0, 19.8; HRESIMS *m/z* 319.1466 $[M + H]^+$ (calcd for $C_{18}H_{23}O_5$, 319.1545).

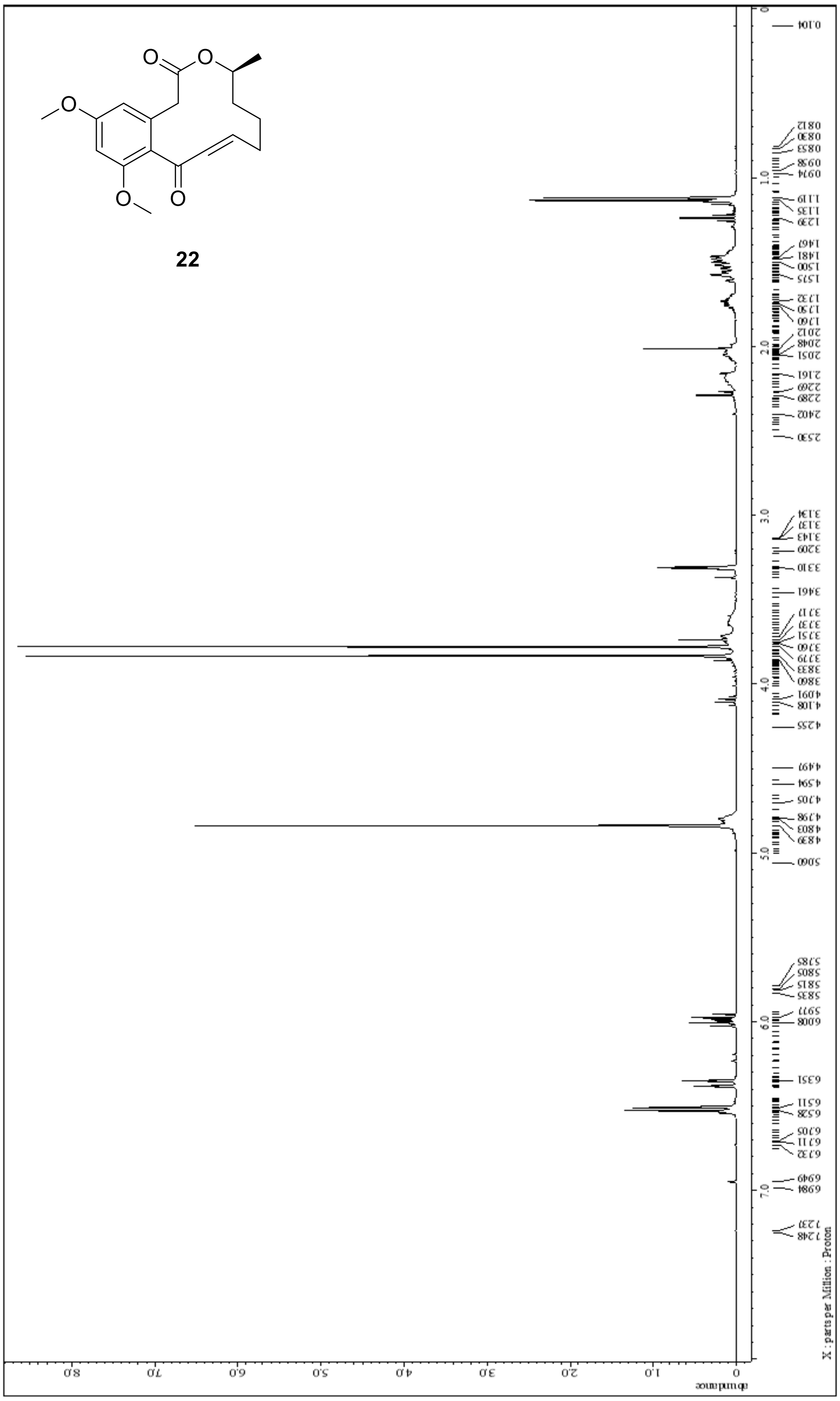
22
X : parts per Million : Proton
abundance

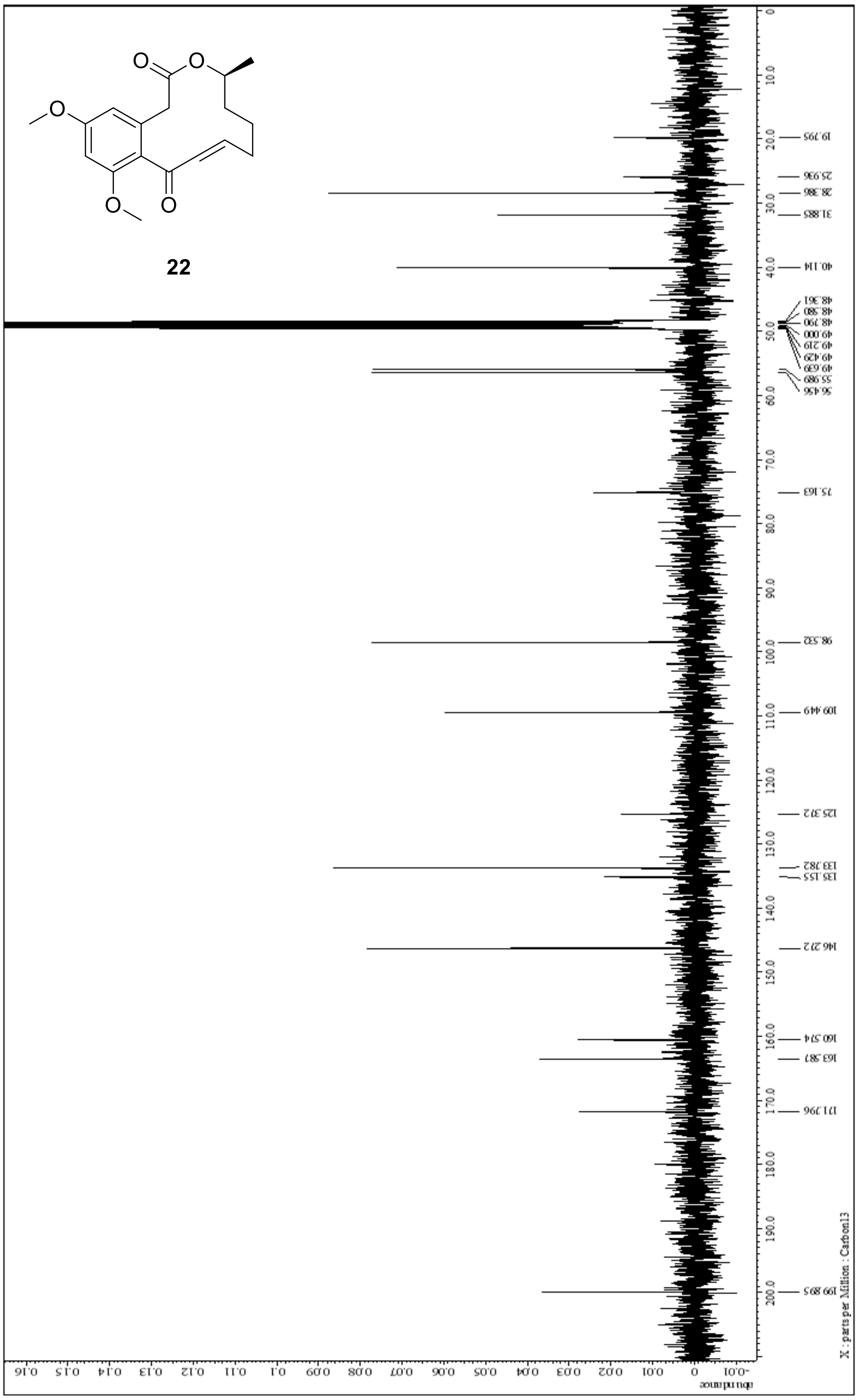

22
19.795
25.936
28.386
31.885
40.114
48.361
48.580
48.790
49.000
49.219
49.429
49.639
55.989
56.456
75.163
98.532
109.449
125.312
133.782
135.155
146.212
160.574
163.587
171.196
199.895
X : parts per Million : Carbon13
abundance

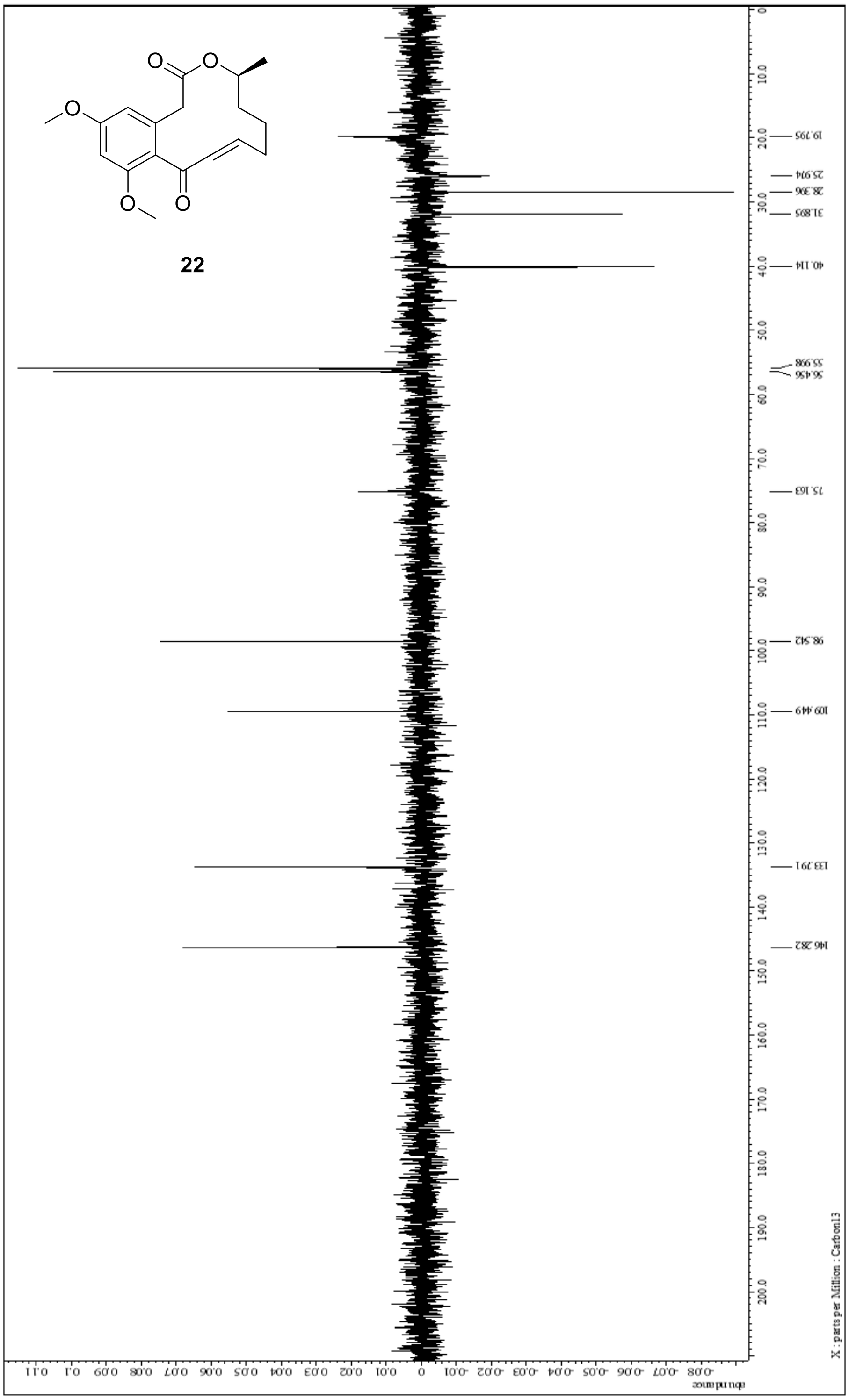

Figure S17. NMR spectrums of (*S*)-11,13-dimethoxy-4-methyl-4,5,6,7-tetrahydro-2*H*-benzo[*d*][1]oxacyclododecine-2,10(1*H*)-dione (**22**)

Synthetic procedures and characterization data for the compound **23**

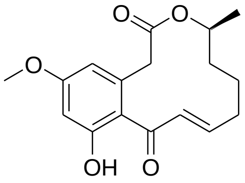

23

(*S*,*E*)-11-hydroxy-13-methoxy-4-methyl-4,5,6,7-tetrahydro-2*H*-benzo[*d*][1]oxacyclododecine-2,10(1*H*)-dione (23)

A flame-dried 50 mL round-bottom flask equipped with a magnetic stir bar was charged with anhydrous ACN (15 mL), followed by adding compound **21** (163 mg, 0.51 mmol), Al powder (138 mg, 5.12 mmol), and DMSO (181 μL, 2.55 mmol). Iodine (427 mg, 1.68 mmol) was added, and the mixture was stirred at 80 °C for 30 min. After completion of the reaction, the mixture was cooled to room temperature and diluted with aq. HCl (2 M, 5 mL). The mixture was extracted with EtOAc (3 × 20 mL) using a separatory funnel, and the organic layer was dried over $MgSO_4$. After filtration, the mixture was concentrated using a rotary evaporator, and the reaction mixture was purified using flash column chromatography (hexane/EtOAc, 7:3) to obtain **23** (70 mg, 45%).

TLC R_f= 0.64 (hexane/EtOAc, 1:1). ^{1}H NMR (400 MHz, $CDCl_3$) δ 6.67 (dd, *J* = 16.5, 1.0 Hz, 1H, H-10), 6.62 (ddd, *J* = 15.1, 8.2, 3.6 Hz, 1H, H-11), 6.41 (d, *J* = 2.8 Hz, 1H, H-6), 6.32 (d, *J* = 2.8 Hz, 1H, H-4), 4.85 (m, 1H, H-15), 4.04 (d, *J* = 17.9 Hz, 2H, H-2), 3.82 (s, 3H, OMe), 3.54 (d, *J* = 17.9 Hz, 2H, H-2), 2.47 (m, 2H, H-12), 2.32 (m, 2H, H-12), 1.97 (m, 2H, H-14), 1.89 (m, 2H, H-13), 1.65 (m, 2H, H-14), 1.64 (m, 2H, H-13), 1.24 (d, *J* = 6.4 Hz, 3H, H-16); ^{13}C NMR (100 MHz, $CDCl_3$) δ 196.4, 171.3, 166.9, 164.4, 148.3, 137.6, 131.5, 114.3, 113.6, 100.6, 72.7, 55.6, 44.2, 34.1, 32.8, 24.2, 20.1; HRESIMS *m/z* 305.1411 $[M + H]^+$ (calcd for $C_{17}H_{21}O_5$, 305.1389).

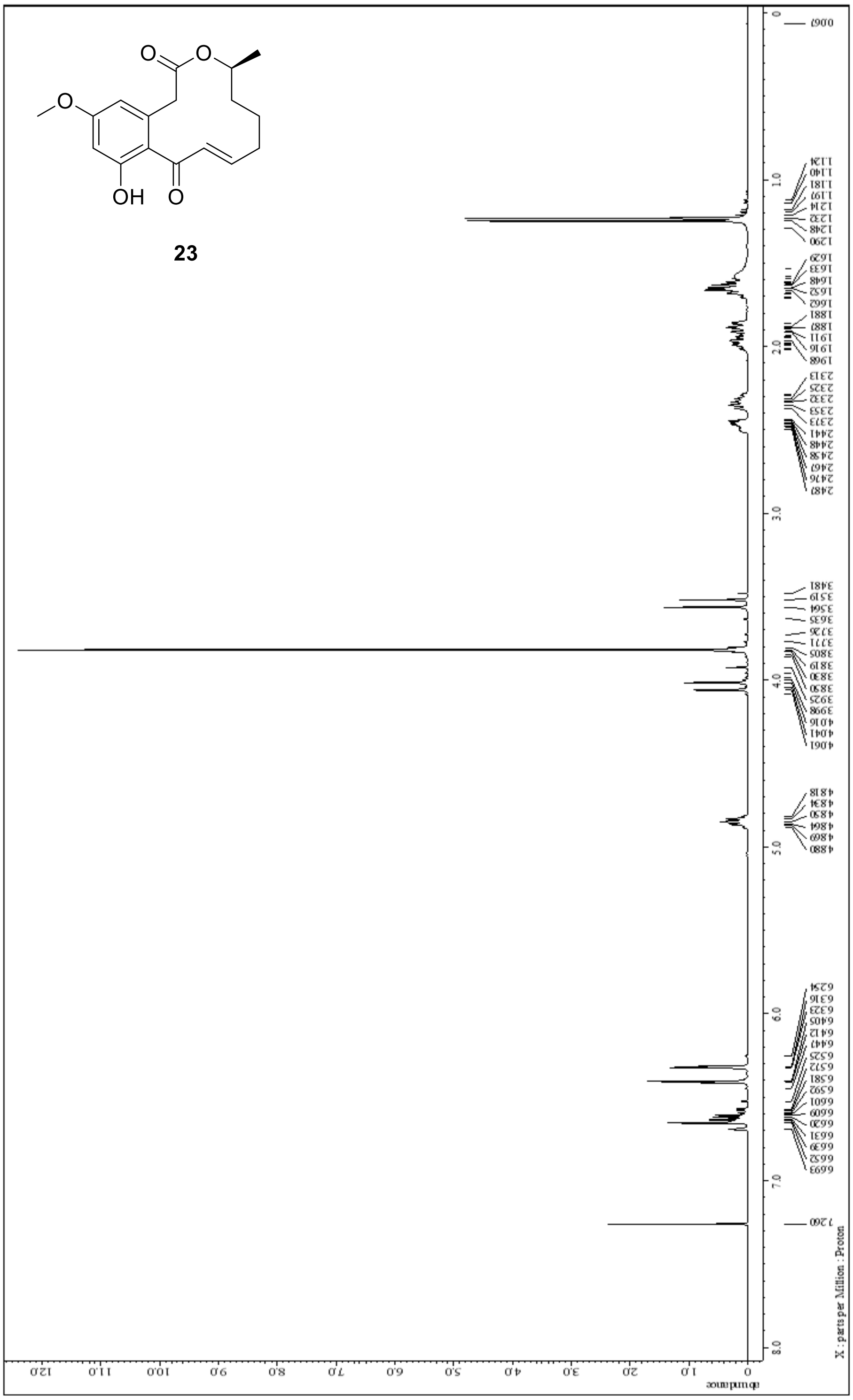
23
X : parts per Million : Proton
abundance

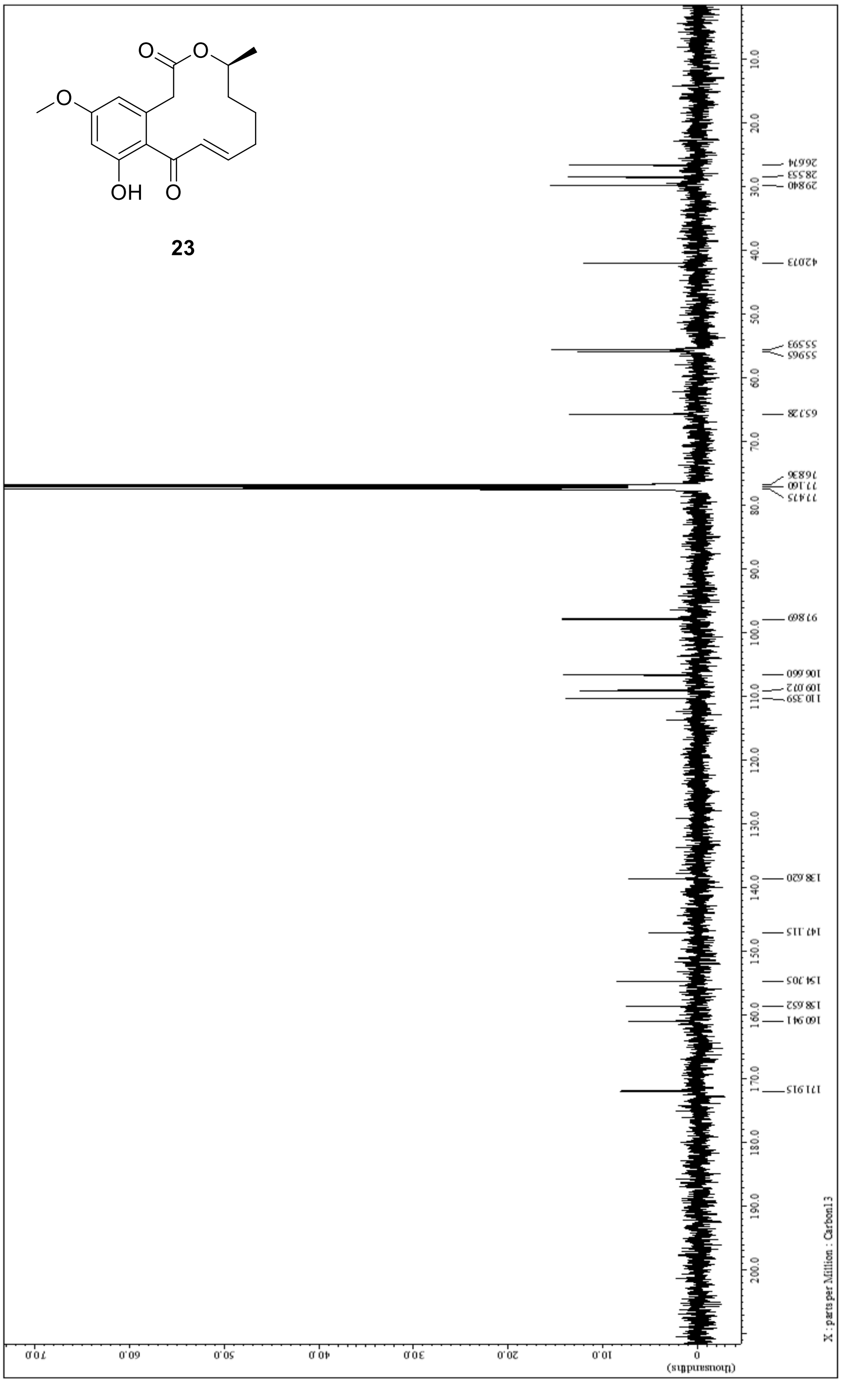
23
OH
26.674
28.553
29.840
42.073
55.593
55.965
65.728
76.836
77.160
77.415
97.869
106.660
109.072
110.359
138.620
147.115
154.305
158.652
160.941
171.915
X : parts per Million : Carbon13
(thousandths)

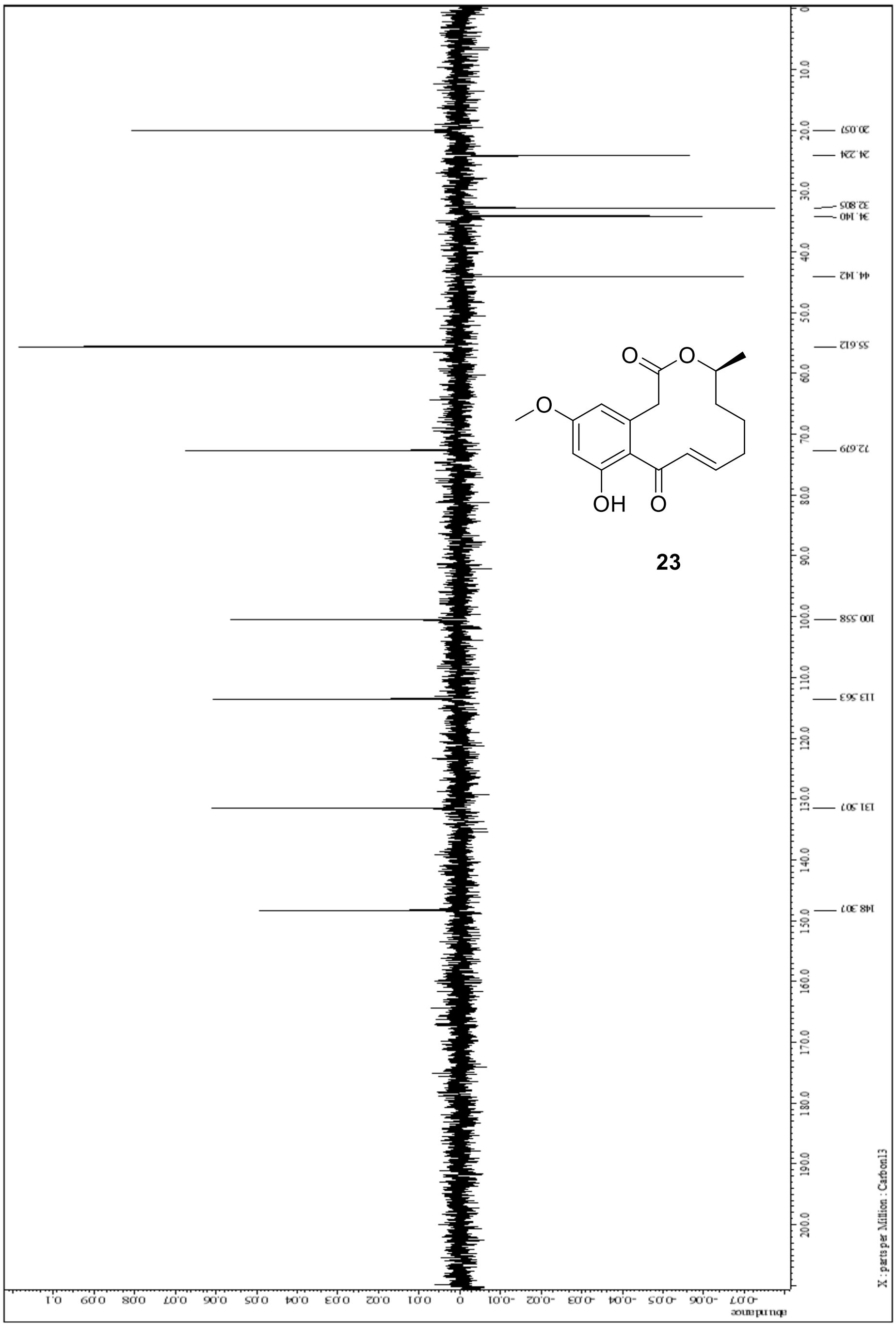

20.057
24.224
32.805
34.140
44.142
55.612
72.679
100.558
113.563
131.507
148.307
X : parts per Million : Carbon13
abundance
OH
23

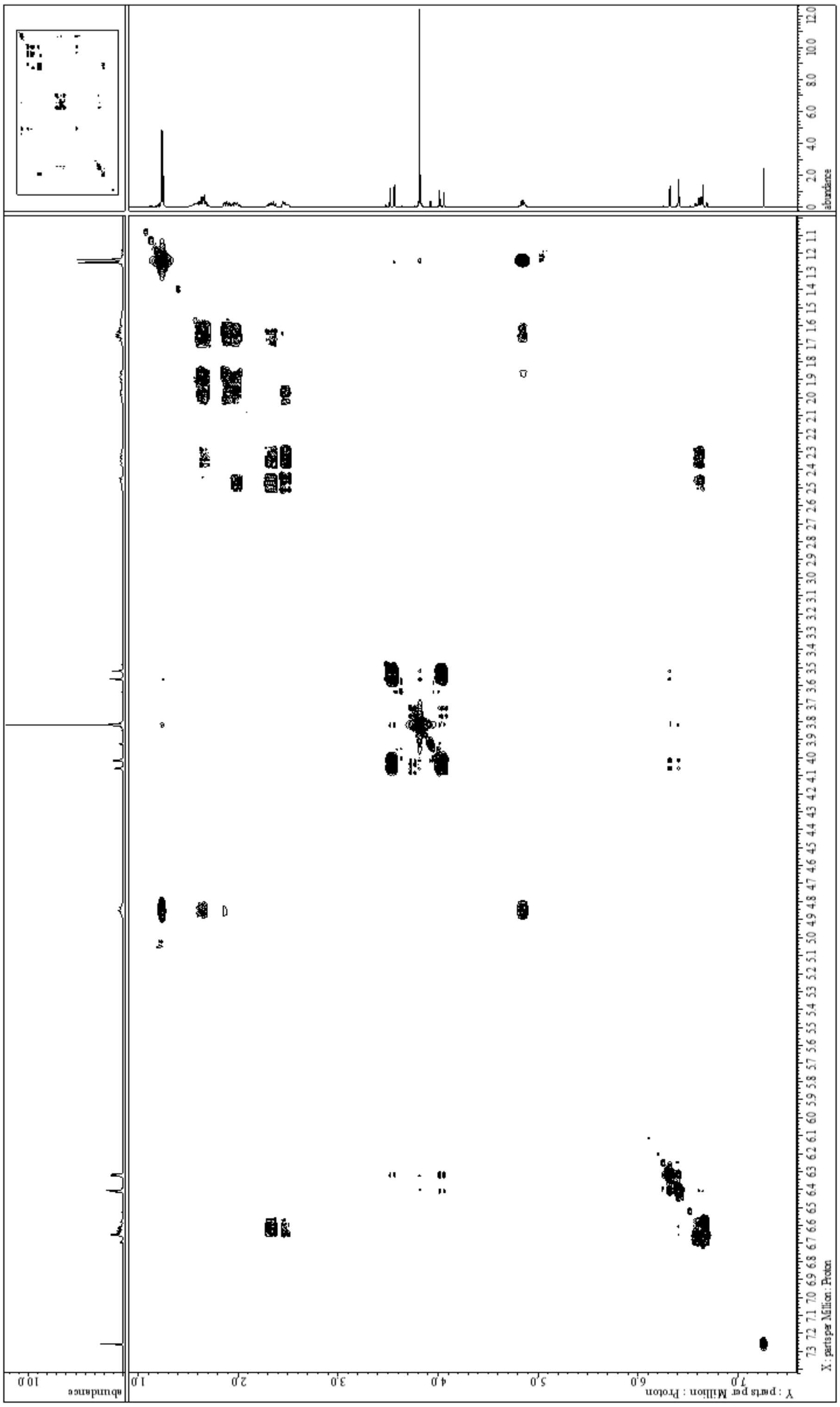
X : parts per Million : Proton
Y : parts per Million : Proton
abundance

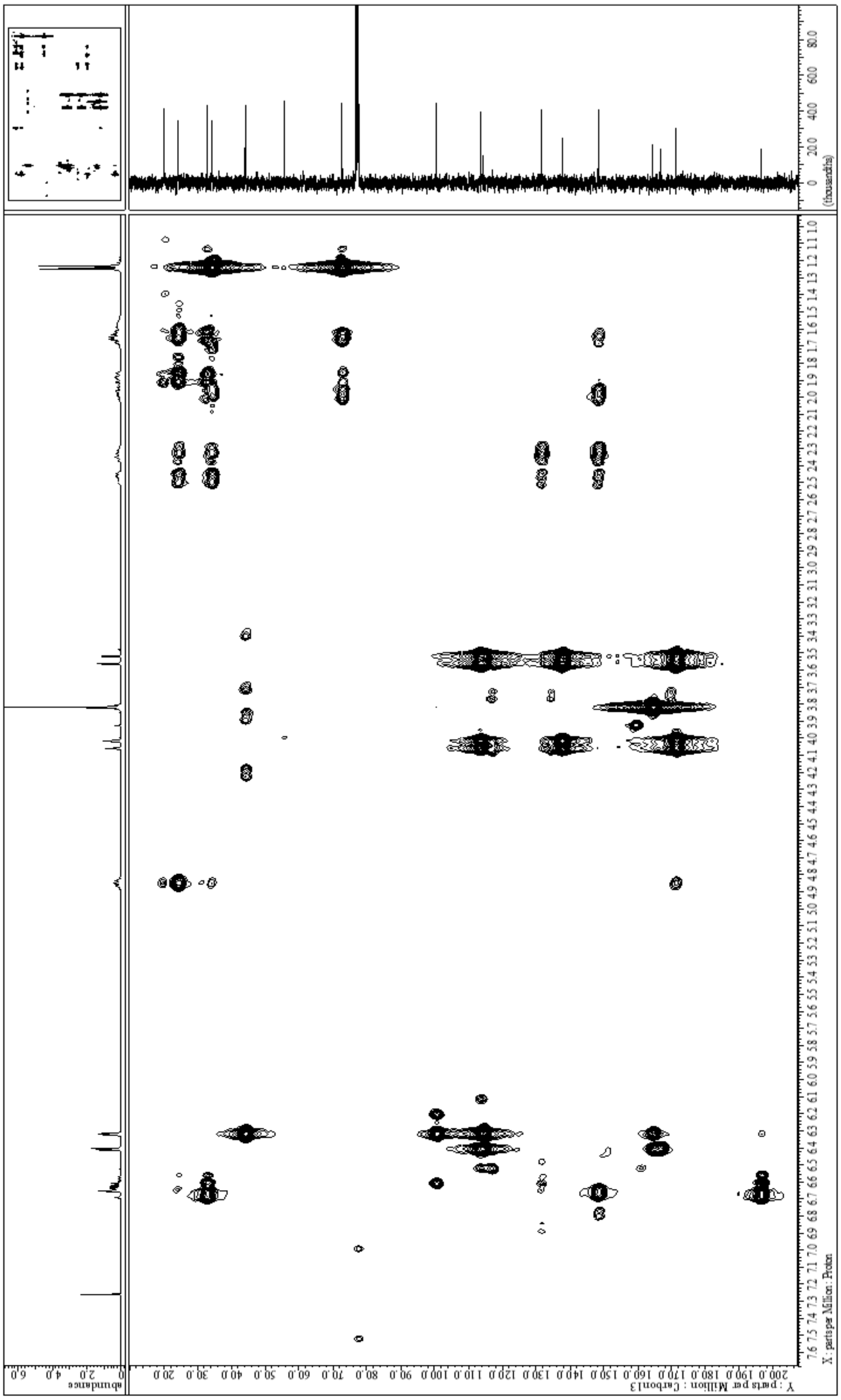
Y : parts per Million : Carbon13
X : parts per Million : Proton
abundance
(thousandths)

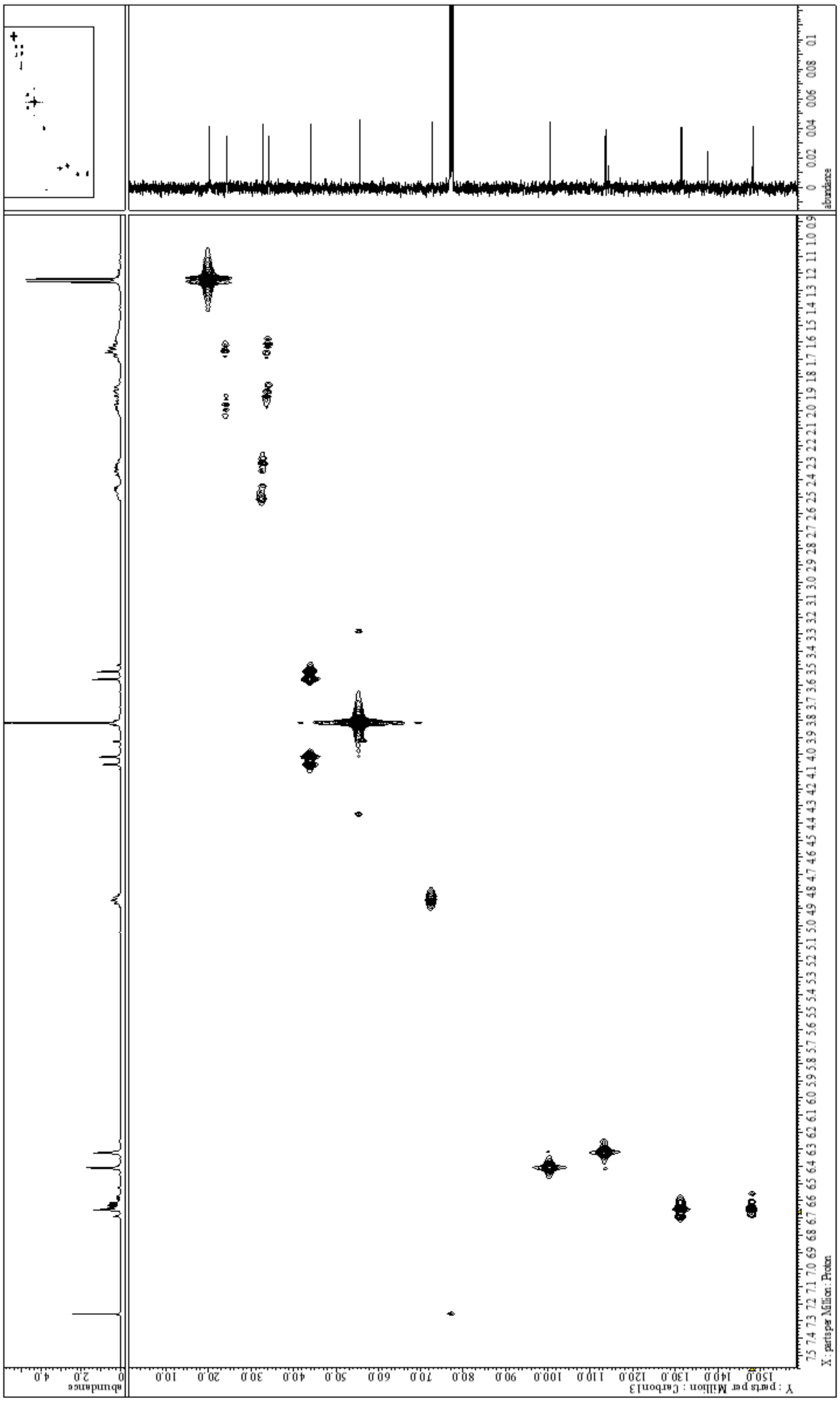

Figure S18. NMR spectrums of (*S*,*E*)-11-hydroxy-13-methoxy-4-methyl-4,5,6,7-tetrahydro-2*H*-benzo[*d*][1]oxacyclododecine-2,10(1*H*)-dione (**23**)
